# Genetic Insights Into Dietary Factors, Metabolic Traits and Myasthenia Gravis Risk: A Large‐Scale Two‐Sample Mendelian Randomization Study in European Populations

**DOI:** 10.1002/fsn3.70236

**Published:** 2025-05-26

**Authors:** Guoliang You, Meng Li, Minheng Zhang, Hongwei Liu, Xuan Chen, Haixia Fan

**Affiliations:** ^1^ Department of Neurology Taiyuan City Central Hospital, The Ninth Clinical Medical College of Shanxi Medical University Taiyuan Shanxi Province China; ^2^ Department of Gerontology The First People's Hospital of Jinzhong Jinzhong Shanxi Province China; ^3^ Department of Sleep Center First Hospital of Shanxi Medical University Taiyuan Shanxi Province China

**Keywords:** dietary factors, inverse‐variance weighted, Mendelian randomization, metabolic factors, myasthenia gravis

## Abstract

The impact of dietary factors and metabolic traits on the risk of myasthenia gravis (MG) is not well understood. This study utilized two‐sample Mendelian randomization (MR) to investigate the causal relationships between 16 dietary factors and 10 metabolic traits with MG risk. Using the inverse variance weighted (IVW) method, we identified significant causal associations and tested for heterogeneity using Cochran's *Q* test. The MR‐Egger intercept was used to assess horizontal pleiotropy, and the Mendelian Randomization Pleiotropy RESidual Sum and Outlier (MR‐PRESSO) framework was applied to detect and correct for potential outliers. Our analysis revealed that increased fresh fruit intake was associated with a reduced risk of MG (odds ratio [OR] = 0.023, 95% confidence interval [CI] = 0.001–0.683, *p* = 0.029). In contrast, higher body mass index (BMI) (OR = 2.696; 95% CI = 1.524–4.770; *p* < 0.001), waist circumference (OR = 2.995, 95% CI = 1.457–6.156, *p* = 0.003), hypothyroidism (OR = 1.337, 95% CI = 1.033–1.730, *p* = 0.027), and hyperthyroidism (OR = 2.240, 95% CI = 1.001–4.683, *p* < 0.001) were positively associated with MG risk. After adjusting for the false discovery rate (FDR), BMI and hyperthyroidism remained significantly linked to MG. No significant associations were found between MG and the other 15 dietary factors or 6 metabolic traits. These findings highlight the potential nutritional and metabolic pathways that may contribute to MG risk, suggesting that dietary interventions, particularly increasing fruit intake, and managing metabolic factors like BMI and thyroid health could play a role in the prevention and management of MG.

## Graphical abstract

This Mendelian randomization study reveals that genetically predicted higher fresh fruit intake significantly reduces MG risk (OR = 0.023, *p* = 0.029), while elevated BMI, waist circumference, and thyroid dysfunction (hypothyroidism and hyperthyroidism) increase MG risk. The findings suggest that dietary modifications favoring fruit consumption and metabolic health management may help prevent MG. The study provides genetic evidence supporting lifestyle interventions in MG risk reduction.

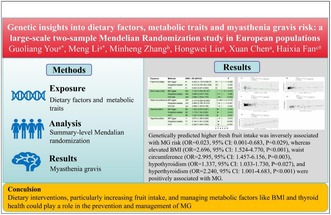

## 1 Introduction

Myasthenia Gravis (MG) is an autoimmune neuromuscular disorder characterized by muscle weakness and fatigue, primarily resulting from dysfunction of acetylcholine receptors at the neuromuscular junction (Gilhus 2016). Although MG affects both sexes, it is notably more prevalent in women, particularly among younger and older populations. Despite significant therapeutic advancements, the precise etiology of MG remains incompletely understood, with genetic susceptibility, immune dysregulation, and environmental factors all playing pivotal roles in its pathogenesis (Wang, Zhou, et al. 2024; Avidan et al. 2014; Lopomo and Berrih‐Aknin 2017). While the influence of environmental triggers, such as infections and medications, in the development of MG has been well explored, there remains a dearth of research examining the impact of dietary habits and metabolic factors. Elucidating these modifiable factors could open novel avenues for the prevention and management of MG, especially given the profound impact of the disease on patients' quality of life.

Dietary habits have long been associated with the pathogenesis of autoimmune diseases, primarily through their effects on systemic inflammation and immune function (Xiao et al. 2024; Choi et al. 2017; Gioia et al. 2020). For instance, omega‐3 fatty acids, antioxidants, and diets rich in fruits and vegetables are believed to possess anti‐inflammatory properties, while excessive intake of sugars, fats, and refined carbohydrates may exacerbate immune responses (Simopoulos 2002; Sahoo et al. 2023; Di Giosia et al. 2022; Lapuente et al. 2019; Barrea et al. 2018; Ma et al. 2022; Malesza et al. 2021). Despite these associations, the causal relationship between dietary habits and MG remains unresolved. Mendelian randomization (MR) has emerged as a robust tool in genetic epidemiology for investigating causal relationships. By leveraging genetic variants as instrumental variables, MR effectively mitigates confounding and reverse causality biases commonly observed in observational studies (Zeitoun and El‐Sohemy 2023; Ference et al. 2021). In this study, we adopt a two‐sample MR approach to investigate genetic evidence supporting causal relationships between dietary habits and the risk of MG.

In addition to dietary habits, metabolic factors, including obesity, body mass index (BMI), lipid metabolism, and insulin resistance, have been implicated in the pathogenesis of autoimmune diseases, including MG. Metabolic disturbances may influence immune cell function and cytokine production, thus contributing to autoimmune inflammation (Hu et al. 2024; Wang, Huang, et al. 2024; Karczewski et al. 2021). While associations between components of metabolic syndrome and MG have been reported, evidence establishing a causal relationship remains limited (Chang et al. 2021; Hurtado Vázquez and González Valverde 2024; Shao et al. 2024). To address this gap, the current study employs a two‐sample MR approach to analyze the genetic effects of metabolic traits on the risk of MG.

MR is a powerful tool in genetic epidemiology, utilizing inherited genetic variants as instrumental variables to explore potential causal links between modifiable exposures and health outcomes (Sekula et al. 2016; Richmond and Davey Smith 2022). This method effectively overcomes two major limitations inherent in observational studies: residual confounding and reverse causality. The robustness of MR relies on Mendel's second law of independent assortment, which ensures that genetic instruments remain unaffected by environmental confounders due to random segregation during meiosis. This natural process of randomization parallels the experimental randomization used in clinical trials, allowing for unbiased estimation of causal effects (Birney 2022; Emdin et al. 2017; Richmond and Davey Smith 2022). The stability of genetic variation, which is established at conception and remains consistent throughout an individual's life, also protects against reverse causation, where diseases could otherwise influence exposure variables (Ference et al. 2021; Lee and Lim 2019). These characteristics allow MR to provide more reliable causal estimates than traditional epidemiological methods, particularly when examining complex, bidirectional relationships between metabolic processes, dietary factors, and MG. Through the analysis of high‐quality genome‐wide association study (GWAS) summary data, our study rigorously explores the causal pathways linking dietary habits and metabolic traits to the risk of MG. These MR analyses not only deepen our understanding of the disease's etiology but also have the potential to uncover novel therapeutic strategies and opportunities for targeted prevention.

## 2 Methods

### 2.1 Study Design

**FIGURE 1:**
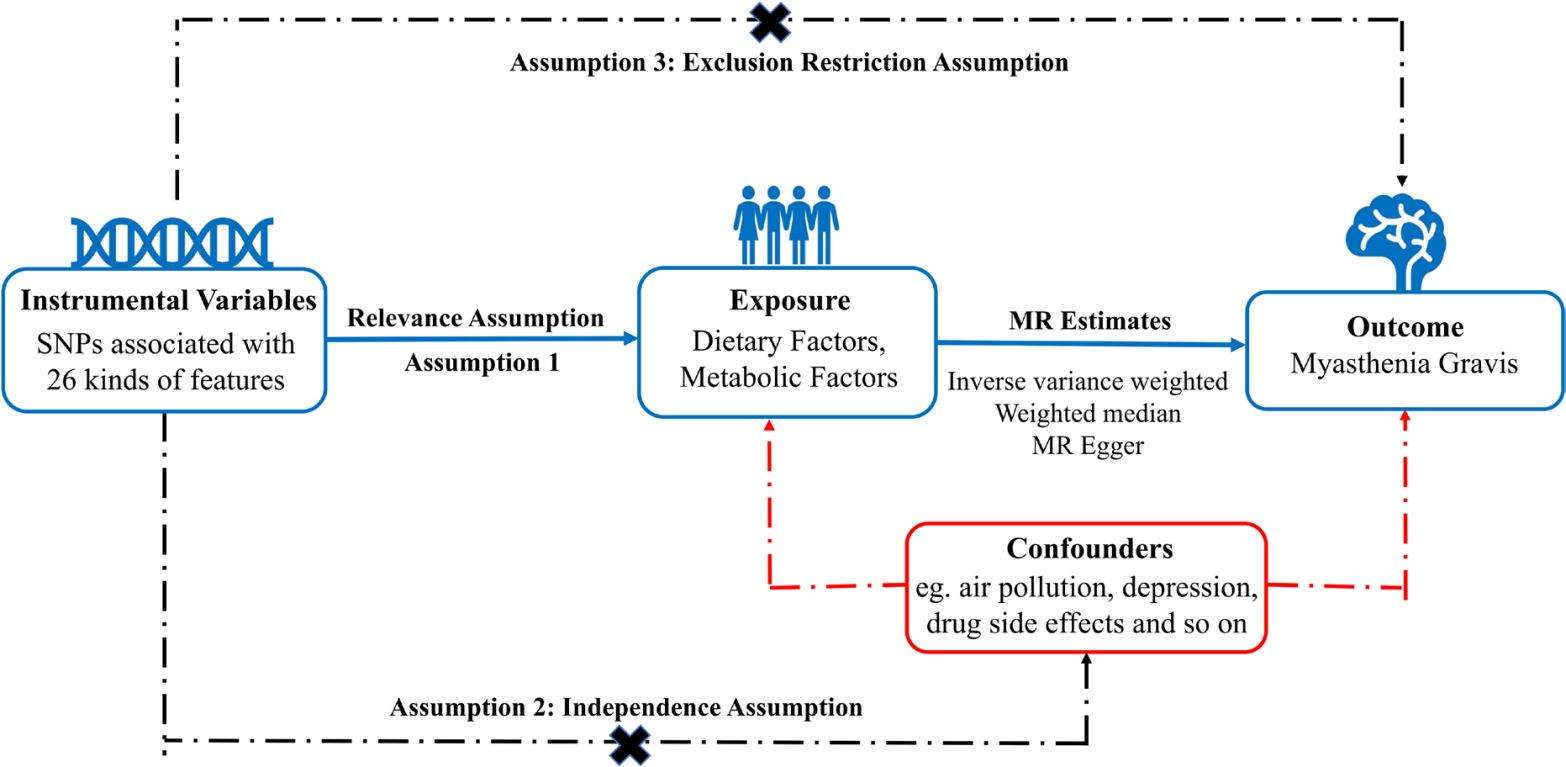
The process of present MR analyses is shown in flow chart. Assumption 1: The IVs selected for this study should demonstrate a significant association with dietary factors and metabolic factors. Assumption 2: The IVs chosen for present study are required to have no significant associations with other potential confounding factors. Assumption 3: The IVs utilized in present study do not have any independent causal pathways leading to myasthenia gravis other than through dietary factors and metabolic factors. IVs, instrumental variable; MR, Mendelian randomization; SNPs, single‐nucleotide polymorphisms.

This study utilizes a two‐sample MR framework to explore the potential causal relationships between dietary and metabolic factors and the risk of MG. By leveraging genetic variants as instrumental variables (IVs), MR effectively addresses the confounding and reverse causality biases that are inherent in traditional observational studies (Bowden and Holmes 2019; Burgess et al. 2013). The MR design in this study adheres to stringent validity criteria: (1) a strong correlation between the instrumental variable and the exposure, ensuring methodological robustness; (2) the instrumental variable is independent of potential confounders, preserving the integrity of the analysis; and (3) the effect of the instrumental variable on the outcome is mediated exclusively through the exposure, eliminating alternative causal pathways. This approach is in accordance with the guidelines outlined in Strengthening the Reporting of Observational Studies in Epidemiology using MR (STROBE‐MR) (Skrivankova et al. 2021). A two‐sample MR analysis was conducted to examine the causal relationships between dietary and metabolic factors and MG (Figure 1). As the study only involved data analysis without any identifiable personal information, the ethical board of Taiyuan Central Hospital granted a waiver of informed consent.

### 2.2 Selection Criteria for Dietary Factors and Metabolic Traits

The dietary factors, numbering 16 in total, and the metabolic traits, amounting to 10, incorporated in this Mendelian randomization analysis were meticulously chosen based on two principal criteria. Firstly, their biological credibility in immune modulation or neuromuscular pathophysiology was considered. Secondly, the accessibility of robust GWAS data was taken into account. Regarding the dietary factors, preference was given to those foods and nutrients that possess well‐established immunological characteristics. These substances have been previously linked to autoimmune diseases; for instance, anti‐inflammatory fruits and pro‐inflammatory processed meats fall into this category. In the case of metabolic traits, their selection was directed by their clinical significance in relation to autoimmune mechanisms, such as the inflammation associated with obesity and thyroid malfunction, as well as their relevance to neuromuscular function. This assessment was based on high‐quality GWAS sources, which included the IEU OpenGWAS, UK Biobank, and FinnGen datasets. By doing so, the validity of our Mendelian randomization methodology was ensured. This comprehensive selection strategy offers a sturdy basis for making causal inferences.

### 2.3 Dietary Exposure Assessment

Dietary data were gathered using a touchscreen‐based questionnaire that was specifically designed and implemented within the Medical Research Council Integrative Epidemiology Unit (MRC‐IEU) dataset, sourced from the UK Biobank. (1) The questionnaire was carefully crafted to obtain detailed information on the frequency of consumption of a wide variety of food and beverage items, such as tea, pork, processed meats, fish or liver oil supplements, and other key dietary components. This design ensured the standardization and consistency of data collection across large‐scale epidemiological cohorts. (2) The validity and reliability of this instrument have undergone rigorous evaluation in independent studies, which compared it with traditional dietary assessment methods, including 24‐h dietary recalls and food frequency questionnaires, as well as with biomarker‐based assessments such as urinary nitrogen excretion and plasma concentrations of micronutrients like vitamin C and carotenoids (Cole et al. 2022; Bradbury et al. 2018). These evaluations revealed strong correlations with biochemical markers and successfully replicated findings from more intensive, small‐scale dietary assessments, thus further reinforcing the robustness of the tool. (3) Digital administration confers distinct methodological advantages, including the mitigation of interviewer bias, optimized data capture efficiency, and enhanced scalability for large‐scale epidemiological research. This approach additionally permits seamless data linkage, facilitating integrative analyses that examine dietary behaviors in relation to genomic, environmental, and phenotypic datasets. Consistent with GWAS best practices, we implemented comprehensive quality control measures including phenotypic harmonization and multivariable adjustment for key confounders (age, sex, energy intake). This methodological rigor both attenuates measurement bias and enhances our ability to detect diet‐associated genetic variants. The resultant data meet the stringent validity requirements for Mendelian randomization inference.

### 2.4 Genome‐Wide Association Study Summary Data for Exposures and Outcomes

The summary data for the GWAS exposures and outcomes were sourced from publicly available repositories. Genetic variants associated with dietary factors (e.g., beef, pork, poultry, oily fish, non‐oily fish, processed meats, tea, water, alcohol consumption frequency, bread, cheese, cereal, dried fruit, fresh fruit, cooked vegetables, and salad/raw vegetables) and metabolic factors (e.g., BMI, waist circumference, hypothyroidism, hyperthyroidism, glycated hemoglobin, fasting insulin, fasting glucose, triglycerides, high‐density lipoprotein [HDL] cholesterol, low‐density lipoprotein [LDL] cholesterol) were obtained from the IEU Open GWAS project. This project encompasses extensive GWAS meta‐analyses and summary statistics from the UK Biobank and the MRC Integrated Epidemiology Department. Genetic associations with Myasthenia Gravis were derived from the FinnGen version 6 dataset, which includes 217,288 individuals of European ancestry, with cases identified based on diagnostic codes from the International Classification of Diseases (ICD) versions 8, 9, and 10.

### 2.5 Selection of Instrumental Variables

In this study, careful attention was given to the selection of IVs to ensure the rigor, validity, and robustness of the MR analysis. We incorporated a set of genetic variants associated with 26 distinct traits, identified as potential risk factors for MG. The selection process adhered to three core principles essential for satisfying the fundamental assumptions of MR: relevance, independence, and exclusion restriction. First, single nucleotide polymorphisms (SNPs) strongly associated with the exposure variables were identified using a stringent genome‐wide significance threshold (*p* < 5.0 × 10^−8^). This threshold was chosen to minimize the risk of false positives and to ensure robust associations between the SNPs and the exposures of interest (Birney 2022). To address potential linkage disequilibrium (LD), which could introduce bias due to correlated genetic variants, we applied an LD clustering procedure. SNPs with an *r* ^2^ value exceeding 0.001 within a 10,000 kb region were systematically excluded, ensuring the independence of the selected IVs (Hemani et al. 2018). The strength of the selected IVs was evaluated using the *F*‐statistic, calculated as *F* = *β* ^2^/SE^2^, where *β* represents the effect size of the SNP on the exposure, and SE denotes the corresponding standard error. An *F*‐statistic greater than 10 indicates a strong instrument, thereby minimizing the risk of weak instrument bias (Pierce et al. 2011). This criterion ensured that the genetic variants used in the analysis had sufficient statistical power to reliably estimate causal relationships. Additionally, the PhenoScanner V2 tool was utilized to assess potential pleiotropic effects (http://www.phenoscanner.medschl.cam.ac.uk/). SNPs associated with confounding factors or directly linked to the outcome were excluded, ensuring that the IVs influenced the outcome solely through the exposures of interest (Kamat et al. 2019).

### 2.6 Statistical Analysis

To investigate the causal relationships between dietary factors, metabolic factors, and MG, we employed a robust statistical framework based on MR principles. The primary analytical approach, inverse variance weighting (IVW), calculates a weighted average of causal estimates derived from individual genetic variants. This method operates under the assumption that all instrumental variables satisfy the three core MR assumptions: (1) relevance (strong correlation with the exposure), (2) independence (no association with confounding factors), and (3) exclusion restriction (no direct effect on the outcome, except through the exposure) (Birney 2022). IVW estimates are computed using weighted linear regression, where the weights are inversely proportional to the variance of the causal estimates. To address potential pleiotropy and heterogeneity, we performed several sensitivity analyses in conjunction with the IVW method. Assuming that at least 50% of the instruments are valid, we employed a weighted median estimator to yield consistent causal estimates (Burgess et al. 2017; Bowden et al. 2019). This approach is particularly robust to the presence of invalid instruments, as it relies on the median of the causal estimates, which is less sensitive to outliers. Furthermore, the MR Egger regression method was utilized to assess and correct for directional pleiotropy, providing unbiased estimates under the InSIDE (instrument strength independent of direct effect) assumption (Bowden et al. 2015). An MR Egger intercept test was conducted to detect pleiotropy, with a non‐significant intercept (*p* > 0.05) suggesting no systematic bias. To further strengthen the robustness of our results, we incorporated the MR‐PRESSO (Mendelian Randomization Pleiotropy RESidual Sum and Outlier) method, which identifies and corrects outlier SNPs that may distort causal estimates (Verbanck et al. 2018). This method is especially useful for detecting and adjusting for horizontal pleiotropy, ensuring that causal estimates are not biased by invalid instruments. Cochran's *Q* statistic was applied to assess heterogeneity among the instruments, with a *p*‐value < 0.05 indicating the need for a random‐effects IVW model to account for variability across SNPs. In the presence of significant heterogeneity, we also employed the penalized weighted median and weighted mode estimators, which provide reliable causal estimates even when subsets of instruments are invalid (Hartwig et al. 2017; Bowden and Holmes 2019). To confirm the stability of our findings, we conducted a leave‐one‐out analysis, systematically excluding each SNP to evaluate its impact on the overall causal estimate. This approach facilitated the detection of potential outliers and affirmed the robustness of our results. Causal relationships were considered statistically significant if the IVW *p*‐value was < 0.05, and if the directional estimates from the IVW, MR Egger, and weighted median methods were consistent. Additionally, the MR Egger intercept test was required to show no evidence of pleiotropy (*p* > 0.05). All statistical analyses were performed using the Two‐Sample MR package (version 4.3.3) in R, with a significance threshold set at *p* < 0.05.

## 3 Results

### 3.1 IVs For Dietary Factors and Metabolic Factors on MG

Table 1 presents an overview of the GWAS data sources utilized in this study. Table S1 provides a comprehensive list of the SNPs extracted for each exposure, accompanied by their respective F‐statistics. The number of SNPs corresponding to each exposure is as follows: beef consumption (14), pork consumption (13), poultry consumption (7), oily fish consumption (61), non‐oily fish consumption (11), processed meat consumption (23), tea consumption (40), water consumption (37), alcohol consumption frequency (95), bread consumption (27), cheese consumption (61), cereal consumption (39), dried fruit consumption (41), fresh fruit consumption (41), cooked vegetable consumption (17), salad/raw vegetable consumption (18), body mass index (BMI) (424), waist circumference (531), hypothyroidism (68), hyperthyroidism (11), HDL cholesterol (326), LDL cholesterol (156), triglycerides (284), glycated hemoglobin (22), fasting insulin (25), and fasting glucose (57). All SNPs incorporated in the analysis exhibited F‐statistics exceeding the threshold of 10, with values spanning from 10 to 25,777. These findings suggest that the SNPs analyzed demonstrate substantial predictive capability for dietary and metabolic factors within the framework of MR.

**TABLE 1:** Details of the GWAS included in the two‐sample Mendelian randomization study.

Trait	GWAS ID	Sample size	Number of SNPs	Consortium	Population	Year	Author
*Dietary habits*							
Beef intake	ukb‐b‐2862	461,053	9,851,867	MRC‐IEU	European	2018	Ben Elsworth
Pork intake	ukb‐b‐5640	460,162	9,851,867	MRC‐IEU	European	2018	Ben Elsworth
Poultry intake	ukb‐b‐8006	461,900	9,851,867	MRC‐IEU	European	2018	Ben Elsworth
Oily fish intake	ukb‐b‐2209	460,443	9,851,867	MRC‐IEU	European	2018	Ben Elsworth
Non‐oily fish intake	ukb‐b‐17,627	460,880	9,851,867	MRC‐IEU	European	2018	Ben Elsworth
Processed meat intake	ukb‐b‐6324	461,981	9,851,867	MRC‐IEU	European	2018	Ben Elsworth
Tea intake	ukb‐b‐6066	447,485	9,851,867	MRC‐IEU	European	2018	Ben Elsworth
Water intake	ukb‐b‐14,898	427,588	9,851,867	MRC‐IEU	European	2018	Ben Elsworth
Alcohol intake frequency	ukb‐b‐5779	462,346	9,851,867	MRC‐IEU	European	2018	Ben Elsworth
Bread intake	ukb‐b‐11,348	452,236	9,851,867	MRC‐IEU	European	2018	Ben Elsworth
Cheese intake	ukb‐b‐1489	451,486	9,851,867	MRC‐IEU	European	2018	Ben Elsworth
Cereal intake	ukb‐b‐15,926	441,640	9,851,867	MRC‐IEU	European	2018	Ben Elsworth
Dried fruit intake	ukb‐b‐16,576	421,764	9,851,867	MRC‐IEU	European	2018	Ben Elsworth
Fresh fruit intake	ukb‐b‐3881	446,462	9,851,867	MRC‐IEU	European	2018	Ben Elsworth
Cooked vegetable intake	ukb‐b‐8089	448,651	9,851,867	MRC‐IEU	European	2018	Ben Elsworth
Salad/raw vegetable intake	ukb‐b‐1996	435,435	9,851,867	MRC‐IEU	European	2018	Ben Elsworth
*Metabolic factor*							
Body mass index	ukb‐b‐2303	454,884	9,851,867	MRC‐IEU	European	2018	Ben Elsworth
Waist circumference	ukb‐b‐9405	462,166	9,851,867	MRC‐IEU	European	2018	Ben Elsworth
Hypothyroidism	ebi‐a‐GCST90018862	410,141	24,138,872	UK Biobank	European	2021	Saori Sakaue
Hyperthyroidism	ebi‐a‐GCST90018860	460,499	24,189,279	UK Biobank	European	2021	Saori Sakaue
HDL cholesterol	ieu‐b‐109	403,943	12,321,875	UK Biobank	European	2020	Richardson Tom
LDL cholesterol	ieu‐b‐110	440,546	12,321,875	UK Biobank	European	2020	Richardson Tom
Triglycerides	ieu‐b‐111	441,016	12,321,875	UK Biobank	European	2020	Richardson Tom
Glycated hemoglobin	ebi‐a‐GCST90002244	146,806	30,649,064	GWAS‐Meta	European	2021	Chen J
Fasting insulin	ebi‐a‐GCST90002238	151,013	29,664,438	GWAS‐Meta	European	2021	Chen J
Fasting glucose	ebi‐a‐GCST90002232	200,622	31,008,728	GWAS‐Meta	European	2021	Chen J
Myasthenia gravis	finn‐b‐G6_MYASTHENIA	217,288	16,380,458	FinnGen	European	2021	NA

Abbreviations: GWAS, Genome‐Wide Association Study; IMGGC, International Myasthenia Gravis Genomics Consortium; SNPs, single nucleotide polymorphisms.

### 3.2 Two‐Sample MR Analysis

**FIGURE 2:**
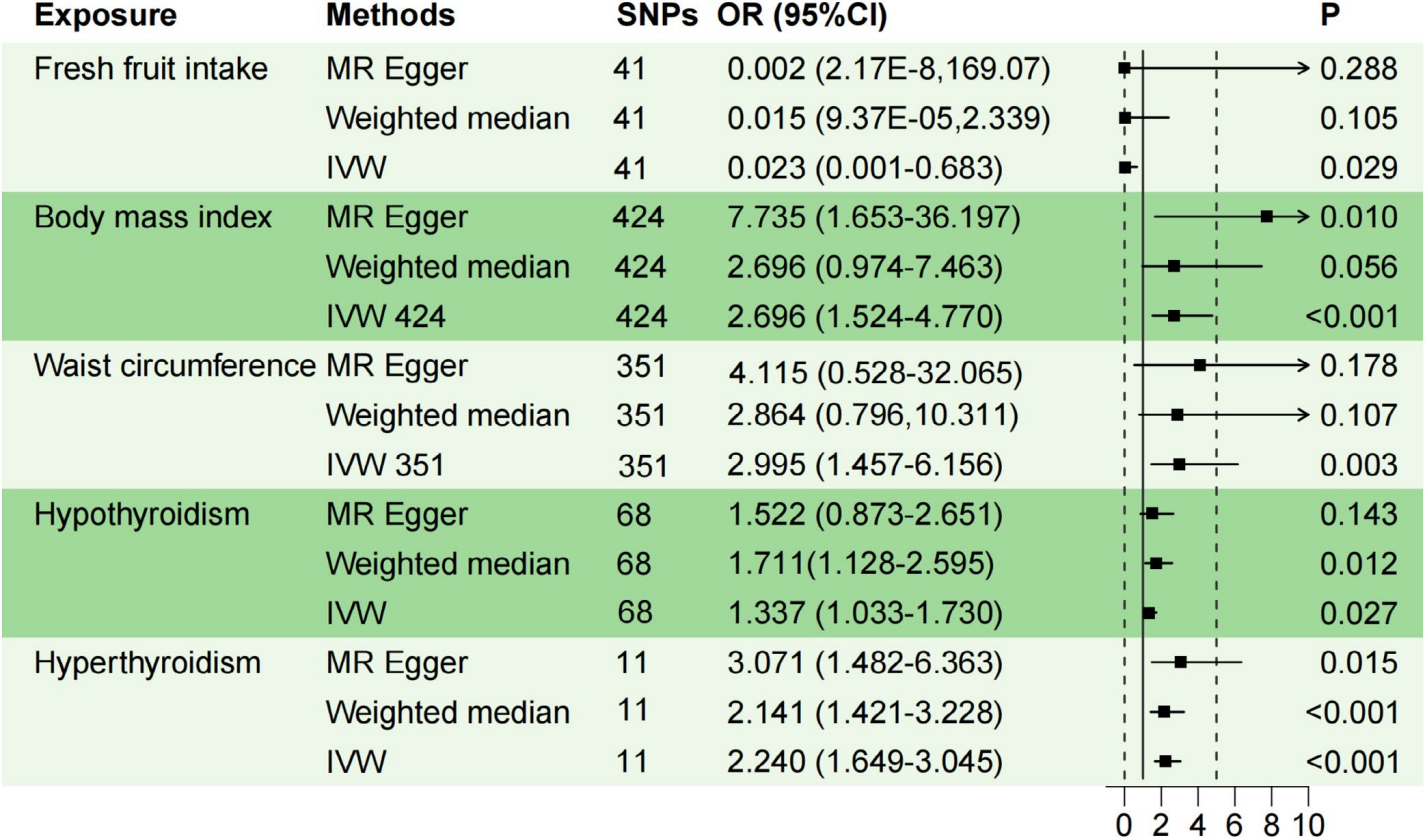
Forest plot for the causal effects for body mass index, waist circumference, hypothyroidism, hyperthyroidism, fresh fruit intake, and myasthenia gravis. summary of the MR estimates derived from the IVW, MR‐Egger, and weighted median methods. C, confidence interval; IVW, inverse variance weighted; MR, Mendelian randomization; OR, odds ratio.

**FIGURE 3:**
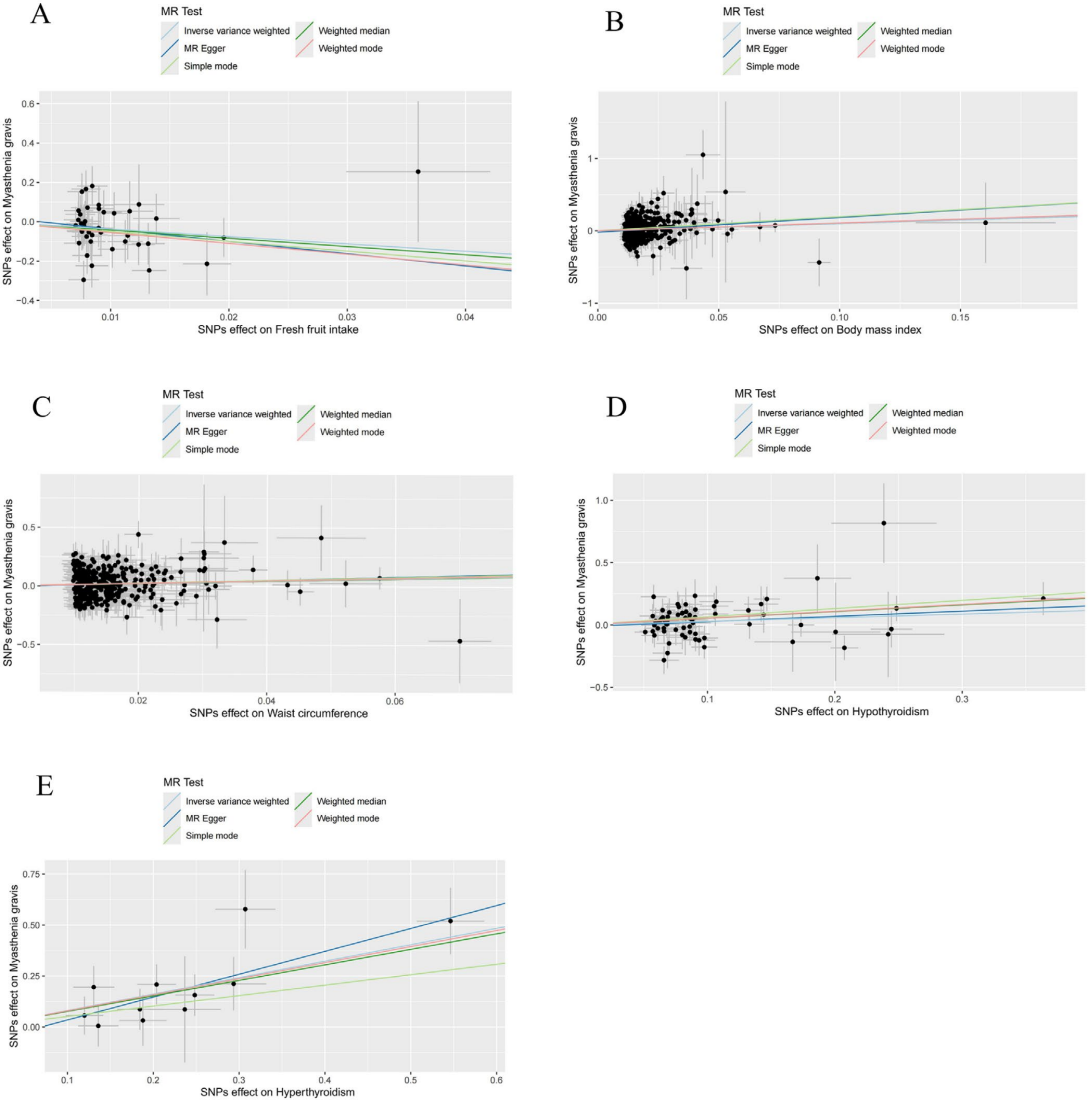
Scatter plots of fresh fruit intake (A), body mass index (B), waist circumference (C), hypothyroidism (D), hyperthyroidism (E) and myasthenia gravis. MR, Mendelian randomization; SNPs, single‐nucleotide polymorphisms.

A two‐sample MR analysis was conducted to explore the causal relationships between dietary and metabolic factors and the risk of MG. The IVW method identified significant associations for several exposures. Specifically, a higher BMI was strongly associated with an increased risk of MG (IVW: OR = 2.696; 95% CI = 1.524–4.770; *p* < 0.001). Similarly, a greater waist circumference was linked to an elevated risk of MG (IVW: OR = 2.995; 95% CI = 1.457–6.156; *p* = 0.003) (Figure 2). Moreover, both hypothyroidism (IVW: OR = 1.337; 95% CI = 1.033–1.730; *p* = 0.027) and hyperthyroidism (IVW: OR = 2.240; 95% CI = 1.649–3.045; *p* < 0.001) were positively correlated with an increased risk of MG. In contrast, a higher intake of fresh fruit was associated with a protective effect against MG (IVW: OR = 0.023; 95% CI = 0.001–0.683; *p* = 0.029) (Figure 2). After adjusting for the false discovery rate, the associations between BMI, hyperthyroidism, and MG risk remained significant. No causal relationships were observed for other exposures, including beef intake, pork intake, poultry intake, oily fish intake, non‐oily fish intake, processed meat intake, tea intake, water intake, alcohol intake frequency, bread intake, cheese intake, cereal intake, dried fruit intake, cooked vegetable intake, salad/raw vegetable intake, HDL cholesterol, LDL cholesterol, triglycerides, glycated hemoglobin, fasting insulin, or fasting glucose. Figure 3 presents scatter plots of the five analytical methods (IVW, MR Egger, weighted median, simple mode, and weighted mode) for exposures significantly associated with MG risk. The causal effect estimates derived from MR Egger, weighted median, and weighted mode analyses were consistent in both magnitude and direction with those obtained using the IVW method, as detailed in Table S2.

### 3.3 Heterogeneity, Pleiotropy, and Sensitivity Analysis

Table 2 summarizes the results of the heterogeneity, pleiotropy, and sensitivity analyses. Sensitivity analyses, including Cochran's *Q* statistic, the MR Egger intercept, the MR‐PRESSO global test, and leave‐one‐out analysis, consistently affirmed the reliability of our two‐sample MR results. To evaluate heterogeneity, Cochran's *Q* statistic was applied using both the IVW and MR Egger methods. Significant heterogeneity was observed for grain intake (*p* = 0.043, *Q* = 54.122) and LDL cholesterol (*p* = 0.044, *Q* = 186.218), prompting the use of the IVW method with multiplicative random effects for these exposures. With respect to pleiotropy, neither the MR‐PRESSO test nor the MR Egger intercept revealed significant horizontal pleiotropy, thereby further enhancing the robustness of our findings, as detailed in Table 2. Additionally, the leave‐one‐out analysis confirmed that no individual SNP exerted a disproportionate influence on the overall causal estimates, thereby reinforcing the stability of our conclusions (Figure S1).

**TABLE 2:** The heterogeneity and pleiotropy analysis in the two‐sample Mendelian randomization study.

Exposure	Outcome	Method	Heterogeneity		Horizontal pleiotropy		MR PRESSO	
			*Q*	*Q*_*p*	MRE intercept	*p*	Qutliers	*p*
Beef intake	Myasthenia gravis	MR‐Egger	10.945	0.534	−0.267	0.154	0	0.461
		IVW	13.265	0.428				
Pork intake	Myasthenia gravis	MR‐Egger	11.613	0.393	−0.102	0.592	0	0.465
		IVW	11.934	0.451				
Poultry intake	Myasthenia gravis	MR‐Egger	6.986	0.222	0.551	0.301	0	0.279
		IVW	8.840	0.183				
Oily fish intake	Myasthenia gravis	MR‐Egger	77.214	0.056	0.038	0.567	0	0.095
		IVW	77.648	0.062				
Non‐oily fish intake	Myasthenia gravis	MR‐Egger	12.553	0.184	0.189	0.324	0	0.173
		IVW	14.071	0.170				
Processed meat intake	Myasthenia gravis	MR‐Egger	21.498	0.429	−0.017	0.884	0	0.495
		IVW	21.520	0.489				
Tea intake	Myasthenia gravis	MR‐Egger	36.319	0.547	−0.019	0.614	0	0.621
		IVW	36.578	0.581				
Water intake	Myasthenia gravis	MR‐Egger	35.480	0.446	0.025	0.630	0	0.578
		IVW	35.719	0.482				
Alcohol intake frequency	Myasthenia gravis	MR‐Egger	82.256	0.780	0.030	0.391	0	0.734
		IVW	82.997	0.784				
Bread intake	Myasthenia gravis	MR‐Egger	21.238	0.679	−0.026	0.785	0	0.593
		IVW	21.314	0.726				
Cheese intake	Myasthenia gravis	MR‐Egger	63.718	0.314	−0.035	0.582	0	0.374
		IVW	64.049	0.336				
Cereal intake	Myasthenia gravis	MR‐Egger	53.832	0.036	0.040	0.658	0	0.093
		IVW	54.122	0.043				
Dried fruit intake	Myasthenia gravis	MR‐Egger	38.385	0.498	0.071	0.334	0	0.564
		IVW	39.341	0.500				
Fresh fruit intake	Myasthenia gravis	MR‐Egger	41.467	0.364	0.025	0.654	0	0.478
		IVW	41.684	0.397				
Cooked vegetable intake	Myasthenia gravis	MR‐Egger	18.116	0.257	0.290	0.342	0	0.266
		IVW	19.276	0.255				
Salad/raw vegetable intake	Myasthenia gravis	MR‐Egger	22.840	0.118	0.172	0.296	0	0.148
		IVW	24.504	0.106				
Body mass index	Myasthenia gravis	MR‐Egger	433.828	0.335	−0.021	0.151	0	0.348
		IVW	435.961	0.321				
Waist circumference	Myasthenia gravis	MR‐Egger	355.842	0.389	−0.005	0.746	0	0.465
		IVW	355.950	0.402				
Hypothyroidism	Myasthenia gravis	MR‐Egger	73.584	0.244	−0.015	0.607	0	0.241
		IVW	73.882	0.264				
Hyperthyroidism	Myasthenia gravis	MR‐Egger	6.256	0.714	−0.078	0.374	0	0.421
		IVW	7.132	0.713				
HDL cholesterol	Myasthenia gravis	MR‐Egger	327.066	0.442	−0.018	0.064	0	0.479
		IVW	330.565	0.404				
LDL cholesterol	Myasthenia gravis	MR‐Egger	185.029	0.045	−0.015	0.321	0	0.054
		IVW	186.218	0.044				
Triglycerides	Myasthenia gravis	MR‐Egger	281.525	0.497	0.014	0.166	2	0.602
		IVW	283.454	0.481				
Glycated hemoglobin	Myasthenia gravis	MR‐Egger	20.799	0.409	0.006	0.919	0	0.568
		IVW	20.810	0.471				
Fasting insulin	Myasthenia gravis	MR‐Egger	32.896	0.083	0.054	0.585	0	0.111
		IVW	33.335	0.097				
Fasting glucose	Myasthenia gravis	MR‐Egger	42.361	0.894	−0.012	0.644	0	0.846
			42.577	0.907				

Abbreviations: EA, effect allele; HDL, high density lipoprotein; LDL, low density lipoprotein; OA, other allele; SE, standard error; SNPs, single nucleotide polymorphisms.

## 4 Discussion

Recent advances in understanding the pathogenesis of MG have significantly expanded our knowledge, particularly regarding the complex interplay between genetic susceptibility, metabolic dysregulation, and environmental triggers. MG is an autoimmune disorder characterized by muscle weakness and fatigue, and it is increasingly recognized as a multifactorial condition shaped by the interaction between genetic predisposition and environmental factors. Emerging evidence suggests that fluctuations in metabolic factors, such as BMI, may exacerbate immune dysregulation, thereby contributing to disease progression (Gilhus et al. 2019; Narayanaswami et al. 2021). Furthermore, dietary patterns, especially those rich in anti‐inflammatory components, have been linked to the modulation of autoimmune responses, highlighting the potential role of nutrition in managing MG (Kariagina and Doseff 2022; Campmans‐Kuijpers and Dijkstra 2021; Schönenberger et al. 2021). In addition, specific genetic variants associated with MG susceptibility have been identified, particularly in the major histocompatibility complex (MHC) region, which plays a crucial role in immune system regulation (Howard 2018). Despite these insights, the causal relationships between dietary habits, metabolic health, and MG remain poorly understood. Unraveling these causal links could transform therapeutic strategies by offering novel approaches for mitigating disease severity and improving patient outcomes through targeted lifestyle interventions.

This investigation sought to elucidate the potential causal links between 26 distinct traits and MG. The results underscore several critical insights, particularly regarding the complex interplay between metabolic dysregulation, endocrine disturbances, and dietary influences in the pathophysiology of MG. Specifically, we observed robust positive correlations between MG and BMI, waist circumference, hypothyroidism, and hyperthyroidism, suggesting that both metabolic abnormalities and thyroid dysfunction may serve as contributory factors in the onset and progression of MG. Conversely, our analysis revealed a significant inverse relationship between the intake of fresh fruit and the incidence of MG, suggesting a potential protective role for specific dietary patterns in mitigating the disease. This finding further corroborates the expanding body of literature advocating for the modulation of immune responses through dietary interventions.

Our analysis identified significant associations between MG and BMI and waist circumference, highlighting the need for further exploration of these metabolic factors in the context of autoimmune diseases such as MG. The observed positive correlation between MG and BMI suggests that obesity may play a crucial role in the disease's pathogenesis. Obesity is a well‐established risk factor for a range of chronic conditions, including cardiovascular diseases, diabetes, and autoimmune disorders (Haidar and Horwich 2023; Frank et al. 2019). Elevated BMI is frequently linked with mild systemic inflammation, which can impair immune system function (Keirns et al. 2023). In the case of MG, this persistent inflammatory state may exacerbate the autoimmune response by promoting the activation of pro‐inflammatory cytokines and immune cells, thus contributing to the onset or worsening of MG symptoms. Immune dysregulation in autoimmune diseases like MG leads to the destruction of the neuromuscular junction, impairing muscle function (Huda 2023). Additionally, increased adiposity may result in the production of adipokines that further modulate immune cell activity and amplify inflammation. Therefore, obesity can be viewed as a facilitator of immune dysfunction, predisposing individuals with higher BMI to autoimmune diseases such as MG. Similarly, the positive correlation between waist circumference and MG may reflect the role of visceral fat accumulation in the development of autoimmune diseases. Visceral fat, particularly around abdominal organs, is particularly harmful due to its active endocrine function. It secretes pro‐inflammatory adipokines that not only affect metabolic pathways but also modulate immune responses (Kawai et al. 2021). Increased waist circumference often indicates central obesity, which is associated with metabolic syndrome, a cluster of conditions including insulin resistance, hypertension, and dyslipidemia, all of which elevate the risk of autoimmune diseases (Karczewski et al. 2021). Central fat distribution may also impair physical function, exacerbating muscle weakness, a hallmark of MG. Therefore, larger waist circumference may serve as an indicator of metabolic imbalance, which, through its effects on inflammation and immune function, could contribute to the onset or progression of MG. Notably, while the positive correlations between BMI and waist circumference suggest that metabolic disorders may predispose individuals to MG, it is important to recognize that weight management and abdominal obesity may have complex relationships with disease severity. For example, individuals with higher BMI, resulting from metabolic dysfunction and more severe systemic inflammation, may experience more severe MG. On the other hand, weight loss or improved metabolic control could reduce inflammation, potentially leading to more favorable disease outcomes.

The relationships between hypothyroidism, hyperthyroidism, and Myasthenia Gravis (MG) are particularly intriguing, given that both thyroid dysfunction and MG are autoimmune in nature, involving immune system dysregulation. Investigating these connections offers valuable insights into the mechanisms that link metabolic and immune disorders, which may contribute to the onset and progression of MG. Hypothyroidism is marked by reduced thyroid function and insufficient thyroid hormone secretion, often leading to systemic effects such as a slowed metabolism, muscle weakness, and fatigue (Chaker et al. 2017). These symptoms overlap with those commonly seen in MG, such as muscle weakness and fatigue, suggesting a potential interaction between the two conditions. From an immunological perspective, hypothyroidism is typically caused by autoimmune diseases like Hashimoto's thyroiditis, where the immune system targets the thyroid gland (Chaker et al. 2017). This shared autoimmune basis may explain the relationship between hypothyroidism and MG. Additionally, hypothyroidism is associated with a heightened inflammatory state, which could amplify the autoimmune response underlying MG (Ralli et al. 2020). In contrast, hyperthyroidism results from an overproduction of thyroid hormones and is associated with symptoms such as rapid heart rate, weight loss, and muscle weakness. In the context of MG, hyperthyroidism may have a more complex relationship, potentially worsening or even triggering the autoimmune processes that drive the disease. Like hypothyroidism, hyperthyroidism can also cause muscle weakness, a hallmark of MG. However, the exact mechanisms through which hyperthyroidism affects MG are not fully understood, although they may involve changes in immune function. Hyperthyroidism often leads to elevated levels of thyroid hormones, which can impact immune cell function (van der Spek et al. 2017; De Luca et al. 2020). These hormones may stimulate T cells and other immune mediators, potentially intensifying the autoimmune response in MG. Furthermore, hyperthyroidism may induce a hypermetabolic state, which could alter immune system activity and exacerbate the progression of autoimmune diseases, including MG (Wiersinga et al. 2023).

The significant negative correlation between fresh fruit intake and Myasthenia Gravis (MG) suggests that dietary factors, particularly those with anti‐inflammatory properties, may play a key role in modulating the immune system and reducing the risk of autoimmune diseases like MG. It is well‐established that fresh fruits, especially those rich in vitamins, antioxidants, and fiber, have beneficial effects on immune function and inflammation. This relationship warrants further investigation to better understand how fresh fruit consumption influences the pathophysiology of MG and contributes to disease management. Fresh fruits are abundant in antioxidants such as vitamin C, vitamin E, and polyphenols, which help neutralize reactive oxygen species (ROS) and alleviate oxidative stress. Oxidative stress, which contributes to inflammation and tissue damage, is implicated in the pathogenesis of autoimmune diseases, including MG (Wang et al. 2012; Wójcik et al. 2021). Many fruits also contain bioactive compounds with anti‐inflammatory properties, such as flavonoids and carotenoids. These compounds have the ability to inhibit the production of inflammatory mediators, including TNF‐α, IL‐6, and CRP, which are elevated in MG (Zhu et al. 2018; Tüzün et al. 2011). In addition, fresh fruits provide a valuable source of dietary fiber that supports the health of the gut microbiome. A balanced gut microbiome plays a crucial role in immune regulation by maintaining the integrity of the intestinal barrier and preventing systemic inflammation (Amoroso et al. 2020; Belkaid and Hand 2014). In addition, fruits provide essential vitamins and minerals such as vitamin D, magnesium, and zinc, which are crucial for immune function. For example, vitamin D deficiency has been associated with an increased susceptibility to autoimmune diseases (Murdaca et al. 2019; Charoenngam and Holick 2020). The negative correlation between fresh fruit intake and MG underscores the potential role of diet in preventing autoimmune disorders. Given their antioxidant, anti‐inflammatory, and immune‐regulatory properties, fresh fruits may offer protective effects against MG. As such, incorporating fresh fruits into the diet could serve as a simple yet effective strategy for reducing the risk of MG and enhancing overall health.

Although this MR analysis provides valuable insights into the causal relationships between dietary and metabolic factors and MG, several limitations should be acknowledged. First, the study relies on genetic variants as IVs, which may not fully capture the complexity of dietary and metabolic exposures, potentially introducing minor instrument bias. Second, the analysis is based on summary data from European populations, which limits the generalizability of the findings to other racial and ethnic groups. Third, while MR reduces confounding and reverse causality, it cannot entirely exclude residual pleiotropy, where genetic variants affect the outcome through pathways other than the exposure. Despite conducting sensitivity analyses, this issue remains a potential concern. Fourth, the study focuses on a limited set of dietary and metabolic factors, possibly overlooking other relevant exposures. To address these limitations, future research should seek to diversify the population by including different racial and ethnic groups, thereby enhancing the generalizability of the results. Furthermore, the potential genetic and environmental differences in the relationship between diet, metabolism, and MG warrant further exploration. Additionally, incorporating more detailed dietary data, such as specific nutrients, food preparation methods, and overall dietary patterns, could provide deeper insights into how these factors influence the risk of MG. Randomized controlled trials (RCTs) that test dietary and metabolic interventions, such as increasing fresh fruit intake or implementing weight management programs, could offer practical evidence for the prevention and management of MG. Finally, using advanced MR techniques along with larger GWAS datasets will be essential to address pleiotropy issues and improve causal inferences. Exploring gene–environment interactions will further contribute to a more comprehensive understanding of MG's etiology and its modifiable risk factors, ultimately aiding in the development of more effective prevention and treatment strategies.

## 5 Conclusion

In conclusion, this study underscores the pivotal role of metabolic and dietary factors in the pathogenesis and progression of Myasthenia Gravis (MG). The findings suggest that these factors influence the development of the disease, but further research is necessary to fully unravel the underlying mechanisms. Incorporating clinical variables, more detailed dietary data, and longitudinal studies that track changes over time will be crucial for establishing a clearer temporal relationship between lifestyle factors and MG. Additionally, a deeper understanding of gene–environment interactions in MG is vital, as it will provide invaluable insights, ultimately enabling the development of more personalized prevention and treatment strategies.

## Author Contributions

**Guoliang You:** conceptualization (equal), data curation (equal), formal analysis (equal), funding acquisition (equal), investigation (equal), methodology (equal), project administration (equal), resources (equal), software (equal), supervision (equal), validation (equal), visualization (equal), writing – original draft (equal), writing – review and editing (equal). **Meng Li:** conceptualization (equal), data curation (equal), formal analysis (equal), investigation (equal), methodology (equal), project administration (equal), resources (equal), software (equal), supervision (equal), validation (equal), visualization (equal), writing – original draft (equal), writing – review and editing (equal). **Minheng Zhang:** data curation (equal), funding acquisition (equal), methodology (equal), resources (equal), supervision (equal), visualization (equal), writing – review and editing (equal). **Hongwei Liu:** conceptualization (equal), data curation (equal), formal analysis (equal), investigation (equal), methodology (equal), project administration (equal), resources (equal), software (equal), supervision (equal), validation (equal), visualization (equal), writing – original draft (equal), writing – review and editing (equal). **Xuan Chen:** conceptualization (equal), formal analysis (equal), investigation (equal), project administration (equal), software (equal), validation (equal), writing – original draft (equal). **Haixia Fan:** conceptualization (equal), data curation (equal), formal analysis (equal), funding acquisition (equal), investigation (equal), methodology (equal), project administration (equal), resources (equal), software (equal), supervision (equal), validation (equal), visualization (equal), writing – original draft (equal), writing – review and editing (equal).

## Conflicts of Interest

The authors declare no conflicts of interest.

## Supporting information


**Supplementary Figure 1.** Leave‐one‐out analysis illustrating the causal effects of 26 kinds of features on myasthenia gravis [Page 2–27].
**Supplementary Figure 1A**. MR leave‐one‐out sensitivity analysis for beef intake on myasthenia gravis.
**Supplementary Figure 1B**. MR leave‐one‐out sensitivity analysis for pork intake on myasthenia gravis.
**Supplementary Figure 1C**. MR leave‐one‐out sensitivity analysis for poultry intake on myasthenia gravis.
**Supplementary Figure 1D**. MR leave‐one‐out sensitivity analysis for oily fish intake on myasthenia gravis.
**Supplementary Figure 1E**. MR leave‐one‐out sensitivity analysis for non‐oily fish intake on myasthenia gravis.
**Supplementary Figure 1F**. MR leave‐one‐out sensitivity analysis for processed meat intake on myasthenia gravis.
**Supplementary Figure 1G**. MR leave‐one‐out sensitivity analysis for tea intake on myasthenia gravis.
**Supplementary Figure 1H**. MR leave‐one‐out sensitivity analysis for water intake on myasthenia gravis.
**Supplementary Figure 1I**. MR leave‐one‐out sensitivity analysis for alcohol intake frequency on myasthenia gravis.
**Supplementary Figure 1J**. MR leave‐one‐out sensitivity analysis for bread intake on myasthenia gravis.
**Supplementary Figure 1K**. MR leave‐one‐out sensitivity analysis for cheese intake on myasthenia gravis.
**Supplementary Figure 1L**. MR leave‐one‐out sensitivity analysis for cereal intake on myasthenia gravis.
**Supplementary Figure 1M**. MR leave‐one‐out sensitivity analysis for dried fruit intake on myasthenia gravis.
**Supplementary Figure 1N**. MR leave‐one‐out sensitivity analysis for fresh fruit intake on myasthenia gravis.
**Supplementary Figure 1O**. MR leave‐one‐out sensitivity analysis for cooked vegetable intake on myasthenia gravis.
**Supplementary Figure 1P**. MR leave‐one‐out sensitivity analysis for salad/raw vegetable intake on myasthenia gravis.
**Supplementary Figure 1Q**. MR leave‐one‐out sensitivity analysis for body mass index on myasthenia gravis.
**Supplementary Figure 1R**. MR leave‐one‐out sensitivity analysis for waist circumference on myasthenia gravis.
**Supplementary Figure 1S**. MR leave‐one‐out sensitivity analysis for hypothyroidism on myasthenia gravis.
**Supplementary Figure 1T**. MR leave‐one‐out sensitivity analysis for hyperthyroidism on myasthenia gravis.
**Supplementary Figure 1U**. MR leave‐one‐out sensitivity analysis for HDL cholesterol on myasthenia gravis.
**Supplementary Figure 1V**. MR leave‐one‐out sensitivity analysis for LDL cholesterol on myasthenia gravis.
**Supplementary Figure 1W**. MR leave‐one‐out sensitivity analysis for triglycerides on myasthenia gravis.
**Supplementary Figure 1X**. MR leave‐one‐out sensitivity analysis for glycated hemoglobin on myasthenia gravis.
**Supplementary Figure 1Y**. MR leave‐one‐out sensitivity analysis for fasting insulin on myasthenia gravis.
**Supplementary Figure 1Z**. MR leave‐one‐out sensitivity analysis for fasting glucose on myasthenia gravis.

**Supplementary Table 1.** Single nucleotide polymorphisms (SNPs) were used as instrumental variables (IVs) from dietary habits and metabolic factor [Page 2–96].
**Supplementary Table 2**. Specific information for univariable MR analysis of dietary habits and metabolic factor as exposure and migraine as outcome [Page 97–102].

## Data Availability

The datasets used and/or analyzed in this study are available from the corresponding author upon reasonable request.
