# Supplementary material for: Genetic Insights Into Dietary Factors, Metabolic Traits and Myasthenia Gravis Risk: A Large‐Scale Two‐Sample Mendelian Randomization Study in European Populations
Source: Food Sci Nutr. 2025 May 26;13(6):e70236. doi: 10.1002/fsn3.70236 (PMC12121443; doi:10.1002/fsn3.70236)
Supplement: Supplementary file 1 — Supplementary Figure 1. Leave‐one‐out analysis illustrating the causal effects of 26 kinds of features on myasthenia gravis [Page 2–27]. Supplementary Figure 1A. MR leave‐one‐out sensitivity analysis for beef intake on myasthenia gravis. Supplementary Figure 1B. MR leave‐one‐out sensitivity analysis for pork intake on myasthenia gravis. Supplementary Figure 1C. MR leave‐one‐out sensitivity analysis for poultry intake on myasthenia gravis. Supplementary Figure 1D. MR leave‐one‐out sensitivity analysis for oily fish intake on myasthenia gravis. Supplementary Figure 1E. MR leave‐one‐out sensitivity analysis for non‐oily fish intake on myasthenia gravis. Supplementary Figure 1F. MR leave‐one‐out sensitivity analysis for processed meat intake on myasthenia gravis. Supplementary Figure 1G. MR leave‐one‐out sensitivity analysis for tea intake on myasthenia gravis. Supplementary Figure 1H. MR leave‐one‐out sensitivity analysis for water intake on myasthenia gravis. Supplementary Figure 1I. MR leave‐one‐out sensitivity analysis for alcohol intake frequency on myasthenia gravis. Supplementary Figure 1J. MR leave‐one‐out sensitivity analysis for bread intake on myasthenia gravis. Supplementary Figure 1K. MR leave‐one‐out sensitivity analysis for cheese intake on myasthenia gravis. Supplementary Figure 1L. MR leave‐one‐out sensitivity analysis for cereal intake on myasthenia gravis. Supplementary Figure 1M. MR leave‐one‐out sensitivity analysis for dried fruit intake on myasthenia gravis. Supplementary Figure 1N. MR leave‐one‐out sensitivity analysis for fresh fruit intake on myasthenia gravis. Supplementary Figure 1O. MR leave‐one‐out sensitivity analysis for cooked vegetable intake on myasthenia gravis. Supplementary Figure 1P. MR leave‐one‐out sensitivity analysis for salad/raw vegetable intake on myasthenia gravis. Supplementary Figure 1Q. MR leave‐one‐out sensitivity analysis for body mass index on myasthenia gravis. Supplementary Figure 1R. MR leave‐one‐out sensitivity an [file FSN3-13-e70236-s001.docx]

**Supplementary Figure 1** Leave-one-out analysis illustrating the causal effects of 26 kinds of features on myasthenia gravis [Page 2-27]


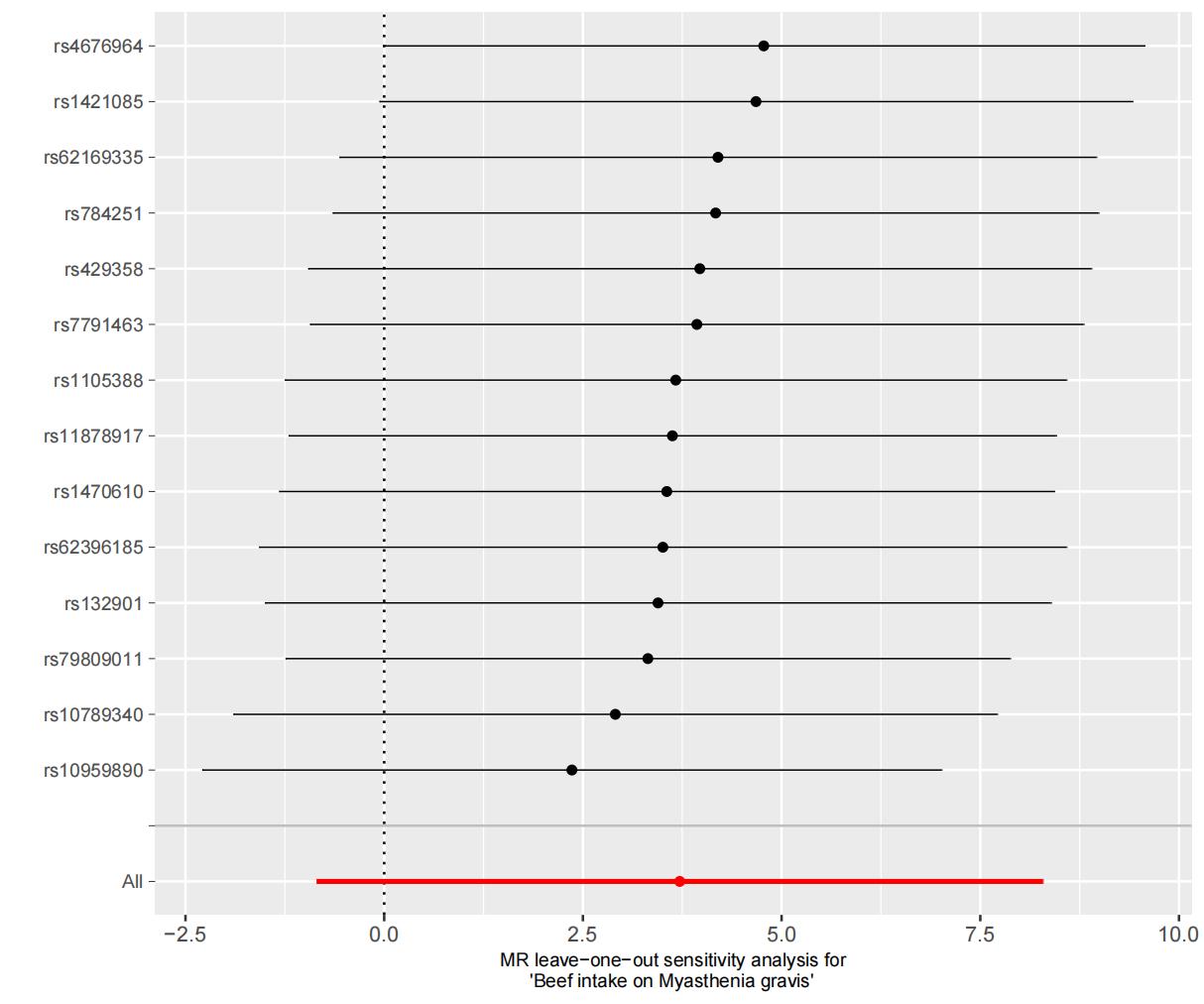


**Supplementary Figure 1A** MR leave-one-out sensitivity analysis for beef intake on myasthenia gravis

**Abbreviation:** MR**,** Mendelian randomization


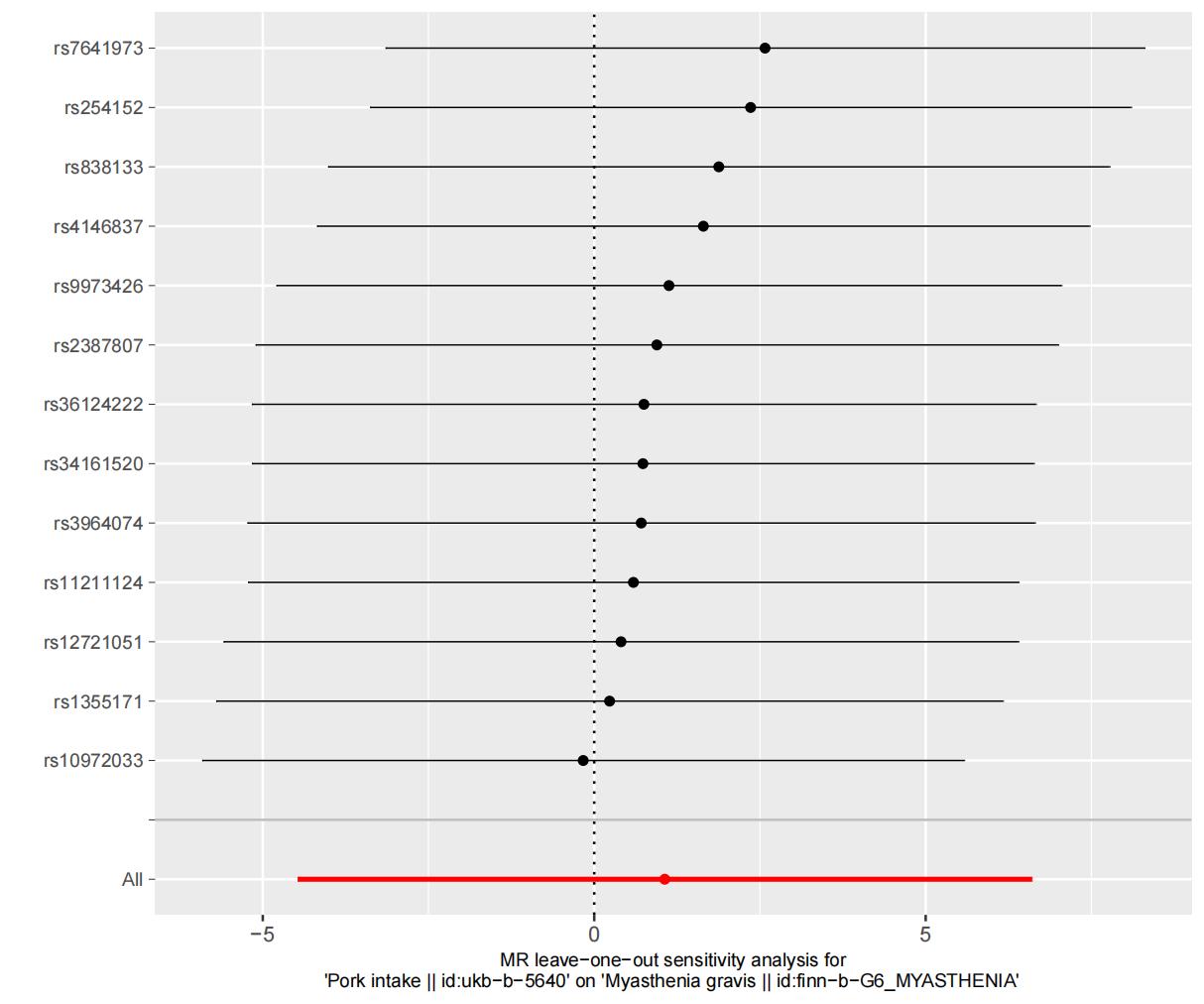


**Supplementary Figure 1B** MR leave-one-out sensitivity analysis for pork intake on myasthenia gravis

**Abbreviation:** MR**,** Mendelian randomization


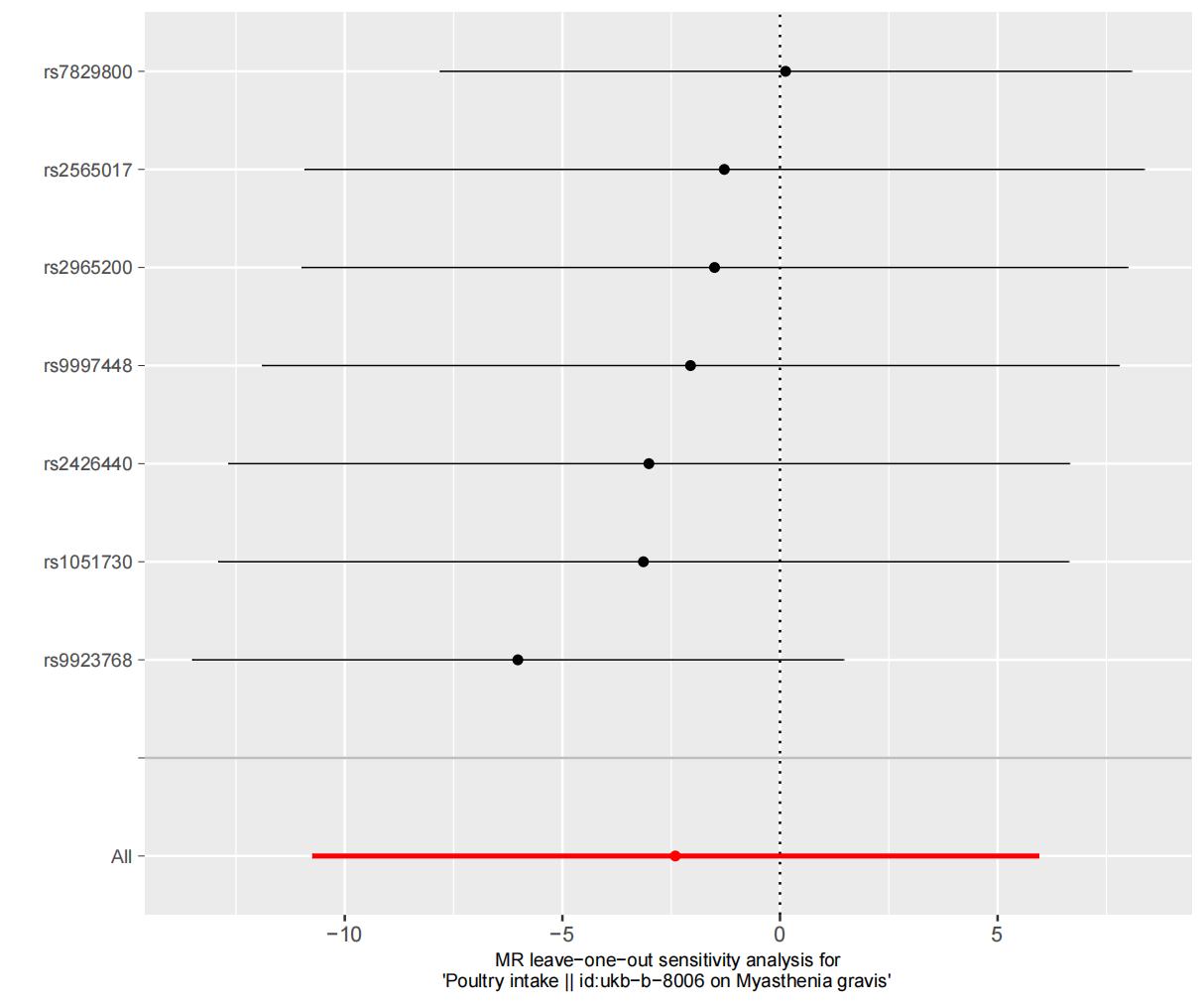


**Supplementary Figure 1C** MR leave-one-out sensitivity analysis for poultry intake intake on myasthenia gravis

**Abbreviation:** MR**,** Mendelian randomization


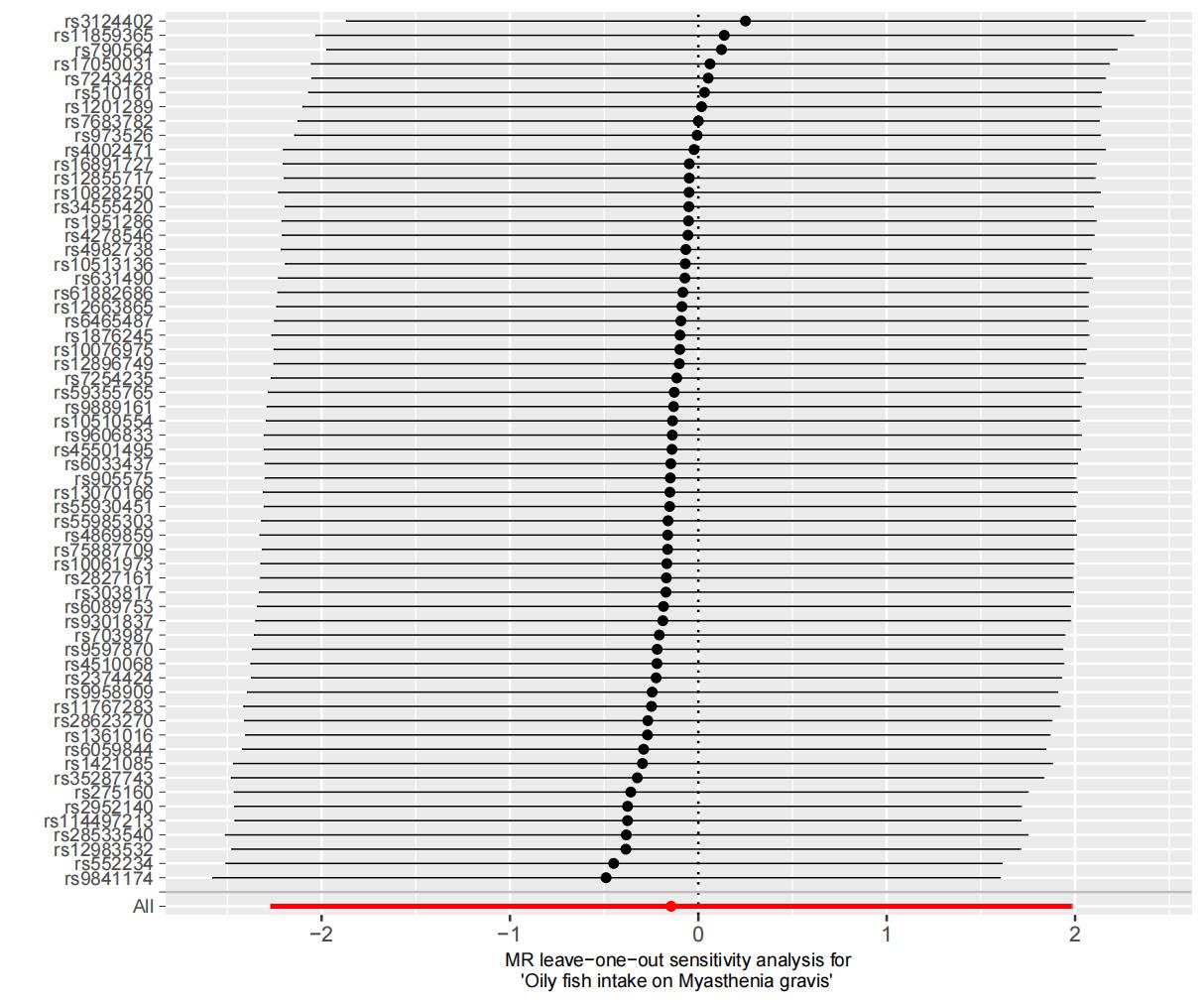


**Supplementary Figure 1D** MR leave-one-out sensitivity analysis for oily fish intake on myasthenia gravis

**Abbreviation:** MR**,** Mendelian randomization


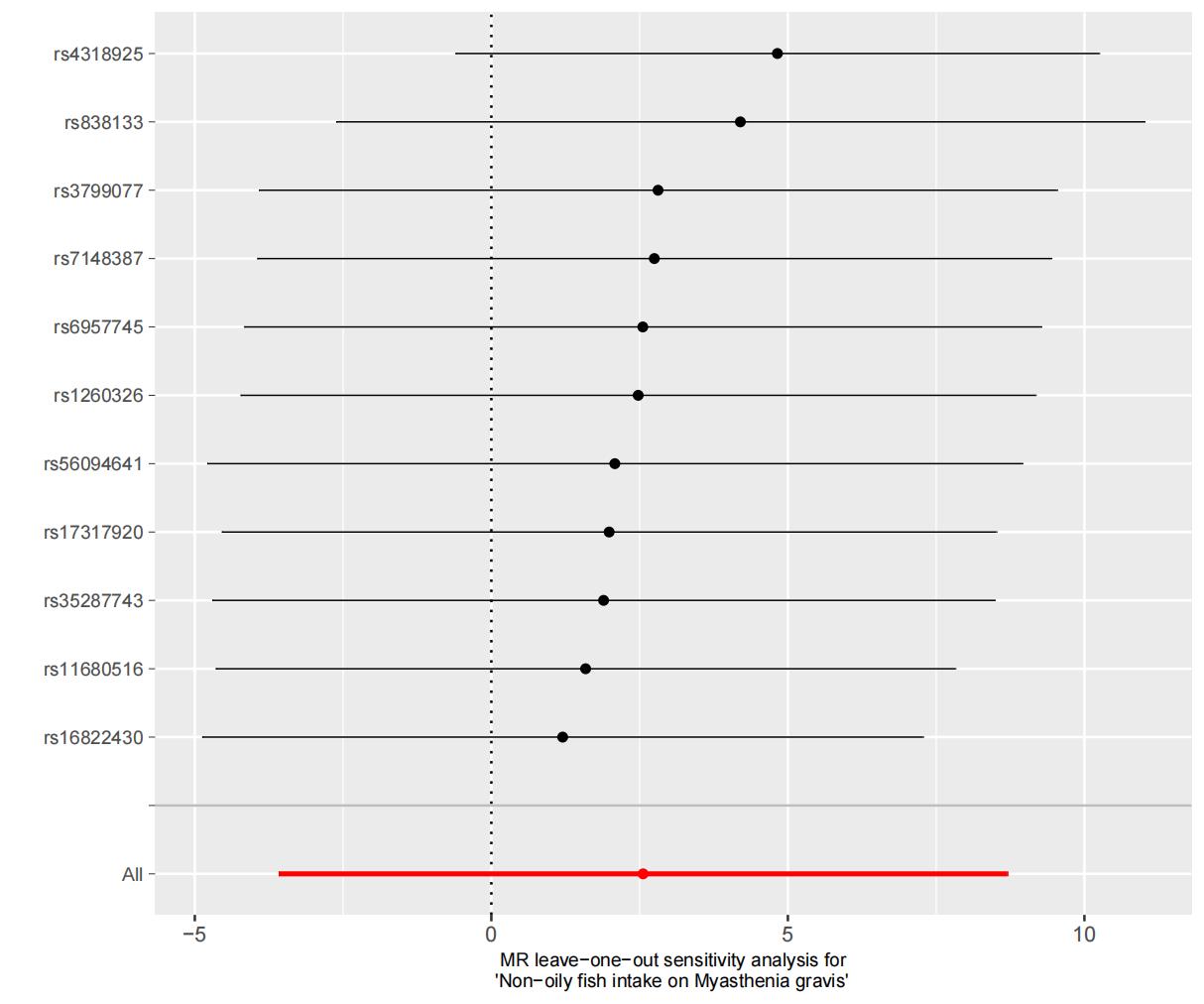


**Supplementary Figure 1E** MR leave-one-out sensitivity analysis for non-oily fish intake on myasthenia gravis

**Abbreviation:** MR**,** Mendelian randomization


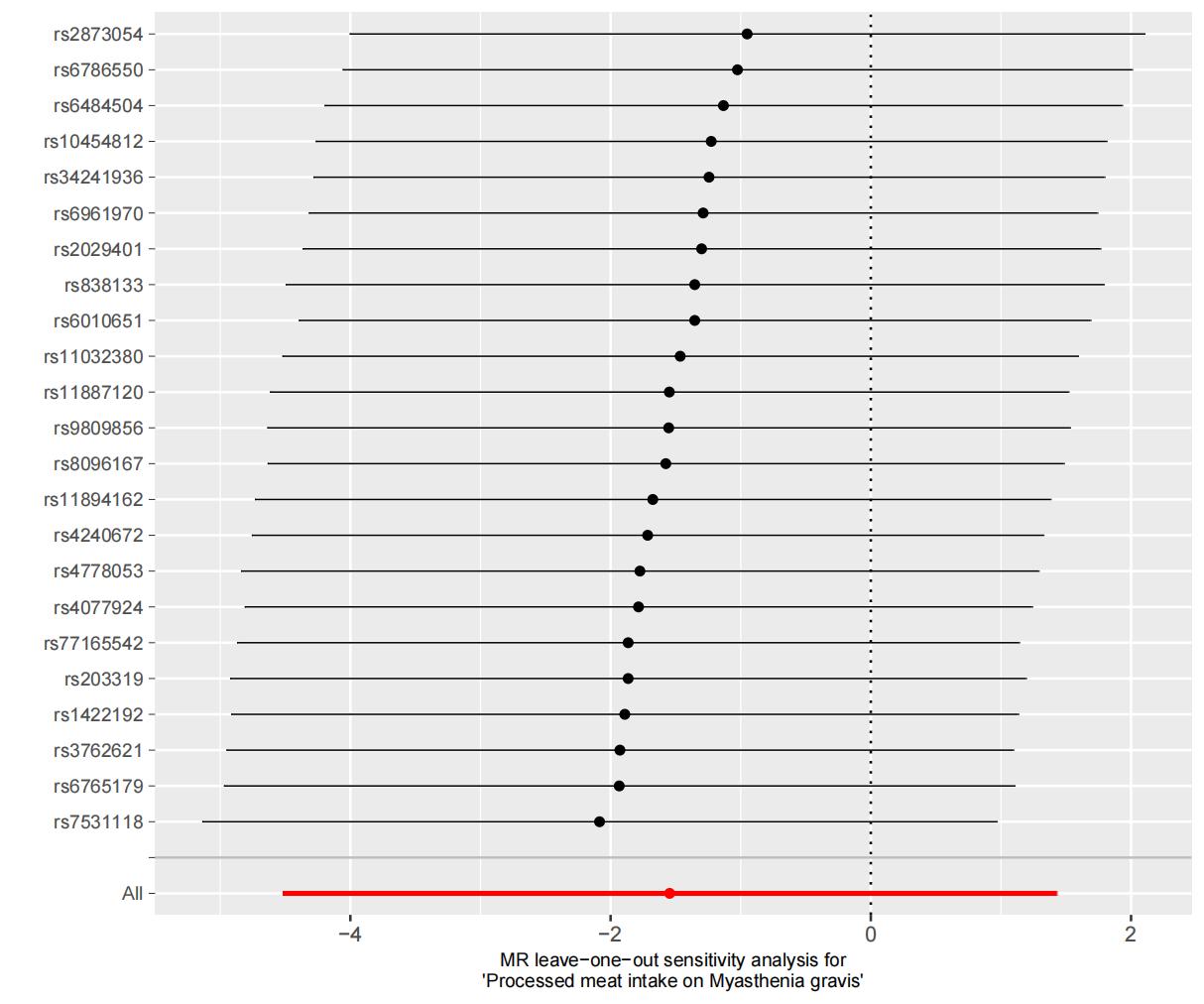


**Supplementary Figure 1F** MR leave-one-out sensitivity analysis for processed meat intake on myasthenia gravis

**Abbreviation:** MR**,** Mendelian randomization


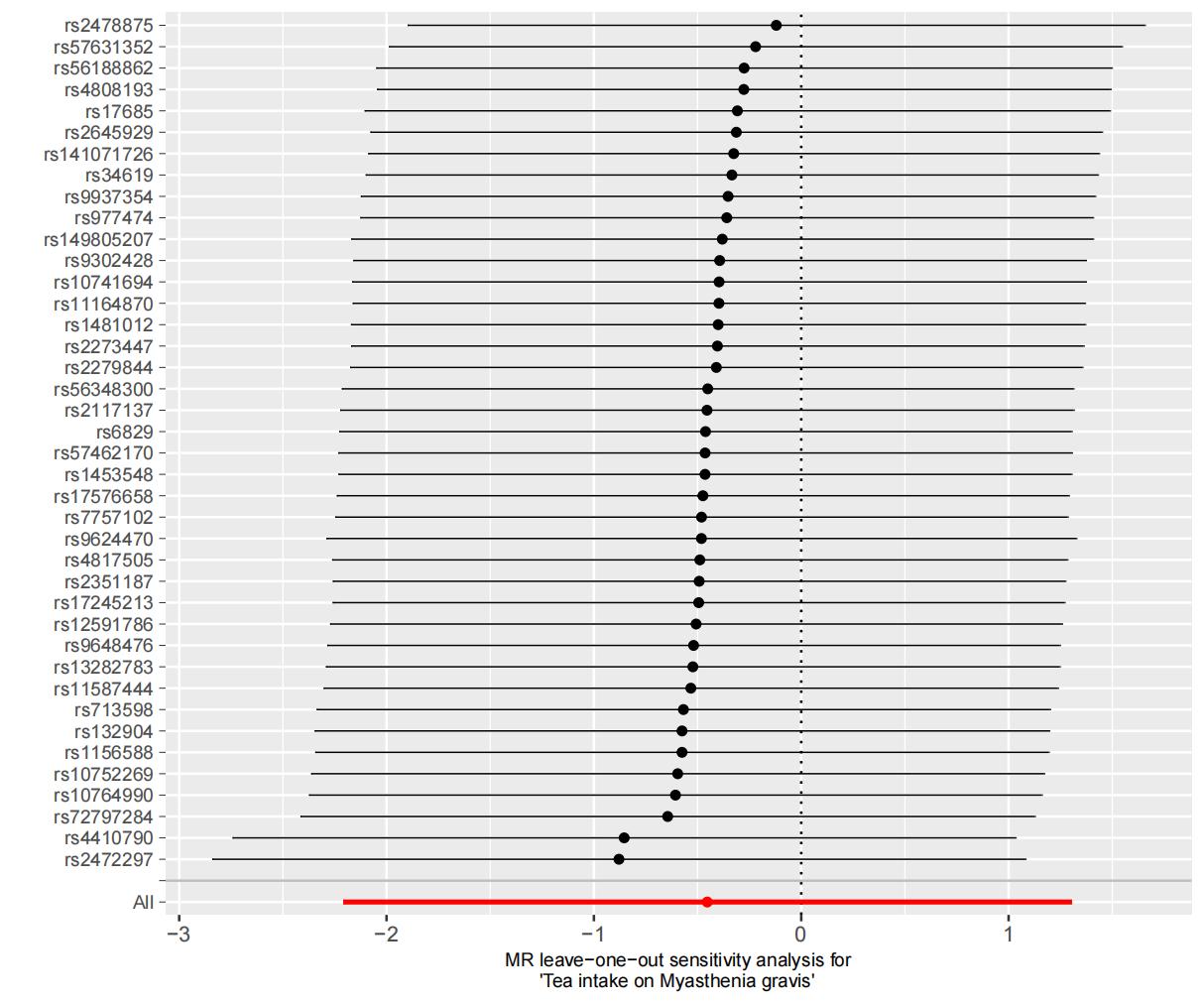


**Supplementary Figure 1G** MR leave-one-out sensitivity analysis for tea intake on myasthenia gravis

**Abbreviation:** MR**,** Mendelian randomization


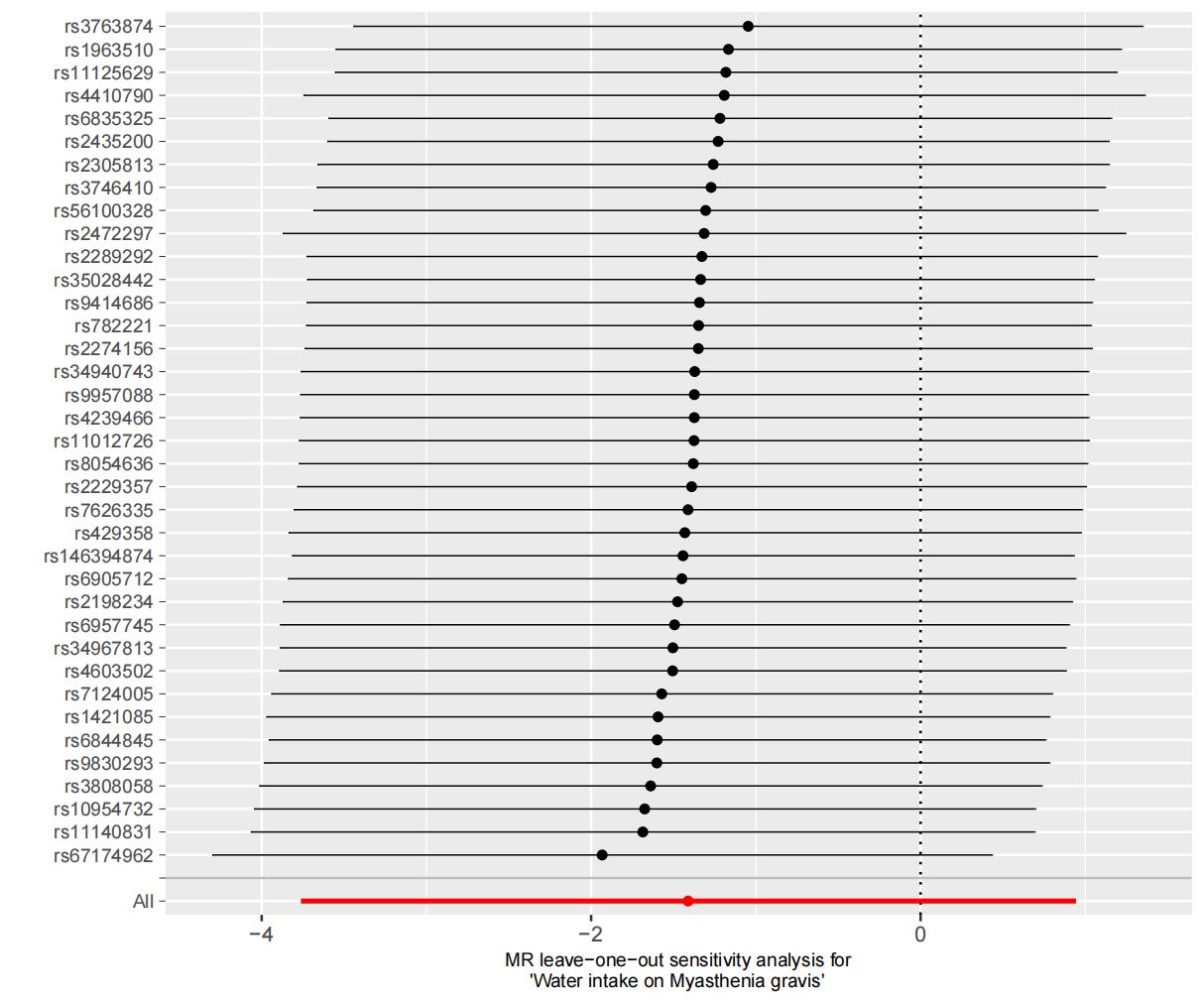


**Supplementary Figure 1H** MR leave-one-out sensitivity analysis for water intake on myasthenia gravis

**Abbreviation:** MR**,** Mendelian randomization


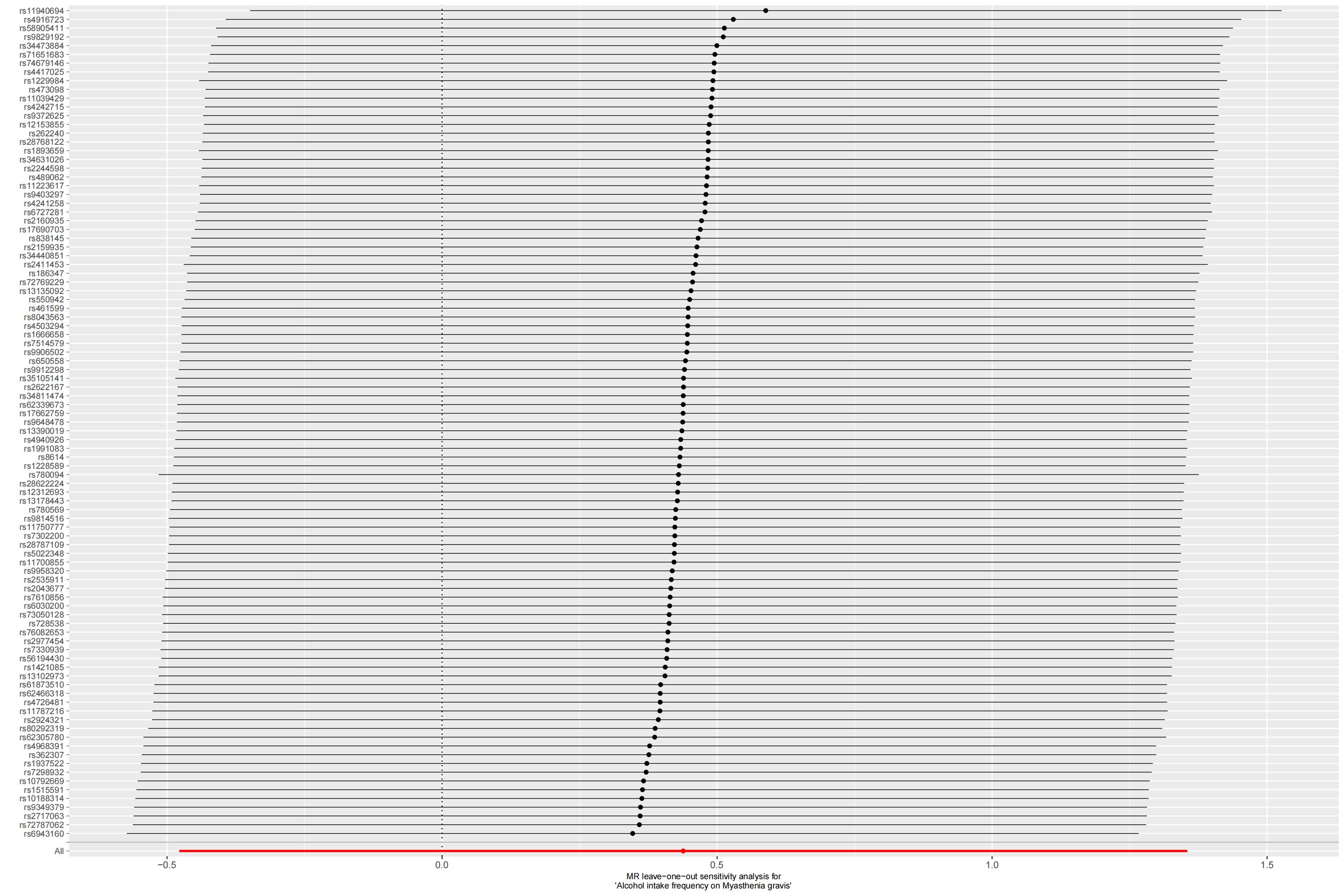


**Supplementary Figure 1I** MR leave-one-out sensitivity analysis for alcohol intake frequency on myasthenia gravis

**Abbreviation:** MR**,** Mendelian randomization


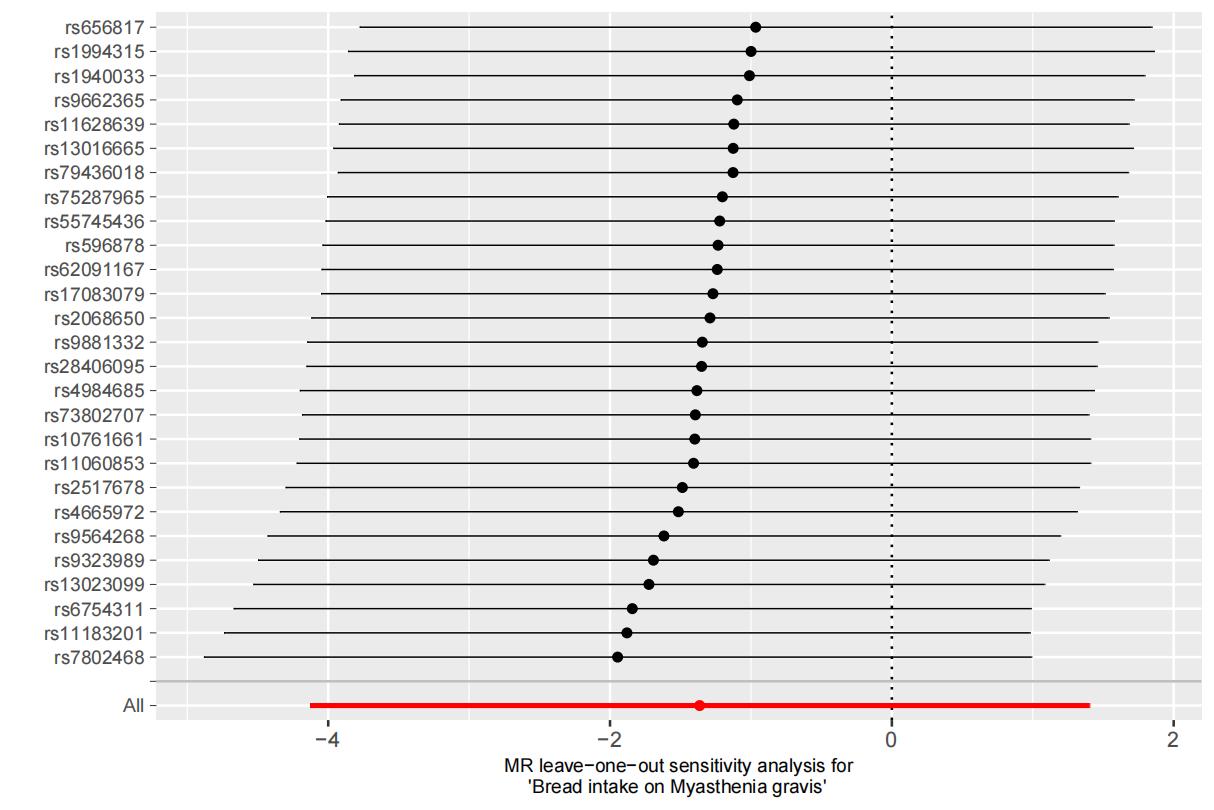


**Supplementary Figure 1J** MR leave-one-out sensitivity analysis for bread intake on myasthenia gravis

**Abbreviation:** MR**,** Mendelian randomization


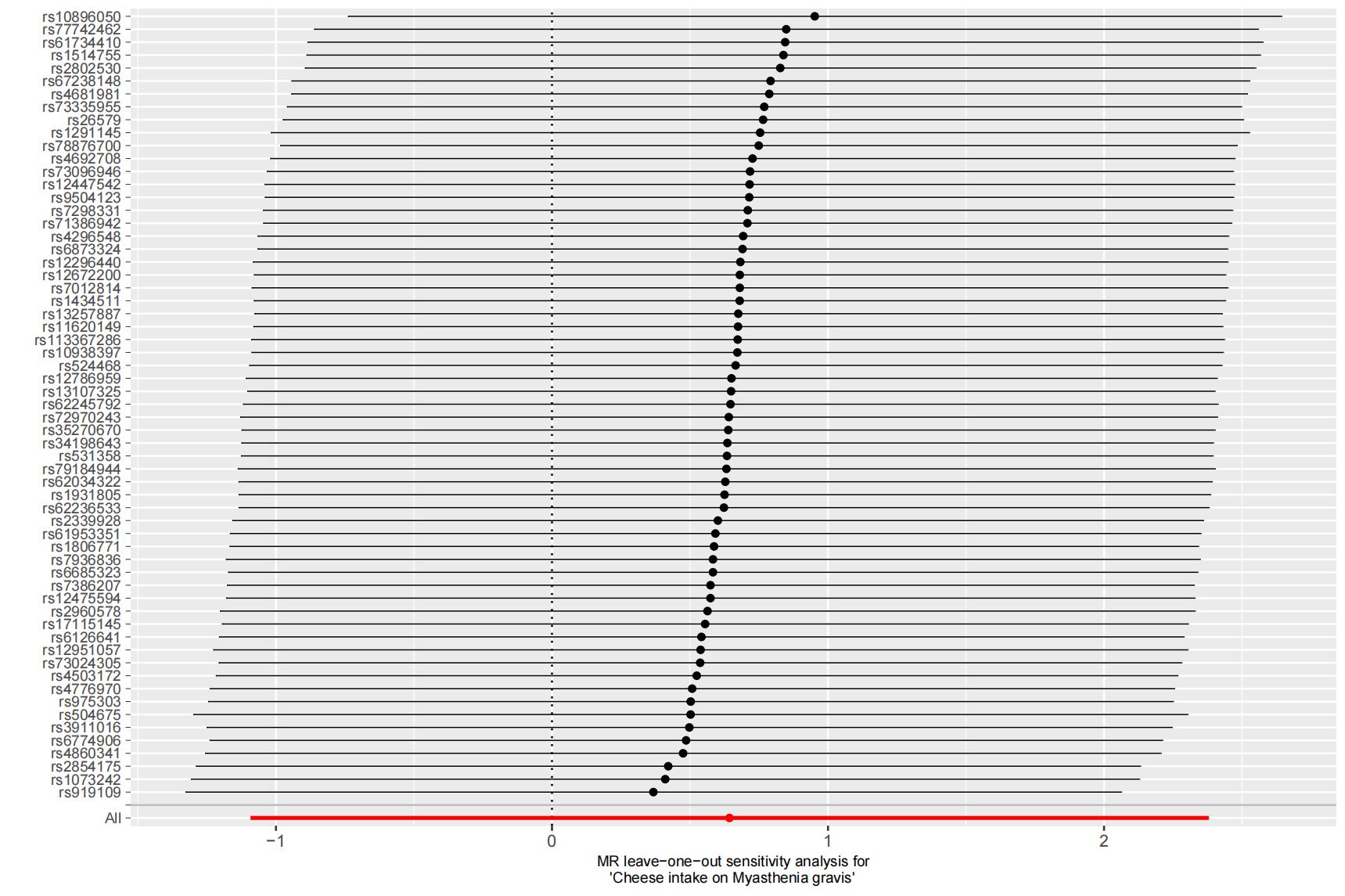


**Supplementary Figure 1K** MR leave-one-out sensitivity analysis for cheese intake on myasthenia gravis

**Abbreviation:** MR**,** Mendelian randomization


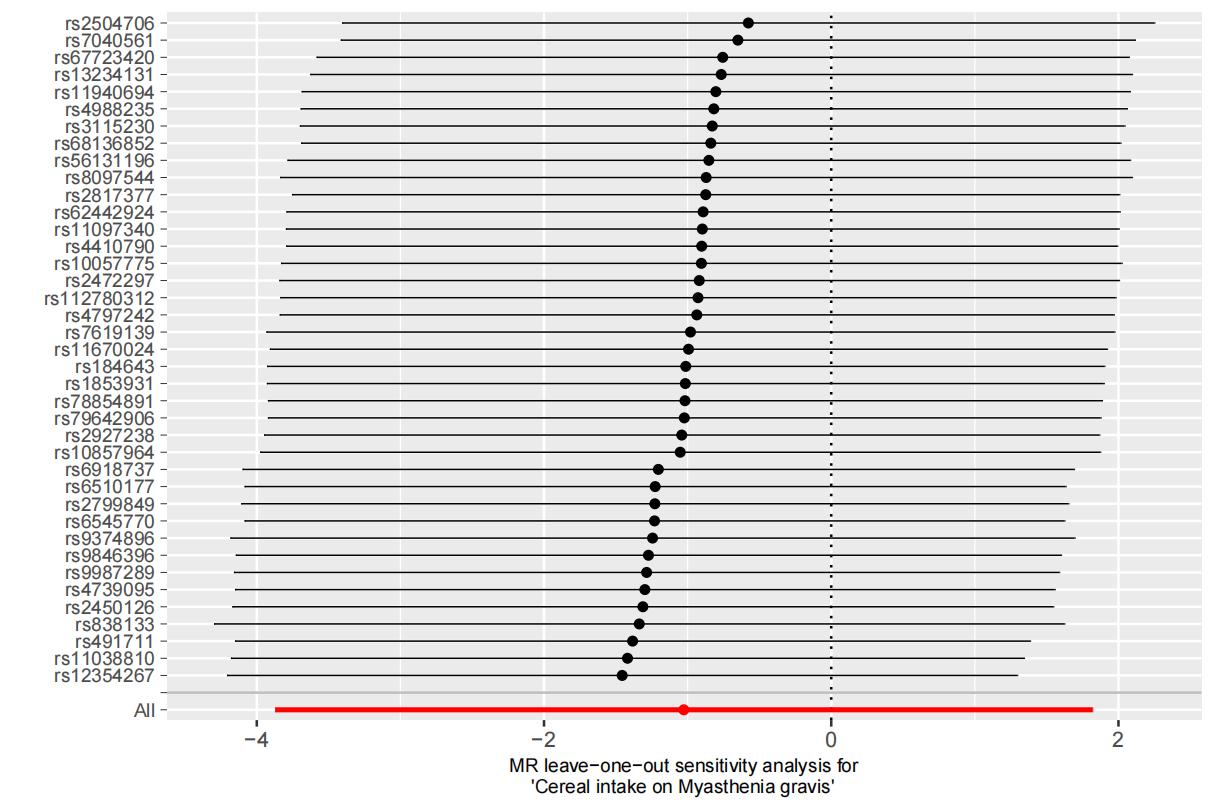


**Supplementary Figure 1L** MR leave-one-out sensitivity analysis for cereal intake on myasthenia gravis

**Abbreviation:** MR**,** Mendelian randomization


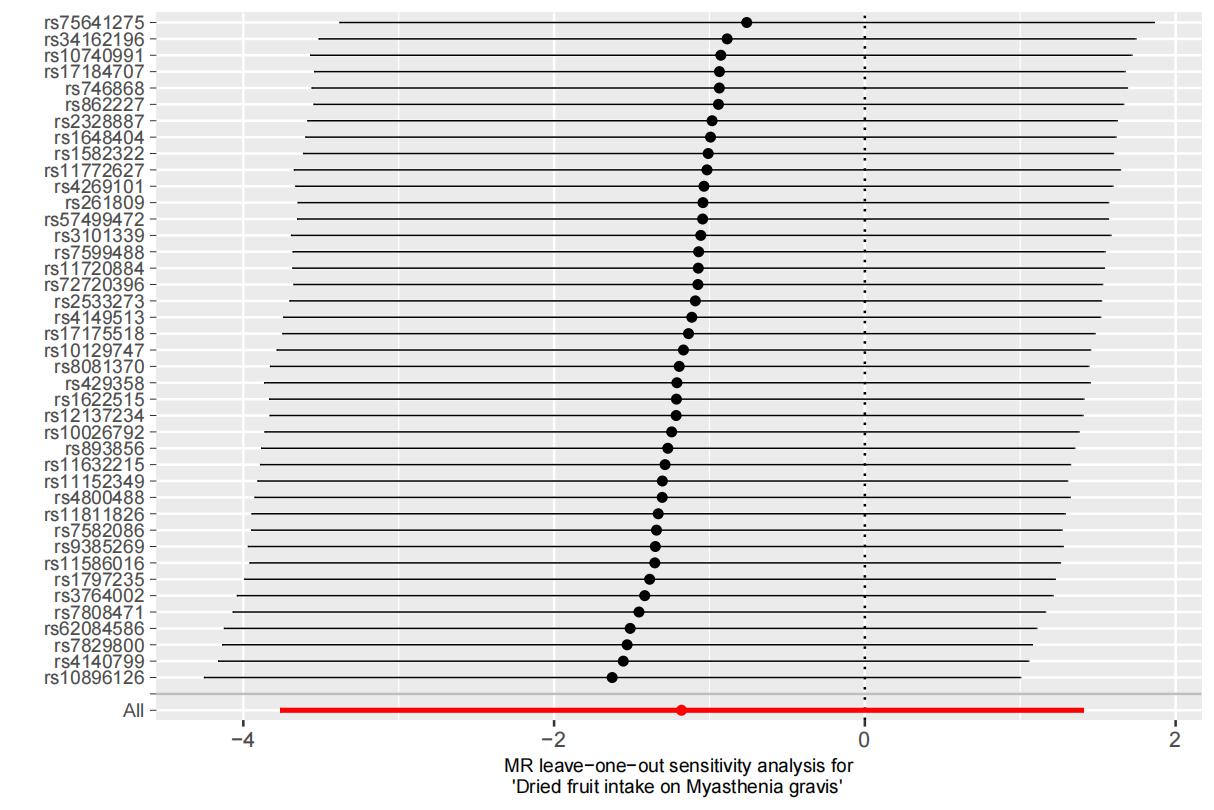


**Supplementary Figure 1M** MR leave-one-out sensitivity analysis for dried fruit intake on myasthenia gravis

**Abbreviation:** MR**,** Mendelian randomization


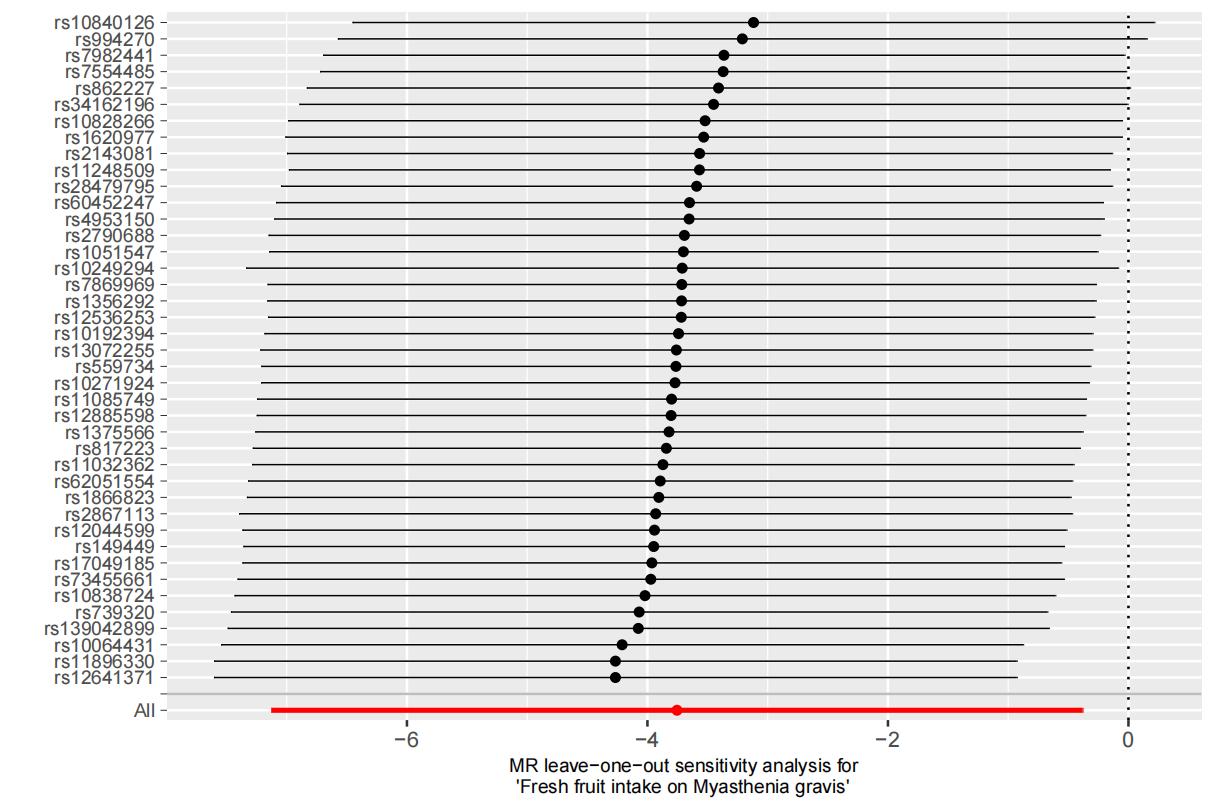


**Supplementary Figure 1N** MR leave-one-out sensitivity analysis for fresh fruit intake on myasthenia gravis

**Abbreviation:** MR**,** Mendelian randomization


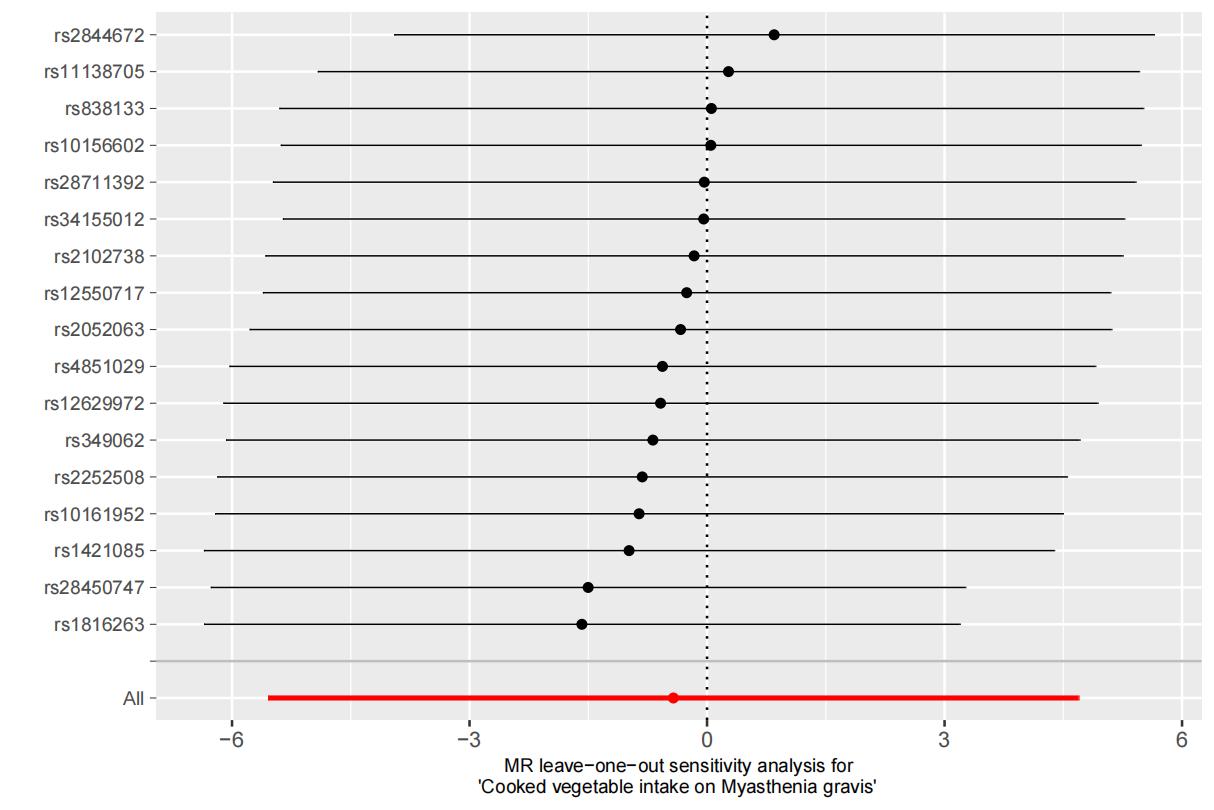


**Supplementary Figure 1O** MR leave-one-out sensitivity analysis for cooked vegetable intake on myasthenia gravis

**Abbreviation:** MR**,** Mendelian randomization


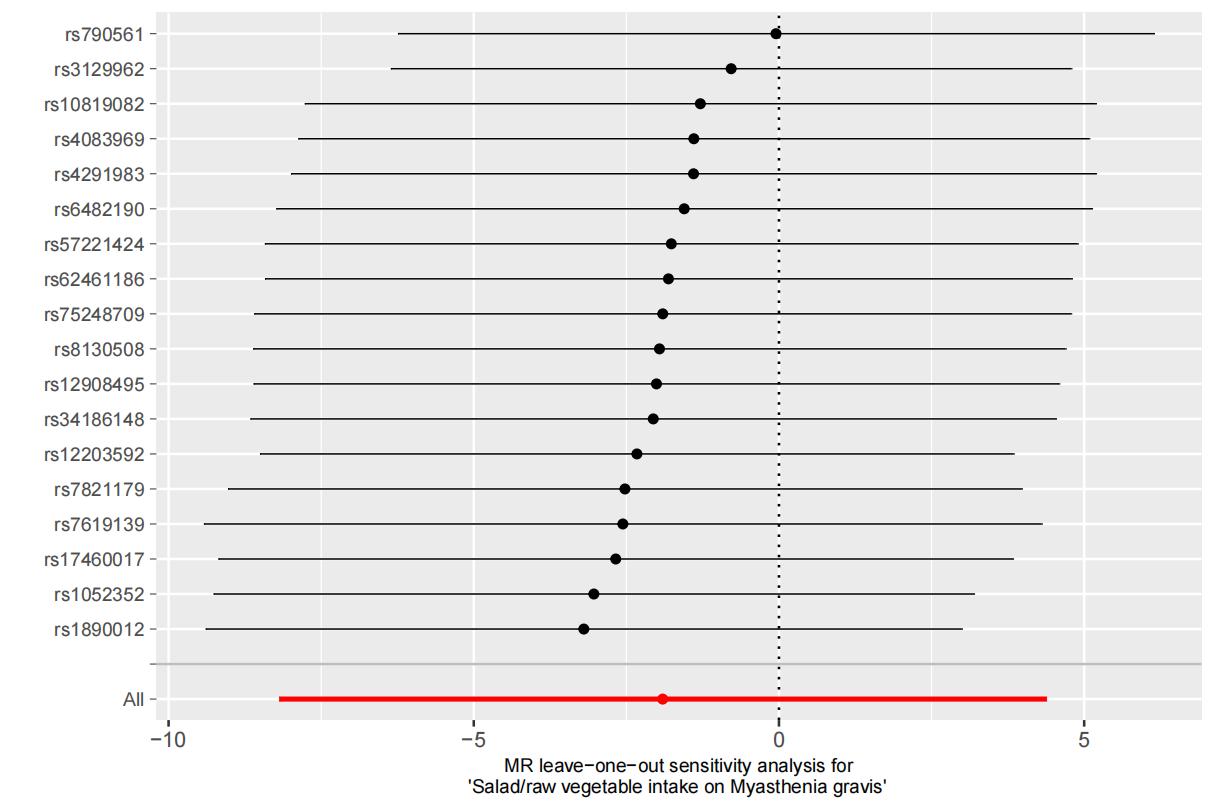


**Supplementary Figure 1P** MR leave-one-out sensitivity analysis for salad/raw vegetable intake on myasthenia gravis

**Abbreviation:** MR**,** Mendelian randomization


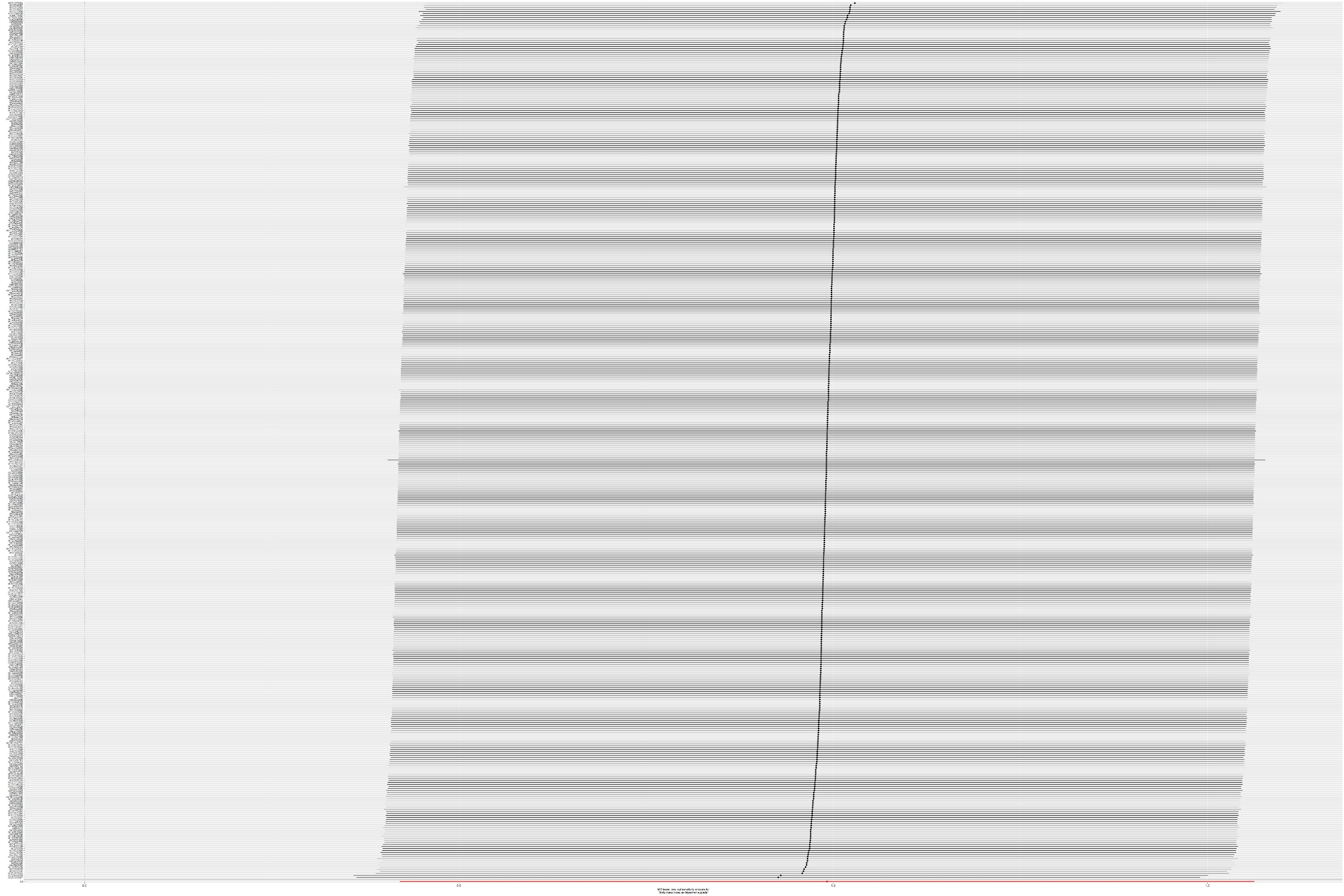


**Supplementary Figure 1Q** MR leave-one-out sensitivity analysis for body mass index on myasthenia gravis

**Abbreviation:** MR**,** Mendelian randomization


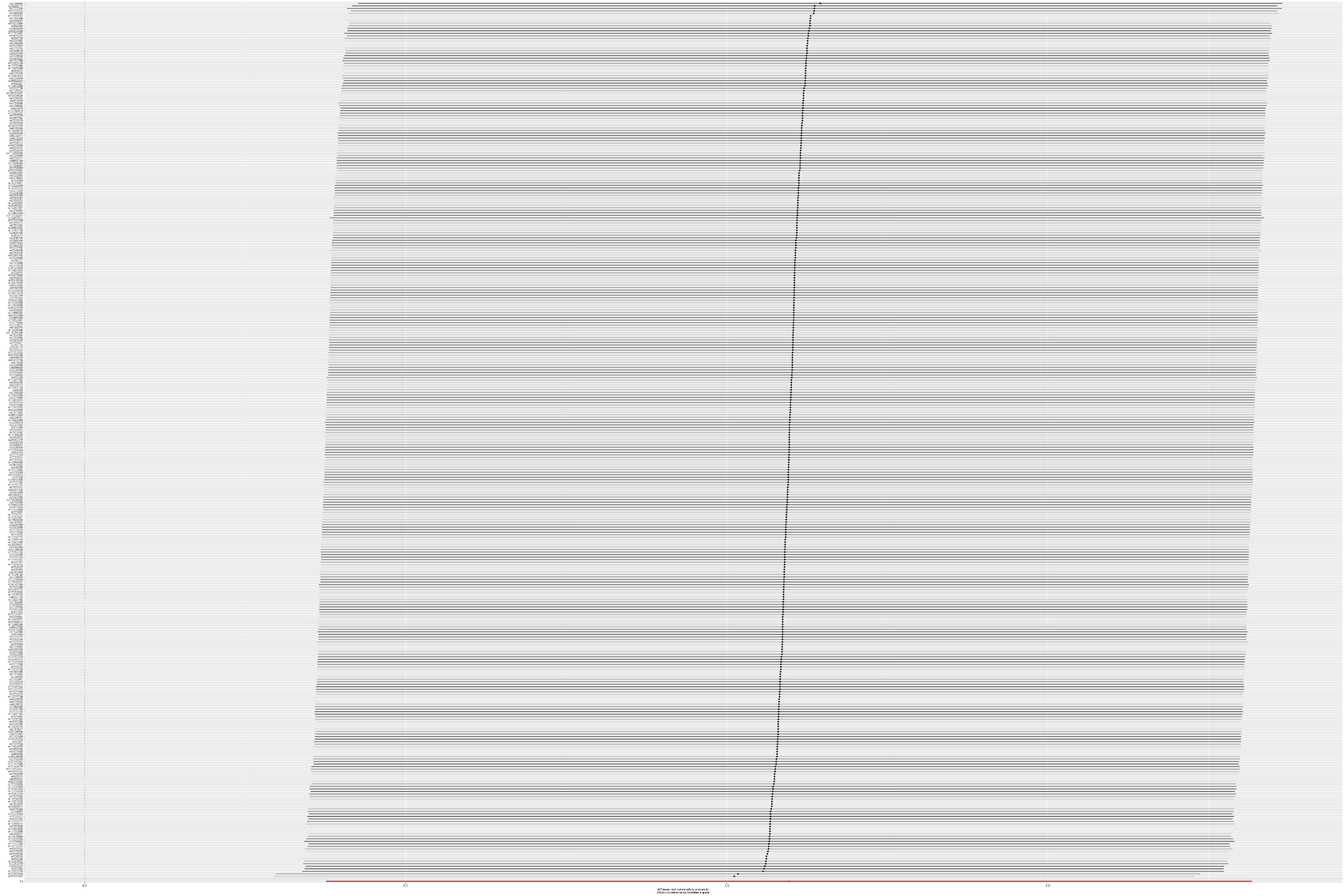


**Supplementary Figure 1R** MR leave-one-out sensitivity analysis for waist circumference on myasthenia gravis

**Abbreviation:** MR**,** Mendelian randomization


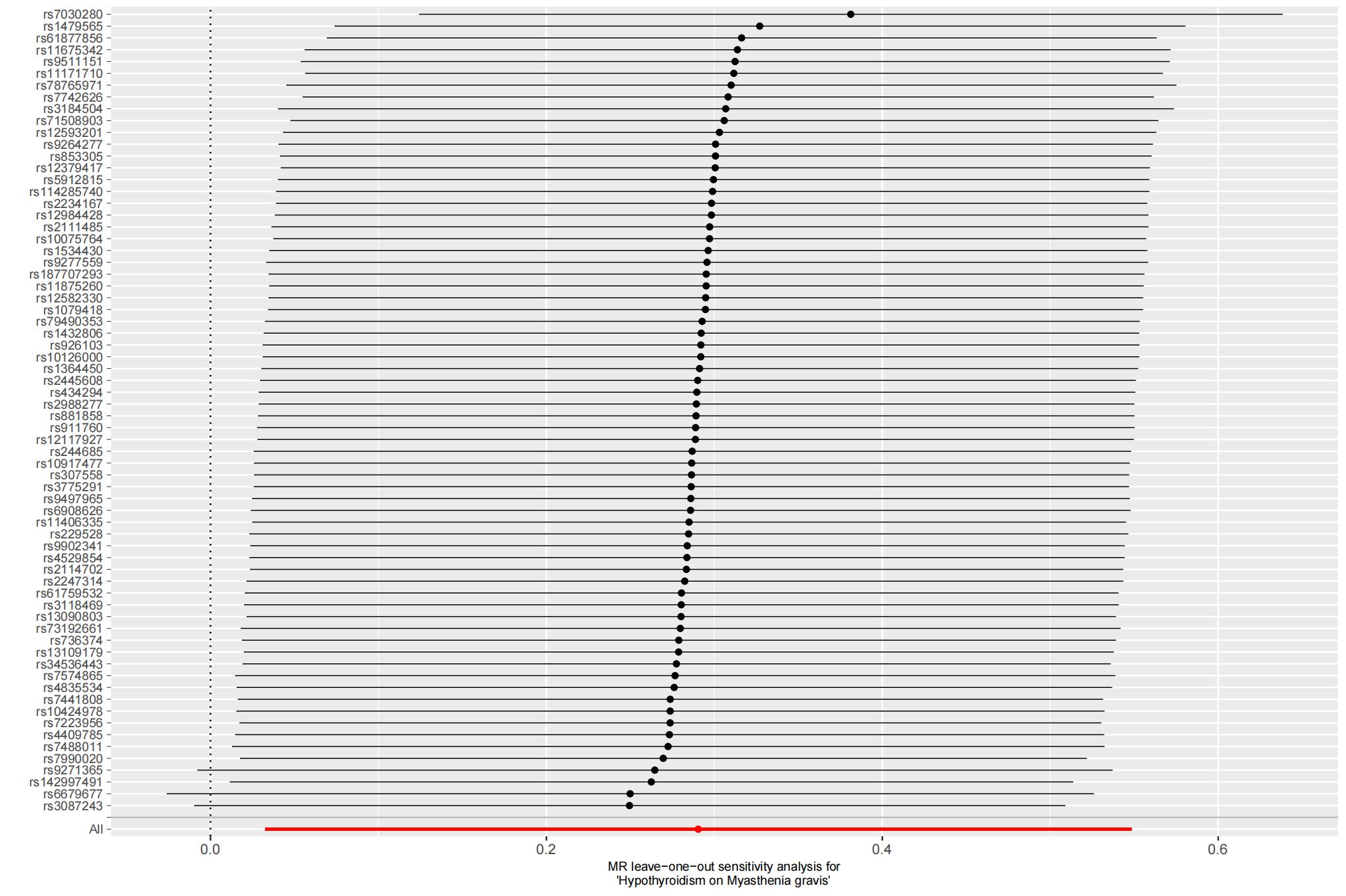


**Supplementary Figure 1S** MR leave-one-out sensitivity analysis for hypothyroidism on myasthenia gravis

**Abbreviation:** MR**,** Mendelian randomization


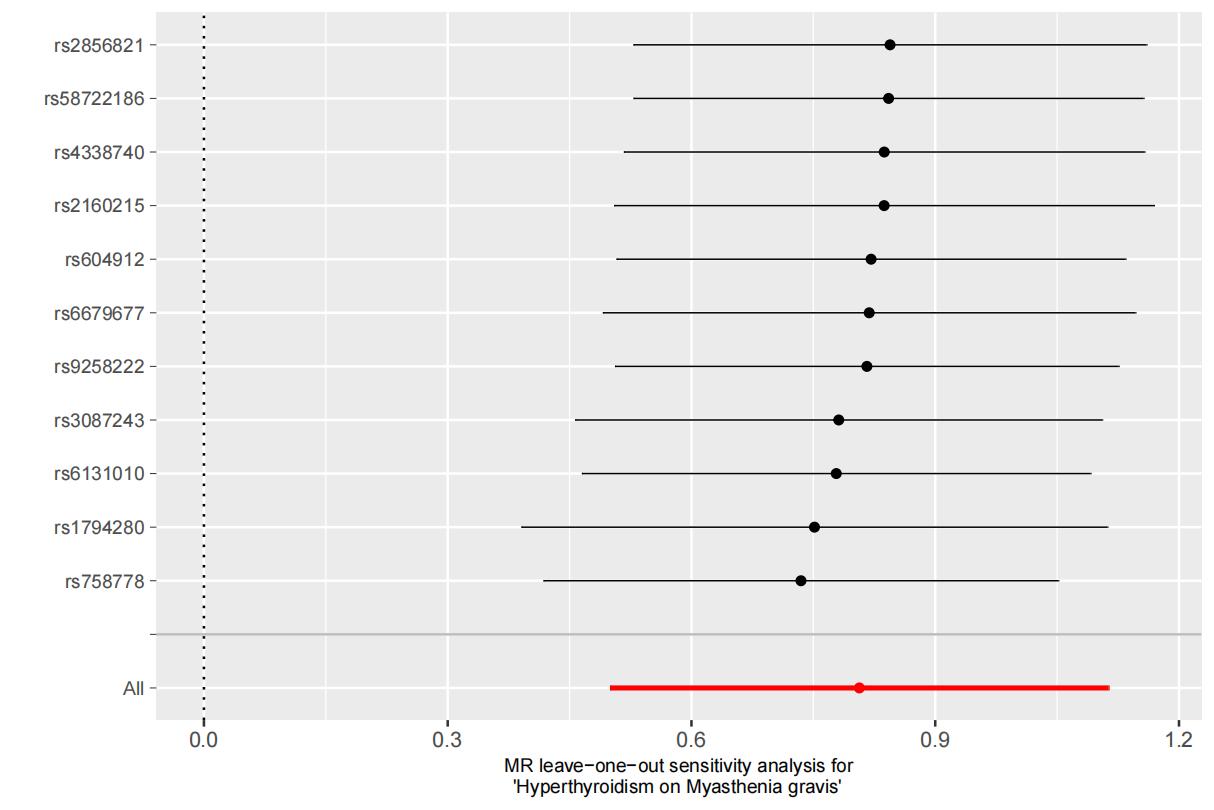


**Supplementary Figure 1T** MR leave-one-out sensitivity analysis for hyperthyroidism on myasthenia gravis

**Abbreviation:** MR**,** Mendelian randomization


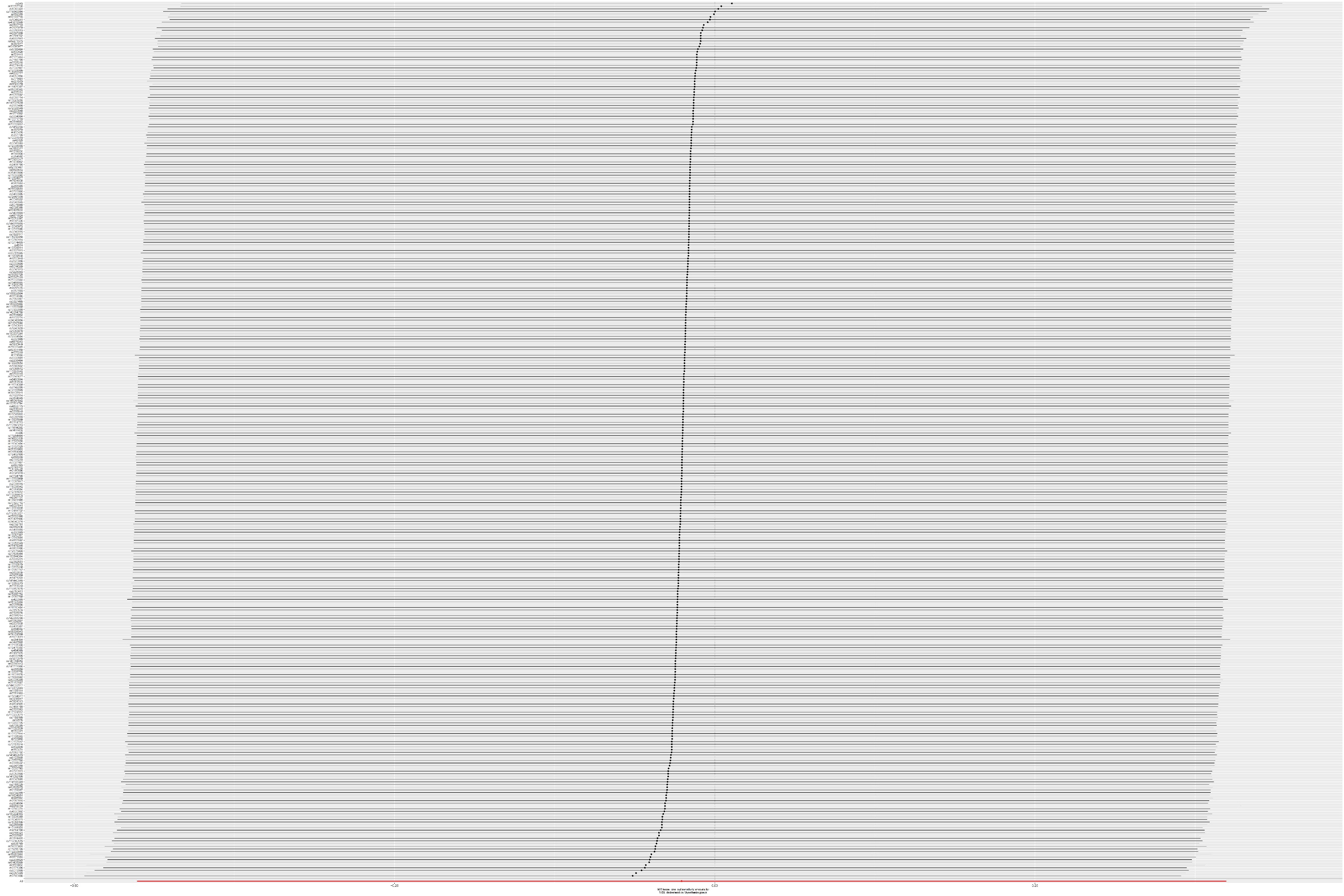


**Supplementary Figure 1U** MR leave-one-out sensitivity analysis for HDL cholesterol on myasthenia gravis

**Abbreviation:** MR**,** Mendelian randomization; HDL, high density lipoprotein


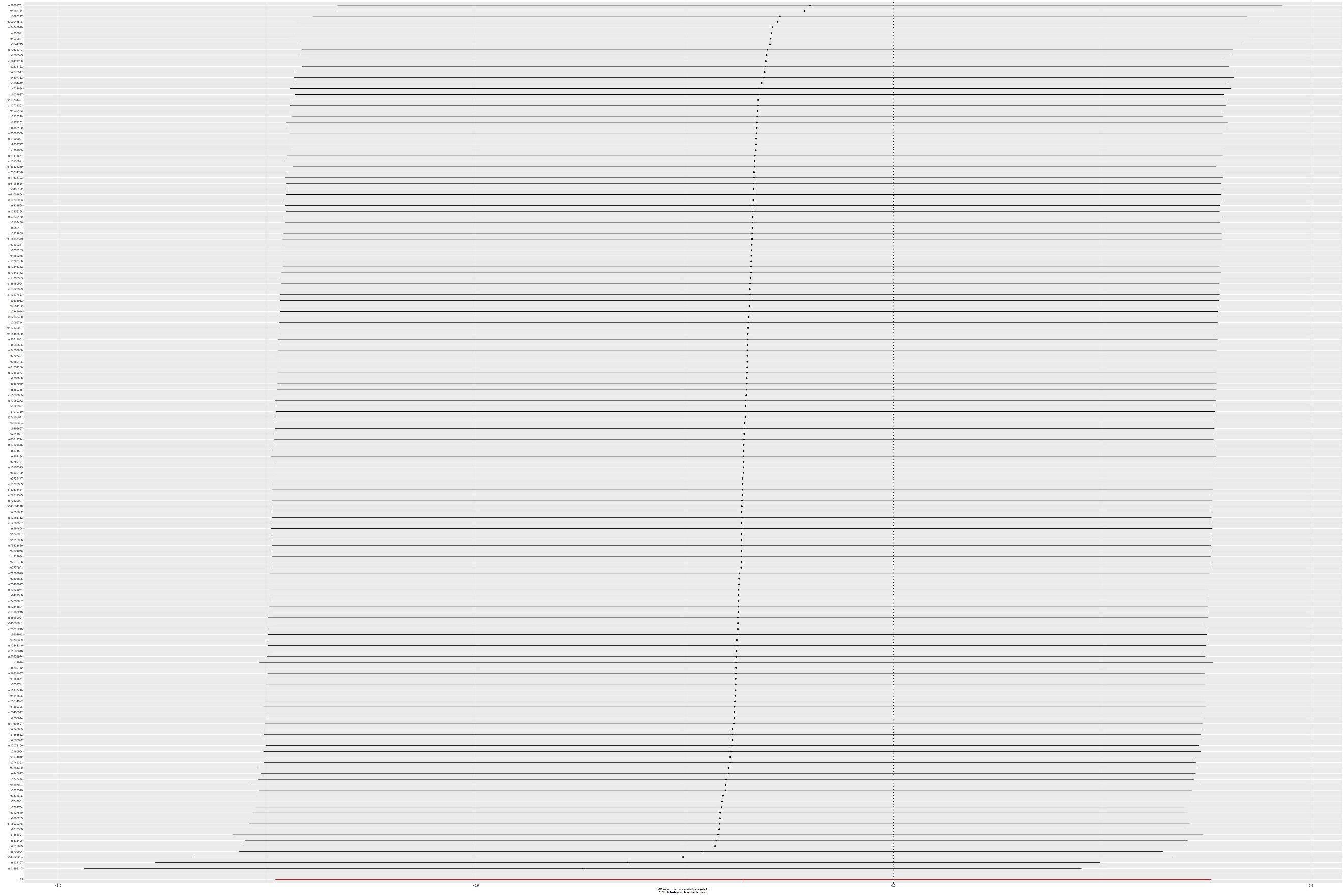


**Supplementary Figure 1V** MR leave-one-out sensitivity analysis for LDL cholesterol on myasthenia gravis

**Abbreviation:** MR**,** Mendelian randomization; LDL, low density lipoprotein


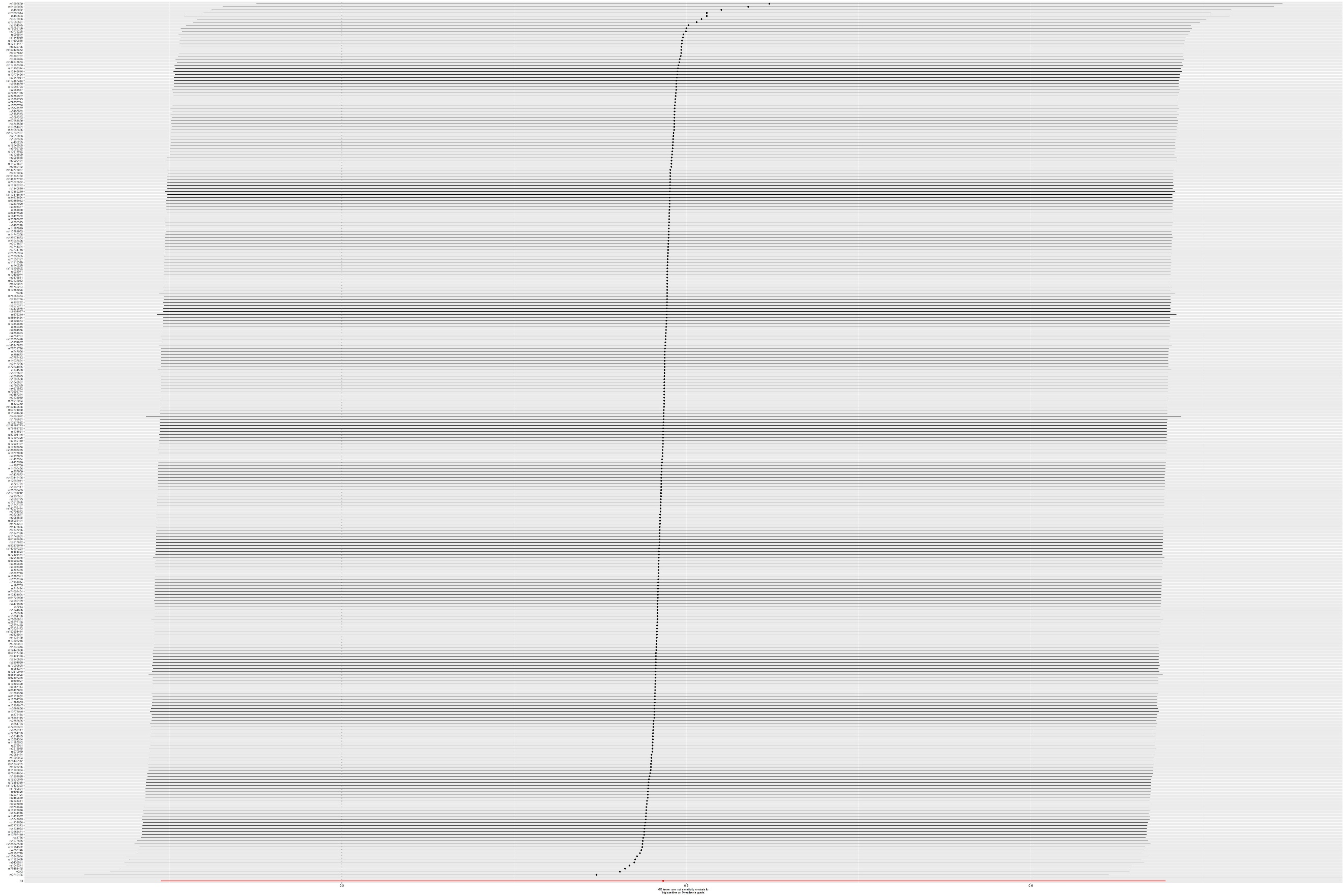


**Supplementary Figure 1W** MR leave-one-out sensitivity analysis for triglycerides on myasthenia gravis

**Abbreviation:** MR**,** Mendelian randomization


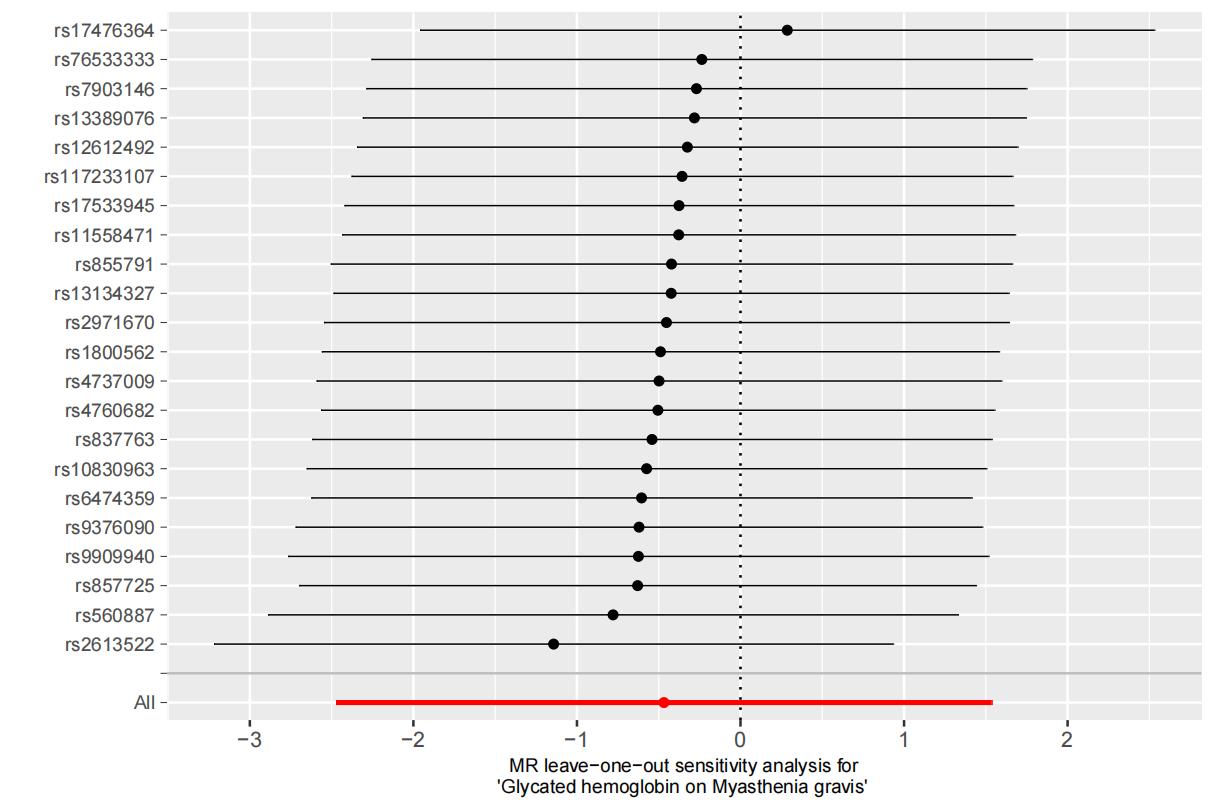


**Supplementary Figure 1X** MR leave-one-out sensitivity analysis for glycated hemoglobin on myasthenia gravis

**Abbreviation:** MR**,** Mendelian randomization


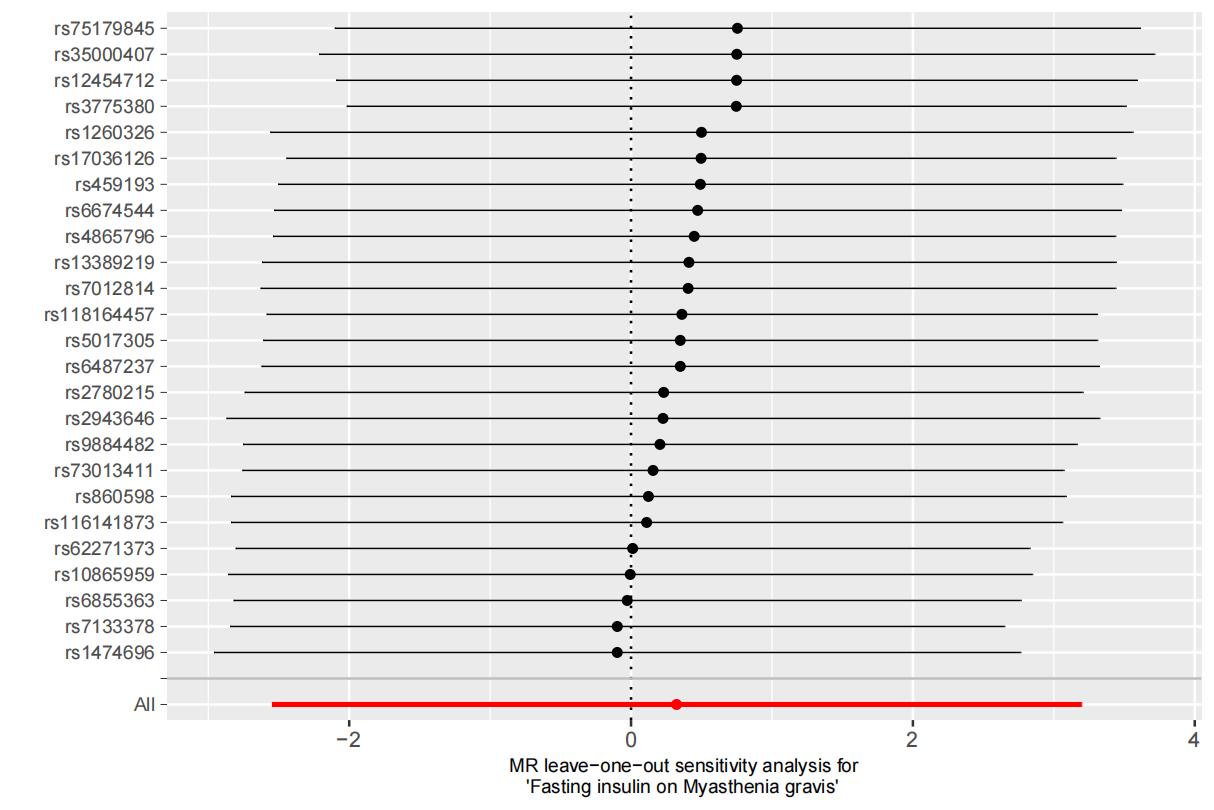


**Supplementary Figure 1Y** MR leave-one-out sensitivity analysis for fasting insulin on myasthenia gravis

**Abbreviation:** MR**,** Mendelian randomization


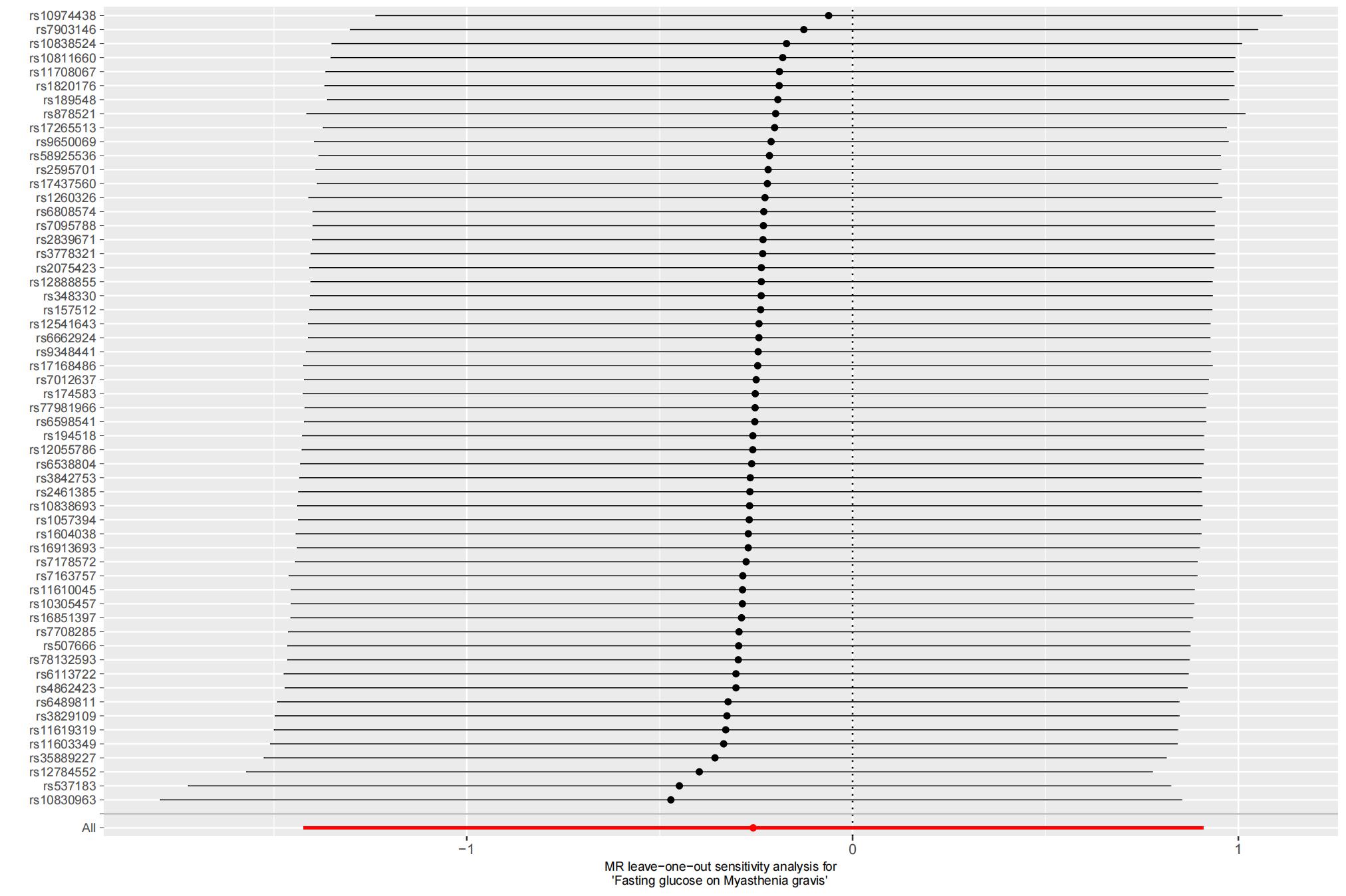


**Supplementary Figure 1Z** MR leave-one-out sensitivity analysis for fasting glucose on myasthenia gravis

**Abbreviation:** MR**,** Mendelian randomization
