# Supplementary material for: Genetic Insights Into Dietary Factors, Metabolic Traits and Myasthenia Gravis Risk: A Large‐Scale Two‐Sample Mendelian Randomization Study in European Populations
Source: Food Sci Nutr. 2025 May 26;13(6):e70236. doi: 10.1002/fsn3.70236 (PMC12121443; doi:10.1002/fsn3.70236)
Supplement: Supplementary file 2 — Supplementary Table 1. Single nucleotide polymorphisms (SNPs) were used as instrumental variables (IVs) from dietary habits and metabolic factor [Page 2–96]. Supplementary Table 2. Specific information for univariable MR analysis of dietary habits and metabolic factor as exposure and migraine as outcome [Page 97–102]. [file FSN3-13-e70236-s002.docx]

**Supplementary Table 1**. Single nucleotide polymorphisms (SNPs) were used as instrumental variables (IVs) from dietary habits and metabolic factor [Page 2-96]

**Supplementary Table 2**. Specific information for univariable MR analysis of dietary habits and metabolic factor as exposure and migraine as outcome [Page 97-102]

**Supplementary Table 1**. Single nucleotide polymorphisms (SNPs) were used as instrumental variables (IVs) from dietary habits and metabolic factor

| **Exposure** | **SNPs** | **EA** | **OA** | **EAF** | **Beta** | **SE** | **P value** | **R^2^** | **F** |
| --- | --- | --- | --- | --- | --- | --- | --- | --- | --- |
| Beef intake | rs11165829 | G | C | 0.360 | -0.010 | 0.002 | 9.80E-09 | 4.79E-05 | 22.104 |
| Beef intake | rs1105388 | T | C | 0.300 | -0.011 | 0.002 | 1.30E-09 | 5.44E-05 | 25.066 |
| Beef intake | rs10789340 | G | A | 0.627 | -0.014 | 0.002 | 6.80E-15 | 8.88E-05 | 40.965 |
| Beef intake | rs1470610 | C | G | 0.196 | -0.012 | 0.002 | 1.50E-08 | 4.71E-05 | 21.701 |
| Beef intake | rs62169335 | T | C | 0.543 | -0.010 | 0.002 | 2.40E-08 | 4.66E-05 | 21.468 |
| Beef intake | rs4676964 | T | C | 0.511 | 0.013 | 0.002 | 9.60E-15 | 8.91E-05 | 41.090 |
| Beef intake | rs62396185 | C | G | 0.260 | -0.015 | 0.002 | 2.80E-14 | 8.48E-05 | 39.104 |
| Beef intake | rs7791463 | A | G | 0.535 | 0.010 | 0.002 | 2.40E-08 | 4.54E-05 | 20.917 |
| Beef intake | rs79809011 | A | G | 0.029 | -0.028 | 0.005 | 3.40E-08 | 4.50E-05 | 20.762 |
| Beef intake | rs9407624 | A | T | 0.488 | -0.014 | 0.002 | 1.10E-15 | 9.47E-05 | 43.643 |
| Beef intake | rs10959890 | C | T | 0.212 | -0.013 | 0.002 | 1.50E-09 | 5.37E-05 | 24.739 |
| Beef intake | rs12247907 | C | G | 0.486 | 0.010 | 0.002 | 8.90E-09 | 4.84E-05 | 22.308 |
| Beef intake | rs1421085 | C | T | 0.403 | -0.012 | 0.002 | 3.50E-12 | 7.07E-05 | 32.619 |
| Beef intake | rs784251 | T | C | 0.478 | -0.010 | 0.002 | 1.70E-09 | 5.33E-05 | 24.593 |
| Beef intake | rs429358 | C | T | 0.154 | -0.015 | 0.002 | 3.60E-10 | 5.75E-05 | 26.531 |
| Beef intake | rs11878917 | A | G | 0.110 | 0.015 | 0.003 | 4.60E-08 | 4.41E-05 | 20.313 |
| Beef intake | rs132901 | T | C | 0.788 | 0.014 | 0.002 | 2.90E-11 | 6.49E-05 | 29.906 |
| Pork intake | rs11211124 | C | T | 0.231 | -0.010 | 0.002 | 1.40E-08 | 3.52E-05 | 16.175 |
| Pork intake | rs9973426 | G | A | 0.177 | 0.011 | 0.002 | 1.00E-08 | 3.57E-05 | 16.444 |
| Pork intake | rs7641973 | A | G | 0.353 | 0.008 | 0.002 | 4.20E-08 | 3.26E-05 | 15.002 |
| Pork intake | rs254152 | G | C | 0.235 | -0.010 | 0.002 | 2.20E-09 | 3.91E-05 | 17.986 |
| Pork intake | rs9379832 | G | A | 0.255 | -0.011 | 0.002 | 1.80E-11 | 5.01E-05 | 23.036 |
| Pork intake | rs10972033 | T | G | 0.456 | 0.009 | 0.001 | 1.30E-09 | 4.00E-05 | 18.395 |
| Pork intake | rs1355171 | A | C | 0.489 | -0.011 | 0.001 | 1.00E-13 | 6.03E-05 | 27.743 |
| Pork intake | rs34161520 | G | C | 0.160 | 0.012 | 0.002 | 9.60E-09 | 3.62E-05 | 16.653 |
| Pork intake | rs2387807 | T | C | 0.078 | -0.015 | 0.003 | 4.10E-08 | 3.27E-05 | 15.042 |
| Pork intake | rs4146837 | T | C | 0.456 | 0.009 | 0.001 | 4.00E-09 | 3.83E-05 | 17.646 |
| Pork intake | rs3964074 | C | T | 0.547 | -0.009 | 0.001 | 1.60E-09 | 3.97E-05 | 18.272 |
| Pork intake | rs36124222 | C | T | 0.433 | 0.008 | 0.002 | 2.10E-08 | 3.47E-05 | 15.981 |
| Pork intake | rs12721051 | G | C | 0.188 | -0.012 | 0.002 | 5.60E-11 | 4.67E-05 | 21.511 |
| Pork intake | rs838133 | G | A | 0.549 | 0.011 | 0.002 | 9.00E-13 | 5.88E-05 | 27.043 |
| Poultry intake | rs9997448 | T | C | 0.369 | -0.010 | 0.002 | 2.70E-08 | 5.10E-05 | 23.562 |
| Poultry intake | rs7829800 | G | A | 0.671 | 0.011 | 0.002 | 3.70E-09 | 5.80E-05 | 26.785 |
| Poultry intake | rs7046351 | A | T | 0.510 | 0.011 | 0.002 | 1.10E-09 | 6.13E-05 | 28.317 |
| Poultry intake | rs1051730 | A | G | 0.331 | -0.011 | 0.002 | 1.70E-08 | 5.24E-05 | 24.212 |
| Poultry intake | rs9923768 | A | G | 0.599 | 0.011 | 0.002 | 1.60E-08 | 5.31E-05 | 24.532 |
| Poultry intake | rs2565017 | A | G | 0.373 | 0.011 | 0.002 | 5.90E-09 | 5.61E-05 | 25.905 |
| Poultry intake | rs2965200 | A | G | 0.640 | -0.010 | 0.002 | 4.20E-08 | 5.00E-05 | 23.109 |
| Poultry intake | rs2426440 | G | A | 0.733 | 0.011 | 0.002 | 4.70E-08 | 4.93E-05 | 22.774 |
| Oily fish intake | rs973526 | T | C | 0.513 | -0.012 | 0.002 | 2.50E-09 | 6.62E-05 | 30.464 |
| Oily fish intake | rs45501495 | T | C | 0.236 | 0.016 | 0.002 | 3.70E-12 | 8.87E-05 | 40.829 |
| Oily fish intake | rs55930451 | T | C | 0.108 | -0.017 | 0.003 | 2.90E-08 | 5.60E-05 | 25.803 |
| Oily fish intake | rs55985303 | A | G | 0.241 | 0.013 | 0.002 | 6.60E-09 | 6.16E-05 | 28.361 |
| Oily fish intake | rs17050031 | T | C | 0.480 | -0.012 | 0.002 | 3.50E-10 | 7.25E-05 | 33.374 |
| Oily fish intake | rs275160 | C | T | 0.701 | 0.012 | 0.002 | 8.00E-09 | 6.16E-05 | 28.371 |
| Oily fish intake | rs13070166 | A | T | 0.229 | 0.014 | 0.002 | 4.40E-10 | 7.12E-05 | 32.794 |
| Oily fish intake | rs114497213 | T | G | 0.055 | 0.027 | 0.004 | 1.10E-10 | 7.73E-05 | 35.617 |
| Oily fish intake | rs10513136 | A | G | 0.065 | -0.023 | 0.004 | 1.60E-09 | 6.65E-05 | 30.631 |
| Oily fish intake | rs1876245 | C | T | 0.431 | 0.015 | 0.002 | 5.00E-15 | 1.12E-04 | 51.623 |
| Oily fish intake | rs10510554 | C | T | 0.569 | 0.011 | 0.002 | 1.20E-08 | 5.98E-05 | 27.546 |
| Oily fish intake | rs905575 | G | C | 0.824 | 0.014 | 0.003 | 3.60E-08 | 5.59E-05 | 25.743 |
| Oily fish intake | rs9841174 | C | T | 0.374 | 0.015 | 0.002 | 8.50E-14 | 1.02E-04 | 47.086 |
| Oily fish intake | rs1201289 | G | T | 0.395 | -0.011 | 0.002 | 4.40E-08 | 5.50E-05 | 25.311 |
| Oily fish intake | rs7683782 | G | C | 0.833 | 0.014 | 0.003 | 1.90E-08 | 5.82E-05 | 26.800 |
| Oily fish intake | rs10076975 | C | T | 0.381 | 0.011 | 0.002 | 1.10E-08 | 5.96E-05 | 27.448 |
| Oily fish intake | rs10061973 | T | G | 0.514 | -0.011 | 0.002 | 1.50E-08 | 5.88E-05 | 27.093 |
| Oily fish intake | rs16891727 | A | C | 0.130 | -0.024 | 0.003 | 6.80E-17 | 1.27E-04 | 58.523 |
| Oily fish intake | rs34555420 | T | G | 0.098 | -0.024 | 0.003 | 1.50E-13 | 9.97E-05 | 45.914 |
| Oily fish intake | rs12663865 | A | G | 0.758 | 0.013 | 0.002 | 1.10E-08 | 5.98E-05 | 27.543 |
| Oily fish intake | rs4869859 | C | T | 0.450 | 0.014 | 0.002 | 3.10E-13 | 9.72E-05 | 44.738 |
| Oily fish intake | rs11767283 | G | A | 0.222 | 0.018 | 0.002 | 2.50E-14 | 1.08E-04 | 49.639 |
| Oily fish intake | rs6465487 | G | A | 0.400 | -0.012 | 0.002 | 2.70E-10 | 7.32E-05 | 33.726 |
| Oily fish intake | rs11986122 | G | C | 0.423 | 0.015 | 0.002 | 2.90E-14 | 1.07E-04 | 49.339 |
| Oily fish intake | rs790564 | C | A | 0.723 | 0.015 | 0.002 | 7.90E-12 | 8.65E-05 | 39.809 |
| Oily fish intake | rs552234 | A | G | 0.495 | -0.012 | 0.002 | 1.10E-09 | 6.78E-05 | 31.232 |
| Oily fish intake | rs9886779 | A | T | 0.439 | -0.011 | 0.002 | 2.70E-08 | 5.66E-05 | 26.070 |
| Oily fish intake | rs10828250 | G | C | 0.309 | -0.020 | 0.002 | 2.60E-22 | 1.73E-04 | 79.866 |
| Oily fish intake | rs703987 | C | G | 0.615 | 0.011 | 0.002 | 1.70E-08 | 5.86E-05 | 26.972 |
| Oily fish intake | rs61882686 | A | C | 0.085 | 0.020 | 0.003 | 8.00E-09 | 6.09E-05 | 28.025 |
| Oily fish intake | rs4278546 | G | A | 0.441 | 0.013 | 0.002 | 9.30E-11 | 7.77E-05 | 35.791 |
| Oily fish intake | rs2374424 | G | A | 0.602 | -0.011 | 0.002 | 4.90E-09 | 6.28E-05 | 28.908 |
| Oily fish intake | rs510161 | G | C | 0.310 | -0.011 | 0.002 | 4.50E-08 | 5.47E-05 | 25.169 |
| Oily fish intake | rs631490 | C | G | 0.709 | -0.015 | 0.002 | 6.00E-13 | 9.46E-05 | 43.544 |
| Oily fish intake | rs303817 | G | A | 0.751 | 0.014 | 0.002 | 8.00E-10 | 6.89E-05 | 31.747 |
| Oily fish intake | rs35287743 | T | G | 0.116 | -0.028 | 0.003 | 7.00E-21 | 1.63E-04 | 75.141 |
| Oily fish intake | rs9597870 | G | T | 0.246 | -0.013 | 0.002 | 1.10E-08 | 6.02E-05 | 27.735 |
| Oily fish intake | rs3124402 | G | A | 0.733 | -0.022 | 0.002 | 1.90E-24 | 1.89E-04 | 87.203 |
| Oily fish intake | rs12855717 | T | C | 0.527 | -0.012 | 0.002 | 2.00E-10 | 7.46E-05 | 34.337 |
| Oily fish intake | rs1361016 | G | T | 0.845 | 0.015 | 0.003 | 1.70E-08 | 5.86E-05 | 27.004 |
| Oily fish intake | rs9301837 | A | C | 0.143 | -0.016 | 0.003 | 8.10E-09 | 6.08E-05 | 28.016 |
| Oily fish intake | rs4982738 | A | G | 0.583 | 0.011 | 0.002 | 3.50E-08 | 5.74E-05 | 26.411 |
| Oily fish intake | rs12896749 | C | G | 0.385 | -0.011 | 0.002 | 2.50E-08 | 5.69E-05 | 26.206 |
| Oily fish intake | rs1951286 | G | T | 0.645 | -0.015 | 0.002 | 3.00E-13 | 9.74E-05 | 44.853 |
| Oily fish intake | rs28533540 | A | G | 0.534 | 0.015 | 0.002 | 2.80E-14 | 1.07E-04 | 49.132 |
| Oily fish intake | rs1421085 | C | T | 0.403 | 0.018 | 0.002 | 2.50E-21 | 1.64E-04 | 75.713 |
| Oily fish intake | rs11859365 | C | A | 0.254 | 0.023 | 0.002 | 9.40E-25 | 1.93E-04 | 88.826 |
| Oily fish intake | rs9889161 | T | G | 0.358 | -0.013 | 0.002 | 2.80E-11 | 8.16E-05 | 37.552 |
| Oily fish intake | rs28623270 | T | A | 0.149 | -0.018 | 0.003 | 7.30E-11 | 8.05E-05 | 37.078 |
| Oily fish intake | rs2952140 | T | C | 0.483 | -0.011 | 0.002 | 2.50E-08 | 5.69E-05 | 26.216 |
| Oily fish intake | rs4510068 | T | G | 0.403 | -0.013 | 0.002 | 4.00E-11 | 8.15E-05 | 37.518 |
| Oily fish intake | rs7243428 | G | A | 0.225 | -0.013 | 0.002 | 1.50E-08 | 5.87E-05 | 27.019 |
| Oily fish intake | rs9958909 | G | T | 0.140 | 0.016 | 0.003 | 1.40E-08 | 5.97E-05 | 27.500 |
| Oily fish intake | rs59355765 | T | C | 0.160 | -0.016 | 0.003 | 4.70E-10 | 7.10E-05 | 32.706 |
| Oily fish intake | rs4002471 | T | C | 0.547 | -0.019 | 0.002 | 1.50E-23 | 1.84E-04 | 84.508 |
| Oily fish intake | rs7254235 | G | A | 0.577 | -0.011 | 0.002 | 4.30E-08 | 5.52E-05 | 25.396 |
| Oily fish intake | rs75887709 | G | A | 0.136 | -0.016 | 0.003 | 1.60E-08 | 5.94E-05 | 27.374 |
| Oily fish intake | rs12983532 | T | C | 0.251 | -0.013 | 0.002 | 2.00E-09 | 6.74E-05 | 31.051 |
| Oily fish intake | rs6033437 | A | C | 0.257 | 0.012 | 0.002 | 1.70E-08 | 5.94E-05 | 27.361 |
| Oily fish intake | rs6059844 | G | A | 0.495 | 0.011 | 0.002 | 9.20E-09 | 6.05E-05 | 27.856 |
| Oily fish intake | rs6089753 | T | C | 0.531 | -0.012 | 0.002 | 1.80E-09 | 6.64E-05 | 30.554 |
| Oily fish intake | rs2827161 | G | T | 0.423 | 0.011 | 0.002 | 3.20E-08 | 5.60E-05 | 25.787 |
| Oily fish intake | rs9606833 | C | T | 0.244 | 0.017 | 0.002 | 2.70E-14 | 1.06E-04 | 48.959 |
| Non-oily fish intake | rs16822430 | C | T | 0.233 | 0.012 | 0.002 | 1.40E-09 | 4.84E-05 | 22.313 |
| Non-oily fish intake | rs1260326 | C | T | 0.604 | -0.010 | 0.002 | 7.90E-09 | 4.37E-05 | 20.119 |
| Non-oily fish intake | rs11680516 | C | T | 0.202 | 0.012 | 0.002 | 1.40E-09 | 4.87E-05 | 22.455 |
| Non-oily fish intake | rs3799077 | G | T | 0.310 | -0.011 | 0.002 | 1.00E-09 | 4.93E-05 | 22.720 |
| Non-oily fish intake | rs4318925 | T | C | 0.177 | -0.015 | 0.002 | 1.30E-12 | 6.60E-05 | 30.398 |
| Non-oily fish intake | rs6957745 | C | T | 0.203 | -0.012 | 0.002 | 1.80E-09 | 4.80E-05 | 22.120 |
| Non-oily fish intake | rs17317920 | G | A | 0.479 | 0.009 | 0.002 | 2.80E-08 | 4.10E-05 | 18.885 |
| Non-oily fish intake | rs35287743 | T | G | 0.116 | -0.018 | 0.003 | 3.60E-12 | 6.45E-05 | 29.711 |
| Non-oily fish intake | rs7148387 | G | A | 0.591 | -0.009 | 0.002 | 1.70E-08 | 4.19E-05 | 19.302 |
| Non-oily fish intake | rs56094641 | G | A | 0.405 | 0.013 | 0.002 | 2.50E-14 | 7.63E-05 | 35.186 |
| Non-oily fish intake | rs838133 | G | A | 0.549 | 0.016 | 0.002 | 4.70E-22 | 1.30E-04 | 59.775 |
| Processed meat intake | rs7531118 | C | T | 0.531 | -0.014 | 0.002 | 2.80E-11 | 9.86E-05 | 45.567 |
| Processed meat intake | rs77165542 | T | C | 0.035 | 0.034 | 0.006 | 3.30E-09 | 7.86E-05 | 36.310 |
| Processed meat intake | rs11887120 | T | C | 0.398 | 0.012 | 0.002 | 3.10E-08 | 6.85E-05 | 31.671 |
| Processed meat intake | rs11894162 | T | C | 0.547 | 0.012 | 0.002 | 1.10E-08 | 7.19E-05 | 33.234 |
| Processed meat intake | rs4077924 | C | T | 0.702 | 0.012 | 0.002 | 4.50E-08 | 6.54E-05 | 30.203 |
| Processed meat intake | rs3762621 | T | C | 0.183 | -0.015 | 0.003 | 3.60E-08 | 6.73E-05 | 31.100 |
| Processed meat intake | rs9809856 | G | A | 0.476 | 0.013 | 0.002 | 2.50E-10 | 8.81E-05 | 40.699 |
| Processed meat intake | rs2873054 | C | A | 0.353 | 0.014 | 0.002 | 1.60E-10 | 8.95E-05 | 41.354 |
| Processed meat intake | rs6786550 | C | T | 0.635 | 0.012 | 0.002 | 2.10E-08 | 6.88E-05 | 31.807 |
| Processed meat intake | rs6765179 | A | G | 0.310 | -0.013 | 0.002 | 1.80E-08 | 6.97E-05 | 32.180 |
| Processed meat intake | rs10454812 | C | A | 0.103 | -0.020 | 0.003 | 6.70E-09 | 7.36E-05 | 34.021 |
| Processed meat intake | rs2029401 | G | A | 0.586 | 0.015 | 0.002 | 6.30E-12 | 1.04E-04 | 47.983 |
| Processed meat intake | rs1422192 | A | G | 0.158 | 0.017 | 0.003 | 3.40E-09 | 7.66E-05 | 35.407 |
| Processed meat intake | rs6961970 | A | C | 0.245 | -0.014 | 0.002 | 9.50E-09 | 7.26E-05 | 33.541 |
| Processed meat intake | rs4240672 | A | G | 0.494 | 0.017 | 0.002 | 3.00E-16 | 1.47E-04 | 67.703 |
| Processed meat intake | rs6484504 | C | T | 0.725 | 0.015 | 0.002 | 4.40E-11 | 9.55E-05 | 44.144 |
| Processed meat intake | rs11032380 | T | A | 0.333 | -0.013 | 0.002 | 2.10E-09 | 7.89E-05 | 36.449 |
| Processed meat intake | rs4778053 | G | C | 0.844 | 0.016 | 0.003 | 1.30E-08 | 7.15E-05 | 33.022 |
| Processed meat intake | rs34241936 | G | A | 0.037 | 0.033 | 0.006 | 1.10E-08 | 7.76E-05 | 35.841 |
| Processed meat intake | rs8096167 | C | T | 0.193 | -0.015 | 0.003 | 4.70E-08 | 6.62E-05 | 30.602 |
| Processed meat intake | rs838133 | G | A | 0.549 | 0.019 | 0.002 | 1.60E-18 | 1.79E-04 | 82.708 |
| Processed meat intake | rs6010651 | C | A | 0.379 | -0.012 | 0.002 | 1.10E-08 | 7.25E-05 | 33.486 |
| Processed meat intake | rs203319 | T | C | 0.205 | -0.016 | 0.003 | 2.80E-10 | 8.78E-05 | 40.558 |
| Tea intake | rs11587444 | G | A | 0.393 | 0.014 | 0.002 | 1.00E-10 | 9.40E-05 | 42.063 |
| Tea intake | rs11164870 | G | C | 0.605 | -0.012 | 0.002 | 4.20E-08 | 6.84E-05 | 30.609 |
| Tea intake | rs56188862 | C | T | 0.387 | -0.016 | 0.002 | 4.30E-13 | 1.18E-04 | 52.742 |
| Tea intake | rs1156588 | G | A | 0.210 | -0.015 | 0.003 | 2.90E-09 | 7.93E-05 | 35.471 |
| Tea intake | rs57462170 | A | G | 0.109 | 0.019 | 0.003 | 1.90E-08 | 7.11E-05 | 31.821 |
| Tea intake | rs2117137 | G | A | 0.405 | 0.013 | 0.002 | 1.70E-09 | 8.14E-05 | 36.425 |
| Tea intake | rs1481012 | G | A | 0.112 | -0.026 | 0.003 | 5.30E-15 | 1.37E-04 | 61.411 |
| Tea intake | rs34619 | A | G | 0.431 | 0.012 | 0.002 | 4.30E-08 | 6.73E-05 | 30.105 |
| Tea intake | rs72797284 | G | A | 0.271 | -0.017 | 0.002 | 7.00E-13 | 1.16E-04 | 51.771 |
| Tea intake | rs7757102 | G | A | 0.555 | -0.012 | 0.002 | 3.10E-08 | 6.88E-05 | 30.793 |
| Tea intake | rs2478875 | G | A | 0.209 | 0.022 | 0.003 | 5.10E-17 | 1.58E-04 | 70.874 |
| Tea intake | rs149805207 | G | A | 0.009 | -0.072 | 0.013 | 1.10E-08 | 8.76E-05 | 39.205 |
| Tea intake | rs4410790 | C | T | 0.631 | 0.041 | 0.002 | 3.40E-76 | 7.66E-04 | 342.831 |
| Tea intake | rs17685 | A | G | 0.278 | 0.023 | 0.002 | 1.60E-22 | 2.13E-04 | 95.485 |
| Tea intake | rs141071726 | A | G | 0.027 | 0.041 | 0.007 | 2.20E-09 | 8.63E-05 | 38.608 |
| Tea intake | rs9648476 | A | G | 0.623 | 0.013 | 0.002 | 1.10E-08 | 7.34E-05 | 32.855 |
| Tea intake | rs713598 | G | C | 0.402 | 0.013 | 0.002 | 5.20E-10 | 8.63E-05 | 38.625 |
| Tea intake | rs13282783 | T | C | 0.286 | -0.014 | 0.002 | 7.90E-09 | 7.53E-05 | 33.717 |
| Tea intake | rs56348300 | G | C | 0.185 | 0.016 | 0.003 | 6.10E-09 | 7.59E-05 | 33.987 |
| Tea intake | rs10764990 | A | G | 0.607 | -0.012 | 0.002 | 1.90E-08 | 7.09E-05 | 31.725 |
| Tea intake | rs10752269 | A | G | 0.506 | -0.013 | 0.002 | 1.30E-09 | 8.28E-05 | 37.073 |
| Tea intake | rs2351187 | A | G | 0.319 | 0.013 | 0.002 | 1.60E-08 | 7.23E-05 | 32.364 |
| Tea intake | rs17245213 | A | G | 0.208 | -0.015 | 0.003 | 2.00E-08 | 7.07E-05 | 31.642 |
| Tea intake | rs10741694 | C | T | 0.628 | 0.015 | 0.002 | 7.90E-12 | 1.05E-04 | 47.075 |
| Tea intake | rs1453548 | A | T | 0.665 | -0.013 | 0.002 | 3.00E-09 | 7.93E-05 | 35.494 |
| Tea intake | rs977474 | T | C | 0.834 | 0.022 | 0.003 | 2.40E-14 | 1.32E-04 | 58.862 |
| Tea intake | rs2783129 | G | C | 0.485 | -0.012 | 0.002 | 3.80E-08 | 6.88E-05 | 30.775 |
| Tea intake | rs17576658 | A | G | 0.247 | -0.013 | 0.002 | 4.10E-08 | 6.76E-05 | 30.261 |
| Tea intake | rs6829 | T | C | 0.596 | -0.012 | 0.002 | 3.70E-08 | 6.84E-05 | 30.598 |
| Tea intake | rs2645929 | G | A | 0.813 | -0.015 | 0.003 | 3.50E-08 | 6.83E-05 | 30.543 |
| Tea intake | rs12591786 | T | C | 0.159 | -0.018 | 0.003 | 3.70E-10 | 9.08E-05 | 40.656 |
| Tea intake | rs2472297 | T | C | 0.262 | 0.053 | 0.002 | 2.30E-109 | 1.10E-03 | 493.046 |
| Tea intake | rs9937354 | A | G | 0.424 | -0.014 | 0.002 | 4.90E-11 | 9.70E-05 | 43.413 |
| Tea intake | rs9302428 | G | C | 0.636 | 0.012 | 0.002 | 2.60E-08 | 6.94E-05 | 31.079 |
| Tea intake | rs2279844 | A | G | 0.379 | -0.012 | 0.002 | 4.00E-08 | 6.77E-05 | 30.283 |
| Tea intake | rs4808193 | C | T | 0.335 | 0.015 | 0.002 | 1.70E-11 | 1.02E-04 | 45.576 |
| Tea intake | rs57631352 | G | A | 0.297 | -0.013 | 0.002 | 1.70E-08 | 7.17E-05 | 32.078 |
| Tea intake | rs2273447 | T | A | 0.204 | 0.017 | 0.003 | 3.30E-11 | 9.91E-05 | 44.332 |
| Tea intake | rs4817505 | C | T | 0.390 | 0.015 | 0.002 | 4.20E-12 | 1.08E-04 | 48.345 |
| Tea intake | rs132904 | C | G | 0.779 | 0.017 | 0.003 | 7.80E-11 | 9.50E-05 | 42.513 |
| Tea intake | rs9624470 | A | G | 0.580 | 0.025 | 0.002 | 1.30E-31 | 3.10E-04 | 138.563 |
| Water intake | rs34967813 | G | A | 0.311 | -0.012 | 0.002 | 7.10E-10 | 6.23E-05 | 26.634 |
| Water intake | rs2305813 | C | G | 0.123 | -0.019 | 0.003 | 5.00E-12 | 7.90E-05 | 33.792 |
| Water intake | rs782221 | T | C | 0.200 | -0.013 | 0.002 | 1.90E-08 | 5.18E-05 | 22.142 |
| Water intake | rs182050989 | T | C | 0.028 | 0.031 | 0.005 | 2.40E-08 | 5.13E-05 | 21.955 |
| Water intake | rs11890994 | T | A | 0.372 | 0.011 | 0.002 | 3.60E-09 | 5.75E-05 | 24.584 |
| Water intake | rs2198234 | T | G | 0.529 | 0.012 | 0.002 | 1.30E-10 | 6.79E-05 | 29.023 |
| Water intake | rs11125629 | G | A | 0.547 | 0.011 | 0.002 | 2.50E-09 | 5.85E-05 | 25.030 |
| Water intake | rs9830293 | G | A | 0.073 | 0.019 | 0.003 | 4.00E-08 | 4.96E-05 | 21.196 |
| Water intake | rs7626335 | C | A | 0.668 | 0.011 | 0.002 | 2.50E-08 | 5.14E-05 | 21.981 |
| Water intake | rs6844845 | G | A | 0.057 | -0.022 | 0.004 | 3.30E-08 | 5.02E-05 | 21.447 |
| Water intake | rs6835325 | G | T | 0.301 | -0.012 | 0.002 | 2.00E-09 | 5.92E-05 | 25.330 |
| Water intake | rs67174962 | A | G | 0.232 | -0.012 | 0.002 | 1.30E-08 | 5.37E-05 | 22.943 |
| Water intake | rs6905712 | A | T | 0.665 | 0.011 | 0.002 | 8.90E-09 | 5.48E-05 | 23.423 |
| Water intake | rs2274156 | T | C | 0.259 | -0.011 | 0.002 | 2.90E-08 | 5.06E-05 | 21.637 |
| Water intake | rs6957745 | C | T | 0.203 | -0.015 | 0.002 | 1.40E-11 | 7.56E-05 | 32.320 |
| Water intake | rs3808058 | T | C | 0.118 | 0.018 | 0.003 | 7.50E-11 | 6.99E-05 | 29.869 |
| Water intake | rs4410790 | C | T | 0.630 | -0.031 | 0.002 | 8.60E-61 | 4.44E-04 | 190.091 |
| Water intake | rs10954732 | A | G | 0.671 | 0.011 | 0.002 | 3.60E-08 | 4.98E-05 | 21.282 |
| Water intake | rs2656285 | C | T | 0.710 | 0.012 | 0.002 | 1.10E-09 | 6.17E-05 | 26.394 |
| Water intake | rs9414686 | T | C | 0.178 | 0.015 | 0.002 | 8.60E-10 | 6.38E-05 | 27.261 |
| Water intake | rs10758255 | A | T | 0.610 | -0.011 | 0.002 | 6.70E-09 | 5.54E-05 | 23.678 |
| Water intake | rs11140831 | G | A | 0.514 | -0.012 | 0.002 | 1.50E-11 | 7.66E-05 | 32.755 |
| Water intake | rs35028442 | C | G | 0.137 | 0.016 | 0.003 | 9.90E-10 | 6.39E-05 | 27.322 |
| Water intake | rs11012726 | C | T | 0.307 | -0.012 | 0.002 | 6.20E-10 | 6.38E-05 | 27.281 |
| Water intake | rs3763874 | A | G | 0.418 | 0.016 | 0.002 | 6.90E-19 | 1.30E-04 | 55.470 |
| Water intake | rs7124005 | T | C | 0.415 | 0.010 | 0.002 | 2.50E-08 | 5.22E-05 | 22.318 |
| Water intake | rs2229357 | A | G | 0.240 | -0.012 | 0.002 | 7.90E-09 | 5.44E-05 | 23.265 |
| Water intake | rs34940743 | G | A | 0.347 | -0.010 | 0.002 | 4.40E-08 | 4.89E-05 | 20.922 |
| Water intake | rs146394874 | A | C | 0.049 | 0.026 | 0.004 | 5.80E-10 | 6.41E-05 | 27.406 |
| Water intake | rs1963510 | G | A | 0.458 | 0.013 | 0.002 | 2.60E-13 | 8.79E-05 | 37.594 |
| Water intake | rs4603502 | C | T | 0.292 | -0.013 | 0.002 | 5.40E-11 | 7.07E-05 | 30.237 |
| Water intake | rs2472297 | T | C | 0.261 | -0.033 | 0.002 | 2.40E-59 | 4.30E-04 | 184.113 |
| Water intake | rs8054636 | C | T | 0.537 | -0.010 | 0.002 | 2.40E-08 | 5.31E-05 | 22.692 |
| Water intake | rs2289292 | T | C | 0.334 | -0.013 | 0.002 | 3.40E-11 | 7.26E-05 | 31.034 |
| Water intake | rs1421085 | C | T | 0.404 | 0.012 | 0.002 | 7.70E-11 | 6.94E-05 | 29.696 |
| Water intake | rs2435200 | A | G | 0.413 | 0.011 | 0.002 | 1.10E-09 | 6.10E-05 | 26.067 |
| Water intake | rs4239466 | C | A | 0.392 | 0.011 | 0.002 | 2.00E-08 | 5.40E-05 | 23.085 |
| Water intake | rs9957088 | T | C | 0.253 | -0.012 | 0.002 | 1.40E-08 | 5.29E-05 | 22.630 |
| Water intake | rs429358 | C | T | 0.154 | 0.017 | 0.003 | 3.00E-11 | 7.26E-05 | 31.064 |
| Water intake | rs56100328 | G | A | 0.090 | -0.018 | 0.003 | 4.80E-08 | 5.06E-05 | 21.626 |
| Water intake | rs3746410 | G | A | 0.204 | 0.017 | 0.002 | 7.30E-14 | 9.21E-05 | 39.374 |
| Alcohol intake frequency | rs780569 | A | T | 0.709 | 0.020 | 0.003 | 4.00E-09 | 1.62E-04 | 74.858 |
| Alcohol intake frequency | rs4503294 | T | C | 0.565 | 0.018 | 0.003 | 3.40E-09 | 1.62E-04 | 74.845 |
| Alcohol intake frequency | rs28787109 | A | G | 0.404 | 0.018 | 0.003 | 7.70E-09 | 1.53E-04 | 70.653 |
| Alcohol intake frequency | rs2244598 | C | T | 0.605 | -0.018 | 0.003 | 3.80E-09 | 1.61E-04 | 74.640 |
| Alcohol intake frequency | rs4417025 | A | G | 0.361 | -0.019 | 0.003 | 2.70E-09 | 1.64E-04 | 75.722 |
| Alcohol intake frequency | rs7514579 | C | A | 0.232 | 0.020 | 0.004 | 4.60E-08 | 1.38E-04 | 63.824 |
| Alcohol intake frequency | rs2717063 | A | C | 0.586 | -0.020 | 0.003 | 4.00E-11 | 2.01E-04 | 93.124 |
| Alcohol intake frequency | rs6727281 | T | C | 0.184 | -0.024 | 0.004 | 5.50E-10 | 1.78E-04 | 82.153 |
| Alcohol intake frequency | rs780094 | C | T | 0.615 | -0.051 | 0.003 | 1.30E-60 | 1.23E-03 | 569.920 |
| Alcohol intake frequency | rs13390019 | C | T | 0.134 | 0.030 | 0.004 | 4.30E-11 | 2.04E-04 | 94.133 |
| Alcohol intake frequency | rs10188314 | T | C | 0.471 | -0.020 | 0.003 | 7.20E-11 | 1.95E-04 | 90.219 |
| Alcohol intake frequency | rs4241258 | T | C | 0.138 | 0.025 | 0.004 | 1.30E-08 | 1.49E-04 | 68.953 |
| Alcohol intake frequency | rs72769229 | T | A | 0.155 | -0.023 | 0.004 | 3.40E-08 | 1.40E-04 | 64.812 |
| Alcohol intake frequency | rs17662759 | C | T | 0.089 | 0.030 | 0.005 | 3.40E-08 | 1.47E-04 | 68.173 |
| Alcohol intake frequency | rs1991083 | T | C | 0.680 | -0.022 | 0.003 | 6.30E-12 | 2.18E-04 | 100.934 |
| Alcohol intake frequency | rs473098 | T | C | 0.558 | -0.022 | 0.003 | 9.10E-13 | 2.33E-04 | 107.835 |
| Alcohol intake frequency | rs9829192 | T | G | 0.435 | 0.017 | 0.003 | 2.80E-08 | 1.41E-04 | 65.172 |
| Alcohol intake frequency | rs76082653 | T | C | 0.054 | 0.046 | 0.007 | 3.80E-12 | 2.21E-04 | 102.421 |
| Alcohol intake frequency | rs262240 | T | C | 0.469 | -0.017 | 0.003 | 1.40E-08 | 1.47E-04 | 68.185 |
| Alcohol intake frequency | rs9814516 | T | G | 0.237 | -0.025 | 0.004 | 1.60E-12 | 2.28E-04 | 105.597 |
| Alcohol intake frequency | rs7610856 | A | C | 0.429 | -0.024 | 0.003 | 7.70E-15 | 2.79E-04 | 129.036 |
| Alcohol intake frequency | rs1515591 | G | T | 0.383 | 0.018 | 0.003 | 4.90E-09 | 1.57E-04 | 72.647 |
| Alcohol intake frequency | rs1228589 | A | G | 0.246 | 0.021 | 0.004 | 2.30E-09 | 1.65E-04 | 76.183 |
| Alcohol intake frequency | rs28622224 | T | C | 0.280 | -0.019 | 0.003 | 3.20E-08 | 1.40E-04 | 64.694 |
| Alcohol intake frequency | rs13135092 | G | A | 0.083 | 0.044 | 0.005 | 1.60E-15 | 2.94E-04 | 135.983 |
| Alcohol intake frequency | rs11940694 | G | A | 0.604 | -0.044 | 0.003 | 1.00E-44 | 9.14E-04 | 422.950 |
| Alcohol intake frequency | rs362307 | T | C | 0.075 | 0.043 | 0.006 | 8.40E-14 | 2.59E-04 | 119.715 |
| Alcohol intake frequency | rs1229984 | C | T | 0.973 | -0.262 | 0.009 | 1.40E-178 | 3.63E-03 | 1683.704 |
| Alcohol intake frequency | rs13102973 | C | T | 0.619 | -0.019 | 0.003 | 4.90E-10 | 1.78E-04 | 82.167 |
| Alcohol intake frequency | rs62339673 | A | C | 0.627 | 0.018 | 0.003 | 6.60E-09 | 1.57E-04 | 72.412 |
| Alcohol intake frequency | rs34811474 | A | G | 0.231 | -0.020 | 0.004 | 1.90E-08 | 1.45E-04 | 66.853 |
| Alcohol intake frequency | rs2159935 | A | G | 0.490 | -0.019 | 0.003 | 8.30E-10 | 1.72E-04 | 79.739 |
| Alcohol intake frequency | rs62305780 | G | C | 0.102 | -0.049 | 0.005 | 9.90E-22 | 4.32E-04 | 199.932 |
| Alcohol intake frequency | rs13178443 | T | C | 0.276 | -0.019 | 0.003 | 3.80E-08 | 1.39E-04 | 64.339 |
| Alcohol intake frequency | rs11750777 | A | G | 0.209 | -0.020 | 0.004 | 3.80E-08 | 1.39E-04 | 64.311 |
| Alcohol intake frequency | rs4916723 | C | A | 0.421 | 0.024 | 0.003 | 1.10E-14 | 2.80E-04 | 129.272 |
| Alcohol intake frequency | rs461599 | C | A | 0.462 | -0.019 | 0.003 | 2.70E-10 | 1.83E-04 | 84.650 |
| Alcohol intake frequency | rs56194430 | T | C | 0.169 | 0.023 | 0.004 | 3.10E-08 | 1.43E-04 | 66.084 |
| Alcohol intake frequency | rs9403297 | A | G | 0.373 | 0.019 | 0.003 | 1.80E-09 | 1.66E-04 | 76.634 |
| Alcohol intake frequency | rs9349379 | G | A | 0.405 | -0.019 | 0.003 | 3.50E-10 | 1.80E-04 | 83.440 |
| Alcohol intake frequency | rs12153855 | C | T | 0.105 | 0.029 | 0.005 | 2.40E-09 | 1.63E-04 | 75.326 |
| Alcohol intake frequency | rs9372625 | A | G | 0.382 | -0.026 | 0.003 | 2.90E-16 | 3.08E-04 | 142.595 |
| Alcohol intake frequency | rs62466318 | T | C | 0.203 | -0.025 | 0.004 | 1.40E-11 | 2.10E-04 | 97.178 |
| Alcohol intake frequency | rs2622167 | A | G | 0.429 | -0.019 | 0.003 | 4.60E-10 | 1.79E-04 | 82.766 |
| Alcohol intake frequency | rs73050128 | A | C | 0.164 | -0.026 | 0.004 | 2.10E-10 | 1.86E-04 | 85.955 |
| Alcohol intake frequency | rs6943160 | C | T | 0.209 | 0.021 | 0.004 | 3.10E-08 | 1.41E-04 | 64.969 |
| Alcohol intake frequency | rs4726481 | T | G | 0.401 | 0.022 | 0.003 | 2.30E-12 | 2.27E-04 | 105.169 |
| Alcohol intake frequency | rs9648478 | A | G | 0.510 | 0.017 | 0.003 | 2.60E-08 | 1.42E-04 | 65.697 |
| Alcohol intake frequency | rs2160935 | T | C | 0.604 | -0.019 | 0.003 | 1.40E-09 | 1.68E-04 | 77.483 |
| Alcohol intake frequency | rs34440851 | T | C | 0.157 | -0.023 | 0.004 | 4.60E-08 | 1.36E-04 | 63.027 |
| Alcohol intake frequency | rs11787216 | T | C | 0.369 | 0.024 | 0.003 | 2.40E-14 | 2.78E-04 | 128.407 |
| Alcohol intake frequency | rs2977454 | G | C | 0.124 | -0.026 | 0.005 | 1.70E-08 | 1.46E-04 | 67.537 |
| Alcohol intake frequency | rs74679146 | C | T | 0.075 | -0.032 | 0.006 | 2.50E-08 | 1.42E-04 | 65.609 |
| Alcohol intake frequency | rs489062 | A | G | 0.437 | 0.017 | 0.003 | 4.90E-08 | 1.36E-04 | 63.090 |
| Alcohol intake frequency | rs34473884 | A | G | 0.248 | -0.020 | 0.004 | 6.20E-09 | 1.55E-04 | 71.544 |
| Alcohol intake frequency | rs61873510 | T | G | 0.328 | 0.020 | 0.003 | 6.90E-10 | 1.83E-04 | 84.597 |
| Alcohol intake frequency | rs4242715 | A | G | 0.681 | -0.019 | 0.003 | 9.30E-09 | 1.51E-04 | 69.961 |
| Alcohol intake frequency | rs10792669 | G | A | 0.505 | 0.017 | 0.003 | 9.90E-09 | 1.52E-04 | 70.252 |
| Alcohol intake frequency | rs11223617 | A | G | 0.206 | 0.025 | 0.004 | 2.30E-11 | 2.06E-04 | 95.289 |
| Alcohol intake frequency | rs550942 | T | C | 0.824 | 0.022 | 0.004 | 2.00E-08 | 1.46E-04 | 67.343 |
| Alcohol intake frequency | rs11039429 | T | C | 0.455 | -0.024 | 0.003 | 8.70E-15 | 2.75E-04 | 127.290 |
| Alcohol intake frequency | rs1666658 | C | T | 0.392 | 0.018 | 0.003 | 6.70E-09 | 1.54E-04 | 71.171 |
| Alcohol intake frequency | rs12312693 | C | T | 0.452 | -0.018 | 0.003 | 6.80E-09 | 1.55E-04 | 71.608 |
| Alcohol intake frequency | rs7302200 | A | G | 0.340 | -0.018 | 0.003 | 8.40E-09 | 1.52E-04 | 70.431 |
| Alcohol intake frequency | rs28768122 | C | T | 0.760 | 0.021 | 0.004 | 5.60E-09 | 1.57E-04 | 72.380 |
| Alcohol intake frequency | rs7298932 | G | A | 0.148 | -0.024 | 0.004 | 3.80E-08 | 1.42E-04 | 65.565 |
| Alcohol intake frequency | rs58905411 | A | G | 0.410 | -0.027 | 0.003 | 5.10E-18 | 3.43E-04 | 158.738 |
| Alcohol intake frequency | rs1937522 | G | A | 0.528 | 0.017 | 0.003 | 2.50E-08 | 1.42E-04 | 65.810 |
| Alcohol intake frequency | rs7330939 | T | C | 0.720 | -0.021 | 0.003 | 3.70E-10 | 1.83E-04 | 84.765 |
| Alcohol intake frequency | rs2535911 | T | C | 0.355 | -0.019 | 0.003 | 2.70E-09 | 1.63E-04 | 75.202 |
| Alcohol intake frequency | rs186347 | T | G | 0.463 | 0.018 | 0.003 | 4.00E-09 | 1.60E-04 | 74.087 |
| Alcohol intake frequency | rs80292319 | C | T | 0.058 | -0.039 | 0.006 | 1.40E-09 | 1.69E-04 | 77.957 |
| Alcohol intake frequency | rs117799466 | C | G | 0.337 | -0.020 | 0.003 | 3.10E-09 | 1.73E-04 | 79.953 |
| Alcohol intake frequency | rs34631026 | T | C | 0.446 | -0.017 | 0.003 | 2.90E-08 | 1.41E-04 | 65.365 |
| Alcohol intake frequency | rs72787062 | A | G | 0.163 | -0.028 | 0.004 | 6.40E-12 | 2.17E-04 | 100.193 |
| Alcohol intake frequency | rs35105141 | T | C | 0.402 | 0.026 | 0.003 | 1.40E-17 | 3.34E-04 | 154.277 |
| Alcohol intake frequency | rs1421085 | C | T | 0.403 | 0.020 | 0.003 | 1.00E-10 | 1.91E-04 | 88.497 |
| Alcohol intake frequency | rs1104608 | C | G | 0.426 | 0.017 | 0.003 | 1.70E-08 | 1.48E-04 | 68.648 |
| Alcohol intake frequency | rs8043563 | C | G | 0.737 | 0.023 | 0.003 | 1.70E-11 | 2.12E-04 | 97.826 |
| Alcohol intake frequency | rs2411453 | G | T | 0.597 | -0.035 | 0.003 | 7.30E-30 | 5.92E-04 | 273.848 |
| Alcohol intake frequency | rs728538 | G | T | 0.169 | 0.023 | 0.004 | 1.80E-08 | 1.47E-04 | 67.921 |
| Alcohol intake frequency | rs9906502 | A | G | 0.177 | 0.024 | 0.004 | 1.90E-09 | 1.65E-04 | 76.236 |
| Alcohol intake frequency | rs8614 | A | C | 0.183 | 0.025 | 0.004 | 2.70E-10 | 1.83E-04 | 84.736 |
| Alcohol intake frequency | rs4968391 | T | G | 0.675 | -0.019 | 0.003 | 2.30E-09 | 1.63E-04 | 75.347 |
| Alcohol intake frequency | rs9912298 | C | A | 0.240 | 0.021 | 0.004 | 9.70E-09 | 1.54E-04 | 71.427 |
| Alcohol intake frequency | rs17690703 | T | C | 0.263 | 0.025 | 0.003 | 2.90E-13 | 2.43E-04 | 112.269 |
| Alcohol intake frequency | rs650558 | T | C | 0.248 | 0.021 | 0.004 | 3.40E-09 | 1.60E-04 | 74.147 |
| Alcohol intake frequency | rs1893659 | A | C | 0.460 | -0.029 | 0.003 | 7.60E-22 | 4.27E-04 | 197.619 |
| Alcohol intake frequency | rs5022348 | T | C | 0.407 | 0.020 | 0.004 | 1.40E-08 | 1.98E-04 | 91.663 |
| Alcohol intake frequency | rs2043677 | T | C | 0.146 | 0.026 | 0.004 | 1.60E-09 | 1.70E-04 | 78.454 |
| Alcohol intake frequency | rs9958320 | C | T | 0.153 | 0.025 | 0.004 | 5.90E-09 | 1.60E-04 | 74.100 |
| Alcohol intake frequency | rs62097995 | A | T | 0.424 | 0.020 | 0.003 | 6.90E-11 | 1.95E-04 | 90.349 |
| Alcohol intake frequency | rs2924321 | A | G | 0.540 | -0.020 | 0.003 | 1.60E-10 | 1.89E-04 | 87.486 |
| Alcohol intake frequency | rs4940926 | C | T | 0.735 | -0.019 | 0.003 | 2.80E-08 | 1.42E-04 | 65.709 |
| Alcohol intake frequency | rs838145 | A | G | 0.543 | 0.022 | 0.003 | 6.70E-13 | 2.39E-04 | 110.631 |
| Alcohol intake frequency | rs6030200 | A | G | 0.314 | -0.020 | 0.003 | 2.40E-09 | 1.64E-04 | 75.996 |
| Alcohol intake frequency | rs11700855 | G | A | 0.093 | -0.030 | 0.005 | 1.20E-08 | 1.50E-04 | 69.563 |
| Alcohol intake frequency | rs71651683 | T | C | 0.014 | -0.070 | 0.013 | 3.60E-08 | 1.39E-04 | 64.269 |
| Alcohol intake frequency | rs1894544 | C | G | 0.454 | 0.017 | 0.003 | 1.10E-08 | 1.50E-04 | 69.364 |
| Bread intake | rs9662365 | T | C | 0.499 | 0.012 | 0.002 | 9.60E-10 | 7.40E-05 | 33.471 |
| Bread intake | rs13023099 | A | C | 0.572 | -0.011 | 0.002 | 1.40E-08 | 6.44E-05 | 29.141 |
| Bread intake | rs6754311 | C | T | 0.264 | 0.014 | 0.002 | 3.10E-10 | 7.77E-05 | 35.158 |
| Bread intake | rs4665972 | C | T | 0.605 | -0.014 | 0.002 | 3.20E-12 | 9.69E-05 | 43.827 |
| Bread intake | rs75287965 | A | G | 0.063 | -0.025 | 0.004 | 1.50E-09 | 7.22E-05 | 32.669 |
| Bread intake | rs13016665 | A | C | 0.423 | 0.015 | 0.002 | 3.50E-13 | 1.06E-04 | 48.069 |
| Bread intake | rs1492988 | G | C | 0.599 | 0.012 | 0.002 | 1.40E-08 | 6.40E-05 | 28.955 |
| Bread intake | rs9832088 | A | T | 0.522 | 0.015 | 0.002 | 1.30E-13 | 1.08E-04 | 48.968 |
| Bread intake | rs9881332 | G | C | 0.582 | 0.011 | 0.002 | 1.90E-08 | 6.29E-05 | 28.452 |
| Bread intake | rs1994315 | C | T | 0.685 | -0.017 | 0.002 | 4.30E-15 | 1.22E-04 | 55.231 |
| Bread intake | rs73802707 | T | C | 0.154 | -0.016 | 0.003 | 8.00E-09 | 6.60E-05 | 29.855 |
| Bread intake | rs2068650 | C | A | 0.472 | -0.014 | 0.002 | 3.10E-12 | 9.68E-05 | 43.794 |
| Bread intake | rs17083079 | A | G | 0.047 | 0.030 | 0.005 | 1.20E-10 | 8.19E-05 | 37.055 |
| Bread intake | rs2517678 | T | C | 0.368 | 0.013 | 0.002 | 2.20E-10 | 8.15E-05 | 36.856 |
| Bread intake | rs596878 | C | A | 0.450 | -0.012 | 0.002 | 5.30E-09 | 6.82E-05 | 30.845 |
| Bread intake | rs79436018 | C | T | 0.116 | -0.018 | 0.003 | 1.60E-08 | 6.38E-05 | 28.839 |
| Bread intake | rs7802468 | T | C | 0.372 | -0.023 | 0.002 | 6.90E-30 | 2.55E-04 | 115.197 |
| Bread intake | rs10761661 | T | C | 0.453 | -0.011 | 0.002 | 1.00E-08 | 6.54E-05 | 29.568 |
| Bread intake | rs55745436 | T | C | 0.237 | 0.013 | 0.002 | 1.00E-08 | 6.52E-05 | 29.501 |
| Bread intake | rs1940033 | T | C | 0.593 | -0.011 | 0.002 | 4.70E-08 | 5.93E-05 | 26.802 |
| Bread intake | rs11183201 | C | T | 0.508 | -0.017 | 0.002 | 5.30E-17 | 1.40E-04 | 63.244 |
| Bread intake | rs6580721 | G | A | 0.189 | 0.017 | 0.003 | 1.30E-11 | 9.06E-05 | 40.986 |
| Bread intake | rs11060853 | G | A | 0.412 | -0.013 | 0.002 | 2.50E-10 | 7.92E-05 | 35.835 |
| Bread intake | rs9564268 | C | T | 0.616 | -0.012 | 0.002 | 3.00E-09 | 7.00E-05 | 31.652 |
| Bread intake | rs9529024 | T | A | 0.370 | -0.013 | 0.002 | 4.20E-10 | 7.78E-05 | 35.195 |
| Bread intake | rs11628639 | C | T | 0.243 | -0.013 | 0.002 | 6.20E-09 | 6.71E-05 | 30.330 |
| Bread intake | rs9323989 | C | T | 0.379 | -0.012 | 0.002 | 1.60E-08 | 6.35E-05 | 28.722 |
| Bread intake | rs28406095 | A | G | 0.462 | -0.011 | 0.002 | 4.40E-08 | 5.96E-05 | 26.950 |
| Bread intake | rs4984685 | A | G | 0.201 | 0.014 | 0.002 | 4.40E-08 | 5.93E-05 | 26.809 |
| Bread intake | rs62091167 | C | A | 0.216 | -0.014 | 0.002 | 1.20E-08 | 6.49E-05 | 29.334 |
| Bread intake | rs656817 | G | A | 0.334 | -0.013 | 0.002 | 1.80E-09 | 7.17E-05 | 32.414 |
| Bread intake | rs7276867 | C | G | 0.542 | 0.011 | 0.002 | 1.50E-08 | 6.39E-05 | 28.918 |
| Cheese intake | rs78876700 | A | G | 0.137 | 0.018 | 0.003 | 3.40E-08 | 7.76E-05 | 35.057 |
| Cheese intake | rs531358 | T | C | 0.650 | 0.013 | 0.002 | 1.80E-08 | 7.89E-05 | 35.626 |
| Cheese intake | rs2802530 | A | G | 0.877 | 0.019 | 0.003 | 4.20E-08 | 7.51E-05 | 33.914 |
| Cheese intake | rs6685323 | T | C | 0.309 | -0.013 | 0.002 | 4.80E-08 | 7.43E-05 | 33.556 |
| Cheese intake | rs2339928 | A | G | 0.704 | 0.015 | 0.002 | 1.20E-09 | 9.20E-05 | 41.536 |
| Cheese intake | rs12475594 | G | A | 0.178 | 0.016 | 0.003 | 4.40E-08 | 7.51E-05 | 33.924 |
| Cheese intake | rs504675 | T | C | 0.353 | 0.027 | 0.002 | 1.00E-31 | 3.44E-04 | 155.282 |
| Cheese intake | rs72970243 | A | G | 0.120 | 0.022 | 0.003 | 6.70E-11 | 1.04E-04 | 47.152 |
| Cheese intake | rs1514755 | G | A | 0.240 | 0.016 | 0.003 | 3.90E-10 | 9.77E-05 | 44.116 |
| Cheese intake | rs79184944 | A | T | 0.134 | 0.020 | 0.003 | 2.40E-09 | 8.94E-05 | 40.353 |
| Cheese intake | rs4296548 | G | T | 0.610 | 0.013 | 0.002 | 1.20E-08 | 8.07E-05 | 36.457 |
| Cheese intake | rs62245792 | A | T | 0.150 | -0.018 | 0.003 | 1.40E-08 | 8.20E-05 | 37.042 |
| Cheese intake | rs77742462 | G | A | 0.021 | -0.047 | 0.008 | 9.80E-09 | 9.06E-05 | 40.904 |
| Cheese intake | rs2352974 | T | C | 0.490 | -0.014 | 0.002 | 1.00E-10 | 1.05E-04 | 47.429 |
| Cheese intake | rs6774906 | C | A | 0.041 | 0.032 | 0.006 | 2.50E-08 | 7.80E-05 | 35.234 |
| Cheese intake | rs4681981 | A | C | 0.469 | -0.012 | 0.002 | 2.90E-08 | 7.70E-05 | 34.782 |
| Cheese intake | rs4860341 | C | T | 0.929 | 0.024 | 0.004 | 2.20E-08 | 7.86E-05 | 35.479 |
| Cheese intake | rs73096946 | C | T | 0.157 | -0.021 | 0.003 | 1.90E-11 | 1.12E-04 | 50.782 |
| Cheese intake | rs13107325 | T | C | 0.075 | -0.029 | 0.004 | 7.00E-12 | 1.18E-04 | 53.086 |
| Cheese intake | rs10938397 | G | A | 0.434 | -0.013 | 0.002 | 1.80E-08 | 7.93E-05 | 35.827 |
| Cheese intake | rs4692708 | C | A | 0.253 | 0.015 | 0.003 | 1.30E-08 | 8.19E-05 | 36.996 |
| Cheese intake | rs26579 | C | G | 0.586 | -0.013 | 0.002 | 2.40E-08 | 7.95E-05 | 35.893 |
| Cheese intake | rs6873324 | C | A | 0.426 | -0.012 | 0.002 | 3.90E-08 | 7.61E-05 | 34.369 |
| Cheese intake | rs9504123 | C | A | 0.275 | 0.014 | 0.003 | 1.50E-08 | 8.00E-05 | 36.133 |
| Cheese intake | rs975303 | G | A | 0.181 | 0.021 | 0.003 | 2.50E-13 | 1.34E-04 | 60.679 |
| Cheese intake | rs1931805 | C | T | 0.500 | 0.013 | 0.002 | 1.60E-08 | 7.98E-05 | 36.048 |
| Cheese intake | rs113367286 | T | C | 0.278 | 0.015 | 0.002 | 1.30E-09 | 9.26E-05 | 41.809 |
| Cheese intake | rs34198643 | T | C | 0.224 | -0.017 | 0.003 | 4.50E-10 | 9.70E-05 | 43.804 |
| Cheese intake | rs12672200 | A | G | 0.326 | -0.014 | 0.002 | 9.00E-09 | 8.32E-05 | 37.575 |
| Cheese intake | rs9649582 | T | A | 0.317 | -0.015 | 0.002 | 1.40E-09 | 9.26E-05 | 41.790 |
| Cheese intake | rs7012814 | A | G | 0.474 | -0.019 | 0.002 | 2.10E-16 | 1.71E-04 | 77.198 |
| Cheese intake | rs7386207 | T | C | 0.564 | -0.012 | 0.002 | 3.60E-08 | 7.68E-05 | 34.691 |
| Cheese intake | rs13257887 | C | T | 0.359 | 0.016 | 0.003 | 2.70E-10 | 1.20E-04 | 54.406 |
| Cheese intake | rs3911016 | G | T | 0.121 | 0.021 | 0.003 | 5.30E-10 | 9.70E-05 | 43.791 |
| Cheese intake | rs4503172 | T | C | 0.608 | 0.013 | 0.002 | 1.60E-08 | 8.00E-05 | 36.138 |
| Cheese intake | rs1806771 | G | T | 0.088 | -0.022 | 0.004 | 4.10E-08 | 7.86E-05 | 35.502 |
| Cheese intake | rs73335955 | C | T | 0.053 | 0.028 | 0.005 | 2.40E-08 | 7.79E-05 | 35.171 |
| Cheese intake | rs10896050 | T | G | 0.193 | -0.018 | 0.003 | 7.20E-11 | 1.06E-04 | 48.012 |
| Cheese intake | rs67238148 | T | G | 0.217 | 0.017 | 0.003 | 1.10E-09 | 9.31E-05 | 42.058 |
| Cheese intake | rs7936836 | A | C | 0.418 | 0.016 | 0.002 | 2.60E-12 | 1.23E-04 | 55.545 |
| Cheese intake | rs73024305 | C | G | 0.055 | 0.033 | 0.005 | 4.00E-11 | 1.10E-04 | 49.511 |
| Cheese intake | rs12786959 | T | A | 0.196 | -0.016 | 0.003 | 1.20E-08 | 8.15E-05 | 36.793 |
| Cheese intake | rs524468 | G | A | 0.261 | -0.014 | 0.003 | 2.40E-08 | 7.82E-05 | 35.297 |
| Cheese intake | rs1024853 | G | C | 0.438 | -0.013 | 0.002 | 1.30E-08 | 8.15E-05 | 36.780 |
| Cheese intake | rs7298331 | C | A | 0.605 | -0.013 | 0.002 | 1.10E-08 | 8.30E-05 | 37.475 |
| Cheese intake | rs12296440 | A | G | 0.170 | 0.019 | 0.003 | 2.80E-10 | 9.95E-05 | 44.941 |
| Cheese intake | rs61953351 | T | G | 0.250 | 0.015 | 0.003 | 1.50E-08 | 7.99E-05 | 36.094 |
| Cheese intake | rs1073242 | A | G | 0.554 | 0.016 | 0.002 | 6.70E-12 | 1.22E-04 | 55.201 |
| Cheese intake | rs11620149 | C | T | 0.143 | -0.018 | 0.003 | 3.60E-08 | 7.67E-05 | 34.633 |
| Cheese intake | rs17115145 | T | C | 0.401 | -0.013 | 0.002 | 1.80E-08 | 7.97E-05 | 35.996 |
| Cheese intake | rs35270670 | G | A | 0.218 | 0.016 | 0.003 | 1.50E-09 | 9.15E-05 | 41.297 |
| Cheese intake | rs4776970 | T | A | 0.358 | 0.015 | 0.002 | 3.50E-11 | 1.09E-04 | 49.279 |
| Cheese intake | rs12447542 | A | G | 0.126 | 0.020 | 0.003 | 6.80E-09 | 8.56E-05 | 38.670 |
| Cheese intake | rs61734410 | T | C | 0.255 | 0.017 | 0.003 | 2.20E-10 | 1.05E-04 | 47.609 |
| Cheese intake | rs62034322 | A | G | 0.380 | -0.014 | 0.002 | 1.40E-09 | 9.15E-05 | 41.311 |
| Cheese intake | rs71386942 | A | C | 0.269 | 0.014 | 0.003 | 9.90E-09 | 8.22E-05 | 37.101 |
| Cheese intake | rs11649653 | G | C | 0.382 | 0.014 | 0.002 | 1.50E-09 | 9.06E-05 | 40.889 |
| Cheese intake | rs919109 | C | G | 0.139 | 0.020 | 0.003 | 7.90E-10 | 9.50E-05 | 42.900 |
| Cheese intake | rs2854175 | A | C | 0.257 | 0.017 | 0.003 | 3.70E-11 | 1.10E-04 | 49.859 |
| Cheese intake | rs12951057 | G | C | 0.166 | -0.021 | 0.003 | 3.60E-12 | 1.24E-04 | 55.856 |
| Cheese intake | rs2960578 | G | T | 0.496 | 0.017 | 0.002 | 2.60E-14 | 1.45E-04 | 65.462 |
| Cheese intake | rs1434511 | T | C | 0.455 | 0.013 | 0.002 | 9.50E-09 | 8.34E-05 | 37.643 |
| Cheese intake | rs1291145 | C | T | 0.686 | -0.020 | 0.002 | 4.40E-17 | 1.77E-04 | 79.789 |
| Cheese intake | rs6126641 | A | G | 0.336 | 0.013 | 0.002 | 3.30E-08 | 7.81E-05 | 35.253 |
| Cheese intake | rs62236533 | A | G | 0.109 | 0.025 | 0.004 | 1.10E-11 | 1.19E-04 | 53.686 |
| Cereal intake | rs10857964 | C | T | 0.205 | 0.014 | 0.002 | 1.70E-10 | 6.47E-05 | 28.579 |
| Cereal intake | rs12354267 | C | T | 0.309 | 0.012 | 0.002 | 1.70E-09 | 5.79E-05 | 25.593 |
| Cereal intake | rs112780312 | A | G | 0.275 | -0.012 | 0.002 | 1.80E-09 | 5.88E-05 | 25.989 |
| Cereal intake | rs184643 | A | G | 0.567 | -0.012 | 0.002 | 1.60E-11 | 7.27E-05 | 32.100 |
| Cereal intake | rs6545770 | T | A | 0.748 | -0.014 | 0.002 | 2.70E-11 | 7.10E-05 | 31.371 |
| Cereal intake | rs4988235 | A | G | 0.737 | 0.011 | 0.002 | 1.50E-08 | 5.04E-05 | 22.257 |
| Cereal intake | rs67723420 | A | T | 0.376 | 0.011 | 0.002 | 1.20E-08 | 5.20E-05 | 22.967 |
| Cereal intake | rs7619139 | A | T | 0.589 | -0.017 | 0.002 | 9.70E-21 | 1.39E-04 | 61.469 |
| Cereal intake | rs9846396 | T | C | 0.442 | 0.012 | 0.002 | 3.00E-11 | 7.06E-05 | 31.196 |
| Cereal intake | rs11097340 | T | C | 0.400 | -0.012 | 0.002 | 2.10E-10 | 6.38E-05 | 28.190 |
| Cereal intake | rs3115230 | A | C | 0.752 | -0.011 | 0.002 | 3.00E-08 | 4.91E-05 | 21.703 |
| Cereal intake | rs11940694 | G | A | 0.604 | -0.013 | 0.002 | 5.00E-12 | 7.67E-05 | 33.886 |
| Cereal intake | rs10057775 | C | T | 0.894 | 0.020 | 0.003 | 4.50E-12 | 7.63E-05 | 33.704 |
| Cereal intake | rs79642906 | A | G | 0.083 | -0.018 | 0.003 | 1.90E-08 | 5.04E-05 | 22.243 |
| Cereal intake | rs1853931 | A | G | 0.531 | -0.011 | 0.002 | 3.80E-10 | 6.40E-05 | 28.271 |
| Cereal intake | rs6918737 | A | T | 0.234 | 0.014 | 0.002 | 7.60E-11 | 6.77E-05 | 29.902 |
| Cereal intake | rs2817377 | A | G | 0.538 | 0.010 | 0.002 | 3.10E-08 | 4.87E-05 | 21.523 |
| Cereal intake | rs2504706 | C | T | 0.235 | 0.018 | 0.002 | 5.30E-18 | 1.19E-04 | 52.435 |
| Cereal intake | rs9374896 | T | C | 0.466 | 0.018 | 0.002 | 1.30E-22 | 1.53E-04 | 67.532 |
| Cereal intake | rs4410790 | C | T | 0.631 | -0.011 | 0.002 | 3.40E-09 | 5.55E-05 | 24.509 |
| Cereal intake | rs62442924 | T | C | 0.194 | 0.013 | 0.002 | 1.70E-08 | 5.07E-05 | 22.410 |
| Cereal intake | rs13234131 | G | A | 0.128 | 0.017 | 0.003 | 1.60E-10 | 6.48E-05 | 28.603 |
| Cereal intake | rs9987289 | G | A | 0.909 | 0.018 | 0.003 | 7.80E-09 | 5.29E-05 | 23.381 |
| Cereal intake | rs4739095 | A | G | 0.766 | -0.013 | 0.002 | 9.90E-10 | 5.94E-05 | 26.228 |
| Cereal intake | rs2927238 | G | T | 0.613 | 0.010 | 0.002 | 2.10E-08 | 4.98E-05 | 21.997 |
| Cereal intake | rs2799849 | T | C | 0.678 | -0.012 | 0.002 | 9.80E-11 | 6.64E-05 | 29.307 |
| Cereal intake | rs7040561 | A | T | 0.851 | -0.016 | 0.003 | 1.10E-10 | 6.73E-05 | 29.704 |
| Cereal intake | rs491711 | C | A | 0.312 | 0.012 | 0.002 | 1.50E-09 | 5.87E-05 | 25.904 |
| Cereal intake | rs2450126 | G | A | 0.157 | -0.015 | 0.002 | 1.30E-09 | 5.87E-05 | 25.927 |
| Cereal intake | rs10837531 | G | C | 0.455 | 0.011 | 0.002 | 2.00E-09 | 5.77E-05 | 25.472 |
| Cereal intake | rs11038810 | G | A | 0.644 | 0.011 | 0.002 | 2.30E-09 | 5.68E-05 | 25.103 |
| Cereal intake | rs627185 | G | C | 0.544 | -0.011 | 0.002 | 1.50E-09 | 5.81E-05 | 25.682 |
| Cereal intake | rs2472297 | T | C | 0.261 | -0.016 | 0.002 | 4.50E-15 | 9.70E-05 | 42.862 |
| Cereal intake | rs1104608 | C | G | 0.426 | 0.011 | 0.002 | 2.30E-09 | 5.77E-05 | 25.492 |
| Cereal intake | rs68136852 | A | C | 0.152 | -0.014 | 0.002 | 1.20E-08 | 5.15E-05 | 22.755 |
| Cereal intake | rs3859193 | A | T | 0.470 | -0.010 | 0.002 | 9.50E-09 | 5.31E-05 | 23.461 |
| Cereal intake | rs8097544 | G | A | 0.145 | -0.025 | 0.003 | 3.20E-22 | 1.51E-04 | 66.554 |
| Cereal intake | rs4797242 | A | C | 0.297 | 0.011 | 0.002 | 4.50E-09 | 5.46E-05 | 24.110 |
| Cereal intake | rs11670024 | G | A | 0.116 | 0.016 | 0.003 | 1.10E-08 | 5.24E-05 | 23.136 |
| Cereal intake | rs6510177 | C | T | 0.806 | -0.013 | 0.002 | 1.20E-08 | 5.32E-05 | 23.507 |
| Cereal intake | rs78854891 | C | T | 0.066 | 0.022 | 0.004 | 1.10E-09 | 6.02E-05 | 26.592 |
| Cereal intake | rs56131196 | A | G | 0.189 | 0.018 | 0.002 | 2.70E-15 | 9.92E-05 | 43.832 |
| Cereal intake | rs838133 | G | A | 0.549 | -0.021 | 0.002 | 3.90E-29 | 2.11E-04 | 93.284 |
| Dried fruit intake | rs261809 | G | A | 0.541 | -0.010 | 0.002 | 9.80E-09 | 4.60E-05 | 19.422 |
| Dried fruit intake | rs11586016 | C | G | 0.371 | 0.010 | 0.002 | 1.10E-08 | 4.55E-05 | 19.209 |
| Dried fruit intake | rs12137234 | T | C | 0.304 | 0.010 | 0.002 | 2.80E-08 | 4.41E-05 | 18.580 |
| Dried fruit intake | rs72720396 | G | A | 0.229 | 0.011 | 0.002 | 8.70E-09 | 4.61E-05 | 19.456 |
| Dried fruit intake | rs11811826 | A | T | 0.224 | 0.013 | 0.002 | 4.40E-11 | 6.08E-05 | 25.637 |
| Dried fruit intake | rs3101339 | C | A | 0.603 | 0.014 | 0.002 | 6.20E-17 | 9.73E-05 | 41.053 |
| Dried fruit intake | rs75641275 | C | A | 0.143 | -0.014 | 0.002 | 2.90E-09 | 4.93E-05 | 20.777 |
| Dried fruit intake | rs7582086 | T | G | 0.468 | -0.010 | 0.002 | 8.80E-09 | 4.62E-05 | 19.471 |
| Dried fruit intake | rs7599488 | T | C | 0.426 | -0.010 | 0.002 | 6.70E-10 | 5.31E-05 | 22.382 |
| Dried fruit intake | rs4149513 | A | G | 0.494 | 0.012 | 0.002 | 2.20E-12 | 6.88E-05 | 29.013 |
| Dried fruit intake | rs17184707 | T | C | 0.213 | -0.011 | 0.002 | 2.10E-08 | 4.38E-05 | 18.488 |
| Dried fruit intake | rs4269101 | G | T | 0.719 | -0.014 | 0.002 | 1.10E-13 | 7.71E-05 | 32.508 |
| Dried fruit intake | rs11720884 | G | A | 0.250 | 0.011 | 0.002 | 7.60E-09 | 4.69E-05 | 19.776 |
| Dried fruit intake | rs57499472 | C | T | 0.404 | 0.010 | 0.002 | 8.10E-09 | 4.73E-05 | 19.959 |
| Dried fruit intake | rs10026792 | A | G | 0.290 | 0.011 | 0.002 | 3.90E-09 | 4.85E-05 | 20.451 |
| Dried fruit intake | rs1648404 | T | C | 0.476 | 0.009 | 0.002 | 1.80E-08 | 4.42E-05 | 18.655 |
| Dried fruit intake | rs746868 | G | C | 0.615 | -0.013 | 0.002 | 5.20E-14 | 7.89E-05 | 33.278 |
| Dried fruit intake | rs9385269 | T | C | 0.525 | 0.012 | 0.002 | 7.20E-13 | 7.26E-05 | 30.637 |
| Dried fruit intake | rs2328887 | C | T | 0.899 | 0.019 | 0.003 | 8.80E-12 | 6.49E-05 | 27.388 |
| Dried fruit intake | rs2533273 | A | C | 0.485 | -0.010 | 0.002 | 3.90E-09 | 4.87E-05 | 20.552 |
| Dried fruit intake | rs7808471 | C | T | 0.322 | -0.012 | 0.002 | 1.10E-10 | 5.81E-05 | 24.515 |
| Dried fruit intake | rs11772627 | C | G | 0.182 | 0.018 | 0.002 | 3.00E-17 | 1.00E-04 | 42.219 |
| Dried fruit intake | rs7829800 | G | A | 0.671 | -0.010 | 0.002 | 5.10E-09 | 4.82E-05 | 20.320 |
| Dried fruit intake | rs10740991 | C | G | 0.718 | 0.017 | 0.002 | 2.00E-19 | 1.14E-04 | 47.901 |
| Dried fruit intake | rs7916868 | T | A | 0.503 | 0.010 | 0.002 | 9.10E-09 | 4.61E-05 | 19.465 |
| Dried fruit intake | rs893856 | A | G | 0.149 | -0.013 | 0.002 | 1.30E-08 | 4.53E-05 | 19.093 |
| Dried fruit intake | rs10896126 | G | A | 0.304 | -0.015 | 0.002 | 1.60E-16 | 9.53E-05 | 40.178 |
| Dried fruit intake | rs11037497 | C | G | 0.446 | 0.010 | 0.002 | 5.70E-10 | 5.39E-05 | 22.718 |
| Dried fruit intake | rs1622515 | G | A | 0.485 | 0.010 | 0.002 | 2.90E-09 | 4.91E-05 | 20.722 |
| Dried fruit intake | rs3764002 | T | C | 0.261 | 0.013 | 0.002 | 5.10E-12 | 6.65E-05 | 28.043 |
| Dried fruit intake | rs4140799 | A | G | 0.532 | 0.009 | 0.002 | 1.80E-08 | 4.45E-05 | 18.783 |
| Dried fruit intake | rs34162196 | T | C | 0.101 | -0.022 | 0.003 | 7.10E-16 | 9.08E-05 | 38.307 |
| Dried fruit intake | rs10129747 | G | A | 0.530 | 0.009 | 0.002 | 2.60E-08 | 4.36E-05 | 18.405 |
| Dried fruit intake | rs1797235 | C | G | 0.375 | -0.010 | 0.002 | 8.90E-09 | 4.70E-05 | 19.845 |
| Dried fruit intake | rs11632215 | C | A | 0.120 | -0.014 | 0.003 | 4.40E-08 | 4.23E-05 | 17.842 |
| Dried fruit intake | rs862227 | G | A | 0.458 | -0.009 | 0.002 | 4.30E-08 | 4.17E-05 | 17.590 |
| Dried fruit intake | rs1582322 | G | A | 0.605 | 0.010 | 0.002 | 6.80E-09 | 4.73E-05 | 19.935 |
| Dried fruit intake | rs62084586 | C | T | 0.166 | 0.013 | 0.002 | 3.20E-09 | 4.96E-05 | 20.926 |
| Dried fruit intake | rs8081370 | T | C | 0.910 | -0.017 | 0.003 | 1.40E-08 | 4.54E-05 | 19.146 |
| Dried fruit intake | rs4800488 | A | C | 0.490 | 0.012 | 0.002 | 7.70E-13 | 7.18E-05 | 30.274 |
| Dried fruit intake | rs17175518 | A | C | 0.233 | 0.011 | 0.002 | 5.90E-09 | 4.72E-05 | 19.911 |
| Dried fruit intake | rs11152349 | A | G | 0.303 | 0.010 | 0.002 | 4.90E-08 | 4.15E-05 | 17.499 |
| Dried fruit intake | rs429358 | C | T | 0.154 | 0.020 | 0.002 | 6.70E-18 | 1.04E-04 | 43.768 |
| Fresh fruit intake | rs34162196 | T | C | 0.101 | -0.018 | 0.002 | 4.00E-19 | 5.97E-05 | 26.639 |
| Fresh fruit intake | rs559734 | C | G | 0.712 | 0.008 | 0.001 | 1.10E-08 | 2.48E-05 | 11.053 |
| Fresh fruit intake | rs10271924 | T | C | 0.493 | -0.007 | 0.001 | 2.00E-08 | 2.48E-05 | 11.083 |
| Fresh fruit intake | rs12536253 | C | G | 0.249 | -0.008 | 0.001 | 8.30E-09 | 2.49E-05 | 11.117 |
| Fresh fruit intake | rs11248509 | T | A | 0.371 | 0.007 | 0.001 | 7.40E-09 | 2.51E-05 | 11.198 |
| Fresh fruit intake | rs7869969 | G | A | 0.331 | 0.008 | 0.001 | 5.70E-09 | 2.54E-05 | 11.320 |
| Fresh fruit intake | rs17049185 | T | G | 0.268 | 0.008 | 0.001 | 7.30E-09 | 2.54E-05 | 11.321 |
| Fresh fruit intake | rs11032362 | A | G | 0.091 | 0.012 | 0.002 | 5.30E-09 | 2.54E-05 | 11.342 |
| Fresh fruit intake | rs62051554 | A | G | 0.109 | 0.012 | 0.002 | 4.60E-09 | 2.61E-05 | 11.650 |
| Fresh fruit intake | rs1356292 | T | C | 0.808 | 0.009 | 0.002 | 3.50E-09 | 2.62E-05 | 11.682 |
| Fresh fruit intake | rs817223 | C | T | 0.481 | -0.007 | 0.001 | 2.80E-09 | 2.64E-05 | 11.779 |
| Fresh fruit intake | rs149449 | A | G | 0.489 | 0.007 | 0.001 | 2.40E-09 | 2.65E-05 | 11.853 |
| Fresh fruit intake | rs12885598 | A | G | 0.597 | 0.008 | 0.001 | 1.70E-09 | 2.72E-05 | 12.138 |
| Fresh fruit intake | rs1866823 | A | G | 0.544 | 0.007 | 0.001 | 2.10E-09 | 2.74E-05 | 12.229 |
| Fresh fruit intake | rs7982441 | C | T | 0.732 | -0.008 | 0.001 | 9.80E-10 | 2.78E-05 | 12.401 |
| Fresh fruit intake | rs10840126 | G | A | 0.376 | -0.008 | 0.001 | 1.90E-09 | 2.80E-05 | 12.480 |
| Fresh fruit intake | rs1051547 | C | T | 0.562 | -0.008 | 0.001 | 1.10E-09 | 2.83E-05 | 12.616 |
| Fresh fruit intake | rs11085749 | A | G | 0.387 | -0.008 | 0.001 | 7.10E-10 | 2.84E-05 | 12.670 |
| Fresh fruit intake | rs10064431 | C | T | 0.522 | -0.008 | 0.001 | 6.00E-10 | 2.86E-05 | 12.780 |
| Fresh fruit intake | rs1375566 | A | G | 0.627 | -0.008 | 0.001 | 6.10E-10 | 2.87E-05 | 12.810 |
| Fresh fruit intake | rs10192394 | T | C | 0.529 | -0.008 | 0.001 | 4.50E-10 | 2.92E-05 | 13.058 |
| Fresh fruit intake | rs60452247 | A | G | 0.363 | 0.008 | 0.001 | 3.40E-10 | 2.94E-05 | 13.116 |
| Fresh fruit intake | rs12044599 | G | A | 0.210 | 0.009 | 0.002 | 3.70E-10 | 2.95E-05 | 13.151 |
| Fresh fruit intake | rs7554485 | C | T | 0.612 | -0.008 | 0.001 | 1.70E-10 | 3.05E-05 | 13.611 |
| Fresh fruit intake | rs12641371 | T | C | 0.433 | 0.008 | 0.001 | 1.40E-10 | 3.07E-05 | 13.727 |
| Fresh fruit intake | rs4953150 | T | C | 0.344 | -0.008 | 0.001 | 6.60E-11 | 3.21E-05 | 14.353 |
| Fresh fruit intake | rs11896330 | A | G | 0.633 | -0.008 | 0.001 | 3.40E-11 | 3.31E-05 | 14.799 |
| Fresh fruit intake | rs2790688 | T | C | 0.154 | 0.011 | 0.002 | 1.50E-11 | 3.42E-05 | 15.249 |
| Fresh fruit intake | rs2143081 | A | G | 0.540 | 0.008 | 0.001 | 1.30E-11 | 3.44E-05 | 15.358 |
| Fresh fruit intake | rs139042899 | C | A | 0.013 | 0.036 | 0.006 | 3.20E-09 | 3.44E-05 | 15.373 |
| Fresh fruit intake | rs2302593 | G | C | 0.487 | 0.009 | 0.001 | 3.10E-12 | 3.63E-05 | 16.224 |
| Fresh fruit intake | rs10838724 | T | G | 0.368 | 0.009 | 0.001 | 2.10E-12 | 3.78E-05 | 16.863 |
| Fresh fruit intake | rs739320 | C | T | 0.606 | -0.009 | 0.001 | 1.90E-12 | 3.87E-05 | 17.263 |
| Fresh fruit intake | rs13072255 | C | A | 0.494 | 0.009 | 0.001 | 2.10E-13 | 4.03E-05 | 18.000 |
| Fresh fruit intake | rs73455661 | G | A | 0.279 | 0.010 | 0.001 | 4.10E-14 | 4.27E-05 | 19.048 |
| Fresh fruit intake | rs28479795 | T | C | 0.221 | 0.011 | 0.001 | 2.50E-14 | 4.35E-05 | 19.425 |
| Fresh fruit intake | rs2867113 | A | G | 0.131 | -0.014 | 0.002 | 1.50E-12 | 4.37E-05 | 19.522 |
| Fresh fruit intake | rs2048522 | T | A | 0.435 | 0.010 | 0.001 | 1.80E-14 | 4.49E-05 | 20.068 |
| Fresh fruit intake | rs862227 | G | A | 0.458 | -0.010 | 0.001 | 1.10E-16 | 5.11E-05 | 22.814 |
| Fresh fruit intake | rs10828266 | G | A | 0.716 | 0.012 | 0.001 | 8.10E-20 | 6.22E-05 | 27.781 |
| Fresh fruit intake | rs994270 | G | C | 0.235 | 0.013 | 0.001 | 4.20E-20 | 6.32E-05 | 28.231 |
| Fresh fruit intake | rs1620977 | G | A | 0.731 | -0.013 | 0.001 | 1.10E-21 | 6.83E-05 | 30.511 |
| Fresh fruit intake | rs10249294 | A | G | 0.373 | 0.020 | 0.001 | 4.10E-54 | 1.79E-04 | 79.938 |
| Cooked vegetable intake | rs2252508 | G | A | 0.480 | 0.009 | 0.002 | 5.70E-09 | 4.14E-05 | 18.557 |
| Cooked vegetable intake | rs2102738 | C | A | 0.172 | -0.012 | 0.002 | 5.30E-09 | 4.22E-05 | 18.925 |
| Cooked vegetable intake | rs4851029 | G | T | 0.527 | 0.010 | 0.002 | 7.80E-11 | 5.16E-05 | 23.155 |
| Cooked vegetable intake | rs12629972 | C | T | 0.588 | 0.012 | 0.002 | 1.20E-13 | 6.74E-05 | 30.260 |
| Cooked vegetable intake | rs28450747 | A | G | 0.233 | -0.010 | 0.002 | 4.30E-08 | 3.69E-05 | 16.534 |
| Cooked vegetable intake | rs1816263 | C | T | 0.280 | 0.010 | 0.002 | 3.70E-08 | 3.70E-05 | 16.614 |
| Cooked vegetable intake | rs2844672 | A | G | 0.624 | -0.010 | 0.002 | 2.10E-09 | 4.36E-05 | 19.571 |
| Cooked vegetable intake | rs12550717 | A | G | 0.372 | 0.009 | 0.002 | 1.40E-08 | 3.95E-05 | 17.703 |
| Cooked vegetable intake | rs10156602 | G | A | 0.361 | 0.011 | 0.002 | 1.80E-11 | 5.59E-05 | 25.087 |
| Cooked vegetable intake | rs11138705 | C | G | 0.757 | 0.010 | 0.002 | 1.40E-08 | 3.96E-05 | 17.746 |
| Cooked vegetable intake | rs2052063 | T | C | 0.516 | -0.009 | 0.002 | 1.60E-09 | 4.47E-05 | 20.045 |
| Cooked vegetable intake | rs349062 | C | G | 0.393 | -0.009 | 0.002 | 2.50E-08 | 3.78E-05 | 16.967 |
| Cooked vegetable intake | rs28711392 | C | T | 0.367 | -0.011 | 0.002 | 4.60E-11 | 5.37E-05 | 24.077 |
| Cooked vegetable intake | rs10161952 | C | A | 0.313 | -0.010 | 0.002 | 1.30E-08 | 3.95E-05 | 17.700 |
| Cooked vegetable intake | rs1421085 | C | T | 0.403 | 0.010 | 0.002 | 8.30E-11 | 5.14E-05 | 23.053 |
| Cooked vegetable intake | rs838133 | G | A | 0.550 | 0.012 | 0.002 | 4.50E-13 | 6.76E-05 | 30.344 |
| Cooked vegetable intake | rs34155012 | T | C | 0.227 | 0.011 | 0.002 | 3.90E-08 | 3.92E-05 | 17.574 |
| Salad/raw vegetable intake | rs9427220 | T | A | 0.555 | -0.008 | 0.001 | 2.80E-08 | 3.17E-05 | 13.795 |
| Salad/raw vegetable intake | rs4083969 | G | C | 0.057 | 0.017 | 0.003 | 3.80E-08 | 3.17E-05 | 13.790 |
| Salad/raw vegetable intake | rs7619139 | A | T | 0.589 | 0.012 | 0.001 | 8.00E-18 | 7.54E-05 | 32.814 |
| Salad/raw vegetable intake | rs13102393 | G | C | 0.499 | 0.008 | 0.001 | 2.40E-08 | 3.18E-05 | 13.862 |
| Salad/raw vegetable intake | rs17460017 | T | A | 0.190 | 0.011 | 0.002 | 7.20E-10 | 3.85E-05 | 16.744 |
| Salad/raw vegetable intake | rs2194027 | A | T | 0.485 | -0.009 | 0.001 | 2.00E-09 | 3.70E-05 | 16.118 |
| Salad/raw vegetable intake | rs3129962 | A | G | 0.129 | -0.013 | 0.002 | 3.70E-10 | 3.98E-05 | 17.337 |
| Salad/raw vegetable intake | rs12203592 | T | C | 0.219 | -0.010 | 0.002 | 1.30E-09 | 3.62E-05 | 15.778 |
| Salad/raw vegetable intake | rs3095337 | C | G | 0.204 | -0.013 | 0.002 | 9.00E-13 | 5.17E-05 | 22.522 |
| Salad/raw vegetable intake | rs75248709 | T | C | 0.046 | -0.020 | 0.004 | 2.20E-08 | 3.41E-05 | 14.867 |
| Salad/raw vegetable intake | rs57221424 | G | C | 0.322 | 0.009 | 0.002 | 5.50E-09 | 3.49E-05 | 15.193 |
| Salad/raw vegetable intake | rs62461186 | C | A | 0.180 | -0.011 | 0.002 | 1.00E-09 | 3.80E-05 | 16.529 |
| Salad/raw vegetable intake | rs790561 | G | A | 0.704 | 0.012 | 0.002 | 1.40E-15 | 6.48E-05 | 28.232 |
| Salad/raw vegetable intake | rs7821179 | C | G | 0.847 | -0.011 | 0.002 | 4.40E-08 | 3.04E-05 | 13.239 |
| Salad/raw vegetable intake | rs10819082 | A | G | 0.667 | -0.009 | 0.002 | 1.40E-09 | 3.73E-05 | 16.239 |
| Salad/raw vegetable intake | rs6482190 | G | A | 0.719 | 0.011 | 0.002 | 1.40E-12 | 5.13E-05 | 22.337 |
| Salad/raw vegetable intake | rs1890012 | G | T | 0.195 | -0.010 | 0.002 | 8.10E-09 | 3.41E-05 | 14.844 |
| Salad/raw vegetable intake | rs12908495 | A | C | 0.243 | -0.009 | 0.002 | 2.00E-08 | 3.21E-05 | 13.987 |
| Salad/raw vegetable intake | rs1052352 | T | C | 0.524 | 0.008 | 0.001 | 1.00E-08 | 3.33E-05 | 14.481 |
| Salad/raw vegetable intake | rs34186148 | C | G | 0.370 | -0.008 | 0.001 | 4.80E-08 | 3.02E-05 | 13.161 |
| Salad/raw vegetable intake | rs4291983 | A | C | 0.518 | -0.008 | 0.001 | 3.70E-09 | 3.52E-05 | 15.343 |
| Salad/raw vegetable intake | rs8130508 | A | G | 0.290 | 0.009 | 0.002 | 3.00E-08 | 3.14E-05 | 13.690 |
| Body mass index | rs72634826 | A | G | 0.260 | -0.021 | 0.002 | 2.10E-19 | 1.65E-04 | 74.930 |
| Body mass index | rs4648450 | A | C | 0.467 | -0.015 | 0.002 | 1.30E-13 | 1.09E-04 | 49.812 |
| Body mass index | rs10799778 | G | T | 0.834 | -0.019 | 0.003 | 3.60E-12 | 9.54E-05 | 43.401 |
| Body mass index | rs12140153 | T | G | 0.094 | -0.034 | 0.003 | 5.70E-22 | 1.92E-04 | 87.542 |
| Body mass index | rs116377258 | G | A | 0.026 | 0.067 | 0.006 | 1.60E-26 | 2.25E-04 | 102.291 |
| Body mass index | rs61813324 | T | C | 0.136 | 0.029 | 0.003 | 6.20E-23 | 1.97E-04 | 89.834 |
| Body mass index | rs539515 | C | A | 0.205 | 0.050 | 0.002 | 7.00E-91 | 8.06E-04 | 367.105 |
| Body mass index | rs2076603 | A | G | 0.644 | -0.012 | 0.002 | 2.80E-09 | 6.95E-05 | 31.609 |
| Body mass index | rs909001 | G | C | 0.172 | 0.016 | 0.003 | 7.60E-10 | 7.48E-05 | 34.030 |
| Body mass index | rs2568958 | A | G | 0.604 | 0.022 | 0.002 | 1.50E-28 | 2.42E-04 | 109.898 |
| Body mass index | rs1890660 | A | G | 0.725 | -0.012 | 0.002 | 4.00E-08 | 5.94E-05 | 27.015 |
| Body mass index | rs2678204 | G | T | 0.340 | 0.024 | 0.002 | 9.00E-31 | 2.63E-04 | 119.466 |
| Body mass index | rs11122450 | G | T | 0.612 | -0.012 | 0.002 | 1.40E-08 | 6.35E-05 | 28.894 |
| Body mass index | rs2791643 | T | C | 0.762 | -0.013 | 0.002 | 2.80E-08 | 6.07E-05 | 27.635 |
| Body mass index | rs6669341 | G | A | 0.583 | -0.017 | 0.002 | 9.20E-18 | 1.45E-04 | 66.107 |
| Body mass index | rs3845344 | T | C | 0.391 | 0.017 | 0.002 | 7.40E-17 | 1.37E-04 | 62.405 |
| Body mass index | rs11165643 | T | C | 0.590 | 0.020 | 0.002 | 7.90E-23 | 1.91E-04 | 86.704 |
| Body mass index | rs1778830 | A | G | 0.362 | 0.014 | 0.002 | 8.80E-12 | 9.23E-05 | 41.994 |
| Body mass index | rs815163 | C | T | 0.563 | -0.017 | 0.002 | 4.60E-17 | 1.39E-04 | 63.167 |
| Body mass index | rs4658403 | T | C | 0.834 | -0.019 | 0.003 | 2.70E-12 | 9.66E-05 | 43.958 |
| Body mass index | rs12072739 | G | A | 0.225 | 0.016 | 0.002 | 1.20E-11 | 9.11E-05 | 41.466 |
| Body mass index | rs7549358 | C | G | 0.644 | -0.011 | 0.002 | 3.30E-08 | 6.02E-05 | 27.367 |
| Body mass index | rs61828088 | A | G | 0.110 | 0.022 | 0.003 | 2.90E-12 | 9.62E-05 | 43.763 |
| Body mass index | rs76702514 | G | C | 0.211 | -0.017 | 0.002 | 1.10E-11 | 9.23E-05 | 41.969 |
| Body mass index | rs7539903 | A | T | 0.615 | -0.014 | 0.002 | 1.20E-11 | 9.09E-05 | 41.351 |
| Body mass index | rs10927006 | C | T | 0.144 | -0.017 | 0.003 | 1.60E-09 | 7.19E-05 | 32.731 |
| Body mass index | rs3866805 | A | C | 0.356 | 0.012 | 0.002 | 1.20E-08 | 6.44E-05 | 29.278 |
| Body mass index | rs6682438 | C | T | 0.673 | 0.013 | 0.002 | 1.80E-09 | 7.12E-05 | 32.405 |
| Body mass index | rs113603865 | T | C | 0.212 | 0.019 | 0.002 | 2.50E-14 | 1.16E-04 | 52.804 |
| Body mass index | rs1167311 | A | G | 0.681 | -0.019 | 0.002 | 1.60E-19 | 1.63E-04 | 74.327 |
| Body mass index | rs7519259 | A | G | 0.528 | 0.014 | 0.002 | 5.80E-12 | 9.44E-05 | 42.931 |
| Body mass index | rs34517439 | A | C | 0.122 | 0.038 | 0.003 | 1.30E-35 | 3.13E-04 | 142.577 |
| Body mass index | rs6688826 | C | T | 0.298 | 0.014 | 0.002 | 7.70E-11 | 8.33E-05 | 37.880 |
| Body mass index | rs12089815 | A | G | 0.549 | -0.013 | 0.002 | 1.90E-10 | 8.04E-05 | 36.570 |
| Body mass index | rs12033257 | G | A | 0.382 | -0.016 | 0.002 | 5.10E-14 | 1.14E-04 | 51.639 |
| Body mass index | rs75035127 | G | A | 0.030 | -0.041 | 0.006 | 1.20E-12 | 9.96E-05 | 45.332 |
| Body mass index | rs7516554 | T | C | 0.400 | 0.012 | 0.002 | 1.50E-09 | 7.22E-05 | 32.847 |
| Body mass index | rs62107261 | C | T | 0.048 | -0.092 | 0.005 | 1.70E-86 | 7.70E-04 | 350.746 |
| Body mass index | rs935166 | A | G | 0.507 | -0.016 | 0.002 | 5.90E-16 | 1.29E-04 | 58.830 |
| Body mass index | rs6713781 | C | G | 0.402 | -0.013 | 0.002 | 1.40E-10 | 8.27E-05 | 37.611 |
| Body mass index | rs4671328 | G | T | 0.551 | -0.021 | 0.002 | 1.80E-25 | 2.18E-04 | 99.096 |
| Body mass index | rs4672338 | T | C | 0.336 | 0.013 | 0.002 | 2.40E-10 | 7.91E-05 | 35.998 |
| Body mass index | rs6752979 | A | G | 0.317 | 0.012 | 0.002 | 1.10E-08 | 6.42E-05 | 29.194 |
| Body mass index | rs10182416 | G | A | 0.512 | 0.013 | 0.002 | 1.80E-11 | 8.90E-05 | 40.478 |
| Body mass index | rs1446585 | G | A | 0.245 | -0.014 | 0.002 | 9.70E-10 | 7.08E-05 | 32.219 |
| Body mass index | rs62176993 | A | G | 0.401 | 0.011 | 0.002 | 3.40E-08 | 6.06E-05 | 27.553 |
| Body mass index | rs62190049 | C | G | 0.390 | -0.011 | 0.002 | 3.70E-08 | 6.07E-05 | 27.604 |
| Body mass index | rs1064213 | A | G | 0.478 | 0.015 | 0.002 | 6.60E-14 | 1.11E-04 | 50.336 |
| Body mass index | rs4482463 | A | C | 0.923 | -0.031 | 0.004 | 1.40E-16 | 1.35E-04 | 61.610 |
| Body mass index | rs6710091 | G | C | 0.348 | -0.012 | 0.002 | 2.70E-08 | 6.08E-05 | 27.652 |
| Body mass index | rs7571496 | G | A | 0.261 | -0.015 | 0.002 | 1.10E-11 | 9.19E-05 | 41.804 |
| Body mass index | rs10169594 | C | T | 0.363 | 0.012 | 0.002 | 1.80E-08 | 6.28E-05 | 28.553 |
| Body mass index | rs6707827 | G | A | 0.704 | 0.012 | 0.002 | 1.70E-08 | 6.35E-05 | 28.876 |
| Body mass index | rs11691869 | A | C | 0.362 | -0.020 | 0.002 | 1.90E-21 | 1.79E-04 | 81.568 |
| Body mass index | rs2381404 | C | T | 0.244 | 0.014 | 0.002 | 5.50E-09 | 6.73E-05 | 30.630 |
| Body mass index | rs6430068 | A | G | 0.109 | 0.019 | 0.003 | 5.60E-09 | 6.79E-05 | 30.873 |
| Body mass index | rs429343 | G | A | 0.577 | -0.017 | 0.002 | 8.40E-18 | 1.46E-04 | 66.440 |
| Body mass index | rs62176243 | T | A | 0.245 | -0.015 | 0.002 | 6.20E-11 | 8.41E-05 | 38.257 |
| Body mass index | rs56133507 | G | T | 0.197 | 0.014 | 0.002 | 1.40E-08 | 6.34E-05 | 28.824 |
| Body mass index | rs34234296 | A | G | 0.392 | -0.015 | 0.002 | 2.80E-13 | 1.07E-04 | 48.862 |
| Body mass index | rs55658481 | A | G | 0.339 | 0.013 | 0.002 | 1.80E-10 | 8.00E-05 | 36.391 |
| Body mass index | rs6744646 | G | A | 0.828 | 0.055 | 0.003 | 1.70E-98 | 8.74E-04 | 397.877 |
| Body mass index | rs59086897 | A | T | 0.488 | 0.033 | 0.002 | 6.60E-64 | 5.61E-04 | 255.159 |
| Body mass index | rs2861685 | C | T | 0.412 | -0.017 | 0.002 | 4.10E-17 | 1.39E-04 | 63.152 |
| Body mass index | rs12692596 | T | C | 0.372 | 0.014 | 0.002 | 1.90E-11 | 8.88E-05 | 40.379 |
| Body mass index | rs2216931 | A | C | 0.662 | 0.017 | 0.002 | 2.40E-15 | 1.24E-04 | 56.422 |
| Body mass index | rs13427822 | G | A | 0.271 | -0.018 | 0.002 | 4.70E-16 | 1.33E-04 | 60.477 |
| Body mass index | rs13012070 | A | G | 0.228 | -0.014 | 0.002 | 1.80E-09 | 7.13E-05 | 32.428 |
| Body mass index | rs35809007 | A | G | 0.363 | -0.017 | 0.002 | 4.10E-17 | 1.40E-04 | 63.882 |
| Body mass index | rs6545714 | A | G | 0.601 | -0.021 | 0.002 | 2.50E-24 | 2.05E-04 | 93.214 |
| Body mass index | rs4832298 | T | C | 0.686 | -0.016 | 0.002 | 8.20E-14 | 1.10E-04 | 49.970 |
| Body mass index | rs11675464 | G | A | 0.563 | 0.012 | 0.002 | 2.80E-09 | 6.96E-05 | 31.651 |
| Body mass index | rs10172070 | T | C | 0.146 | 0.016 | 0.003 | 9.60E-09 | 6.47E-05 | 29.449 |
| Body mass index | rs16846140 | G | A | 0.338 | 0.014 | 0.002 | 6.10E-11 | 8.47E-05 | 38.528 |
| Body mass index | rs13420048 | A | C | 0.365 | -0.016 | 0.002 | 3.10E-14 | 1.14E-04 | 52.031 |
| Body mass index | rs6705567 | C | T | 0.376 | -0.014 | 0.002 | 2.40E-12 | 9.82E-05 | 44.666 |
| Body mass index | rs745249 | T | C | 0.282 | 0.018 | 0.002 | 3.30E-16 | 1.32E-04 | 60.133 |
| Body mass index | rs72617140 | C | A | 0.214 | 0.019 | 0.002 | 1.10E-14 | 1.18E-04 | 53.570 |
| Body mass index | rs2433733 | A | G | 0.678 | -0.018 | 0.002 | 1.00E-16 | 1.36E-04 | 61.849 |
| Body mass index | rs4625852 | A | G | 0.205 | -0.014 | 0.002 | 2.00E-08 | 6.23E-05 | 28.336 |
| Body mass index | rs62246311 | A | G | 0.102 | 0.021 | 0.003 | 2.90E-10 | 7.85E-05 | 35.718 |
| Body mass index | rs13076052 | G | C | 0.275 | 0.014 | 0.002 | 3.10E-10 | 7.93E-05 | 36.078 |
| Body mass index | rs56038322 | A | G | 0.311 | 0.014 | 0.002 | 6.80E-11 | 8.54E-05 | 38.871 |
| Body mass index | rs11708540 | A | G | 0.157 | 0.016 | 0.003 | 4.70E-09 | 6.86E-05 | 31.225 |
| Body mass index | rs11914525 | G | A | 0.354 | -0.018 | 0.002 | 3.70E-18 | 1.49E-04 | 67.924 |
| Body mass index | rs1436348 | G | A | 0.583 | 0.016 | 0.002 | 6.50E-15 | 1.20E-04 | 54.651 |
| Body mass index | rs76183894 | C | T | 0.081 | -0.022 | 0.004 | 2.70E-09 | 7.08E-05 | 32.215 |
| Body mass index | rs13317303 | A | C | 0.150 | -0.016 | 0.003 | 1.00E-08 | 6.51E-05 | 29.593 |
| Body mass index | rs4017425 | T | C | 0.470 | -0.013 | 0.002 | 1.60E-10 | 8.10E-05 | 36.867 |
| Body mass index | rs9843653 | C | T | 0.512 | 0.029 | 0.002 | 7.70E-49 | 4.27E-04 | 194.258 |
| Body mass index | rs73052033 | C | T | 0.185 | -0.030 | 0.003 | 1.00E-31 | 2.72E-04 | 123.923 |
| Body mass index | rs2920503 | T | C | 0.285 | -0.014 | 0.002 | 1.40E-10 | 8.22E-05 | 37.385 |
| Body mass index | rs9860326 | G | C | 0.328 | 0.015 | 0.002 | 7.50E-12 | 9.29E-05 | 42.245 |
| Body mass index | rs6419869 | G | T | 0.886 | 0.022 | 0.003 | 2.00E-12 | 9.76E-05 | 44.413 |
| Body mass index | rs11709402 | G | A | 0.279 | 0.023 | 0.002 | 9.50E-25 | 2.10E-04 | 95.535 |
| Body mass index | rs1471740 | C | T | 0.740 | 0.019 | 0.002 | 9.90E-18 | 1.46E-04 | 66.315 |
| Body mass index | rs2035936 | T | G | 0.056 | 0.036 | 0.004 | 9.40E-17 | 1.41E-04 | 63.933 |
| Body mass index | rs529200 | G | A | 0.528 | 0.017 | 0.002 | 5.80E-18 | 1.48E-04 | 67.190 |
| Body mass index | rs11919665 | T | A | 0.680 | -0.013 | 0.002 | 1.10E-09 | 7.34E-05 | 33.391 |
| Body mass index | rs6777784 | T | G | 0.617 | 0.012 | 0.002 | 1.30E-09 | 7.25E-05 | 32.981 |
| Body mass index | rs75557510 | G | A | 0.061 | -0.031 | 0.004 | 1.60E-13 | 1.10E-04 | 50.207 |
| Body mass index | rs1454687 | G | C | 0.515 | -0.021 | 0.002 | 5.50E-26 | 2.20E-04 | 99.872 |
| Body mass index | rs1471093 | A | G | 0.617 | 0.013 | 0.002 | 5.40E-11 | 8.59E-05 | 39.095 |
| Body mass index | rs2569993 | C | T | 0.320 | 0.013 | 0.002 | 1.20E-09 | 7.36E-05 | 33.483 |
| Body mass index | rs62241847 | G | A | 0.315 | -0.012 | 0.002 | 5.80E-09 | 6.72E-05 | 30.576 |
| Body mass index | rs7619139 | A | T | 0.589 | 0.013 | 0.002 | 1.30E-10 | 8.21E-05 | 37.365 |
| Body mass index | rs754635 | G | C | 0.887 | 0.023 | 0.003 | 5.20E-13 | 1.03E-04 | 46.804 |
| Body mass index | rs9852062 | A | T | 0.557 | -0.014 | 0.002 | 3.60E-12 | 9.62E-05 | 43.784 |
| Body mass index | rs17639546 | A | G | 0.148 | -0.023 | 0.003 | 6.70E-17 | 1.38E-04 | 62.813 |
| Body mass index | rs9835772 | T | A | 0.244 | 0.017 | 0.002 | 6.80E-13 | 1.02E-04 | 46.394 |
| Body mass index | rs3851998 | G | C | 0.743 | -0.014 | 0.002 | 1.20E-09 | 7.37E-05 | 33.547 |
| Body mass index | rs355777 | C | G | 0.408 | 0.016 | 0.002 | 2.10E-14 | 1.16E-04 | 52.819 |
| Body mass index | rs6444950 | A | G | 0.238 | 0.016 | 0.002 | 3.90E-12 | 9.53E-05 | 43.368 |
| Body mass index | rs2606228 | C | A | 0.646 | -0.013 | 0.002 | 1.50E-10 | 8.28E-05 | 37.647 |
| Body mass index | rs6774894 | A | T | 0.358 | 0.013 | 0.002 | 4.30E-10 | 7.70E-05 | 35.016 |
| Body mass index | rs34811474 | A | G | 0.231 | -0.028 | 0.002 | 3.30E-33 | 2.85E-04 | 129.738 |
| Body mass index | rs4261944 | G | T | 0.365 | 0.014 | 0.002 | 7.90E-12 | 9.31E-05 | 42.359 |
| Body mass index | rs6531639 | A | G | 0.248 | -0.014 | 0.002 | 3.00E-09 | 7.29E-05 | 33.147 |
| Body mass index | rs2164300 | T | C | 0.519 | -0.012 | 0.002 | 8.60E-10 | 7.49E-05 | 34.070 |
| Body mass index | rs113079574 | T | C | 0.193 | -0.016 | 0.003 | 2.20E-10 | 8.06E-05 | 36.646 |
| Body mass index | rs4148155 | G | A | 0.113 | -0.022 | 0.003 | 7.80E-13 | 1.01E-04 | 45.956 |
| Body mass index | rs17289010 | G | A | 0.328 | -0.014 | 0.002 | 1.70E-10 | 8.07E-05 | 36.704 |
| Body mass index | rs2051559 | C | T | 0.133 | 0.021 | 0.003 | 2.70E-12 | 9.73E-05 | 44.242 |
| Body mass index | rs10938398 | A | G | 0.433 | 0.029 | 0.002 | 4.00E-48 | 4.22E-04 | 192.170 |
| Body mass index | rs2192158 | G | A | 0.553 | -0.015 | 0.002 | 1.40E-14 | 1.17E-04 | 53.116 |
| Body mass index | rs1346841 | A | G | 0.405 | -0.013 | 0.002 | 2.90E-10 | 7.91E-05 | 35.971 |
| Body mass index | rs4419475 | T | A | 0.407 | 0.012 | 0.002 | 4.90E-09 | 6.78E-05 | 30.860 |
| Body mass index | rs1229984 | C | T | 0.973 | 0.039 | 0.006 | 1.10E-10 | 8.03E-05 | 36.512 |
| Body mass index | rs13107325 | T | C | 0.075 | 0.047 | 0.004 | 5.40E-36 | 3.11E-04 | 141.617 |
| Body mass index | rs1296328 | C | A | 0.559 | -0.018 | 0.002 | 2.10E-19 | 1.62E-04 | 73.851 |
| Body mass index | rs73213484 | T | A | 0.141 | -0.023 | 0.003 | 1.00E-15 | 1.28E-04 | 58.071 |
| Body mass index | rs2102278 | G | A | 0.323 | 0.012 | 0.002 | 2.70E-08 | 6.12E-05 | 27.841 |
| Body mass index | rs11099020 | T | C | 0.641 | -0.014 | 0.002 | 4.90E-12 | 9.49E-05 | 43.168 |
| Body mass index | rs66679256 | T | C | 0.446 | 0.015 | 0.002 | 1.90E-14 | 1.16E-04 | 52.829 |
| Body mass index | rs7442137 | T | C | 0.634 | -0.012 | 0.002 | 1.60E-09 | 7.22E-05 | 32.866 |
| Body mass index | rs9991259 | A | G | 0.633 | 0.011 | 0.002 | 4.40E-08 | 5.94E-05 | 27.026 |
| Body mass index | rs6843852 | T | C | 0.508 | 0.013 | 0.002 | 1.70E-11 | 8.98E-05 | 40.840 |
| Body mass index | rs55920177 | T | A | 0.129 | -0.018 | 0.003 | 8.70E-10 | 7.46E-05 | 33.959 |
| Body mass index | rs698147 | G | A | 0.543 | -0.013 | 0.002 | 2.90E-11 | 8.78E-05 | 39.926 |
| Body mass index | rs111689389 | C | G | 0.283 | -0.014 | 0.002 | 7.40E-10 | 7.52E-05 | 34.204 |
| Body mass index | rs1919243 | C | T | 0.488 | 0.012 | 0.002 | 7.10E-09 | 6.81E-05 | 30.988 |
| Body mass index | rs1582931 | A | G | 0.473 | -0.013 | 0.002 | 1.60E-10 | 8.24E-05 | 37.466 |
| Body mass index | rs1438945 | A | T | 0.715 | -0.014 | 0.002 | 4.90E-10 | 7.74E-05 | 35.190 |
| Body mass index | rs13176429 | C | T | 0.688 | 0.015 | 0.002 | 6.10E-12 | 9.37E-05 | 42.606 |
| Body mass index | rs116374395 | A | G | 0.035 | 0.032 | 0.005 | 3.60E-09 | 6.95E-05 | 31.605 |
| Body mass index | rs396755 | G | C | 0.571 | -0.012 | 0.002 | 6.90E-10 | 7.56E-05 | 34.399 |
| Body mass index | rs10063055 | T | C | 0.253 | 0.013 | 0.002 | 1.40E-08 | 6.37E-05 | 28.971 |
| Body mass index | rs1503526 | C | T | 0.480 | 0.016 | 0.002 | 5.30E-15 | 1.21E-04 | 55.087 |
| Body mass index | rs1459190 | A | G | 0.519 | -0.014 | 0.002 | 6.30E-13 | 1.04E-04 | 47.519 |
| Body mass index | rs252761 | T | G | 0.588 | -0.011 | 0.002 | 2.70E-08 | 6.18E-05 | 28.126 |
| Body mass index | rs2962334 | T | G | 0.020 | 0.043 | 0.007 | 8.40E-10 | 7.44E-05 | 33.840 |
| Body mass index | rs1477290 | C | T | 0.137 | 0.034 | 0.003 | 4.20E-31 | 2.71E-04 | 123.342 |
| Body mass index | rs159037 | C | T | 0.254 | 0.013 | 0.002 | 2.60E-08 | 6.13E-05 | 27.888 |
| Body mass index | rs347551 | G | C | 0.472 | 0.014 | 0.002 | 8.40E-12 | 9.54E-05 | 43.415 |
| Body mass index | rs11134679 | G | A | 0.685 | 0.019 | 0.002 | 5.50E-18 | 1.49E-04 | 67.733 |
| Body mass index | rs2307111 | C | T | 0.395 | -0.028 | 0.002 | 1.80E-42 | 3.70E-04 | 168.364 |
| Body mass index | rs40071 | C | T | 0.179 | -0.026 | 0.003 | 1.30E-23 | 2.00E-04 | 90.915 |
| Body mass index | rs17056301 | C | T | 0.256 | 0.013 | 0.002 | 8.00E-09 | 6.63E-05 | 30.180 |
| Body mass index | rs7707394 | A | G | 0.357 | -0.019 | 0.002 | 1.10E-20 | 1.72E-04 | 78.127 |
| Body mass index | rs28404639 | T | C | 0.366 | -0.012 | 0.002 | 5.80E-09 | 6.74E-05 | 30.670 |
| Body mass index | rs7442885 | G | C | 0.214 | -0.023 | 0.002 | 5.20E-21 | 1.76E-04 | 79.978 |
| Body mass index | rs62379271 | G | T | 0.578 | 0.012 | 0.002 | 7.30E-09 | 6.66E-05 | 30.304 |
| Body mass index | rs7704382 | G | C | 0.433 | 0.012 | 0.002 | 1.30E-09 | 7.30E-05 | 33.225 |
| Body mass index | rs329118 | T | C | 0.419 | -0.017 | 0.002 | 1.00E-16 | 1.37E-04 | 62.229 |
| Body mass index | rs2133561 | T | A | 0.611 | -0.014 | 0.002 | 5.90E-12 | 9.58E-05 | 43.582 |
| Body mass index | rs4958702 | C | T | 0.572 | -0.016 | 0.002 | 9.60E-15 | 1.19E-04 | 53.974 |
| Body mass index | rs1322842 | G | A | 0.609 | -0.013 | 0.002 | 1.10E-10 | 8.35E-05 | 37.968 |
| Body mass index | rs75499503 | T | C | 0.220 | -0.018 | 0.002 | 3.10E-13 | 1.08E-04 | 49.298 |
| Body mass index | rs9366863 | C | T | 0.672 | -0.028 | 0.002 | 2.60E-40 | 3.49E-04 | 158.786 |
| Body mass index | rs1266874 | G | A | 0.350 | 0.014 | 0.002 | 1.20E-11 | 9.10E-05 | 41.404 |
| Body mass index | rs6938973 | C | T | 0.602 | 0.018 | 0.002 | 1.90E-19 | 1.61E-04 | 73.446 |
| Body mass index | rs6922607 | G | A | 0.190 | 0.014 | 0.003 | 1.50E-08 | 6.33E-05 | 28.795 |
| Body mass index | rs6909685 | T | C | 0.327 | -0.015 | 0.002 | 6.20E-12 | 9.43E-05 | 42.920 |
| Body mass index | rs156201 | C | G | 0.753 | 0.013 | 0.002 | 2.50E-08 | 6.14E-05 | 27.924 |
| Body mass index | rs13218383 | G | C | 0.335 | -0.015 | 0.002 | 7.40E-13 | 1.02E-04 | 46.352 |
| Body mass index | rs7762794 | G | A | 0.286 | 0.015 | 0.002 | 2.00E-11 | 8.89E-05 | 40.460 |
| Body mass index | rs2178899 | T | A | 0.129 | -0.026 | 0.003 | 4.80E-18 | 1.48E-04 | 67.413 |
| Body mass index | rs2281819 | A | T | 0.230 | -0.016 | 0.002 | 3.60E-11 | 8.71E-05 | 39.602 |
| Body mass index | rs34045288 | T | C | 0.334 | 0.024 | 0.002 | 6.90E-29 | 2.46E-04 | 112.144 |
| Body mass index | rs9296389 | C | G | 0.411 | 0.012 | 0.002 | 1.10E-08 | 6.44E-05 | 29.295 |
| Body mass index | rs1327259 | G | A | 0.388 | -0.015 | 0.002 | 1.50E-13 | 1.09E-04 | 49.474 |
| Body mass index | rs57989773 | C | T | 0.245 | 0.013 | 0.002 | 1.60E-08 | 6.70E-05 | 30.473 |
| Body mass index | rs2253310 | G | C | 0.626 | 0.018 | 0.002 | 2.40E-18 | 1.51E-04 | 68.770 |
| Body mass index | rs2875762 | C | G | 0.243 | 0.015 | 0.002 | 9.60E-11 | 8.36E-05 | 38.045 |
| Body mass index | rs765874 | A | T | 0.489 | -0.012 | 0.002 | 3.60E-09 | 6.90E-05 | 31.385 |
| Body mass index | rs11757278 | C | T | 0.304 | -0.015 | 0.002 | 1.00E-11 | 9.17E-05 | 41.713 |
| Body mass index | rs9267671 | A | G | 0.061 | 0.026 | 0.004 | 5.30E-10 | 7.64E-05 | 34.753 |
| Body mass index | rs72892910 | T | G | 0.172 | 0.039 | 0.003 | 6.60E-49 | 4.29E-04 | 195.345 |
| Body mass index | rs72948836 | G | A | 0.059 | -0.024 | 0.004 | 7.00E-09 | 6.64E-05 | 30.208 |
| Body mass index | rs9294260 | A | G | 0.477 | 0.015 | 0.002 | 2.00E-13 | 1.08E-04 | 49.136 |
| Body mass index | rs4895799 | T | C | 0.585 | 0.012 | 0.002 | 1.10E-08 | 6.50E-05 | 29.582 |
| Body mass index | rs2781668 | T | C | 0.166 | 0.015 | 0.003 | 3.20E-08 | 6.07E-05 | 27.594 |
| Body mass index | rs72910629 | G | A | 0.136 | 0.016 | 0.003 | 2.50E-08 | 6.22E-05 | 28.276 |
| Body mass index | rs9478496 | C | T | 0.164 | 0.018 | 0.003 | 6.60E-11 | 8.50E-05 | 38.668 |
| Body mass index | rs36007635 | A | G | 0.138 | -0.021 | 0.003 | 1.20E-12 | 1.00E-04 | 45.494 |
| Body mass index | rs3807566 | T | G | 0.438 | -0.013 | 0.002 | 4.30E-10 | 7.76E-05 | 35.301 |
| Body mass index | rs34025316 | T | C | 0.338 | 0.012 | 0.002 | 2.60E-08 | 6.21E-05 | 28.271 |
| Body mass index | rs2190887 | T | C | 0.561 | -0.011 | 0.002 | 4.10E-08 | 5.99E-05 | 27.227 |
| Body mass index | rs58862095 | T | C | 0.419 | -0.023 | 0.002 | 4.90E-29 | 2.49E-04 | 113.413 |
| Body mass index | rs17149254 | C | T | 0.805 | -0.021 | 0.003 | 3.90E-16 | 1.38E-04 | 62.953 |
| Body mass index | rs7805441 | T | C | 0.502 | 0.013 | 0.002 | 2.60E-11 | 8.93E-05 | 40.628 |
| Body mass index | rs3901286 | A | C | 0.152 | -0.023 | 0.003 | 3.70E-16 | 1.32E-04 | 60.170 |
| Body mass index | rs2396625 | A | T | 0.421 | -0.020 | 0.002 | 4.40E-22 | 1.87E-04 | 85.197 |
| Body mass index | rs11525873 | C | T | 0.098 | -0.024 | 0.003 | 8.50E-13 | 1.02E-04 | 46.364 |
| Body mass index | rs16868443 | C | G | 0.360 | 0.012 | 0.002 | 7.90E-09 | 6.61E-05 | 30.058 |
| Body mass index | rs2103123 | T | A | 0.472 | 0.011 | 0.002 | 4.30E-08 | 6.14E-05 | 27.949 |
| Body mass index | rs6950388 | A | G | 0.795 | 0.016 | 0.002 | 9.20E-11 | 8.32E-05 | 37.870 |
| Body mass index | rs17132130 | C | G | 0.222 | -0.018 | 0.002 | 2.70E-13 | 1.06E-04 | 48.417 |
| Body mass index | rs4722398 | T | C | 0.136 | 0.019 | 0.003 | 1.40E-10 | 8.13E-05 | 37.000 |
| Body mass index | rs9638713 | G | A | 0.975 | -0.036 | 0.006 | 2.40E-08 | 6.28E-05 | 28.568 |
| Body mass index | rs4307239 | G | A | 0.459 | 0.012 | 0.002 | 4.60E-10 | 7.73E-05 | 35.178 |
| Body mass index | rs213518 | C | T | 0.146 | 0.016 | 0.003 | 8.40E-09 | 6.59E-05 | 29.984 |
| Body mass index | rs215634 | G | A | 0.612 | -0.015 | 0.002 | 4.10E-14 | 1.14E-04 | 51.873 |
| Body mass index | rs4718964 | T | G | 0.413 | 0.011 | 0.002 | 2.60E-08 | 6.19E-05 | 28.145 |
| Body mass index | rs10950301 | A | G | 0.182 | -0.016 | 0.003 | 3.80E-10 | 7.77E-05 | 35.330 |
| Body mass index | rs6962980 | C | A | 0.556 | -0.016 | 0.002 | 1.60E-15 | 1.26E-04 | 57.256 |
| Body mass index | rs2289379 | T | C | 0.396 | -0.015 | 0.002 | 1.80E-13 | 1.08E-04 | 49.269 |
| Body mass index | rs1805123 | G | T | 0.245 | -0.016 | 0.002 | 1.30E-12 | 9.98E-05 | 45.403 |
| Body mass index | rs13248187 | C | T | 0.268 | 0.016 | 0.002 | 1.50E-12 | 1.00E-04 | 45.652 |
| Body mass index | rs4737188 | T | A | 0.474 | -0.013 | 0.002 | 7.40E-11 | 8.44E-05 | 38.394 |
| Body mass index | rs72673947 | G | A | 0.107 | 0.022 | 0.003 | 7.70E-12 | 9.39E-05 | 42.728 |
| Body mass index | rs56893062 | G | T | 0.303 | 0.013 | 0.002 | 2.40E-09 | 7.08E-05 | 32.220 |
| Body mass index | rs36061954 | T | C | 0.399 | 0.013 | 0.002 | 2.50E-10 | 7.95E-05 | 36.160 |
| Body mass index | rs35957544 | T | G | 0.574 | -0.020 | 0.002 | 1.90E-22 | 1.89E-04 | 86.177 |
| Body mass index | rs1609010 | G | A | 0.566 | 0.022 | 0.002 | 7.10E-27 | 2.29E-04 | 104.195 |
| Body mass index | rs1106761 | A | G | 0.384 | 0.015 | 0.002 | 1.70E-12 | 1.01E-04 | 46.101 |
| Body mass index | rs2616143 | A | G | 0.320 | -0.014 | 0.002 | 1.60E-10 | 8.17E-05 | 37.180 |
| Body mass index | rs12156160 | G | A | 0.150 | 0.016 | 0.003 | 1.60E-08 | 6.37E-05 | 28.962 |
| Body mass index | rs2922757 | T | A | 0.597 | 0.013 | 0.002 | 4.50E-10 | 7.74E-05 | 35.226 |
| Body mass index | rs4876611 | G | A | 0.720 | 0.019 | 0.002 | 3.50E-18 | 1.50E-04 | 68.347 |
| Body mass index | rs10099330 | G | A | 0.453 | 0.012 | 0.002 | 3.70E-09 | 6.91E-05 | 31.456 |
| Body mass index | rs11250094 | C | G | 0.548 | -0.021 | 0.002 | 4.10E-25 | 2.14E-04 | 97.296 |
| Body mass index | rs2725371 | G | A | 0.696 | -0.016 | 0.002 | 3.50E-13 | 1.06E-04 | 48.122 |
| Body mass index | rs12681792 | A | C | 0.193 | 0.015 | 0.003 | 4.40E-09 | 6.88E-05 | 31.316 |
| Body mass index | rs76387394 | G | A | 0.053 | 0.027 | 0.004 | 1.00E-09 | 7.42E-05 | 33.758 |
| Body mass index | rs2114210 | A | G | 0.336 | 0.014 | 0.002 | 7.10E-11 | 8.46E-05 | 38.485 |
| Body mass index | rs7828631 | T | C | 0.110 | 0.018 | 0.003 | 1.20E-08 | 6.47E-05 | 29.417 |
| Body mass index | rs7042372 | G | A | 0.335 | -0.013 | 0.002 | 2.70E-09 | 7.09E-05 | 32.232 |
| Body mass index | rs10756792 | T | C | 0.743 | -0.019 | 0.002 | 2.40E-17 | 1.44E-04 | 65.357 |
| Body mass index | rs16916303 | G | A | 0.120 | -0.020 | 0.003 | 3.10E-10 | 8.04E-05 | 36.559 |
| Body mass index | rs1360201 | T | C | 0.482 | 0.013 | 0.002 | 4.00E-11 | 8.65E-05 | 39.334 |
| Body mass index | rs2482356 | C | T | 0.429 | -0.011 | 0.002 | 2.80E-08 | 6.10E-05 | 27.754 |
| Body mass index | rs10760277 | T | C | 0.385 | 0.014 | 0.002 | 7.90E-12 | 9.34E-05 | 42.511 |
| Body mass index | rs7027304 | T | C | 0.653 | 0.015 | 0.002 | 1.70E-12 | 9.97E-05 | 45.356 |
| Body mass index | rs56203622 | C | T | 0.146 | 0.018 | 0.003 | 2.00E-10 | 8.02E-05 | 36.473 |
| Body mass index | rs12375985 | A | G | 0.355 | -0.015 | 0.002 | 1.50E-12 | 9.94E-05 | 45.226 |
| Body mass index | rs13292699 | C | A | 0.434 | -0.021 | 0.002 | 4.00E-26 | 2.23E-04 | 101.283 |
| Body mass index | rs12001437 | C | T | 0.368 | 0.012 | 0.002 | 2.20E-09 | 7.10E-05 | 32.300 |
| Body mass index | rs7852189 | G | A | 0.316 | 0.017 | 0.002 | 1.10E-15 | 1.27E-04 | 57.839 |
| Body mass index | rs1412239 | G | C | 0.323 | 0.025 | 0.002 | 6.40E-31 | 2.65E-04 | 120.375 |
| Body mass index | rs1999433 | T | C | 0.447 | -0.012 | 0.002 | 3.70E-09 | 6.93E-05 | 31.526 |
| Body mass index | rs7038943 | C | T | 0.339 | -0.014 | 0.002 | 1.50E-11 | 9.02E-05 | 41.045 |
| Body mass index | rs28670671 | C | T | 0.286 | -0.013 | 0.002 | 4.00E-08 | 6.41E-05 | 29.139 |
| Body mass index | rs1330199 | T | G | 0.483 | -0.012 | 0.002 | 2.80E-09 | 7.07E-05 | 32.154 |
| Body mass index | rs7034554 | G | A | 0.374 | -0.014 | 0.002 | 4.70E-11 | 8.58E-05 | 39.027 |
| Body mass index | rs7357754 | G | A | 0.500 | 0.014 | 0.002 | 5.80E-13 | 1.04E-04 | 47.155 |
| Body mass index | rs2398861 | G | A | 0.259 | 0.018 | 0.002 | 1.60E-15 | 1.27E-04 | 57.887 |
| Body mass index | rs7024334 | G | T | 0.779 | -0.014 | 0.002 | 1.70E-08 | 6.31E-05 | 28.699 |
| Body mass index | rs12762034 | C | T | 0.077 | 0.028 | 0.004 | 1.30E-13 | 1.09E-04 | 49.513 |
| Body mass index | rs10510025 | T | C | 0.247 | 0.018 | 0.002 | 5.00E-15 | 1.22E-04 | 55.620 |
| Body mass index | rs117118217 | C | G | 0.018 | 0.044 | 0.008 | 2.30E-08 | 6.85E-05 | 31.178 |
| Body mass index | rs2172131 | C | T | 0.579 | -0.015 | 0.002 | 6.70E-14 | 1.12E-04 | 50.779 |
| Body mass index | rs10903791 | A | G | 0.604 | 0.011 | 0.002 | 4.50E-08 | 5.97E-05 | 27.154 |
| Body mass index | rs11012732 | G | A | 0.332 | 0.021 | 0.002 | 3.30E-24 | 2.05E-04 | 93.115 |
| Body mass index | rs4919197 | T | C | 0.474 | 0.011 | 0.002 | 4.80E-08 | 6.05E-05 | 27.524 |
| Body mass index | rs7893571 | T | G | 0.666 | 0.014 | 0.002 | 9.40E-12 | 9.27E-05 | 42.149 |
| Body mass index | rs7070670 | T | C | 0.328 | -0.013 | 0.002 | 2.70E-09 | 7.12E-05 | 32.370 |
| Body mass index | rs7924036 | T | G | 0.503 | -0.014 | 0.002 | 3.50E-13 | 1.05E-04 | 47.753 |
| Body mass index | rs11000993 | C | T | 0.124 | 0.021 | 0.003 | 1.80E-12 | 9.83E-05 | 44.709 |
| Body mass index | rs577525 | C | T | 0.562 | 0.019 | 0.002 | 3.70E-22 | 1.86E-04 | 84.585 |
| Body mass index | rs7916385 | T | C | 0.150 | -0.024 | 0.003 | 1.80E-15 | 1.48E-04 | 67.519 |
| Body mass index | rs12259464 | A | G | 0.485 | 0.013 | 0.002 | 5.70E-11 | 8.59E-05 | 39.083 |
| Body mass index | rs147568678 | C | T | 0.238 | -0.014 | 0.002 | 1.40E-09 | 7.32E-05 | 33.310 |
| Body mass index | rs11017771 | C | G | 0.206 | -0.014 | 0.002 | 2.00E-08 | 6.28E-05 | 28.572 |
| Body mass index | rs73601548 | T | C | 0.115 | 0.017 | 0.003 | 4.90E-08 | 5.95E-05 | 27.079 |
| Body mass index | rs35972789 | A | C | 0.037 | -0.029 | 0.005 | 2.90E-08 | 6.09E-05 | 27.718 |
| Body mass index | rs17399739 | G | A | 0.069 | 0.028 | 0.004 | 2.00E-12 | 9.85E-05 | 44.825 |
| Body mass index | rs10887578 | C | G | 0.498 | 0.013 | 0.002 | 8.40E-11 | 8.47E-05 | 38.509 |
| Body mass index | rs79780963 | T | C | 0.077 | 0.024 | 0.004 | 2.50E-10 | 7.92E-05 | 36.050 |
| Body mass index | rs705145 | A | C | 0.345 | 0.014 | 0.002 | 1.60E-11 | 9.01E-05 | 40.990 |
| Body mass index | rs67609008 | C | T | 0.284 | 0.018 | 0.002 | 2.80E-15 | 1.25E-04 | 56.762 |
| Body mass index | rs4757144 | A | G | 0.590 | 0.016 | 0.002 | 1.50E-15 | 1.26E-04 | 57.454 |
| Body mass index | rs11024271 | C | T | 0.623 | 0.012 | 0.002 | 2.00E-08 | 6.25E-05 | 28.452 |
| Body mass index | rs6265 | T | C | 0.188 | -0.040 | 0.003 | 5.90E-56 | 4.92E-04 | 224.063 |
| Body mass index | rs594024 | C | T | 0.554 | -0.015 | 0.002 | 1.00E-13 | 1.10E-04 | 50.056 |
| Body mass index | rs7944782 | G | T | 0.510 | 0.016 | 0.002 | 1.40E-15 | 1.28E-04 | 58.082 |
| Body mass index | rs558887 | G | A | 0.308 | -0.013 | 0.002 | 8.00E-10 | 7.54E-05 | 34.283 |
| Body mass index | rs61903695 | G | A | 0.255 | 0.017 | 0.002 | 2.70E-13 | 1.06E-04 | 48.362 |
| Body mass index | rs1793636 | C | G | 0.309 | -0.013 | 0.002 | 6.00E-10 | 7.62E-05 | 34.643 |
| Body mass index | rs7952102 | C | T | 0.388 | -0.014 | 0.002 | 2.80E-12 | 9.66E-05 | 43.923 |
| Body mass index | rs4929923 | C | T | 0.645 | 0.019 | 0.002 | 3.40E-20 | 1.68E-04 | 76.342 |
| Body mass index | rs13642 | T | A | 0.361 | -0.016 | 0.002 | 1.80E-15 | 1.25E-04 | 56.979 |
| Body mass index | rs59227842 | G | A | 0.311 | 0.023 | 0.002 | 1.90E-26 | 2.28E-04 | 103.948 |
| Body mass index | rs55707359 | G | T | 0.015 | 0.053 | 0.008 | 1.10E-10 | 8.49E-05 | 38.632 |
| Body mass index | rs7124681 | A | C | 0.408 | 0.026 | 0.002 | 1.40E-37 | 3.24E-04 | 147.476 |
| Body mass index | rs2234458 | T | C | 0.640 | -0.021 | 0.002 | 1.10E-23 | 1.99E-04 | 90.659 |
| Body mass index | rs10160769 | C | G | 0.217 | -0.015 | 0.002 | 4.00E-10 | 7.93E-05 | 36.054 |
| Body mass index | rs2512892 | C | T | 0.566 | 0.013 | 0.002 | 5.50E-11 | 8.56E-05 | 38.939 |
| Body mass index | rs12364470 | G | T | 0.165 | 0.019 | 0.003 | 1.10E-12 | 1.00E-04 | 45.717 |
| Body mass index | rs10742752 | C | T | 0.612 | 0.012 | 0.002 | 1.00E-08 | 6.50E-05 | 29.585 |
| Body mass index | rs7947143 | A | G | 0.163 | -0.018 | 0.003 | 3.60E-11 | 8.70E-05 | 39.573 |
| Body mass index | rs1048932 | A | C | 0.413 | -0.015 | 0.002 | 2.10E-14 | 1.16E-04 | 52.771 |
| Body mass index | rs7925100 | A | G | 0.396 | 0.014 | 0.002 | 1.10E-12 | 1.00E-04 | 45.681 |
| Body mass index | rs349071 | A | G | 0.500 | -0.013 | 0.002 | 9.20E-11 | 8.37E-05 | 38.098 |
| Body mass index | rs329651 | T | G | 0.804 | 0.016 | 0.003 | 4.80E-10 | 7.76E-05 | 35.312 |
| Body mass index | rs55726687 | A | G | 0.210 | 0.025 | 0.002 | 2.70E-24 | 2.05E-04 | 93.184 |
| Body mass index | rs6560906 | C | T | 0.692 | -0.012 | 0.002 | 1.70E-08 | 6.30E-05 | 28.677 |
| Body mass index | rs1458156 | T | C | 0.488 | 0.014 | 0.002 | 3.10E-12 | 9.67E-05 | 43.987 |
| Body mass index | rs1126930 | C | G | 0.035 | 0.032 | 0.005 | 4.60E-09 | 6.81E-05 | 30.988 |
| Body mass index | rs3897102 | T | C | 0.411 | 0.012 | 0.002 | 1.90E-09 | 7.29E-05 | 33.165 |
| Body mass index | rs7132908 | A | G | 0.384 | 0.029 | 0.002 | 1.10E-46 | 4.09E-04 | 186.179 |
| Body mass index | rs4267103 | C | T | 0.186 | 0.016 | 0.003 | 5.40E-10 | 7.67E-05 | 34.909 |
| Body mass index | rs317656 | A | T | 0.725 | -0.014 | 0.002 | 1.20E-10 | 8.24E-05 | 37.497 |
| Body mass index | rs55966114 | T | C | 0.193 | 0.015 | 0.003 | 3.80E-09 | 6.93E-05 | 31.528 |
| Body mass index | rs4764949 | G | A | 0.326 | -0.018 | 0.002 | 3.10E-17 | 1.42E-04 | 64.536 |
| Body mass index | rs961498 | C | G | 0.503 | 0.012 | 0.002 | 2.20E-09 | 7.22E-05 | 32.835 |
| Body mass index | rs147730268 | T | G | 0.087 | -0.035 | 0.004 | 9.80E-22 | 1.91E-04 | 86.685 |
| Body mass index | rs10505836 | C | A | 0.860 | 0.018 | 0.003 | 7.60E-10 | 7.62E-05 | 34.687 |
| Body mass index | rs78086698 | C | T | 0.040 | 0.032 | 0.005 | 2.30E-10 | 8.04E-05 | 36.567 |
| Body mass index | rs723672 | T | C | 0.432 | 0.011 | 0.002 | 4.00E-08 | 6.05E-05 | 27.537 |
| Body mass index | rs2271189 | A | G | 0.403 | -0.016 | 0.002 | 2.60E-15 | 1.25E-04 | 56.680 |
| Body mass index | rs11115160 | A | G | 0.238 | -0.013 | 0.002 | 2.40E-08 | 6.25E-05 | 28.452 |
| Body mass index | rs12427047 | T | C | 0.243 | -0.017 | 0.002 | 1.90E-13 | 1.07E-04 | 48.860 |
| Body mass index | rs73193736 | G | A | 0.244 | -0.018 | 0.002 | 3.60E-15 | 1.25E-04 | 56.779 |
| Body mass index | rs11610621 | A | T | 0.148 | 0.016 | 0.003 | 5.00E-09 | 6.79E-05 | 30.868 |
| Body mass index | rs56858768 | A | G | 0.297 | 0.016 | 0.002 | 5.60E-13 | 1.04E-04 | 47.364 |
| Body mass index | rs11842871 | T | G | 0.260 | -0.015 | 0.002 | 2.00E-11 | 8.97E-05 | 40.787 |
| Body mass index | rs4477562 | T | C | 0.129 | 0.030 | 0.003 | 3.70E-23 | 1.99E-04 | 90.331 |
| Body mass index | rs9888533 | T | C | 0.538 | 0.012 | 0.002 | 2.20E-09 | 7.35E-05 | 33.450 |
| Body mass index | rs1183668 | G | C | 0.370 | -0.016 | 0.002 | 1.90E-14 | 1.18E-04 | 53.501 |
| Body mass index | rs1967772 | A | G | 0.285 | -0.017 | 0.002 | 3.10E-14 | 1.16E-04 | 52.723 |
| Body mass index | rs17446299 | G | C | 0.166 | 0.016 | 0.003 | 1.90E-09 | 7.18E-05 | 32.649 |
| Body mass index | rs1441264 | A | G | 0.594 | 0.018 | 0.002 | 3.70E-18 | 1.57E-04 | 71.264 |
| Body mass index | rs7996639 | A | G | 0.449 | 0.014 | 0.002 | 1.20E-12 | 1.02E-04 | 46.323 |
| Body mass index | rs56399737 | T | C | 0.449 | -0.016 | 0.002 | 3.60E-15 | 1.24E-04 | 56.391 |
| Body mass index | rs6561937 | A | T | 0.754 | -0.016 | 0.002 | 6.60E-12 | 9.43E-05 | 42.882 |
| Body mass index | rs4055791 | T | C | 0.417 | -0.018 | 0.002 | 4.40E-19 | 1.59E-04 | 72.165 |
| Body mass index | rs7331420 | A | G | 0.285 | -0.014 | 0.002 | 1.60E-10 | 8.19E-05 | 37.258 |
| Body mass index | rs9571687 | A | C | 0.329 | -0.013 | 0.002 | 4.60E-10 | 7.75E-05 | 35.273 |
| Body mass index | rs9522173 | T | A | 0.394 | -0.012 | 0.002 | 1.30E-09 | 7.31E-05 | 33.249 |
| Body mass index | rs8015400 | A | C | 0.677 | 0.021 | 0.002 | 9.60E-24 | 2.01E-04 | 91.326 |
| Body mass index | rs1451963 | T | G | 0.082 | 0.023 | 0.004 | 3.10E-10 | 7.89E-05 | 35.912 |
| Body mass index | rs1286058 | A | T | 0.704 | 0.015 | 0.002 | 1.70E-11 | 9.02E-05 | 41.055 |
| Body mass index | rs10144067 | T | C | 0.591 | 0.018 | 0.002 | 1.50E-19 | 1.65E-04 | 75.138 |
| Body mass index | rs12881629 | G | A | 0.083 | 0.022 | 0.004 | 8.30E-10 | 7.47E-05 | 33.999 |
| Body mass index | rs12889639 | A | G | 0.651 | 0.016 | 0.002 | 5.80E-14 | 1.12E-04 | 51.115 |
| Body mass index | rs12885458 | G | T | 0.508 | -0.016 | 0.002 | 1.80E-16 | 1.35E-04 | 61.504 |
| Body mass index | rs3902951 | G | T | 0.237 | 0.015 | 0.002 | 8.80E-10 | 7.64E-05 | 34.753 |
| Body mass index | rs1860750 | A | T | 0.511 | 0.012 | 0.002 | 4.00E-09 | 6.90E-05 | 31.386 |
| Body mass index | rs8020365 | A | T | 0.220 | 0.025 | 0.002 | 6.70E-25 | 2.13E-04 | 96.733 |
| Body mass index | rs6575340 | A | G | 0.636 | 0.021 | 0.002 | 9.10E-24 | 2.02E-04 | 91.829 |
| Body mass index | rs3803286 | G | A | 0.667 | -0.018 | 0.002 | 3.10E-18 | 1.51E-04 | 68.547 |
| Body mass index | rs113624107 | A | G | 0.226 | 0.014 | 0.002 | 1.50E-09 | 7.28E-05 | 33.112 |
| Body mass index | rs7159965 | G | C | 0.164 | 0.015 | 0.003 | 3.60E-08 | 6.06E-05 | 27.581 |
| Body mass index | rs217672 | C | A | 0.272 | 0.017 | 0.002 | 2.90E-14 | 1.16E-04 | 52.549 |
| Body mass index | rs61992671 | G | A | 0.492 | -0.016 | 0.002 | 1.20E-14 | 1.29E-04 | 58.792 |
| Body mass index | rs62007782 | A | G | 0.265 | -0.016 | 0.002 | 6.80E-13 | 1.03E-04 | 46.677 |
| Body mass index | rs28457680 | A | T | 0.137 | 0.018 | 0.003 | 6.60E-10 | 7.67E-05 | 34.879 |
| Body mass index | rs3784710 | C | T | 0.227 | -0.030 | 0.002 | 2.30E-35 | 3.05E-04 | 138.925 |
| Body mass index | rs35364449 | T | C | 0.110 | 0.022 | 0.003 | 6.70E-12 | 9.47E-05 | 43.077 |
| Body mass index | rs7169847 | T | G | 0.636 | -0.014 | 0.002 | 2.00E-11 | 9.01E-05 | 40.990 |
| Body mass index | rs4284600 | C | T | 0.467 | 0.012 | 0.002 | 1.30E-09 | 7.41E-05 | 33.706 |
| Body mass index | rs34153025 | C | T | 0.022 | -0.038 | 0.007 | 1.80E-08 | 6.41E-05 | 29.166 |
| Body mass index | rs79212998 | G | T | 0.069 | -0.023 | 0.004 | 2.80E-09 | 7.06E-05 | 32.118 |
| Body mass index | rs8024137 | T | A | 0.848 | 0.016 | 0.003 | 2.40E-08 | 6.23E-05 | 28.363 |
| Body mass index | rs9944241 | C | T | 0.484 | -0.015 | 0.002 | 2.50E-12 | 1.06E-04 | 48.184 |
| Body mass index | rs11639144 | A | G | 0.238 | -0.014 | 0.002 | 8.10E-09 | 6.66E-05 | 30.309 |
| Body mass index | rs140159717 | T | C | 0.082 | -0.025 | 0.004 | 3.90E-11 | 9.21E-05 | 41.906 |
| Body mass index | rs7498044 | A | G | 0.217 | -0.017 | 0.002 | 4.70E-12 | 9.71E-05 | 44.157 |
| Body mass index | rs355393 | G | A | 0.470 | -0.012 | 0.002 | 8.50E-09 | 6.61E-05 | 30.083 |
| Body mass index | rs11071646 | A | G | 0.022 | -0.040 | 0.007 | 4.80E-09 | 6.85E-05 | 31.141 |
| Body mass index | rs57488047 | C | T | 0.468 | -0.015 | 0.002 | 4.50E-14 | 1.15E-04 | 52.288 |
| Body mass index | rs56803094 | G | A | 0.227 | -0.015 | 0.002 | 5.00E-10 | 7.75E-05 | 35.249 |
| Body mass index | rs3211995 | A | G | 0.160 | -0.015 | 0.003 | 1.70E-08 | 6.36E-05 | 28.954 |
| Body mass index | rs4613074 | C | T | 0.185 | -0.024 | 0.003 | 3.70E-20 | 1.68E-04 | 76.344 |
| Body mass index | rs35154326 | G | A | 0.274 | -0.013 | 0.002 | 2.80E-09 | 7.12E-05 | 32.380 |
| Body mass index | rs56094641 | G | A | 0.405 | 0.073 | 0.002 | 1.00E-200 | 2.59E-03 | 1182.038 |
| Body mass index | rs9673839 | G | A | 0.491 | 0.013 | 0.002 | 8.80E-11 | 8.44E-05 | 38.380 |
| Body mass index | rs7201895 | A | G | 0.354 | -0.015 | 0.002 | 8.00E-13 | 1.03E-04 | 46.876 |
| Body mass index | rs249293 | C | G | 0.696 | 0.013 | 0.002 | 2.10E-09 | 7.19E-05 | 32.706 |
| Body mass index | rs5011579 | G | C | 0.715 | 0.014 | 0.002 | 1.30E-10 | 8.20E-05 | 37.323 |
| Body mass index | rs12149660 | A | G | 0.115 | -0.022 | 0.003 | 1.60E-12 | 1.00E-04 | 45.596 |
| Body mass index | rs7206608 | G | C | 0.322 | 0.013 | 0.002 | 2.60E-10 | 7.93E-05 | 36.094 |
| Body mass index | rs3814883 | T | C | 0.482 | 0.024 | 0.002 | 2.50E-33 | 2.89E-04 | 131.439 |
| Body mass index | rs2342892 | G | T | 0.516 | -0.013 | 0.002 | 1.70E-10 | 8.09E-05 | 36.797 |
| Body mass index | rs117342986 | T | C | 0.026 | 0.037 | 0.007 | 1.80E-08 | 6.91E-05 | 31.443 |
| Body mass index | rs879620 | T | C | 0.613 | 0.024 | 0.002 | 5.70E-32 | 2.76E-04 | 125.773 |
| Body mass index | rs7498665 | G | A | 0.400 | 0.027 | 0.002 | 9.30E-39 | 3.37E-04 | 153.344 |
| Body mass index | rs862320 | T | C | 0.410 | -0.023 | 0.002 | 2.20E-30 | 2.61E-04 | 118.827 |
| Body mass index | rs7774 | A | C | 0.310 | 0.015 | 0.002 | 1.50E-12 | 1.01E-04 | 45.840 |
| Body mass index | rs1017529 | A | C | 0.175 | 0.015 | 0.003 | 1.20E-08 | 6.70E-05 | 30.470 |
| Body mass index | rs2306593 | T | C | 0.488 | -0.017 | 0.002 | 1.70E-17 | 1.45E-04 | 65.821 |
| Body mass index | rs11079849 | T | C | 0.329 | -0.020 | 0.002 | 6.30E-22 | 1.85E-04 | 84.170 |
| Body mass index | rs7218014 | C | T | 0.197 | 0.019 | 0.003 | 7.80E-14 | 1.12E-04 | 50.726 |
| Body mass index | rs11150745 | G | A | 0.318 | -0.021 | 0.002 | 2.30E-22 | 1.89E-04 | 85.913 |
| Body mass index | rs4790292 | A | C | 0.154 | -0.026 | 0.003 | 6.90E-21 | 1.76E-04 | 80.185 |
| Body mass index | rs16966801 | G | A | 0.197 | 0.015 | 0.003 | 6.70E-09 | 6.69E-05 | 30.433 |
| Body mass index | rs62072006 | C | A | 0.145 | 0.016 | 0.003 | 2.30E-08 | 6.25E-05 | 28.433 |
| Body mass index | rs35867081 | G | A | 0.512 | -0.015 | 0.002 | 2.00E-13 | 1.08E-04 | 49.324 |
| Body mass index | rs9674487 | G | C | 0.001 | 0.160 | 0.029 | 2.90E-08 | 6.84E-05 | 31.108 |
| Body mass index | rs118136827 | T | G | 0.281 | -0.014 | 0.002 | 1.10E-09 | 7.39E-05 | 33.632 |
| Body mass index | rs8076669 | C | T | 0.562 | 0.014 | 0.002 | 5.90E-12 | 9.43E-05 | 42.921 |
| Body mass index | rs1320251 | T | C | 0.455 | -0.018 | 0.002 | 1.50E-18 | 1.55E-04 | 70.334 |
| Body mass index | rs59237168 | C | T | 0.216 | -0.016 | 0.002 | 8.20E-11 | 8.41E-05 | 38.275 |
| Body mass index | rs56161855 | T | A | 0.133 | 0.023 | 0.003 | 1.10E-14 | 1.19E-04 | 54.141 |
| Body mass index | rs1788808 | G | A | 0.495 | -0.021 | 0.002 | 8.80E-25 | 2.11E-04 | 95.906 |
| Body mass index | rs559231 | T | G | 0.393 | 0.013 | 0.002 | 3.20E-10 | 7.93E-05 | 36.092 |
| Body mass index | rs6567160 | C | T | 0.233 | 0.054 | 0.002 | 2.50E-114 | 1.03E-03 | 467.554 |
| Body mass index | rs7232171 | T | G | 0.583 | 0.013 | 0.002 | 1.20E-10 | 8.25E-05 | 37.553 |
| Body mass index | rs1834144 | A | C | 0.373 | -0.014 | 0.002 | 8.10E-12 | 9.35E-05 | 42.529 |
| Body mass index | rs512121 | C | T | 0.192 | -0.016 | 0.003 | 6.70E-10 | 7.63E-05 | 34.716 |
| Body mass index | rs60764613 | T | G | 0.145 | 0.020 | 0.003 | 1.60E-12 | 1.00E-04 | 45.684 |
| Body mass index | rs784257 | C | T | 0.813 | 0.018 | 0.003 | 2.40E-12 | 9.87E-05 | 44.900 |
| Body mass index | rs9951619 | G | T | 0.767 | 0.015 | 0.002 | 2.90E-10 | 8.01E-05 | 36.435 |
| Body mass index | rs57636386 | C | T | 0.084 | -0.041 | 0.004 | 1.00E-29 | 2.57E-04 | 116.735 |
| Body mass index | rs12956148 | A | C | 0.278 | 0.014 | 0.002 | 7.90E-10 | 7.53E-05 | 34.252 |
| Body mass index | rs10417386 | C | T | 0.696 | 0.012 | 0.002 | 1.30E-08 | 6.45E-05 | 29.353 |
| Body mass index | rs55714539 | C | A | 0.344 | 0.018 | 0.002 | 3.50E-17 | 1.44E-04 | 65.307 |
| Body mass index | rs8112818 | G | A | 0.400 | -0.020 | 0.002 | 2.30E-23 | 1.99E-04 | 90.322 |
| Body mass index | rs12459368 | G | A | 0.268 | -0.017 | 0.002 | 1.20E-14 | 1.18E-04 | 53.741 |
| Body mass index | rs12462975 | A | G | 0.330 | 0.019 | 0.002 | 8.50E-20 | 1.67E-04 | 76.115 |
| Body mass index | rs12977787 | A | G | 0.541 | 0.013 | 0.002 | 1.70E-11 | 9.01E-05 | 40.988 |
| Body mass index | rs56356382 | C | T | 0.192 | -0.023 | 0.003 | 6.70E-19 | 1.58E-04 | 71.986 |
| Body mass index | rs56352336 | C | T | 0.155 | -0.017 | 0.003 | 1.80E-09 | 7.28E-05 | 33.111 |
| Body mass index | rs7250833 | T | C | 0.289 | 0.014 | 0.002 | 7.10E-10 | 7.58E-05 | 34.502 |
| Body mass index | rs429358 | C | T | 0.154 | -0.027 | 0.003 | 5.90E-22 | 1.85E-04 | 84.102 |
| Body mass index | rs10423928 | A | T | 0.194 | -0.033 | 0.003 | 2.20E-40 | 3.51E-04 | 159.794 |
| Body mass index | rs3764625 | G | T | 0.588 | -0.011 | 0.002 | 1.50E-08 | 6.41E-05 | 29.149 |
| Body mass index | rs73026725 | A | C | 0.154 | -0.022 | 0.003 | 9.70E-16 | 1.29E-04 | 58.577 |
| Body mass index | rs7259070 | C | T | 0.596 | 0.022 | 0.002 | 3.80E-26 | 2.27E-04 | 103.197 |
| Body mass index | rs34481751 | A | C | 0.165 | -0.019 | 0.003 | 2.20E-12 | 1.01E-04 | 45.966 |
| Body mass index | rs2153740 | G | A | 0.480 | -0.011 | 0.002 | 1.20E-08 | 6.55E-05 | 29.780 |
| Body mass index | rs4456769 | T | C | 0.333 | 0.015 | 0.002 | 3.80E-12 | 9.61E-05 | 43.707 |
| Body mass index | rs2425816 | A | G | 0.415 | 0.012 | 0.002 | 7.90E-10 | 7.53E-05 | 34.273 |
| Body mass index | rs909892 | A | G | 0.135 | -0.018 | 0.003 | 8.60E-10 | 7.56E-05 | 34.372 |
| Body mass index | rs73142879 | T | C | 0.192 | -0.027 | 0.003 | 9.30E-27 | 2.30E-04 | 104.658 |
| Body mass index | rs55886426 | G | C | 0.053 | -0.028 | 0.005 | 1.90E-09 | 7.76E-05 | 35.279 |
| Body mass index | rs6134916 | T | C | 0.493 | -0.011 | 0.002 | 4.60E-08 | 5.98E-05 | 27.199 |
| Body mass index | rs1884897 | G | A | 0.627 | 0.020 | 0.002 | 1.70E-21 | 1.82E-04 | 82.841 |
| Body mass index | rs947088 | T | G | 0.718 | 0.013 | 0.002 | 9.80E-09 | 6.59E-05 | 29.967 |
| Body mass index | rs6023649 | G | A | 0.743 | -0.014 | 0.002 | 1.50E-09 | 7.50E-05 | 34.122 |
| Body mass index | rs8132491 | A | G | 0.313 | -0.016 | 0.002 | 2.10E-12 | 1.04E-04 | 47.199 |
| Body mass index | rs8134638 | C | T | 0.376 | 0.012 | 0.002 | 1.90E-09 | 7.22E-05 | 32.834 |
| Body mass index | rs17193211 | T | C | 0.067 | -0.025 | 0.004 | 1.10E-09 | 7.64E-05 | 34.772 |
| Body mass index | rs394608 | C | T | 0.538 | 0.019 | 0.002 | 2.30E-20 | 1.72E-04 | 78.085 |
| Body mass index | rs406388 | G | C | 0.177 | 0.016 | 0.003 | 1.40E-09 | 7.35E-05 | 33.457 |
| Body mass index | rs5995843 | G | A | 0.346 | -0.018 | 0.002 | 5.00E-17 | 1.40E-04 | 63.902 |
| Body mass index | rs28489620 | A | G | 0.290 | -0.015 | 0.002 | 7.70E-12 | 9.48E-05 | 43.137 |
| Waist circumference | rs3768321 | T | G | 0.197 | 0.018 | 0.002 | 3.80E-15 | 9.86E-05 | 45.589 |
| Waist circumference | rs6669341 | G | A | 0.583 | -0.013 | 0.002 | 4.50E-12 | 7.62E-05 | 35.242 |
| Waist circumference | rs11165493 | A | G | 0.343 | 0.011 | 0.002 | 1.30E-08 | 5.22E-05 | 24.108 |
| Waist circumference | rs588660 | A | G | 0.584 | 0.016 | 0.002 | 1.00E-17 | 1.17E-04 | 54.145 |
| Waist circumference | rs2618039 | T | A | 0.381 | 0.012 | 0.002 | 5.20E-11 | 6.88E-05 | 31.816 |
| Waist circumference | rs75035127 | G | A | 0.030 | -0.033 | 0.005 | 1.50E-10 | 6.55E-05 | 30.292 |
| Waist circumference | rs815163 | C | T | 0.563 | -0.013 | 0.002 | 2.70E-13 | 8.49E-05 | 39.254 |
| Waist circumference | rs2678204 | G | T | 0.340 | 0.016 | 0.002 | 7.90E-17 | 1.11E-04 | 51.193 |
| Waist circumference | rs7539903 | A | T | 0.616 | -0.011 | 0.002 | 5.30E-09 | 5.44E-05 | 25.130 |
| Waist circumference | rs4844809 | C | G | 0.132 | 0.016 | 0.003 | 1.90E-09 | 5.75E-05 | 26.562 |
| Waist circumference | rs7537581 | A | C | 0.532 | 0.011 | 0.002 | 2.60E-09 | 5.74E-05 | 26.530 |
| Waist circumference | rs4908672 | T | C | 0.393 | 0.011 | 0.002 | 4.70E-10 | 6.20E-05 | 28.642 |
| Waist circumference | rs41279738 | G | T | 0.026 | 0.052 | 0.006 | 1.70E-20 | 1.38E-04 | 63.931 |
| Waist circumference | rs115056380 | A | G | 0.048 | -0.024 | 0.004 | 1.30E-08 | 5.15E-05 | 23.819 |
| Waist circumference | rs35216639 | A | C | 0.111 | 0.017 | 0.003 | 3.30E-09 | 5.57E-05 | 25.744 |
| Waist circumference | rs539515 | C | A | 0.205 | 0.038 | 0.002 | 1.50E-65 | 4.66E-04 | 215.543 |
| Waist circumference | rs3866805 | A | C | 0.356 | 0.010 | 0.002 | 2.50E-08 | 4.98E-05 | 23.005 |
| Waist circumference | rs156902 | T | G | 0.267 | -0.014 | 0.002 | 1.40E-08 | 7.49E-05 | 34.599 |
| Waist circumference | rs12140153 | T | G | 0.094 | -0.027 | 0.003 | 2.10E-17 | 1.21E-04 | 55.799 |
| Waist circumference | rs7519259 | A | G | 0.528 | 0.013 | 0.002 | 1.70E-12 | 8.00E-05 | 36.980 |
| Waist circumference | rs2568958 | A | G | 0.604 | 0.017 | 0.002 | 1.60E-20 | 1.37E-04 | 63.324 |
| Waist circumference | rs34517439 | A | C | 0.122 | 0.031 | 0.003 | 1.90E-28 | 2.00E-04 | 92.282 |
| Waist circumference | rs945211 | C | G | 0.616 | 0.010 | 0.002 | 4.10E-08 | 4.81E-05 | 22.220 |
| Waist circumference | rs6682438 | C | T | 0.673 | 0.012 | 0.002 | 5.40E-11 | 6.85E-05 | 31.665 |
| Waist circumference | rs6693294 | G | A | 0.689 | -0.017 | 0.002 | 1.10E-18 | 1.24E-04 | 57.379 |
| Waist circumference | rs587271 | T | C | 0.687 | 0.012 | 0.002 | 4.10E-09 | 6.00E-05 | 27.709 |
| Waist circumference | rs11162968 | C | T | 0.316 | 0.012 | 0.002 | 2.10E-10 | 6.47E-05 | 29.886 |
| Waist circumference | rs12072739 | G | A | 0.224 | 0.016 | 0.002 | 3.90E-14 | 9.15E-05 | 42.285 |
| Waist circumference | rs61813324 | T | C | 0.136 | 0.022 | 0.003 | 9.50E-17 | 1.13E-04 | 52.367 |
| Waist circumference | rs1108548 | G | A | 0.277 | 0.012 | 0.002 | 1.20E-09 | 5.94E-05 | 27.441 |
| Waist circumference | rs72634826 | A | G | 0.260 | -0.015 | 0.002 | 5.40E-13 | 8.54E-05 | 39.450 |
| Waist circumference | rs649458 | A | T | 0.860 | -0.018 | 0.003 | 1.50E-12 | 7.95E-05 | 36.759 |
| Waist circumference | rs3845344 | T | C | 0.391 | 0.011 | 0.002 | 4.30E-09 | 5.49E-05 | 25.379 |
| Waist circumference | rs12042959 | G | A | 0.144 | -0.015 | 0.003 | 5.20E-09 | 5.49E-05 | 25.397 |
| Waist circumference | rs1405261 | A | T | 0.434 | -0.010 | 0.002 | 4.80E-08 | 4.77E-05 | 22.043 |
| Waist circumference | rs77165542 | T | C | 0.035 | -0.070 | 0.005 | 9.90E-47 | 3.36E-04 | 155.404 |
| Waist circumference | rs9308964 | T | C | 0.565 | -0.010 | 0.002 | 3.50E-08 | 4.88E-05 | 22.532 |
| Waist circumference | rs4851283 | G | C | 0.685 | -0.017 | 0.002 | 2.50E-19 | 1.31E-04 | 60.386 |
| Waist circumference | rs4482463 | A | C | 0.923 | -0.026 | 0.003 | 1.30E-14 | 9.53E-05 | 44.038 |
| Waist circumference | rs76286777 | C | T | 0.218 | 0.023 | 0.002 | 1.80E-27 | 1.88E-04 | 86.797 |
| Waist circumference | rs10185199 | A | G | 0.281 | -0.011 | 0.002 | 2.00E-08 | 5.30E-05 | 24.481 |
| Waist circumference | rs6739755 | G | A | 0.603 | -0.016 | 0.002 | 3.10E-18 | 1.21E-04 | 56.100 |
| Waist circumference | rs1441098 | T | A | 0.544 | -0.010 | 0.002 | 3.50E-08 | 4.87E-05 | 22.500 |
| Waist circumference | rs3087523 | A | G | 0.125 | 0.017 | 0.003 | 1.00E-09 | 6.01E-05 | 27.757 |
| Waist circumference | rs34234296 | A | G | 0.392 | -0.013 | 0.002 | 1.30E-12 | 8.21E-05 | 37.946 |
| Waist circumference | rs13427822 | G | A | 0.271 | -0.014 | 0.002 | 3.20E-12 | 7.91E-05 | 36.579 |
| Waist circumference | rs35882248 | T | C | 0.317 | 0.016 | 0.002 | 2.80E-16 | 1.07E-04 | 49.408 |
| Waist circumference | rs72618637 | A | T | 0.189 | -0.013 | 0.002 | 2.10E-08 | 5.17E-05 | 23.917 |
| Waist circumference | rs13420048 | A | C | 0.365 | -0.013 | 0.002 | 7.00E-13 | 8.26E-05 | 38.188 |
| Waist circumference | rs35681682 | C | T | 0.408 | -0.010 | 0.002 | 1.00E-08 | 4.98E-05 | 23.012 |
| Waist circumference | rs308911 | G | A | 0.714 | -0.011 | 0.002 | 6.90E-09 | 5.37E-05 | 24.832 |
| Waist circumference | rs429343 | G | A | 0.577 | -0.012 | 0.002 | 5.90E-12 | 7.57E-05 | 34.986 |
| Waist circumference | rs1609303 | A | T | 0.631 | 0.015 | 0.002 | 2.10E-16 | 1.08E-04 | 50.144 |
| Waist circumference | rs12478299 | C | T | 0.252 | -0.012 | 0.002 | 1.90E-08 | 5.06E-05 | 23.364 |
| Waist circumference | rs73985439 | C | A | 0.307 | 0.012 | 0.002 | 1.60E-10 | 6.55E-05 | 30.278 |
| Waist circumference | rs12463617 | C | A | 0.828 | 0.043 | 0.002 | 1.80E-74 | 5.31E-04 | 245.636 |
| Waist circumference | rs1731246 | T | G | 0.757 | -0.012 | 0.002 | 1.30E-08 | 5.15E-05 | 23.802 |
| Waist circumference | rs11898037 | C | T | 0.368 | 0.011 | 0.002 | 1.20E-08 | 5.19E-05 | 24.006 |
| Waist circumference | rs13410783 | G | A | 0.370 | 0.014 | 0.002 | 2.40E-14 | 9.27E-05 | 42.852 |
| Waist circumference | rs1861410 | T | C | 0.555 | -0.016 | 0.002 | 6.40E-19 | 1.27E-04 | 58.502 |
| Waist circumference | rs2861692 | C | T | 0.275 | -0.017 | 0.002 | 7.70E-17 | 1.11E-04 | 51.148 |
| Waist circumference | rs13033310 | A | G | 0.253 | 0.013 | 0.002 | 1.30E-09 | 5.95E-05 | 27.503 |
| Waist circumference | rs114964326 | A | G | 0.029 | -0.030 | 0.005 | 2.10E-08 | 5.10E-05 | 23.562 |
| Waist circumference | rs10803762 | A | G | 0.677 | 0.012 | 0.002 | 4.40E-10 | 6.24E-05 | 28.827 |
| Waist circumference | rs10184230 | T | C | 0.648 | -0.012 | 0.002 | 8.90E-11 | 6.70E-05 | 30.967 |
| Waist circumference | rs11675464 | G | A | 0.563 | 0.011 | 0.002 | 3.70E-10 | 6.25E-05 | 28.901 |
| Waist circumference | rs72617140 | C | A | 0.214 | 0.015 | 0.002 | 3.00E-12 | 7.78E-05 | 35.947 |
| Waist circumference | rs1799923 | G | A | 0.887 | 0.017 | 0.003 | 1.20E-09 | 5.89E-05 | 27.234 |
| Waist circumference | rs28350 | G | A | 0.821 | -0.014 | 0.002 | 2.50E-09 | 5.72E-05 | 26.428 |
| Waist circumference | rs9843653 | C | T | 0.512 | 0.020 | 0.002 | 6.70E-28 | 1.92E-04 | 88.554 |
| Waist circumference | rs557951 | G | T | 0.313 | 0.012 | 0.002 | 4.00E-10 | 6.27E-05 | 28.998 |
| Waist circumference | rs7630382 | T | C | 0.532 | 0.013 | 0.002 | 9.30E-14 | 8.89E-05 | 41.097 |
| Waist circumference | rs1454687 | G | C | 0.515 | -0.016 | 0.002 | 1.50E-19 | 1.30E-04 | 60.308 |
| Waist circumference | rs9814758 | G | T | 0.356 | -0.011 | 0.002 | 2.70E-09 | 5.71E-05 | 26.368 |
| Waist circumference | rs9289630 | C | G | 0.389 | 0.014 | 0.002 | 3.50E-15 | 9.96E-05 | 46.043 |
| Waist circumference | rs12107172 | G | A | 0.136 | 0.016 | 0.003 | 1.80E-09 | 5.80E-05 | 26.802 |
| Waist circumference | rs62246311 | A | G | 0.102 | 0.020 | 0.003 | 3.50E-12 | 7.72E-05 | 35.663 |
| Waist circumference | rs2881479 | T | A | 0.146 | 0.018 | 0.003 | 1.80E-12 | 7.95E-05 | 36.740 |
| Waist circumference | rs2470549 | C | T | 0.598 | -0.012 | 0.002 | 5.20E-11 | 6.87E-05 | 31.752 |
| Waist circumference | rs4017425 | T | C | 0.470 | -0.010 | 0.002 | 1.60E-08 | 5.11E-05 | 23.615 |
| Waist circumference | rs6799080 | A | G | 0.353 | 0.010 | 0.002 | 4.30E-08 | 4.78E-05 | 22.108 |
| Waist circumference | rs62261725 | G | A | 0.326 | -0.015 | 0.002 | 6.70E-15 | 9.72E-05 | 44.919 |
| Waist circumference | rs6551304 | G | A | 0.832 | 0.017 | 0.002 | 2.10E-12 | 7.92E-05 | 36.600 |
| Waist circumference | rs1078455 | C | T | 0.310 | 0.011 | 0.002 | 9.70E-09 | 5.34E-05 | 24.663 |
| Waist circumference | rs6791983 | A | C | 0.750 | 0.012 | 0.002 | 2.30E-09 | 5.69E-05 | 26.285 |
| Waist circumference | rs10490869 | T | A | 0.210 | 0.016 | 0.002 | 1.20E-13 | 8.84E-05 | 40.882 |
| Waist circumference | rs62243489 | G | T | 0.259 | -0.016 | 0.002 | 3.00E-14 | 9.30E-05 | 43.006 |
| Waist circumference | rs9835772 | T | A | 0.244 | 0.012 | 0.002 | 5.60E-09 | 5.42E-05 | 25.062 |
| Waist circumference | rs13322435 | G | A | 0.404 | -0.017 | 0.002 | 1.90E-20 | 1.39E-04 | 64.021 |
| Waist circumference | rs4856720 | C | G | 0.539 | 0.011 | 0.002 | 1.80E-10 | 6.50E-05 | 30.026 |
| Waist circumference | rs73052033 | C | T | 0.185 | -0.021 | 0.002 | 7.90E-20 | 1.33E-04 | 61.503 |
| Waist circumference | rs1436348 | G | A | 0.583 | 0.012 | 0.002 | 5.40E-12 | 7.59E-05 | 35.079 |
| Waist circumference | rs1711171 | C | T | 0.750 | 0.018 | 0.002 | 1.80E-17 | 1.16E-04 | 53.529 |
| Waist circumference | rs1357079 | C | T | 0.570 | 0.011 | 0.002 | 6.20E-10 | 6.13E-05 | 28.333 |
| Waist circumference | rs7372674 | A | C | 0.357 | 0.012 | 0.002 | 1.70E-10 | 6.50E-05 | 30.031 |
| Waist circumference | rs74395133 | C | T | 0.134 | -0.016 | 0.003 | 6.60E-10 | 6.15E-05 | 28.415 |
| Waist circumference | rs1320903 | A | G | 0.320 | 0.017 | 0.002 | 5.20E-19 | 1.27E-04 | 58.602 |
| Waist circumference | rs8192675 | C | T | 0.289 | 0.016 | 0.002 | 4.50E-16 | 1.05E-04 | 48.545 |
| Waist circumference | rs1117619 | G | C | 0.250 | -0.012 | 0.002 | 5.00E-09 | 5.45E-05 | 25.179 |
| Waist circumference | rs3113509 | T | C | 0.732 | -0.012 | 0.002 | 1.00E-09 | 5.96E-05 | 27.568 |
| Waist circumference | rs7377083 | A | C | 0.431 | 0.014 | 0.002 | 3.20E-15 | 1.01E-04 | 46.617 |
| Waist circumference | rs59068084 | T | G | 0.410 | 0.010 | 0.002 | 2.50E-08 | 4.98E-05 | 22.996 |
| Waist circumference | rs1296328 | C | A | 0.559 | -0.013 | 0.002 | 3.10E-13 | 8.58E-05 | 39.653 |
| Waist circumference | rs6846041 | G | C | 0.321 | 0.012 | 0.002 | 1.30E-10 | 6.60E-05 | 30.493 |
| Waist circumference | rs6849518 | T | C | 0.124 | 0.022 | 0.003 | 7.10E-16 | 1.04E-04 | 48.093 |
| Waist circumference | rs1229984 | C | T | 0.973 | 0.030 | 0.005 | 2.80E-08 | 4.81E-05 | 22.234 |
| Waist circumference | rs11099020 | T | C | 0.641 | -0.011 | 0.002 | 1.70E-09 | 5.82E-05 | 26.912 |
| Waist circumference | rs6536575 | C | T | 0.519 | 0.011 | 0.002 | 1.40E-09 | 5.87E-05 | 27.132 |
| Waist circumference | rs1037702 | A | G | 0.622 | -0.010 | 0.002 | 3.60E-08 | 4.88E-05 | 22.560 |
| Waist circumference | rs1051613 | A | G | 0.545 | -0.010 | 0.002 | 3.10E-08 | 4.91E-05 | 22.706 |
| Waist circumference | rs1346841 | A | G | 0.405 | -0.011 | 0.002 | 6.40E-09 | 5.42E-05 | 25.047 |
| Waist circumference | rs4689465 | C | T | 0.525 | -0.011 | 0.002 | 1.30E-09 | 5.89E-05 | 27.229 |
| Waist circumference | rs10938398 | A | G | 0.434 | 0.022 | 0.002 | 1.00E-34 | 2.42E-04 | 112.020 |
| Waist circumference | rs4148155 | G | A | 0.113 | -0.019 | 0.003 | 1.50E-11 | 7.23E-05 | 33.427 |
| Waist circumference | rs4525978 | T | C | 0.735 | -0.011 | 0.002 | 2.10E-08 | 5.05E-05 | 23.325 |
| Waist circumference | rs4527444 | G | A | 0.541 | 0.011 | 0.002 | 4.50E-09 | 5.50E-05 | 25.421 |
| Waist circumference | rs923994 | G | A | 0.783 | -0.013 | 0.002 | 2.20E-09 | 5.75E-05 | 26.564 |
| Waist circumference | rs4419475 | T | A | 0.407 | 0.011 | 0.002 | 6.00E-10 | 6.13E-05 | 28.312 |
| Waist circumference | rs400031 | G | A | 0.756 | 0.012 | 0.002 | 2.10E-08 | 5.39E-05 | 24.901 |
| Waist circumference | rs2307111 | C | T | 0.395 | -0.024 | 0.002 | 3.30E-39 | 2.75E-04 | 126.971 |
| Waist circumference | rs13182474 | C | G | 0.319 | -0.012 | 0.002 | 1.00E-09 | 5.97E-05 | 27.590 |
| Waist circumference | rs67632512 | A | C | 0.117 | 0.017 | 0.003 | 3.80E-09 | 5.69E-05 | 26.301 |
| Waist circumference | rs40067 | A | G | 0.170 | -0.016 | 0.002 | 6.10E-11 | 6.88E-05 | 31.815 |
| Waist circumference | rs2133561 | T | A | 0.611 | -0.012 | 0.002 | 3.90E-11 | 7.13E-05 | 32.936 |
| Waist circumference | rs852983 | A | G | 0.460 | -0.010 | 0.002 | 4.20E-08 | 4.80E-05 | 22.191 |
| Waist circumference | rs3936510 | T | G | 0.201 | 0.014 | 0.002 | 7.30E-10 | 6.05E-05 | 27.950 |
| Waist circumference | rs10471636 | A | G | 0.509 | -0.010 | 0.002 | 3.00E-08 | 5.11E-05 | 23.618 |
| Waist circumference | rs7707394 | A | G | 0.357 | -0.017 | 0.002 | 2.10E-19 | 1.29E-04 | 59.790 |
| Waist circumference | rs2161097 | T | C | 0.438 | 0.014 | 0.002 | 2.20E-15 | 1.00E-04 | 46.380 |
| Waist circumference | rs347551 | G | C | 0.472 | 0.013 | 0.002 | 3.90E-12 | 7.95E-05 | 36.755 |
| Waist circumference | rs13163306 | A | G | 0.466 | -0.010 | 0.002 | 3.50E-08 | 4.87E-05 | 22.498 |
| Waist circumference | rs7708584 | G | A | 0.572 | -0.012 | 0.002 | 1.40E-11 | 7.28E-05 | 33.663 |
| Waist circumference | rs36140 | C | A | 0.635 | 0.011 | 0.002 | 2.10E-09 | 5.79E-05 | 26.752 |
| Waist circumference | rs4706004 | G | A | 0.217 | -0.013 | 0.002 | 5.40E-10 | 6.16E-05 | 28.457 |
| Waist circumference | rs876605 | G | A | 0.740 | -0.011 | 0.002 | 4.20E-08 | 4.80E-05 | 22.174 |
| Waist circumference | rs7442885 | G | C | 0.214 | -0.020 | 0.002 | 6.80E-21 | 1.41E-04 | 65.179 |
| Waist circumference | rs34483452 | A | C | 0.136 | 0.027 | 0.003 | 7.30E-25 | 1.72E-04 | 79.650 |
| Waist circumference | rs1582931 | A | G | 0.473 | -0.014 | 0.002 | 1.70E-14 | 9.57E-05 | 44.235 |
| Waist circumference | rs4469245 | T | A | 0.663 | -0.012 | 0.002 | 1.10E-09 | 5.93E-05 | 27.426 |
| Waist circumference | rs245767 | G | A | 0.730 | 0.015 | 0.002 | 4.50E-13 | 8.39E-05 | 38.792 |
| Waist circumference | rs9654453 | C | T | 0.129 | 0.017 | 0.003 | 1.70E-10 | 6.54E-05 | 30.211 |
| Waist circumference | rs1625623 | T | C | 0.372 | 0.011 | 0.002 | 9.80E-09 | 5.39E-05 | 24.912 |
| Waist circumference | rs4552632 | A | G | 0.617 | -0.010 | 0.002 | 3.30E-08 | 4.89E-05 | 22.600 |
| Waist circumference | rs7752202 | T | C | 0.145 | 0.018 | 0.003 | 2.70E-12 | 7.77E-05 | 35.936 |
| Waist circumference | rs9294260 | A | G | 0.477 | 0.013 | 0.002 | 2.40E-13 | 8.66E-05 | 40.046 |
| Waist circumference | rs765876 | G | A | 0.489 | -0.010 | 0.002 | 4.10E-08 | 4.81E-05 | 22.223 |
| Waist circumference | rs3806114 | A | G | 0.668 | -0.011 | 0.002 | 1.80E-08 | 5.18E-05 | 23.962 |
| Waist circumference | rs72892910 | T | G | 0.172 | 0.030 | 0.002 | 5.20E-37 | 2.59E-04 | 119.760 |
| Waist circumference | rs9370243 | T | G | 0.082 | 0.020 | 0.003 | 1.90E-09 | 5.77E-05 | 26.669 |
| Waist circumference | rs1902066 | C | T | 0.562 | 0.011 | 0.002 | 1.40E-09 | 5.90E-05 | 27.291 |
| Waist circumference | rs7755574 | T | G | 0.283 | 0.011 | 0.002 | 3.90E-08 | 4.83E-05 | 22.333 |
| Waist circumference | rs9378676 | C | A | 0.234 | 0.013 | 0.002 | 7.00E-10 | 6.09E-05 | 28.138 |
| Waist circumference | rs11757278 | C | T | 0.304 | -0.013 | 0.002 | 4.90E-11 | 6.91E-05 | 31.955 |
| Waist circumference | rs28366156 | C | T | 0.131 | -0.019 | 0.003 | 1.50E-12 | 8.00E-05 | 36.958 |
| Waist circumference | rs1327259 | G | A | 0.388 | -0.012 | 0.002 | 3.10E-10 | 6.37E-05 | 29.460 |
| Waist circumference | rs72959041 | A | G | 0.049 | 0.030 | 0.004 | 5.70E-13 | 8.53E-05 | 39.437 |
| Waist circumference | rs10947793 | G | A | 0.372 | -0.013 | 0.002 | 8.10E-12 | 7.56E-05 | 34.940 |
| Waist circumference | rs2744938 | G | A | 0.148 | 0.032 | 0.003 | 5.40E-37 | 2.57E-04 | 118.823 |
| Waist circumference | rs1321521 | A | C | 0.345 | 0.014 | 0.002 | 5.10E-14 | 9.04E-05 | 41.796 |
| Waist circumference | rs10499014 | G | C | 0.269 | -0.013 | 0.002 | 5.90E-11 | 6.93E-05 | 32.041 |
| Waist circumference | rs6938973 | C | T | 0.601 | 0.012 | 0.002 | 4.90E-11 | 6.92E-05 | 31.963 |
| Waist circumference | rs2253310 | G | C | 0.626 | 0.018 | 0.002 | 6.50E-23 | 1.55E-04 | 71.725 |
| Waist circumference | rs520478 | T | G | 0.701 | -0.013 | 0.002 | 2.30E-10 | 6.55E-05 | 30.277 |
| Waist circumference | rs9478496 | C | T | 0.164 | 0.015 | 0.002 | 1.90E-09 | 5.81E-05 | 26.855 |
| Waist circumference | rs2183947 | A | G | 0.225 | -0.022 | 0.002 | 4.90E-25 | 1.70E-04 | 78.582 |
| Waist circumference | rs34045288 | T | C | 0.334 | 0.020 | 0.002 | 5.80E-27 | 1.85E-04 | 85.423 |
| Waist circumference | rs1570298 | T | A | 0.743 | 0.012 | 0.002 | 3.80E-09 | 5.54E-05 | 25.597 |
| Waist circumference | rs36007635 | A | G | 0.138 | -0.017 | 0.003 | 1.70E-10 | 6.52E-05 | 30.130 |
| Waist circumference | rs1182199 | A | C | 0.304 | -0.014 | 0.002 | 3.60E-12 | 7.74E-05 | 35.776 |
| Waist circumference | rs3807566 | T | G | 0.438 | -0.012 | 0.002 | 1.70E-11 | 7.27E-05 | 33.590 |
| Waist circumference | rs10248298 | A | C | 0.366 | 0.013 | 0.002 | 8.80E-13 | 8.15E-05 | 37.690 |
| Waist circumference | rs10236214 | T | C | 0.642 | 0.014 | 0.002 | 1.90E-13 | 8.75E-05 | 40.457 |
| Waist circumference | rs58862095 | T | C | 0.419 | -0.017 | 0.002 | 7.10E-20 | 1.34E-04 | 61.885 |
| Waist circumference | rs12375196 | A | C | 0.424 | 0.013 | 0.002 | 3.80E-13 | 8.54E-05 | 39.457 |
| Waist circumference | rs4344019 | G | A | 0.917 | 0.020 | 0.003 | 5.70E-10 | 6.15E-05 | 28.404 |
| Waist circumference | rs61223906 | A | G | 0.339 | -0.011 | 0.002 | 8.30E-09 | 5.30E-05 | 24.497 |
| Waist circumference | rs11767811 | A | G | 0.181 | -0.015 | 0.002 | 8.90E-11 | 6.71E-05 | 31.023 |
| Waist circumference | rs11773362 | T | C | 0.336 | -0.010 | 0.002 | 3.20E-08 | 4.90E-05 | 22.643 |
| Waist circumference | rs4722398 | T | C | 0.136 | 0.017 | 0.003 | 1.00E-10 | 6.65E-05 | 30.717 |
| Waist circumference | rs73068448 | T | C | 0.171 | -0.015 | 0.002 | 2.00E-10 | 6.67E-05 | 30.806 |
| Waist circumference | rs4718964 | T | G | 0.413 | 0.012 | 0.002 | 1.80E-11 | 7.27E-05 | 33.624 |
| Waist circumference | rs2470946 | T | G | 0.401 | 0.012 | 0.002 | 1.50E-10 | 6.56E-05 | 30.338 |
| Waist circumference | rs215669 | A | G | 0.612 | -0.013 | 0.002 | 1.10E-11 | 7.43E-05 | 34.361 |
| Waist circumference | rs10257197 | G | A | 0.842 | -0.015 | 0.002 | 3.00E-10 | 6.39E-05 | 29.525 |
| Waist circumference | rs10269774 | A | G | 0.326 | 0.012 | 0.002 | 3.60E-10 | 6.29E-05 | 29.073 |
| Waist circumference | rs883403 | C | T | 0.154 | -0.018 | 0.002 | 9.50E-13 | 8.15E-05 | 37.678 |
| Waist circumference | rs55794894 | A | G | 0.135 | -0.015 | 0.003 | 2.80E-08 | 4.94E-05 | 22.835 |
| Waist circumference | rs10957087 | A | T | 0.160 | 0.014 | 0.002 | 1.20E-08 | 5.22E-05 | 24.111 |
| Waist circumference | rs7845090 | A | G | 0.709 | -0.020 | 0.002 | 6.30E-23 | 1.57E-04 | 72.623 |
| Waist circumference | rs1609010 | G | A | 0.566 | 0.015 | 0.002 | 1.30E-16 | 1.10E-04 | 50.650 |
| Waist circumference | rs11787216 | T | C | 0.369 | 0.012 | 0.002 | 1.20E-09 | 6.16E-05 | 28.492 |
| Waist circumference | rs17446091 | C | T | 0.202 | 0.015 | 0.002 | 2.30E-11 | 7.17E-05 | 33.127 |
| Waist circumference | rs2725371 | G | A | 0.696 | -0.015 | 0.002 | 2.60E-15 | 1.01E-04 | 46.651 |
| Waist circumference | rs36061954 | T | C | 0.399 | 0.011 | 0.002 | 3.60E-10 | 6.29E-05 | 29.094 |
| Waist circumference | rs11778934 | G | C | 0.536 | -0.012 | 0.002 | 1.10E-11 | 7.42E-05 | 34.312 |
| Waist circumference | rs1559900 | T | C | 0.286 | 0.013 | 0.002 | 1.10E-10 | 6.66E-05 | 30.800 |
| Waist circumference | rs4876611 | G | A | 0.720 | 0.015 | 0.002 | 5.30E-14 | 9.08E-05 | 41.957 |
| Waist circumference | rs12549000 | A | T | 0.106 | 0.016 | 0.003 | 1.90E-08 | 5.12E-05 | 23.684 |
| Waist circumference | rs4072917 | A | G | 0.474 | 0.012 | 0.002 | 7.40E-11 | 6.85E-05 | 31.653 |
| Waist circumference | rs59104534 | T | C | 0.299 | 0.011 | 0.002 | 4.70E-08 | 4.81E-05 | 22.239 |
| Waist circumference | rs13264909 | T | A | 0.429 | -0.012 | 0.002 | 1.20E-11 | 7.39E-05 | 34.163 |
| Waist circumference | rs28580375 | G | C | 0.219 | 0.013 | 0.002 | 6.00E-09 | 5.43E-05 | 25.107 |
| Waist circumference | rs2482704 | T | G | 0.427 | -0.012 | 0.002 | 1.50E-10 | 6.54E-05 | 30.242 |
| Waist circumference | rs10992854 | C | T | 0.682 | -0.011 | 0.002 | 5.40E-09 | 5.49E-05 | 25.394 |
| Waist circumference | rs1019240 | T | A | 0.643 | 0.012 | 0.002 | 3.70E-11 | 7.06E-05 | 32.633 |
| Waist circumference | rs10732335 | C | A | 0.443 | -0.015 | 0.002 | 3.10E-16 | 1.07E-04 | 49.568 |
| Waist circumference | rs4742782 | G | C | 0.316 | 0.012 | 0.002 | 8.00E-11 | 6.74E-05 | 31.172 |
| Waist circumference | rs1752169 | A | C | 0.251 | 0.014 | 0.002 | 4.50E-12 | 7.69E-05 | 35.550 |
| Waist circumference | rs10757898 | A | G | 0.520 | -0.010 | 0.002 | 4.00E-08 | 4.96E-05 | 22.920 |
| Waist circumference | rs1360201 | T | C | 0.482 | 0.010 | 0.002 | 4.40E-08 | 4.79E-05 | 22.138 |
| Waist circumference | rs34882821 | T | G | 0.339 | 0.011 | 0.002 | 2.10E-08 | 5.05E-05 | 23.363 |
| Waist circumference | rs1411432 | C | A | 0.186 | 0.015 | 0.002 | 7.70E-11 | 6.82E-05 | 31.536 |
| Waist circumference | rs1111817 | G | C | 0.365 | -0.011 | 0.002 | 2.00E-08 | 5.17E-05 | 23.891 |
| Waist circumference | rs13288841 | A | G | 0.322 | 0.019 | 0.002 | 2.20E-23 | 1.58E-04 | 73.204 |
| Waist circumference | rs1619442 | C | T | 0.859 | -0.016 | 0.003 | 2.40E-10 | 6.42E-05 | 29.660 |
| Waist circumference | rs3949781 | A | T | 0.538 | 0.012 | 0.002 | 1.80E-10 | 6.59E-05 | 30.447 |
| Waist circumference | rs113132247 | A | G | 0.153 | 0.015 | 0.002 | 8.40E-10 | 6.07E-05 | 28.058 |
| Waist circumference | rs12001437 | C | T | 0.368 | 0.011 | 0.002 | 3.80E-09 | 5.56E-05 | 25.689 |
| Waist circumference | rs7034554 | G | A | 0.374 | -0.011 | 0.002 | 1.10E-09 | 5.94E-05 | 27.455 |
| Waist circumference | rs703984 | C | G | 0.415 | -0.011 | 0.002 | 2.90E-10 | 6.39E-05 | 29.531 |
| Waist circumference | rs11196657 | C | T | 0.237 | 0.012 | 0.002 | 3.40E-09 | 5.60E-05 | 25.868 |
| Waist circumference | rs67609008 | C | T | 0.284 | 0.011 | 0.002 | 2.50E-08 | 5.01E-05 | 23.149 |
| Waist circumference | rs11012732 | G | A | 0.332 | 0.019 | 0.002 | 2.40E-24 | 1.66E-04 | 76.758 |
| Waist circumference | rs71495038 | A | G | 0.077 | 0.022 | 0.003 | 2.60E-11 | 7.11E-05 | 32.869 |
| Waist circumference | rs10827380 | T | C | 0.314 | 0.012 | 0.002 | 1.80E-09 | 5.81E-05 | 26.855 |
| Waist circumference | rs10887578 | C | G | 0.498 | 0.011 | 0.002 | 3.10E-09 | 5.69E-05 | 26.301 |
| Waist circumference | rs4075353 | A | G | 0.344 | -0.011 | 0.002 | 2.20E-08 | 5.06E-05 | 23.391 |
| Waist circumference | rs10795418 | G | A | 0.665 | 0.013 | 0.002 | 2.40E-11 | 7.19E-05 | 33.229 |
| Waist circumference | rs7070670 | T | C | 0.328 | -0.012 | 0.002 | 3.50E-10 | 6.38E-05 | 29.493 |
| Waist circumference | rs10824211 | T | C | 0.140 | 0.015 | 0.003 | 6.30E-09 | 5.45E-05 | 25.185 |
| Waist circumference | rs12245654 | C | A | 0.073 | 0.023 | 0.003 | 3.20E-11 | 7.15E-05 | 33.042 |
| Waist circumference | rs2439823 | G | A | 0.546 | 0.016 | 0.002 | 6.80E-18 | 1.20E-04 | 55.243 |
| Waist circumference | rs4290163 | T | G | 0.393 | 0.011 | 0.002 | 5.40E-10 | 6.17E-05 | 28.538 |
| Waist circumference | rs10787738 | T | C | 0.255 | 0.015 | 0.002 | 2.10E-13 | 8.89E-05 | 41.097 |
| Waist circumference | rs2172131 | C | T | 0.579 | -0.012 | 0.002 | 1.90E-11 | 7.23E-05 | 33.403 |
| Waist circumference | rs2696309 | C | T | 0.720 | 0.011 | 0.002 | 1.60E-08 | 5.10E-05 | 23.594 |
| Waist circumference | rs12225345 | G | A | 0.166 | 0.015 | 0.002 | 1.30E-09 | 5.89E-05 | 27.231 |
| Waist circumference | rs11603984 | T | G | 0.135 | -0.017 | 0.003 | 8.90E-11 | 6.74E-05 | 31.162 |
| Waist circumference | rs61903695 | G | A | 0.255 | 0.013 | 0.002 | 5.90E-11 | 6.87E-05 | 31.770 |
| Waist circumference | rs35023999 | C | A | 0.508 | -0.011 | 0.002 | 2.40E-10 | 6.43E-05 | 29.715 |
| Waist circumference | rs111258054 | T | C | 0.184 | 0.016 | 0.002 | 9.50E-12 | 7.63E-05 | 35.272 |
| Waist circumference | rs1502317 | T | C | 0.277 | -0.017 | 0.002 | 4.30E-18 | 1.21E-04 | 55.798 |
| Waist circumference | rs11824092 | C | T | 0.636 | 0.012 | 0.002 | 2.40E-11 | 7.23E-05 | 33.399 |
| Waist circumference | rs2225909 | C | T | 0.774 | 0.016 | 0.002 | 1.40E-13 | 8.73E-05 | 40.339 |
| Waist circumference | rs35243581 | T | C | 0.317 | 0.017 | 0.002 | 1.70E-19 | 1.31E-04 | 60.382 |
| Waist circumference | rs7925100 | A | G | 0.396 | 0.014 | 0.002 | 1.70E-14 | 9.43E-05 | 43.593 |
| Waist circumference | rs1013402 | G | A | 0.318 | 0.025 | 0.002 | 3.90E-39 | 2.73E-04 | 126.324 |
| Waist circumference | rs10835676 | G | C | 0.240 | 0.012 | 0.002 | 4.00E-09 | 5.57E-05 | 25.753 |
| Waist circumference | rs11223204 | G | A | 0.434 | 0.012 | 0.002 | 1.50E-11 | 7.33E-05 | 33.887 |
| Waist circumference | rs11218510 | A | G | 0.400 | -0.012 | 0.002 | 2.90E-10 | 6.39E-05 | 29.517 |
| Waist circumference | rs7933085 | G | A | 0.508 | 0.011 | 0.002 | 1.40E-09 | 5.93E-05 | 27.394 |
| Waist circumference | rs7115013 | T | C | 0.443 | -0.011 | 0.002 | 3.70E-09 | 5.59E-05 | 25.816 |
| Waist circumference | rs12287076 | C | G | 0.707 | 0.021 | 0.002 | 1.30E-26 | 1.84E-04 | 84.956 |
| Waist circumference | rs7952436 | T | C | 0.082 | -0.029 | 0.003 | 5.90E-19 | 1.27E-04 | 58.494 |
| Waist circumference | rs11215381 | C | T | 0.526 | 0.011 | 0.002 | 4.60E-10 | 6.23E-05 | 28.787 |
| Waist circumference | rs12273545 | T | C | 0.056 | 0.022 | 0.004 | 9.40E-09 | 5.28E-05 | 24.423 |
| Waist circumference | rs735033 | G | A | 0.605 | -0.010 | 0.002 | 2.50E-08 | 5.04E-05 | 23.277 |
| Waist circumference | rs7966251 | A | G | 0.255 | -0.012 | 0.002 | 1.70E-08 | 5.11E-05 | 23.628 |
| Waist circumference | rs3764002 | T | C | 0.262 | -0.016 | 0.002 | 2.80E-15 | 9.98E-05 | 46.150 |
| Waist circumference | rs11058233 | G | A | 0.751 | 0.016 | 0.002 | 1.20E-14 | 9.55E-05 | 44.138 |
| Waist circumference | rs7132908 | A | G | 0.384 | 0.022 | 0.002 | 1.40E-31 | 2.19E-04 | 101.257 |
| Waist circumference | rs3816760 | A | G | 0.307 | 0.013 | 0.002 | 5.70E-12 | 7.59E-05 | 35.098 |
| Waist circumference | rs10505836 | C | A | 0.860 | 0.015 | 0.003 | 4.10E-09 | 5.61E-05 | 25.949 |
| Waist circumference | rs894736 | G | A | 0.363 | 0.016 | 0.002 | 3.60E-17 | 1.14E-04 | 52.924 |
| Waist circumference | rs704061 | C | T | 0.455 | 0.015 | 0.002 | 4.00E-16 | 1.06E-04 | 49.014 |
| Waist circumference | rs55726687 | A | G | 0.210 | 0.020 | 0.002 | 1.10E-19 | 1.32E-04 | 60.904 |
| Waist circumference | rs145350287 | A | T | 0.040 | -0.032 | 0.005 | 2.00E-12 | 7.91E-05 | 36.539 |
| Waist circumference | rs36165342 | C | T | 0.479 | 0.011 | 0.002 | 2.10E-09 | 5.73E-05 | 26.468 |
| Waist circumference | rs76895963 | G | T | 0.021 | 0.048 | 0.007 | 2.80E-12 | 9.50E-05 | 43.909 |
| Waist circumference | rs1458156 | T | C | 0.488 | 0.015 | 0.002 | 3.50E-16 | 1.07E-04 | 49.261 |
| Waist circumference | rs11614326 | A | G | 0.545 | -0.010 | 0.002 | 1.60E-08 | 5.22E-05 | 24.132 |
| Waist circumference | rs9568867 | A | G | 0.129 | 0.023 | 0.003 | 4.20E-17 | 1.15E-04 | 53.206 |
| Waist circumference | rs1441264 | A | G | 0.594 | 0.015 | 0.002 | 7.60E-16 | 1.09E-04 | 50.236 |
| Waist circumference | rs9584870 | C | T | 0.366 | -0.011 | 0.002 | 1.10E-08 | 5.43E-05 | 25.087 |
| Waist circumference | rs34140906 | C | T | 0.170 | -0.018 | 0.002 | 5.40E-14 | 9.10E-05 | 42.040 |
| Waist circumference | rs12877270 | A | G | 0.442 | 0.012 | 0.002 | 1.10E-10 | 6.78E-05 | 31.332 |
| Waist circumference | rs1183668 | G | C | 0.370 | -0.012 | 0.002 | 9.30E-11 | 6.78E-05 | 31.339 |
| Waist circumference | rs1218824 | A | G | 0.661 | 0.012 | 0.002 | 4.20E-11 | 6.99E-05 | 32.295 |
| Waist circumference | rs9316661 | C | T | 0.801 | -0.016 | 0.002 | 3.80E-12 | 7.76E-05 | 35.866 |
| Waist circumference | rs9888533 | T | C | 0.538 | 0.011 | 0.002 | 3.00E-09 | 5.83E-05 | 26.960 |
| Waist circumference | rs11842871 | T | G | 0.260 | -0.013 | 0.002 | 4.00E-10 | 6.29E-05 | 29.080 |
| Waist circumference | rs1336486 | G | T | 0.329 | 0.012 | 0.002 | 6.00E-11 | 6.88E-05 | 31.809 |
| Waist circumference | rs7324067 | C | T | 0.761 | 0.012 | 0.002 | 7.70E-09 | 5.35E-05 | 24.718 |
| Waist circumference | rs61969511 | A | G | 0.279 | 0.012 | 0.002 | 3.80E-09 | 5.66E-05 | 26.138 |
| Waist circumference | rs484455 | A | G | 0.481 | -0.012 | 0.002 | 1.40E-10 | 6.64E-05 | 30.699 |
| Waist circumference | rs1188209 | G | A | 0.552 | 0.010 | 0.002 | 1.60E-08 | 5.19E-05 | 23.973 |
| Waist circumference | rs3212038 | G | A | 0.329 | 0.013 | 0.002 | 3.50E-11 | 7.07E-05 | 32.655 |
| Waist circumference | rs11160600 | G | A | 0.092 | 0.018 | 0.003 | 7.10E-09 | 5.46E-05 | 25.237 |
| Waist circumference | rs8013377 | C | A | 0.270 | -0.017 | 0.002 | 1.90E-16 | 1.09E-04 | 50.225 |
| Waist circumference | rs4900715 | A | G | 0.507 | -0.011 | 0.002 | 1.90E-10 | 6.53E-05 | 30.174 |
| Waist circumference | rs61992671 | G | A | 0.492 | -0.013 | 0.002 | 3.80E-12 | 8.45E-05 | 39.049 |
| Waist circumference | rs12880641 | G | T | 0.662 | -0.014 | 0.002 | 2.40E-13 | 8.58E-05 | 39.672 |
| Waist circumference | rs2180454 | C | T | 0.772 | 0.018 | 0.002 | 3.70E-17 | 1.14E-04 | 52.644 |
| Waist circumference | rs1191600 | A | C | 0.594 | -0.011 | 0.002 | 3.80E-09 | 5.63E-05 | 26.013 |
| Waist circumference | rs217672 | C | A | 0.272 | 0.013 | 0.002 | 2.80E-10 | 6.42E-05 | 29.651 |
| Waist circumference | rs10150482 | A | G | 0.220 | 0.022 | 0.002 | 4.50E-24 | 1.66E-04 | 76.572 |
| Waist circumference | rs6575340 | A | G | 0.636 | 0.016 | 0.002 | 4.60E-18 | 1.21E-04 | 55.876 |
| Waist circumference | rs3784692 | T | C | 0.602 | 0.019 | 0.002 | 2.60E-24 | 1.66E-04 | 76.502 |
| Waist circumference | rs11636611 | T | C | 0.503 | 0.011 | 0.002 | 2.30E-09 | 5.74E-05 | 26.529 |
| Waist circumference | rs17296856 | C | A | 0.281 | -0.016 | 0.002 | 5.00E-15 | 9.86E-05 | 45.552 |
| Waist circumference | rs6493498 | C | T | 0.545 | -0.013 | 0.002 | 4.20E-13 | 8.49E-05 | 39.224 |
| Waist circumference | rs7169847 | T | G | 0.636 | -0.010 | 0.002 | 3.80E-08 | 4.89E-05 | 22.581 |
| Waist circumference | rs56803094 | G | A | 0.227 | -0.013 | 0.002 | 2.60E-09 | 5.72E-05 | 26.445 |
| Waist circumference | rs80243702 | A | G | 0.161 | 0.015 | 0.002 | 6.70E-10 | 6.20E-05 | 28.666 |
| Waist circumference | rs1657930 | A | G | 0.803 | -0.014 | 0.002 | 2.30E-10 | 6.42E-05 | 29.695 |
| Waist circumference | rs7498044 | A | G | 0.217 | -0.015 | 0.002 | 4.90E-12 | 7.81E-05 | 36.087 |
| Waist circumference | rs8024137 | T | A | 0.848 | 0.014 | 0.003 | 4.60E-08 | 4.82E-05 | 22.291 |
| Waist circumference | rs7171864 | A | G | 0.660 | 0.013 | 0.002 | 1.10E-11 | 7.45E-05 | 34.453 |
| Waist circumference | rs34994596 | C | T | 0.297 | -0.015 | 0.002 | 1.10E-13 | 8.86E-05 | 40.934 |
| Waist circumference | rs28375268 | T | G | 0.645 | -0.013 | 0.002 | 3.30E-12 | 7.83E-05 | 36.204 |
| Waist circumference | rs7498665 | G | A | 0.400 | 0.027 | 0.002 | 4.90E-48 | 3.40E-04 | 157.166 |
| Waist circumference | rs3814883 | T | C | 0.482 | 0.024 | 0.002 | 1.10E-40 | 2.87E-04 | 132.772 |
| Waist circumference | rs13333747 | C | T | 0.183 | -0.023 | 0.002 | 1.90E-22 | 1.54E-04 | 70.989 |
| Waist circumference | rs12103006 | G | A | 0.569 | 0.012 | 0.002 | 5.10E-12 | 7.64E-05 | 35.294 |
| Waist circumference | rs756717 | A | G | 0.399 | -0.011 | 0.002 | 6.80E-09 | 5.51E-05 | 25.452 |
| Waist circumference | rs862227 | G | A | 0.458 | -0.011 | 0.002 | 8.80E-10 | 5.99E-05 | 27.663 |
| Waist circumference | rs7206608 | G | C | 0.322 | 0.012 | 0.002 | 3.80E-10 | 6.28E-05 | 29.044 |
| Waist circumference | rs2376885 | A | G | 0.324 | -0.011 | 0.002 | 2.80E-08 | 4.94E-05 | 22.849 |
| Waist circumference | rs9926784 | C | T | 0.185 | -0.013 | 0.002 | 8.70E-09 | 5.29E-05 | 24.454 |
| Waist circumference | rs56094641 | G | A | 0.405 | 0.058 | 0.002 | 1.00E-200 | 1.60E-03 | 738.707 |
| Waist circumference | rs12926311 | C | G | 0.354 | -0.012 | 0.002 | 3.20E-11 | 7.09E-05 | 32.791 |
| Waist circumference | rs879620 | T | C | 0.613 | 0.019 | 0.002 | 4.90E-26 | 1.79E-04 | 82.895 |
| Waist circumference | rs11639596 | C | A | 0.251 | -0.012 | 0.002 | 1.10E-08 | 5.30E-05 | 24.480 |
| Waist circumference | rs862320 | T | C | 0.410 | -0.018 | 0.002 | 4.40E-23 | 1.57E-04 | 72.649 |
| Waist circumference | rs9673839 | G | A | 0.491 | 0.011 | 0.002 | 1.30E-09 | 5.95E-05 | 27.479 |
| Waist circumference | rs1025065 | G | T | 0.639 | -0.010 | 0.002 | 3.60E-08 | 4.88E-05 | 22.549 |
| Waist circumference | rs3826408 | T | C | 0.457 | 0.011 | 0.002 | 4.10E-10 | 6.25E-05 | 28.900 |
| Waist circumference | rs8078135 | T | C | 0.490 | -0.010 | 0.002 | 1.60E-08 | 5.14E-05 | 23.779 |
| Waist circumference | rs2020942 | T | C | 0.395 | 0.011 | 0.002 | 3.40E-09 | 5.62E-05 | 25.988 |
| Waist circumference | rs319775 | C | T | 0.609 | 0.010 | 0.002 | 3.00E-08 | 4.91E-05 | 22.707 |
| Waist circumference | rs11150745 | G | A | 0.318 | -0.016 | 0.002 | 6.80E-17 | 1.12E-04 | 51.845 |
| Waist circumference | rs113866544 | C | T | 0.068 | 0.030 | 0.004 | 1.40E-17 | 1.17E-04 | 54.111 |
| Waist circumference | rs62072003 | T | C | 0.143 | 0.014 | 0.003 | 1.80E-08 | 5.12E-05 | 23.642 |
| Waist circumference | rs7218014 | C | T | 0.197 | 0.022 | 0.002 | 2.70E-23 | 1.59E-04 | 73.526 |
| Waist circumference | rs17681738 | T | C | 0.329 | 0.011 | 0.002 | 3.30E-08 | 4.90E-05 | 22.664 |
| Waist circumference | rs2306593 | T | C | 0.488 | -0.015 | 0.002 | 1.60E-17 | 1.17E-04 | 54.013 |
| Waist circumference | rs9916444 | G | C | 0.342 | 0.011 | 0.002 | 1.30E-09 | 5.92E-05 | 27.375 |
| Waist circumference | rs3935190 | A | G | 0.537 | -0.013 | 0.002 | 2.80E-12 | 7.92E-05 | 36.604 |
| Waist circumference | rs4790841 | T | C | 0.154 | -0.021 | 0.002 | 1.30E-17 | 1.18E-04 | 54.548 |
| Waist circumference | rs9902846 | T | C | 0.316 | 0.013 | 0.002 | 4.20E-12 | 7.73E-05 | 35.748 |
| Waist circumference | rs11653367 | G | A | 0.328 | -0.015 | 0.002 | 2.50E-15 | 1.01E-04 | 46.879 |
| Waist circumference | rs8097672 | T | A | 0.145 | 0.017 | 0.003 | 5.00E-11 | 6.99E-05 | 32.312 |
| Waist circumference | rs1834144 | A | C | 0.373 | -0.015 | 0.002 | 3.40E-15 | 9.99E-05 | 46.190 |
| Waist circumference | rs1788808 | G | A | 0.495 | -0.021 | 0.002 | 3.60E-31 | 2.17E-04 | 100.176 |
| Waist circumference | rs784257 | C | T | 0.813 | 0.016 | 0.002 | 2.30E-12 | 7.97E-05 | 36.850 |
| Waist circumference | rs57636386 | C | T | 0.084 | -0.031 | 0.003 | 1.50E-21 | 1.47E-04 | 67.816 |
| Waist circumference | rs1942826 | A | G | 0.126 | 0.017 | 0.003 | 4.90E-10 | 6.21E-05 | 28.716 |
| Waist circumference | rs2584205 | A | G | 0.733 | 0.011 | 0.002 | 3.70E-08 | 4.87E-05 | 22.514 |
| Waist circumference | rs6567160 | C | T | 0.233 | 0.045 | 0.002 | 7.19E-101 | 7.28E-04 | 336.922 |
| Waist circumference | rs559231 | T | G | 0.393 | 0.011 | 0.002 | 5.20E-09 | 5.52E-05 | 25.493 |
| Waist circumference | rs72976986 | A | G | 0.190 | -0.016 | 0.002 | 7.80E-12 | 7.65E-05 | 35.348 |
| Waist circumference | rs2302209 | T | C | 0.289 | 0.020 | 0.002 | 1.10E-23 | 1.62E-04 | 74.690 |
| Waist circumference | rs429358 | C | T | 0.154 | -0.027 | 0.002 | 7.50E-28 | 1.92E-04 | 88.803 |
| Waist circumference | rs10423928 | A | T | 0.194 | -0.027 | 0.002 | 6.80E-32 | 2.21E-04 | 102.083 |
| Waist circumference | rs2074881 | T | C | 0.168 | -0.015 | 0.002 | 8.00E-10 | 6.11E-05 | 28.218 |
| Waist circumference | rs8112818 | G | A | 0.400 | -0.016 | 0.002 | 3.80E-18 | 1.22E-04 | 56.243 |
| Waist circumference | rs11666480 | G | C | 0.536 | 0.013 | 0.002 | 1.90E-13 | 8.78E-05 | 40.594 |
| Waist circumference | rs12983532 | T | C | 0.251 | -0.015 | 0.002 | 7.60E-13 | 8.46E-05 | 39.097 |
| Waist circumference | rs12462975 | A | G | 0.330 | 0.018 | 0.002 | 5.90E-20 | 1.36E-04 | 62.851 |
| Waist circumference | rs2903738 | T | A | 0.221 | -0.013 | 0.002 | 6.80E-10 | 6.10E-05 | 28.213 |
| Waist circumference | rs10406327 | G | C | 0.479 | 0.010 | 0.002 | 8.00E-09 | 5.36E-05 | 24.766 |
| Waist circumference | rs7259070 | C | T | 0.596 | 0.015 | 0.002 | 6.60E-17 | 1.14E-04 | 52.656 |
| Waist circumference | rs73142879 | T | C | 0.192 | -0.024 | 0.002 | 2.80E-26 | 1.82E-04 | 84.057 |
| Waist circumference | rs1056441 | C | T | 0.675 | 0.013 | 0.002 | 3.10E-11 | 7.08E-05 | 32.718 |
| Waist circumference | rs6069037 | A | C | 0.731 | -0.011 | 0.002 | 3.60E-08 | 4.86E-05 | 22.476 |
| Waist circumference | rs852042 | G | A | 0.759 | -0.012 | 0.002 | 3.00E-08 | 4.92E-05 | 22.760 |
| Waist circumference | rs4456769 | T | C | 0.333 | 0.013 | 0.002 | 1.80E-12 | 7.98E-05 | 36.891 |
| Waist circumference | rs6030803 | C | T | 0.127 | -0.018 | 0.003 | 6.10E-11 | 6.91E-05 | 31.950 |
| Waist circumference | rs76040172 | A | G | 0.054 | -0.029 | 0.004 | 1.50E-13 | 8.88E-05 | 41.029 |
| Waist circumference | rs13047416 | G | C | 0.377 | -0.014 | 0.002 | 6.60E-14 | 9.08E-05 | 41.956 |
| Waist circumference | rs11704728 | T | C | 0.196 | 0.013 | 0.002 | 3.90E-09 | 5.61E-05 | 25.921 |
| Waist circumference | rs6001877 | A | G | 0.340 | -0.011 | 0.002 | 2.40E-08 | 5.02E-05 | 23.211 |
| Waist circumference | rs28489620 | A | G | 0.290 | -0.012 | 0.002 | 4.40E-10 | 6.35E-05 | 29.348 |
| Waist circumference | rs165656 | C | G | 0.517 | 0.010 | 0.002 | 1.10E-08 | 5.30E-05 | 24.507 |
| Hypothyroidism | rs6679677 | A | C | 0.108 | 0.364 | 0.016 | 2.39E-115 | 2.56E-02 | 10766.012 |
| Hypothyroidism | rs2988277 | T | C | 0.287 | 0.059 | 0.011 | 2.49E-08 | 1.44E-03 | 591.136 |
| Hypothyroidism | rs2234167 | A | G | 0.102 | 0.083 | 0.015 | 3.75E-08 | 1.25E-03 | 512.078 |
| Hypothyroidism | rs926103 | C | T | 0.698 | -0.068 | 0.010 | 7.65E-11 | 1.94E-03 | 796.412 |
| Hypothyroidism | rs78765971 | G | GAC | 0.147 | 0.244 | 0.016 | 1.68E-51 | 1.50E-02 | 6235.059 |
| Hypothyroidism | rs12117927 | A | C | 0.477 | 0.063 | 0.011 | 2.29E-09 | 1.96E-03 | 805.994 |
| Hypothyroidism | rs114285740 | C | G | 0.027 | 0.167 | 0.030 | 3.06E-08 | 1.48E-03 | 609.338 |
| Hypothyroidism | rs10917477 | G | A | 0.387 | 0.064 | 0.010 | 1.75E-10 | 1.94E-03 | 798.539 |
| Hypothyroidism | rs7574865 | G | T | 0.743 | -0.132 | 0.012 | 1.67E-29 | 6.67E-03 | 2755.027 |
| Hypothyroidism | rs1534430 | T | C | 0.428 | -0.086 | 0.010 | 1.44E-17 | 3.62E-03 | 1490.988 |
| Hypothyroidism | rs11420448 | GT | G | 0.047 | 0.153 | 0.028 | 2.39E-08 | 2.09E-03 | 860.017 |
| Hypothyroidism | rs3087243 | A | G | 0.386 | -0.147 | 0.010 | 4.77E-47 | 1.02E-02 | 4221.519 |
| Hypothyroidism | rs2111485 | G | A | 0.479 | 0.081 | 0.010 | 1.43E-15 | 3.30E-03 | 1357.539 |
| Hypothyroidism | rs11675342 | T | C | 0.388 | 0.091 | 0.010 | 1.40E-19 | 3.90E-03 | 1604.668 |
| Hypothyroidism | rs13090803 | T | G | 0.191 | 0.083 | 0.013 | 9.00E-11 | 2.12E-03 | 871.606 |
| Hypothyroidism | rs73192661 | T | C | 0.429 | -0.106 | 0.010 | 4.05E-26 | 5.51E-03 | 2274.317 |
| Hypothyroidism | rs307558 | A | G | 0.743 | -0.069 | 0.012 | 8.01E-09 | 1.81E-03 | 743.465 |
| Hypothyroidism | rs4835534 | C | T | 0.156 | -0.142 | 0.013 | 7.06E-27 | 5.33E-03 | 2196.558 |
| Hypothyroidism | rs7441808 | G | A | 0.211 | 0.077 | 0.011 | 5.17E-12 | 1.95E-03 | 802.586 |
| Hypothyroidism | rs187707293 | A | T | 0.013 | 0.242 | 0.044 | 3.99E-08 | 1.55E-03 | 634.915 |
| Hypothyroidism | rs3775291 | T | C | 0.288 | -0.065 | 0.011 | 1.65E-09 | 1.73E-03 | 709.387 |
| Hypothyroidism | rs13109179 | A | G | 0.462 | 0.065 | 0.010 | 9.42E-11 | 2.08E-03 | 855.233 |
| Hypothyroidism | rs1479565 | A | G | 0.499 | 0.098 | 0.010 | 7.53E-22 | 4.75E-03 | 1958.742 |
| Hypothyroidism | rs434294 | G | A | 0.323 | -0.068 | 0.011 | 3.36E-10 | 2.04E-03 | 838.187 |
| Hypothyroidism | rs1432806 | G | A | 0.339 | 0.058 | 0.011 | 2.89E-08 | 1.52E-03 | 625.350 |
| Hypothyroidism | rs244685 | G | T | 0.799 | -0.086 | 0.013 | 7.06E-11 | 2.36E-03 | 971.546 |
| Hypothyroidism | rs10075764 | G | A | 0.302 | -0.057 | 0.010 | 4.26E-08 | 1.37E-03 | 562.996 |
| Hypothyroidism | rs9264277 | C | T | 0.645 | -0.086 | 0.011 | 9.03E-15 | 3.40E-03 | 1400.247 |
| Hypothyroidism | rs28418426 | C | T | 0.501 | 0.188 | 0.013 | 2.21E-45 | 1.76E-02 | 7354.375 |
| Hypothyroidism | rs7742626 | C | T | 0.267 | 0.069 | 0.012 | 3.41E-09 | 1.84E-03 | 757.496 |
| Hypothyroidism | rs1065386 | C | G | 0.480 | -0.118 | 0.013 | 3.93E-21 | 6.99E-03 | 2885.705 |
| Hypothyroidism | rs881858 | A | G | 0.748 | 0.067 | 0.011 | 8.46E-10 | 1.67E-03 | 685.089 |
| Hypothyroidism | rs6908626 | T | G | 0.171 | 0.144 | 0.014 | 2.04E-24 | 5.88E-03 | 2426.032 |
| Hypothyroidism | rs2247314 | C | T | 0.376 | -0.086 | 0.010 | 1.06E-16 | 3.47E-03 | 1429.117 |
| Hypothyroidism | rs9271365 | G | T | 0.443 | 0.248 | 0.011 | 4.91E-123 | 3.05E-02 | 12883.953 |
| Hypothyroidism | rs9273371 | T | C | 0.193 | 0.079 | 0.014 | 1.65E-08 | 1.95E-03 | 802.143 |
| Hypothyroidism | rs9277559 | C | T | 0.331 | -0.133 | 0.012 | 1.86E-28 | 7.83E-03 | 3238.557 |
| Hypothyroidism | rs1364450 | C | A | 0.126 | 0.089 | 0.014 | 1.97E-10 | 1.73E-03 | 711.468 |
| Hypothyroidism | rs9497965 | T | C | 0.409 | 0.083 | 0.010 | 3.71E-16 | 3.31E-03 | 1360.607 |
| Hypothyroidism | rs1079418 | G | A | 0.262 | -0.066 | 0.011 | 2.14E-09 | 1.67E-03 | 686.396 |
| Hypothyroidism | rs141232332 | G | T | 0.451 | 0.224 | 0.013 | 4.48E-70 | 2.49E-02 | 10479.666 |
| Hypothyroidism | rs2921053 | C | G | 0.590 | -0.060 | 0.010 | 3.36E-09 | 1.74E-03 | 713.393 |
| Hypothyroidism | rs853305 | C | T | 0.719 | -0.080 | 0.011 | 4.40E-13 | 2.60E-03 | 1068.994 |
| Hypothyroidism | rs2445608 | A | G | 0.430 | -0.059 | 0.010 | 3.79E-09 | 1.72E-03 | 708.360 |
| Hypothyroidism | rs7030280 | T | C | 0.746 | 0.208 | 0.011 | 1.02E-82 | 1.63E-02 | 6809.251 |
| Hypothyroidism | rs911760 | A | C | 0.212 | 0.088 | 0.013 | 1.95E-12 | 2.58E-03 | 1062.033 |
| Hypothyroidism | rs10126000 | A | C | 0.689 | -0.068 | 0.010 | 5.13E-11 | 2.00E-03 | 822.029 |
| Hypothyroidism | rs12379417 | A | G | 0.318 | 0.058 | 0.010 | 1.51E-08 | 1.47E-03 | 605.101 |
| Hypothyroidism | rs3118469 | T | A | 0.295 | 0.080 | 0.011 | 3.82E-14 | 2.68E-03 | 1103.364 |
| Hypothyroidism | rs71508903 | T | C | 0.211 | 0.093 | 0.013 | 9.34E-14 | 2.90E-03 | 1193.986 |
| Hypothyroidism | rs4529854 | T | C | 0.723 | -0.077 | 0.011 | 6.56E-13 | 2.36E-03 | 970.294 |
| Hypothyroidism | rs736374 | A | G | 0.375 | 0.083 | 0.010 | 6.00E-16 | 3.25E-03 | 1335.298 |
| Hypothyroidism | rs61877856 | T | C | 0.197 | -0.066 | 0.012 | 1.14E-08 | 1.37E-03 | 563.655 |
| Hypothyroidism | rs4409785 | C | T | 0.143 | 0.107 | 0.013 | 8.04E-16 | 2.79E-03 | 1149.075 |
| Hypothyroidism | rs11171710 | A | G | 0.444 | -0.070 | 0.010 | 3.19E-12 | 2.41E-03 | 988.915 |
| Hypothyroidism | rs7488011 | T | C | 0.356 | 0.105 | 0.011 | 2.52E-21 | 5.08E-03 | 2093.164 |
| Hypothyroidism | rs3184504 | C | T | 0.669 | -0.173 | 0.010 | 7.50E-65 | 1.33E-02 | 5539.391 |
| Hypothyroidism | rs12582330 | T | G | 0.624 | -0.061 | 0.011 | 2.05E-08 | 1.75E-03 | 717.107 |
| Hypothyroidism | rs7990020 | C | A | 0.453 | 0.058 | 0.010 | 9.97E-09 | 1.65E-03 | 677.945 |
| Hypothyroidism | rs9511151 | A | G | 0.280 | -0.098 | 0.011 | 3.08E-20 | 3.84E-03 | 1579.828 |
| Hypothyroidism | rs11406335 | G | T | 0.417 | -0.057 | 0.010 | 3.44E-08 | 1.58E-03 | 649.011 |
| Hypothyroidism | rs79490353 | C | T | 0.024 | 0.201 | 0.035 | 8.82E-09 | 1.89E-03 | 778.277 |
| Hypothyroidism | rs2114702 | A | T | 0.267 | 0.070 | 0.011 | 3.00E-10 | 1.92E-03 | 787.599 |
| Hypothyroidism | rs12593201 | A | G | 0.325 | 0.091 | 0.011 | 7.69E-16 | 3.60E-03 | 1480.160 |
| Hypothyroidism | rs142997491 | G | A | 0.014 | 0.239 | 0.041 | 7.02E-09 | 1.52E-03 | 625.240 |
| Hypothyroidism | rs61759532 | T | C | 0.189 | 0.091 | 0.012 | 1.42E-13 | 2.51E-03 | 1032.116 |
| Hypothyroidism | rs9902341 | T | C | 0.172 | 0.080 | 0.013 | 4.68E-10 | 1.83E-03 | 751.785 |
| Hypothyroidism | rs7223956 | C | T | 0.897 | -0.090 | 0.014 | 4.27E-10 | 1.50E-03 | 617.502 |
| Hypothyroidism | rs11875260 | G | A | 0.173 | 0.075 | 0.014 | 2.54E-08 | 1.61E-03 | 662.743 |
| Hypothyroidism | rs34536443 | C | G | 0.044 | -0.186 | 0.026 | 1.46E-12 | 2.92E-03 | 1201.796 |
| Hypothyroidism | rs10424978 | A | C | 0.624 | -0.078 | 0.010 | 2.77E-14 | 2.82E-03 | 1158.808 |
| Hypothyroidism | rs12984428 | A | G | 0.356 | -0.066 | 0.010 | 1.11E-10 | 1.99E-03 | 818.381 |
| Hypothyroidism | rs2412976 | G | C | 0.479 | 0.064 | 0.010 | 5.47E-10 | 2.03E-03 | 832.311 |
| Hypothyroidism | rs229528 | T | C | 0.496 | 0.090 | 0.010 | 2.31E-19 | 4.08E-03 | 1678.893 |
| Hypothyroidism | rs5912815 | G | T | 0.577 | -0.051 | 0.008 | 1.05E-09 | 1.27E-03 | 523.526 |
| Hyperthyroidism | rs6679677 | A | C | 0.112 | 0.294 | 0.038 | 8.79E-15 | 1.71E-02 | 8028.577 |
| Hyperthyroidism | rs3087243 | A | G | 0.380 | -0.204 | 0.022 | 7.94E-20 | 1.96E-02 | 9194.678 |
| Hyperthyroidism | rs758778 | C | T | 0.204 | 0.307 | 0.035 | 1.02E-18 | 3.06E-02 | 14540.604 |
| Hyperthyroidism | rs385863 | G | C | 0.505 | 0.134 | 0.021 | 4.09E-10 | 8.91E-03 | 4139.956 |
| Hyperthyroidism | rs1794280 | T | A | 0.099 | 0.546 | 0.039 | 2.29E-44 | 5.30E-02 | 25777.319 |
| Hyperthyroidism | rs2856821 | C | T | 0.196 | -0.188 | 0.027 | 7.69E-12 | 1.11E-02 | 5187.153 |
| Hyperthyroidism | rs9258222 | A | G | 0.087 | -0.237 | 0.042 | 1.44E-08 | 8.89E-03 | 4130.982 |
| Hyperthyroidism | rs28375776 | G | C | 0.127 | -0.243 | 0.041 | 2.25E-09 | 1.31E-02 | 6120.985 |
| Hyperthyroidism | rs604912 | G | A | 0.539 | 0.120 | 0.022 | 3.15E-08 | 7.13E-03 | 3308.257 |
| Hyperthyroidism | rs2160215 | C | T | 0.426 | 0.248 | 0.023 | 3.82E-28 | 3.01E-02 | 14312.561 |
| Hyperthyroidism | rs4338740 | C | T | 0.207 | 0.184 | 0.026 | 2.87E-12 | 1.12E-02 | 5200.142 |
| Hyperthyroidism | rs58722186 | T | C | 0.302 | 0.136 | 0.023 | 5.23E-09 | 7.79E-03 | 3615.313 |
| Hyperthyroidism | rs6131010 | G | A | 0.713 | 0.131 | 0.024 | 3.60E-08 | 6.98E-03 | 3238.414 |
| HDL cholesterol | rs114165349 | C | G | 0.023 | -0.081 | 0.006 | 3.90E-37 | 2.97E-04 | 119.922 |
| HDL cholesterol | rs3768321 | T | G | 0.197 | -0.045 | 0.002 | 1.50E-79 | 6.46E-04 | 261.301 |
| HDL cholesterol | rs12045101 | T | C | 0.241 | -0.015 | 0.002 | 6.70E-11 | 7.74E-05 | 31.283 |
| HDL cholesterol | rs2642438 | G | A | 0.703 | 0.028 | 0.002 | 2.10E-40 | 3.20E-04 | 129.133 |
| HDL cholesterol | rs76962725 | A | G | 0.037 | -0.028 | 0.005 | 4.60E-08 | 5.54E-05 | 22.378 |
| HDL cholesterol | rs185073199 | A | T | 0.010 | 0.064 | 0.010 | 2.40E-10 | 8.26E-05 | 33.375 |
| HDL cholesterol | rs75246752 | C | G | 0.013 | 0.049 | 0.008 | 5.40E-09 | 6.14E-05 | 24.811 |
| HDL cholesterol | rs1771582 | G | T | 0.557 | 0.013 | 0.002 | 6.00E-11 | 8.27E-05 | 33.406 |
| HDL cholesterol | rs4650994 | A | G | 0.532 | -0.018 | 0.002 | 2.10E-21 | 1.62E-04 | 65.590 |
| HDL cholesterol | rs61805075 | A | G | 0.329 | -0.026 | 0.002 | 6.00E-37 | 2.92E-04 | 117.943 |
| HDL cholesterol | rs141440048 | T | C | 0.018 | 0.044 | 0.008 | 7.80E-09 | 6.62E-05 | 26.758 |
| HDL cholesterol | rs2298632 | T | C | 0.498 | 0.014 | 0.002 | 9.70E-14 | 1.04E-04 | 42.031 |
| HDL cholesterol | rs771481 | A | T | 0.184 | 0.029 | 0.002 | 9.10E-32 | 2.49E-04 | 100.662 |
| HDL cholesterol | rs3747973 | G | A | 0.593 | 0.014 | 0.002 | 2.70E-13 | 9.69E-05 | 39.149 |
| HDL cholesterol | rs2298214 | A | C | 0.577 | -0.012 | 0.002 | 1.60E-10 | 7.52E-05 | 30.363 |
| HDL cholesterol | rs57760538 | G | T | 0.762 | 0.013 | 0.002 | 3.60E-08 | 5.70E-05 | 23.012 |
| HDL cholesterol | rs1168114 | G | A | 0.652 | 0.016 | 0.002 | 7.50E-15 | 1.09E-04 | 44.230 |
| HDL cholesterol | rs6693842 | C | T | 0.365 | 0.014 | 0.002 | 7.10E-12 | 8.65E-05 | 34.957 |
| HDL cholesterol | rs61748951 | A | C | 0.024 | -0.036 | 0.006 | 1.70E-08 | 6.13E-05 | 24.764 |
| HDL cholesterol | rs12740811 | G | A | 0.094 | -0.018 | 0.003 | 1.60E-08 | 5.79E-05 | 23.381 |
| HDL cholesterol | rs2516331 | A | C | 0.376 | 0.013 | 0.002 | 1.10E-10 | 7.55E-05 | 30.480 |
| HDL cholesterol | rs2281718 | T | A | 0.613 | 0.060 | 0.002 | 1.00E-200 | 1.69E-03 | 683.202 |
| HDL cholesterol | rs12740374 | T | G | 0.221 | 0.029 | 0.002 | 2.20E-36 | 2.87E-04 | 115.903 |
| HDL cholesterol | rs140584594 | G | A | 0.730 | 0.031 | 0.002 | 3.10E-48 | 3.81E-04 | 153.869 |
| HDL cholesterol | rs267738 | G | T | 0.219 | 0.021 | 0.002 | 9.90E-21 | 1.58E-04 | 63.678 |
| HDL cholesterol | rs12046972 | C | T | 0.564 | -0.015 | 0.002 | 1.00E-14 | 1.08E-04 | 43.643 |
| HDL cholesterol | rs557933 | C | A | 0.520 | 0.015 | 0.002 | 1.30E-15 | 1.16E-04 | 46.881 |
| HDL cholesterol | rs676210 | A | G | 0.205 | 0.059 | 0.002 | 4.50E-140 | 1.15E-03 | 463.430 |
| HDL cholesterol | rs7583067 | T | C | 0.240 | 0.015 | 0.002 | 6.60E-11 | 7.77E-05 | 31.400 |
| HDL cholesterol | rs17713879 | A | G | 0.365 | 0.014 | 0.002 | 2.00E-12 | 8.95E-05 | 36.145 |
| HDL cholesterol | rs13402475 | G | C | 0.815 | -0.025 | 0.002 | 1.90E-23 | 1.87E-04 | 75.638 |
| HDL cholesterol | rs2362541 | G | T | 0.508 | -0.011 | 0.002 | 1.10E-08 | 5.90E-05 | 23.845 |
| HDL cholesterol | rs13389219 | T | C | 0.393 | 0.028 | 0.002 | 4.50E-46 | 3.67E-04 | 148.223 |
| HDL cholesterol | rs2364723 | C | G | 0.319 | 0.012 | 0.002 | 3.40E-09 | 6.36E-05 | 25.685 |
| HDL cholesterol | rs12475332 | G | T | 0.261 | 0.013 | 0.002 | 2.50E-09 | 6.42E-05 | 25.923 |
| HDL cholesterol | rs907866 | A | G | 0.445 | -0.018 | 0.002 | 1.40E-21 | 1.66E-04 | 66.958 |
| HDL cholesterol | rs2723065 | G | A | 0.377 | 0.015 | 0.002 | 2.40E-14 | 1.05E-04 | 42.473 |
| HDL cholesterol | rs4599108 | T | C | 0.488 | 0.014 | 0.002 | 1.20E-12 | 9.33E-05 | 37.697 |
| HDL cholesterol | rs1446585 | G | A | 0.244 | 0.017 | 0.002 | 1.10E-14 | 1.04E-04 | 42.013 |
| HDL cholesterol | rs1083470 | A | G | 0.616 | 0.012 | 0.002 | 3.40E-09 | 6.35E-05 | 25.647 |
| HDL cholesterol | rs12998038 | T | C | 0.259 | 0.013 | 0.002 | 1.60E-09 | 6.67E-05 | 26.964 |
| HDL cholesterol | rs12986742 | C | T | 0.476 | -0.011 | 0.002 | 3.00E-08 | 5.61E-05 | 22.643 |
| HDL cholesterol | rs6705285 | T | G | 0.609 | 0.012 | 0.002 | 1.90E-09 | 6.32E-05 | 25.541 |
| HDL cholesterol | rs2196808 | C | T | 0.732 | 0.013 | 0.002 | 2.50E-09 | 6.49E-05 | 26.230 |
| HDL cholesterol | rs112350227 | T | C | 0.028 | -0.032 | 0.006 | 4.10E-08 | 5.48E-05 | 22.149 |
| HDL cholesterol | rs59037995 | A | T | 0.829 | -0.017 | 0.003 | 1.30E-11 | 8.40E-05 | 33.929 |
| HDL cholesterol | rs59104589 | T | C | 0.358 | 0.015 | 0.002 | 3.50E-14 | 1.04E-04 | 41.884 |
| HDL cholesterol | rs17326656 | T | G | 0.239 | -0.022 | 0.002 | 1.50E-23 | 1.82E-04 | 73.619 |
| HDL cholesterol | rs11688682 | C | G | 0.271 | 0.015 | 0.002 | 2.80E-11 | 8.58E-05 | 34.652 |
| HDL cholesterol | rs72926946 | A | C | 0.296 | -0.021 | 0.002 | 3.10E-23 | 1.78E-04 | 71.899 |
| HDL cholesterol | rs1047891 | A | C | 0.316 | -0.019 | 0.002 | 2.20E-20 | 1.54E-04 | 62.389 |
| HDL cholesterol | rs78058190 | A | G | 0.050 | -0.078 | 0.005 | 1.80E-57 | 5.88E-04 | 237.683 |
| HDL cholesterol | rs2943645 | T | C | 0.647 | -0.043 | 0.002 | 5.90E-106 | 8.64E-04 | 349.125 |
| HDL cholesterol | rs11456863 | A | T | 0.649 | 0.019 | 0.002 | 2.00E-21 | 1.65E-04 | 66.621 |
| HDL cholesterol | rs13097947 | C | T | 0.649 | 0.016 | 0.002 | 3.80E-15 | 1.16E-04 | 46.884 |
| HDL cholesterol | rs62246443 | C | T | 0.174 | -0.015 | 0.003 | 3.80E-09 | 6.29E-05 | 25.410 |
| HDL cholesterol | rs113851927 | G | GT | 0.687 | 0.011 | 0.002 | 4.30E-08 | 5.53E-05 | 22.335 |
| HDL cholesterol | rs4916426 | G | A | 0.745 | 0.014 | 0.002 | 2.00E-10 | 7.50E-05 | 30.289 |
| HDL cholesterol | rs7622114 | A | C | 0.581 | 0.012 | 0.002 | 2.50E-09 | 6.52E-05 | 26.323 |
| HDL cholesterol | rs2159607 | T | G | 0.811 | -0.024 | 0.002 | 7.20E-23 | 1.76E-04 | 71.051 |
| HDL cholesterol | rs72964564 | C | A | 0.250 | -0.013 | 0.002 | 1.20E-08 | 5.93E-05 | 23.970 |
| HDL cholesterol | rs6765484 | T | C | 0.473 | 0.023 | 0.002 | 3.90E-32 | 2.53E-04 | 102.159 |
| HDL cholesterol | rs4855582 | T | C | 0.430 | 0.011 | 0.002 | 6.50E-09 | 6.11E-05 | 24.681 |
| HDL cholesterol | rs1225053 | C | T | 0.264 | -0.015 | 0.002 | 3.90E-12 | 8.80E-05 | 35.542 |
| HDL cholesterol | rs62271373 | A | T | 0.060 | -0.041 | 0.004 | 2.50E-23 | 1.86E-04 | 75.335 |
| HDL cholesterol | rs13066793 | G | A | 0.090 | 0.022 | 0.003 | 3.30E-11 | 7.99E-05 | 32.288 |
| HDL cholesterol | rs6762415 | G | T | 0.535 | -0.011 | 0.002 | 1.20E-08 | 5.91E-05 | 23.862 |
| HDL cholesterol | rs77320712 | T | G | 0.235 | -0.013 | 0.002 | 1.90E-08 | 5.84E-05 | 23.608 |
| HDL cholesterol | rs10513801 | G | T | 0.137 | -0.030 | 0.003 | 6.70E-28 | 2.18E-04 | 87.982 |
| HDL cholesterol | rs13087167 | C | G | 0.633 | 0.017 | 0.002 | 2.90E-17 | 1.30E-04 | 52.637 |
| HDL cholesterol | rs830620 | T | C | 0.415 | 0.015 | 0.002 | 9.60E-15 | 1.09E-04 | 43.875 |
| HDL cholesterol | rs3732356 | T | G | 0.934 | -0.030 | 0.004 | 1.00E-14 | 1.11E-04 | 44.672 |
| HDL cholesterol | rs9647335 | T | A | 0.192 | 0.028 | 0.002 | 2.40E-30 | 2.39E-04 | 96.575 |
| HDL cholesterol | rs76247316 | C | T | 0.480 | -0.011 | 0.002 | 2.40E-09 | 6.47E-05 | 26.146 |
| HDL cholesterol | rs2268840 | C | T | 0.228 | 0.017 | 0.002 | 2.20E-14 | 1.06E-04 | 42.721 |
| HDL cholesterol | rs880674 | C | T | 0.142 | 0.015 | 0.003 | 4.40E-08 | 5.47E-05 | 22.107 |
| HDL cholesterol | rs2237035 | T | G | 0.386 | 0.014 | 0.002 | 1.20E-12 | 9.18E-05 | 37.085 |
| HDL cholesterol | rs1471251 | T | A | 0.398 | -0.019 | 0.002 | 3.60E-23 | 1.80E-04 | 72.619 |
| HDL cholesterol | rs1383732 | G | A | 0.150 | -0.015 | 0.003 | 1.40E-08 | 5.89E-05 | 23.803 |
| HDL cholesterol | rs10031010 | A | G | 0.185 | 0.014 | 0.002 | 2.10E-08 | 5.74E-05 | 23.189 |
| HDL cholesterol | rs13137144 | A | G | 0.461 | 0.016 | 0.002 | 1.50E-17 | 1.34E-04 | 54.054 |
| HDL cholesterol | rs71603401 | G | A | 0.137 | -0.015 | 0.003 | 3.20E-08 | 5.66E-05 | 22.846 |
| HDL cholesterol | rs73243877 | G | A | 0.168 | -0.025 | 0.003 | 2.50E-23 | 1.80E-04 | 72.720 |
| HDL cholesterol | rs2098918 | T | C | 0.455 | 0.012 | 0.002 | 5.80E-10 | 6.99E-05 | 28.229 |
| HDL cholesterol | rs71269068 | C | T | 0.654 | 0.016 | 0.002 | 3.70E-15 | 1.14E-04 | 46.103 |
| HDL cholesterol | rs13111599 | G | A | 0.737 | 0.013 | 0.002 | 3.50E-09 | 6.34E-05 | 25.614 |
| HDL cholesterol | rs1395221 | T | G | 0.398 | -0.011 | 0.002 | 1.00E-08 | 6.00E-05 | 24.226 |
| HDL cholesterol | rs1055582 | T | C | 0.505 | 0.014 | 0.002 | 2.10E-13 | 9.83E-05 | 39.728 |
| HDL cholesterol | rs13107325 | T | C | 0.075 | -0.080 | 0.004 | 7.00E-109 | 8.95E-04 | 361.876 |
| HDL cholesterol | rs62331150 | T | G | 0.205 | -0.013 | 0.002 | 4.30E-08 | 5.47E-05 | 22.104 |
| HDL cholesterol | rs13144151 | G | A | 0.850 | 0.018 | 0.003 | 3.80E-11 | 8.14E-05 | 32.894 |
| HDL cholesterol | rs1349852 | C | A | 0.475 | 0.011 | 0.002 | 5.80E-09 | 6.27E-05 | 25.332 |
| HDL cholesterol | rs6824451 | A | G | 0.464 | -0.020 | 0.002 | 1.00E-25 | 2.00E-04 | 80.744 |
| HDL cholesterol | rs7665587 | C | T | 0.422 | 0.014 | 0.002 | 4.70E-13 | 9.55E-05 | 38.566 |
| HDL cholesterol | rs4691379 | T | C | 0.319 | 0.012 | 0.002 | 6.20E-09 | 6.12E-05 | 24.734 |
| HDL cholesterol | rs10053349 | C | T | 0.390 | 0.012 | 0.002 | 7.90E-10 | 6.86E-05 | 27.717 |
| HDL cholesterol | rs2910949 | G | T | 0.355 | 0.013 | 0.002 | 2.00E-11 | 8.22E-05 | 33.212 |
| HDL cholesterol | rs11429307 | G | T | 0.809 | 0.031 | 0.002 | 9.70E-37 | 2.93E-04 | 118.525 |
| HDL cholesterol | rs2307111 | C | T | 0.395 | 0.019 | 0.002 | 2.10E-22 | 1.73E-04 | 69.742 |
| HDL cholesterol | rs2963468 | G | A | 0.235 | -0.020 | 0.002 | 3.60E-18 | 1.39E-04 | 56.224 |
| HDL cholesterol | rs7725218 | A | G | 0.338 | -0.012 | 0.002 | 1.20E-09 | 6.72E-05 | 27.131 |
| HDL cholesterol | rs67016280 | A | T | 0.548 | 0.011 | 0.002 | 8.90E-09 | 6.51E-05 | 26.305 |
| HDL cholesterol | rs34073570 | C | T | 0.601 | -0.011 | 0.002 | 1.30E-08 | 5.94E-05 | 23.992 |
| HDL cholesterol | rs1862205 | A | G | 0.405 | 0.011 | 0.002 | 5.80E-09 | 6.17E-05 | 24.937 |
| HDL cholesterol | rs454968 | C | T | 0.645 | 0.011 | 0.002 | 3.20E-08 | 5.58E-05 | 22.527 |
| HDL cholesterol | rs32578 | A | G | 0.309 | 0.013 | 0.002 | 1.10E-10 | 7.58E-05 | 30.624 |
| HDL cholesterol | rs286965 | C | T | 0.632 | -0.013 | 0.002 | 1.10E-11 | 8.37E-05 | 33.807 |
| HDL cholesterol | rs138354839 | A | C | 0.015 | -0.060 | 0.008 | 7.40E-13 | 1.08E-04 | 43.725 |
| HDL cholesterol | rs116006942 | A | G | 0.061 | -0.030 | 0.004 | 1.30E-13 | 1.02E-04 | 41.163 |
| HDL cholesterol | rs115912456 | G | A | 0.041 | 0.028 | 0.005 | 4.50E-09 | 6.24E-05 | 25.210 |
| HDL cholesterol | rs1045241 | T | C | 0.271 | 0.016 | 0.002 | 2.60E-14 | 1.07E-04 | 43.046 |
| HDL cholesterol | rs9327468 | A | C | 0.755 | -0.015 | 0.002 | 1.90E-11 | 8.20E-05 | 33.140 |
| HDL cholesterol | rs2339234 | A | G | 0.683 | -0.012 | 0.002 | 7.30E-09 | 6.13E-05 | 24.769 |
| HDL cholesterol | rs188502504 | C | T | 0.036 | -0.032 | 0.005 | 5.60E-10 | 7.04E-05 | 28.440 |
| HDL cholesterol | rs254562 | G | A | 0.409 | -0.011 | 0.002 | 3.40E-09 | 6.37E-05 | 25.722 |
| HDL cholesterol | rs2814982 | T | C | 0.102 | -0.028 | 0.003 | 1.60E-18 | 1.40E-04 | 56.458 |
| HDL cholesterol | rs6939861 | A | G | 0.262 | -0.014 | 0.002 | 9.70E-11 | 7.79E-05 | 31.481 |
| HDL cholesterol | rs968050 | T | C | 0.482 | 0.014 | 0.002 | 8.10E-13 | 9.32E-05 | 37.644 |
| HDL cholesterol | rs62428831 | C | T | 0.142 | 0.018 | 0.003 | 1.00E-10 | 7.71E-05 | 31.158 |
| HDL cholesterol | rs1281959 | G | C | 0.526 | 0.015 | 0.002 | 1.50E-14 | 1.07E-04 | 43.281 |
| HDL cholesterol | rs1240820 | A | G | 0.293 | 0.013 | 0.002 | 3.50E-10 | 7.20E-05 | 29.098 |
| HDL cholesterol | rs35224557 | GT | G | 0.597 | -0.012 | 0.002 | 2.00E-09 | 6.58E-05 | 26.581 |
| HDL cholesterol | rs36057735 | G | C | 0.199 | -0.030 | 0.002 | 1.00E-36 | 2.91E-04 | 117.628 |
| HDL cholesterol | rs11381821 | C | A | 0.531 | 0.019 | 0.002 | 4.60E-22 | 1.76E-04 | 71.040 |
| HDL cholesterol | rs635769 | C | T | 0.628 | 0.020 | 0.002 | 1.40E-23 | 1.81E-04 | 73.307 |
| HDL cholesterol | rs41272086 | A | G | 0.106 | -0.057 | 0.003 | 8.50E-75 | 6.09E-04 | 245.994 |
| HDL cholesterol | rs9465693 | A | C | 0.305 | -0.012 | 0.002 | 6.20E-09 | 6.21E-05 | 25.070 |
| HDL cholesterol | rs6934962 | T | C | 0.402 | 0.016 | 0.002 | 7.30E-17 | 1.26E-04 | 50.895 |
| HDL cholesterol | rs2750411 | G | T | 0.492 | -0.011 | 0.002 | 1.40E-08 | 5.87E-05 | 23.730 |
| HDL cholesterol | rs75479205 | G | A | 0.189 | 0.014 | 0.002 | 1.20E-08 | 5.93E-05 | 23.968 |
| HDL cholesterol | rs28746806 | C | A | 0.334 | 0.017 | 0.002 | 3.10E-16 | 1.24E-04 | 50.109 |
| HDL cholesterol | rs147627829 | A | G | 0.044 | -0.054 | 0.005 | 2.50E-30 | 2.40E-04 | 96.946 |
| HDL cholesterol | rs12205778 | A | G | 0.259 | 0.016 | 0.002 | 8.60E-14 | 1.01E-04 | 40.846 |
| HDL cholesterol | rs998584 | A | C | 0.483 | -0.034 | 0.002 | 1.10E-71 | 5.84E-04 | 235.952 |
| HDL cholesterol | rs2800710 | C | T | 0.518 | -0.020 | 0.002 | 1.80E-26 | 2.05E-04 | 82.870 |
| HDL cholesterol | rs9347737 | G | A | 0.428 | -0.013 | 0.002 | 4.40E-12 | 8.90E-05 | 35.940 |
| HDL cholesterol | rs6460894 | C | T | 0.339 | -0.012 | 0.002 | 1.60E-09 | 6.63E-05 | 26.803 |
| HDL cholesterol | rs17138358 | C | G | 0.398 | -0.027 | 0.002 | 2.20E-44 | 3.55E-04 | 143.593 |
| HDL cholesterol | rs201441 | G | T | 0.581 | -0.011 | 0.002 | 1.40E-08 | 5.86E-05 | 23.662 |
| HDL cholesterol | rs10233430 | C | T | 0.427 | -0.020 | 0.002 | 2.10E-26 | 2.06E-04 | 83.041 |
| HDL cholesterol | rs55935382 | A | C | 0.325 | 0.018 | 0.002 | 4.90E-18 | 1.37E-04 | 55.158 |
| HDL cholesterol | rs73151974 | T | C | 0.144 | -0.016 | 0.003 | 3.20E-09 | 6.46E-05 | 26.106 |
| HDL cholesterol | rs1534696 | A | C | 0.541 | 0.017 | 0.002 | 2.90E-18 | 1.38E-04 | 55.671 |
| HDL cholesterol | rs2726111 | G | A | 0.667 | -0.015 | 0.002 | 2.60E-13 | 9.74E-05 | 39.349 |
| HDL cholesterol | rs2098368 | T | C | 0.546 | -0.012 | 0.002 | 1.80E-09 | 6.67E-05 | 26.963 |
| HDL cholesterol | rs35493868 | G | C | 0.202 | 0.037 | 0.002 | 3.00E-55 | 4.47E-04 | 180.564 |
| HDL cholesterol | rs183906992 | C | T | 0.043 | 0.029 | 0.005 | 5.50E-10 | 7.10E-05 | 28.683 |
| HDL cholesterol | rs77605964 | A | G | 0.227 | 0.017 | 0.002 | 7.50E-14 | 1.02E-04 | 41.154 |
| HDL cholesterol | rs34940374 | A | G | 0.183 | -0.017 | 0.002 | 5.30E-12 | 8.68E-05 | 35.062 |
| HDL cholesterol | rs13235365 | T | C | 0.274 | 0.026 | 0.002 | 2.10E-33 | 2.65E-04 | 107.058 |
| HDL cholesterol | rs42125 | G | A | 0.023 | -0.040 | 0.007 | 1.30E-08 | 7.21E-05 | 29.110 |
| HDL cholesterol | rs12705595 | A | G | 0.373 | 0.011 | 0.002 | 2.50E-08 | 5.72E-05 | 23.103 |
| HDL cholesterol | rs34518086 | C | T | 0.463 | -0.031 | 0.002 | 3.80E-56 | 4.67E-04 | 188.816 |
| HDL cholesterol | rs7794796 | T | C | 0.332 | -0.017 | 0.002 | 1.20E-16 | 1.26E-04 | 51.078 |
| HDL cholesterol | rs9987289 | G | A | 0.909 | 0.087 | 0.003 | 9.10E-155 | 1.26E-03 | 511.165 |
| HDL cholesterol | rs75609851 | A | G | 0.010 | 0.170 | 0.010 | 1.40E-69 | 5.99E-04 | 241.924 |
| HDL cholesterol | rs140064750 | C | T | 0.025 | -0.044 | 0.006 | 3.70E-12 | 9.30E-05 | 37.566 |
| HDL cholesterol | rs75662196 | C | G | 0.028 | 0.068 | 0.006 | 6.70E-31 | 2.50E-04 | 101.105 |
| HDL cholesterol | rs61596977 | T | C | 0.140 | -0.016 | 0.003 | 3.10E-09 | 6.35E-05 | 25.663 |
| HDL cholesterol | rs2290866 | T | C | 0.253 | -0.012 | 0.002 | 4.60E-08 | 5.38E-05 | 21.713 |
| HDL cholesterol | rs7817574 | C | T | 0.185 | 0.033 | 0.002 | 3.50E-42 | 3.32E-04 | 134.296 |
| HDL cholesterol | rs330089 | C | T | 0.098 | 0.020 | 0.003 | 1.40E-10 | 7.41E-05 | 29.946 |
| HDL cholesterol | rs79153732 | T | C | 0.017 | -0.094 | 0.007 | 4.20E-38 | 3.02E-04 | 121.863 |
| HDL cholesterol | rs10504477 | C | T | 0.412 | -0.015 | 0.002 | 6.10E-15 | 1.10E-04 | 44.436 |
| HDL cholesterol | rs2247355 | T | C | 0.183 | 0.021 | 0.002 | 5.10E-17 | 1.27E-04 | 51.263 |
| HDL cholesterol | rs6469605 | T | C | 0.569 | 0.032 | 0.002 | 5.80E-61 | 4.91E-04 | 198.238 |
| HDL cholesterol | rs71571682 | T | C | 0.235 | 0.018 | 0.002 | 4.80E-15 | 1.16E-04 | 46.907 |
| HDL cholesterol | rs343 | A | C | 0.083 | 0.134 | 0.003 | 1.00E-200 | 2.73E-03 | 1104.291 |
| HDL cholesterol | rs308 | G | T | 0.021 | 0.127 | 0.007 | 1.10E-79 | 6.47E-04 | 261.523 |
| HDL cholesterol | rs13269725 | G | A | 0.078 | -0.026 | 0.004 | 1.90E-13 | 9.76E-05 | 39.410 |
| HDL cholesterol | rs1431659 | G | A | 0.728 | 0.013 | 0.002 | 2.30E-09 | 6.47E-05 | 26.150 |
| HDL cholesterol | rs4871603 | T | C | 0.653 | 0.036 | 0.002 | 5.20E-73 | 5.87E-04 | 237.313 |
| HDL cholesterol | rs61435086 | C | T | 0.012 | 0.089 | 0.009 | 3.70E-25 | 1.94E-04 | 78.434 |
| HDL cholesterol | rs7826177 | C | T | 0.635 | 0.011 | 0.002 | 1.40E-08 | 5.85E-05 | 23.645 |
| HDL cholesterol | rs72647336 | A | G | 0.057 | -0.044 | 0.004 | 2.60E-23 | 2.07E-04 | 83.759 |
| HDL cholesterol | rs4875043 | C | A | 0.217 | -0.015 | 0.002 | 1.30E-10 | 7.62E-05 | 30.764 |
| HDL cholesterol | rs10108282 | A | T | 0.206 | 0.017 | 0.002 | 6.30E-13 | 9.28E-05 | 37.495 |
| HDL cholesterol | rs75032664 | G | C | 0.013 | -0.055 | 0.009 | 1.30E-09 | 7.74E-05 | 31.261 |
| HDL cholesterol | rs142288236 | T | C | 0.015 | -0.078 | 0.008 | 1.60E-22 | 1.75E-04 | 70.603 |
| HDL cholesterol | rs80005209 | G | T | 0.030 | -0.144 | 0.006 | 2.50E-144 | 1.20E-03 | 483.305 |
| HDL cholesterol | rs4871624 | G | T | 0.287 | -0.020 | 0.002 | 3.90E-22 | 1.70E-04 | 68.879 |
| HDL cholesterol | rs2297409 | A | G | 0.195 | -0.033 | 0.002 | 1.00E-43 | 3.49E-04 | 140.953 |
| HDL cholesterol | rs1125873 | T | A | 0.492 | 0.016 | 0.002 | 9.60E-17 | 1.28E-04 | 51.531 |
| HDL cholesterol | rs10119644 | A | T | 0.494 | 0.013 | 0.002 | 1.80E-12 | 9.07E-05 | 36.633 |
| HDL cholesterol | rs1411432 | C | A | 0.186 | -0.014 | 0.002 | 1.30E-08 | 5.92E-05 | 23.933 |
| HDL cholesterol | rs74500135 | C | T | 0.010 | 0.067 | 0.010 | 2.30E-11 | 8.77E-05 | 35.442 |
| HDL cholesterol | rs1412234 | C | T | 0.327 | -0.012 | 0.002 | 3.90E-09 | 6.33E-05 | 25.562 |
| HDL cholesterol | rs7853377 | G | A | 0.216 | 0.015 | 0.002 | 4.70E-11 | 7.88E-05 | 31.821 |
| HDL cholesterol | rs2417125 | G | A | 0.284 | -0.013 | 0.002 | 5.20E-10 | 7.04E-05 | 28.421 |
| HDL cholesterol | rs532436 | A | G | 0.185 | 0.023 | 0.002 | 3.80E-21 | 1.61E-04 | 65.209 |
| HDL cholesterol | rs2520096 | G | A | 0.270 | 0.015 | 0.002 | 7.70E-12 | 8.55E-05 | 34.523 |
| HDL cholesterol | rs36036416 | C | T | 0.093 | 0.022 | 0.003 | 6.60E-12 | 8.56E-05 | 34.583 |
| HDL cholesterol | rs7036107 | G | A | 0.511 | -0.012 | 0.002 | 2.90E-10 | 7.50E-05 | 30.308 |
| HDL cholesterol | rs12686780 | T | C | 0.175 | -0.016 | 0.003 | 1.20E-10 | 7.55E-05 | 30.495 |
| HDL cholesterol | rs2740488 | C | A | 0.265 | -0.069 | 0.002 | 1.00E-200 | 1.84E-03 | 743.572 |
| HDL cholesterol | rs147772065 | C | G | 0.035 | 0.029 | 0.005 | 4.10E-08 | 5.57E-05 | 22.510 |
| HDL cholesterol | rs686030 | A | C | 0.859 | 0.050 | 0.003 | 6.50E-74 | 6.02E-04 | 243.308 |
| HDL cholesterol | rs2066714 | C | T | 0.129 | 0.047 | 0.003 | 3.40E-60 | 4.86E-04 | 196.212 |
| HDL cholesterol | rs11254464 | C | T | 0.423 | 0.013 | 0.002 | 3.90E-11 | 7.95E-05 | 32.100 |
| HDL cholesterol | rs34045894 | A | G | 0.156 | -0.017 | 0.003 | 4.60E-11 | 7.88E-05 | 31.844 |
| HDL cholesterol | rs4917675 | C | T | 0.255 | 0.015 | 0.002 | 3.40E-11 | 8.05E-05 | 32.514 |
| HDL cholesterol | rs2804894 | A | G | 0.735 | 0.017 | 0.002 | 2.30E-15 | 1.17E-04 | 47.154 |
| HDL cholesterol | rs7924036 | T | G | 0.503 | 0.014 | 0.002 | 3.60E-13 | 9.62E-05 | 38.845 |
| HDL cholesterol | rs703966 | A | G | 0.419 | 0.016 | 0.002 | 4.90E-16 | 1.20E-04 | 48.448 |
| HDL cholesterol | rs1970811 | C | T | 0.458 | -0.012 | 0.002 | 1.40E-09 | 6.69E-05 | 27.037 |
| HDL cholesterol | rs11009262 | T | G | 0.057 | -0.023 | 0.004 | 1.20E-08 | 5.88E-05 | 23.758 |
| HDL cholesterol | rs11239536 | A | T | 0.241 | 0.029 | 0.002 | 5.50E-38 | 3.02E-04 | 122.173 |
| HDL cholesterol | rs2068888 | A | G | 0.450 | 0.019 | 0.002 | 1.30E-23 | 1.82E-04 | 73.585 |
| HDL cholesterol | rs10786114 | T | C | 0.875 | 0.024 | 0.003 | 1.20E-16 | 1.25E-04 | 50.419 |
| HDL cholesterol | rs2792751 | C | T | 0.725 | -0.036 | 0.002 | 3.20E-64 | 5.20E-04 | 210.002 |
| HDL cholesterol | rs12781812 | T | G | 0.401 | 0.011 | 0.002 | 3.40E-08 | 5.52E-05 | 22.301 |
| HDL cholesterol | rs12411732 | A | G | 0.146 | -0.031 | 0.003 | 6.80E-29 | 2.33E-04 | 93.976 |
| HDL cholesterol | rs61884005 | G | C | 0.121 | 0.016 | 0.003 | 2.40E-08 | 5.70E-05 | 23.017 |
| HDL cholesterol | rs57512892 | T | C | 0.596 | 0.021 | 0.002 | 9.60E-21 | 2.04E-04 | 82.546 |
| HDL cholesterol | rs559355 | T | A | 0.157 | -0.035 | 0.003 | 9.00E-41 | 3.23E-04 | 130.712 |
| HDL cholesterol | rs117847213 | G | A | 0.041 | 0.030 | 0.005 | 1.20E-09 | 6.84E-05 | 27.634 |
| HDL cholesterol | rs59781045 | T | C | 0.068 | 0.074 | 0.004 | 4.50E-85 | 6.96E-04 | 281.297 |
| HDL cholesterol | rs17309930 | A | C | 0.205 | -0.022 | 0.002 | 1.50E-20 | 1.56E-04 | 63.121 |
| HDL cholesterol | rs2302263 | T | C | 0.089 | -0.036 | 0.003 | 1.60E-27 | 2.15E-04 | 86.715 |
| HDL cholesterol | rs11021232 | C | T | 0.181 | -0.017 | 0.002 | 1.70E-11 | 8.30E-05 | 33.548 |
| HDL cholesterol | rs964184 | C | G | 0.866 | 0.105 | 0.003 | 1.00E-200 | 2.57E-03 | 1042.562 |
| HDL cholesterol | rs141469619 | G | A | 0.010 | -0.203 | 0.010 | 8.70E-89 | 8.10E-04 | 327.446 |
| HDL cholesterol | rs4614 | G | A | 0.406 | -0.018 | 0.002 | 3.80E-20 | 1.53E-04 | 61.901 |
| HDL cholesterol | rs16928809 | A | G | 0.093 | -0.026 | 0.003 | 1.70E-15 | 1.16E-04 | 47.050 |
| HDL cholesterol | rs12575456 | A | G | 0.322 | 0.045 | 0.002 | 1.40E-106 | 8.68E-04 | 350.831 |
| HDL cholesterol | rs117762989 | T | C | 0.043 | -0.027 | 0.005 | 9.30E-09 | 5.95E-05 | 24.056 |
| HDL cholesterol | rs4930352 | T | G | 0.494 | 0.016 | 0.002 | 7.30E-17 | 1.31E-04 | 52.815 |
| HDL cholesterol | rs689183 | T | G | 0.751 | -0.016 | 0.002 | 7.70E-13 | 9.36E-05 | 37.818 |
| HDL cholesterol | rs117291242 | T | C | 0.037 | -0.032 | 0.005 | 4.50E-10 | 7.03E-05 | 28.407 |
| HDL cholesterol | rs79634051 | C | G | 0.028 | 0.039 | 0.006 | 9.00E-12 | 8.42E-05 | 34.023 |
| HDL cholesterol | rs564832 | C | T | 0.315 | -0.012 | 0.002 | 1.20E-08 | 5.92E-05 | 23.897 |
| HDL cholesterol | rs34389751 | T | G | 0.365 | -0.011 | 0.002 | 1.00E-08 | 5.98E-05 | 24.150 |
| HDL cholesterol | rs10750766 | A | C | 0.710 | -0.019 | 0.002 | 9.20E-19 | 1.42E-04 | 57.363 |
| HDL cholesterol | rs2155220 | T | C | 0.438 | -0.011 | 0.002 | 3.80E-08 | 5.45E-05 | 22.011 |
| HDL cholesterol | rs11218738 | A | G | 0.249 | 0.023 | 0.002 | 1.40E-26 | 2.06E-04 | 83.200 |
| HDL cholesterol | rs174566 | G | A | 0.350 | -0.056 | 0.002 | 1.20E-174 | 1.44E-03 | 581.881 |
| HDL cholesterol | rs10774439 | A | G | 0.815 | 0.021 | 0.002 | 1.40E-16 | 1.27E-04 | 51.285 |
| HDL cholesterol | rs11045171 | G | A | 0.198 | 0.028 | 0.002 | 4.30E-32 | 2.55E-04 | 103.097 |
| HDL cholesterol | rs7316878 | C | T | 0.434 | 0.012 | 0.002 | 1.20E-08 | 6.50E-05 | 26.268 |
| HDL cholesterol | rs2645979 | A | G | 0.357 | 0.011 | 0.002 | 8.70E-09 | 6.00E-05 | 24.233 |
| HDL cholesterol | rs12229011 | T | C | 0.097 | -0.026 | 0.003 | 7.80E-16 | 1.19E-04 | 48.022 |
| HDL cholesterol | rs11614202 | G | A | 0.842 | 0.023 | 0.003 | 3.00E-18 | 1.38E-04 | 55.759 |
| HDL cholesterol | rs7488780 | C | G | 0.204 | 0.015 | 0.002 | 8.50E-10 | 6.88E-05 | 27.786 |
| HDL cholesterol | rs200573817 | A | G | 0.755 | 0.015 | 0.002 | 4.60E-11 | 7.87E-05 | 31.798 |
| HDL cholesterol | rs58298943 | T | C | 0.084 | 0.020 | 0.003 | 3.80E-09 | 6.30E-05 | 25.450 |
| HDL cholesterol | rs61352607 | T | G | 0.241 | 0.031 | 0.002 | 4.40E-43 | 3.43E-04 | 138.462 |
| HDL cholesterol | rs3184504 | C | T | 0.517 | 0.027 | 0.002 | 3.90E-44 | 3.52E-04 | 142.128 |
| HDL cholesterol | rs3924313 | A | G | 0.322 | -0.024 | 0.002 | 1.60E-31 | 2.50E-04 | 100.950 |
| HDL cholesterol | rs58123204 | G | A | 0.153 | -0.018 | 0.003 | 2.90E-12 | 8.85E-05 | 35.755 |
| HDL cholesterol | rs202079372 | T | C | 0.496 | -0.011 | 0.002 | 1.10E-08 | 6.01E-05 | 24.267 |
| HDL cholesterol | rs2111216 | G | A | 0.594 | 0.021 | 0.002 | 5.00E-28 | 2.19E-04 | 88.664 |
| HDL cholesterol | rs71445274 | T | A | 0.336 | 0.035 | 0.002 | 2.50E-67 | 5.47E-04 | 220.887 |
| HDL cholesterol | rs921919 | A | G | 0.669 | -0.042 | 0.002 | 1.40E-90 | 7.69E-04 | 310.721 |
| HDL cholesterol | rs113740515 | A | G | 0.209 | 0.038 | 0.002 | 1.20E-58 | 4.73E-04 | 190.976 |
| HDL cholesterol | rs7305678 | G | T | 0.866 | -0.017 | 0.003 | 4.20E-09 | 6.40E-05 | 25.864 |
| HDL cholesterol | rs11171710 | A | G | 0.448 | -0.011 | 0.002 | 2.80E-09 | 6.51E-05 | 26.285 |
| HDL cholesterol | rs111363680 | T | C | 0.031 | 0.033 | 0.006 | 3.10E-08 | 6.54E-05 | 26.426 |
| HDL cholesterol | rs9604045 | T | G | 0.254 | 0.018 | 0.002 | 2.00E-14 | 1.16E-04 | 46.915 |
| HDL cholesterol | rs150861794 | T | C | 0.018 | -0.045 | 0.008 | 4.00E-09 | 7.18E-05 | 29.000 |
| HDL cholesterol | rs76428106 | C | T | 0.013 | -0.062 | 0.009 | 1.20E-12 | 9.97E-05 | 40.294 |
| HDL cholesterol | rs117230571 | G | A | 0.077 | -0.027 | 0.004 | 2.20E-13 | 1.01E-04 | 40.737 |
| HDL cholesterol | rs549058 | T | G | 0.120 | 0.017 | 0.003 | 5.70E-09 | 6.20E-05 | 25.033 |
| HDL cholesterol | rs367677 | G | A | 0.240 | 0.017 | 0.002 | 1.20E-13 | 1.01E-04 | 40.954 |
| HDL cholesterol | rs8014289 | G | A | 0.562 | 0.015 | 0.002 | 4.60E-15 | 1.12E-04 | 45.371 |
| HDL cholesterol | rs17124112 | A | C | 0.080 | -0.021 | 0.004 | 3.80E-09 | 6.36E-05 | 25.701 |
| HDL cholesterol | rs1760940 | C | A | 0.247 | 0.012 | 0.002 | 3.90E-08 | 5.52E-05 | 22.290 |
| HDL cholesterol | rs4899251 | T | C | 0.942 | -0.023 | 0.004 | 2.10E-08 | 5.88E-05 | 23.764 |
| HDL cholesterol | rs8007841 | C | T | 0.657 | -0.013 | 0.002 | 1.80E-10 | 7.47E-05 | 30.174 |
| HDL cholesterol | rs7158166 | C | T | 0.594 | 0.014 | 0.002 | 5.10E-13 | 9.58E-05 | 38.705 |
| HDL cholesterol | rs1955512 | A | G | 0.576 | 0.011 | 0.002 | 2.40E-08 | 5.91E-05 | 23.872 |
| HDL cholesterol | rs13379043 | C | T | 0.280 | 0.020 | 0.002 | 4.70E-20 | 1.59E-04 | 64.414 |
| HDL cholesterol | rs2498786 | G | C | 0.616 | -0.025 | 0.002 | 2.60E-38 | 3.07E-04 | 124.051 |
| HDL cholesterol | rs10162642 | A | G | 0.212 | -0.048 | 0.002 | 4.60E-93 | 7.66E-04 | 309.845 |
| HDL cholesterol | rs35980001 | G | C | 0.787 | -0.119 | 0.002 | 1.00E-200 | 4.74E-03 | 1923.246 |
| HDL cholesterol | rs1270076 | G | A | 0.777 | 0.014 | 0.002 | 3.50E-10 | 7.14E-05 | 28.824 |
| HDL cholesterol | rs147460434 | A | T | 0.818 | -0.015 | 0.002 | 1.20E-09 | 6.76E-05 | 27.303 |
| HDL cholesterol | rs4074448 | A | G | 0.589 | 0.015 | 0.002 | 1.30E-14 | 1.11E-04 | 44.772 |
| HDL cholesterol | rs7170463 | G | A | 0.311 | 0.019 | 0.002 | 4.20E-20 | 1.53E-04 | 61.966 |
| HDL cholesterol | rs150844304 | C | A | 0.026 | -0.091 | 0.006 | 1.40E-51 | 4.14E-04 | 167.207 |
| HDL cholesterol | rs11631178 | C | T | 0.104 | 0.021 | 0.003 | 1.30E-11 | 8.31E-05 | 33.581 |
| HDL cholesterol | rs28362901 | A | C | 0.088 | -0.024 | 0.003 | 1.20E-12 | 9.19E-05 | 37.131 |
| HDL cholesterol | rs28510484 | C | G | 0.170 | -0.015 | 0.003 | 1.20E-09 | 6.75E-05 | 27.261 |
| HDL cholesterol | rs1601934 | A | G | 0.687 | -0.101 | 0.002 | 1.00E-200 | 4.39E-03 | 1779.089 |
| HDL cholesterol | rs3814883 | T | C | 0.482 | -0.015 | 0.002 | 5.90E-16 | 1.17E-04 | 47.248 |
| HDL cholesterol | rs4784709 | A | T | 0.959 | -0.075 | 0.005 | 3.00E-55 | 4.37E-04 | 176.787 |
| HDL cholesterol | rs12921195 | A | C | 0.140 | -0.017 | 0.003 | 3.20E-09 | 6.69E-05 | 27.006 |
| HDL cholesterol | rs201639483 | T | A | 0.596 | 0.011 | 0.002 | 2.40E-08 | 5.69E-05 | 22.975 |
| HDL cholesterol | rs112233856 | G | A | 0.029 | -0.053 | 0.006 | 8.00E-20 | 1.55E-04 | 62.488 |
| HDL cholesterol | rs55781197 | G | A | 0.115 | 0.059 | 0.003 | 4.00E-88 | 7.02E-04 | 283.922 |
| HDL cholesterol | rs12928099 | A | C | 0.296 | 0.021 | 0.002 | 3.70E-25 | 1.91E-04 | 77.320 |
| HDL cholesterol | rs113966472 | A | G | 0.032 | 0.032 | 0.006 | 4.80E-09 | 6.61E-05 | 26.722 |
| HDL cholesterol | rs7186799 | C | A | 0.437 | -0.022 | 0.002 | 5.20E-31 | 2.39E-04 | 96.453 |
| HDL cholesterol | rs71647892 | C | T | 0.012 | -0.063 | 0.009 | 5.20E-13 | 9.27E-05 | 37.451 |
| HDL cholesterol | rs2925979 | C | T | 0.700 | 0.037 | 0.002 | 1.60E-73 | 5.84E-04 | 236.165 |
| HDL cholesterol | rs9933509 | C | T | 0.414 | -0.014 | 0.002 | 2.40E-13 | 9.53E-05 | 38.482 |
| HDL cholesterol | rs75152587 | T | G | 0.013 | -0.094 | 0.008 | 1.10E-28 | 2.24E-04 | 90.613 |
| HDL cholesterol | rs9989419 | G | A | 0.606 | 0.144 | 0.002 | 1.00E-200 | 9.87E-03 | 4026.828 |
| HDL cholesterol | rs4330777 | A | G | 0.475 | -0.020 | 0.002 | 1.30E-25 | 1.95E-04 | 78.742 |
| HDL cholesterol | rs140164052 | A | G | 0.032 | -0.042 | 0.005 | 1.20E-14 | 1.07E-04 | 43.333 |
| HDL cholesterol | rs12926854 | G | A | 0.270 | 0.012 | 0.002 | 9.50E-09 | 5.91E-05 | 23.875 |
| HDL cholesterol | rs11640494 | A | G | 0.457 | -0.015 | 0.002 | 6.50E-16 | 1.17E-04 | 47.231 |
| HDL cholesterol | rs79600951 | G | C | 0.092 | -0.107 | 0.003 | 1.00E-200 | 1.91E-03 | 771.603 |
| HDL cholesterol | rs116857878 | T | C | 0.020 | 0.048 | 0.007 | 4.50E-12 | 9.30E-05 | 37.573 |
| HDL cholesterol | rs9916613 | A | T | 0.356 | -0.013 | 0.002 | 2.30E-11 | 8.13E-05 | 32.845 |
| HDL cholesterol | rs34138141 | T | G | 0.281 | -0.018 | 0.002 | 1.60E-16 | 1.24E-04 | 50.138 |
| HDL cholesterol | rs145947882 | C | A | 0.026 | -0.165 | 0.006 | 4.70E-161 | 1.38E-03 | 558.197 |
| HDL cholesterol | rs112001035 | A | G | 0.060 | -0.047 | 0.004 | 3.30E-30 | 2.47E-04 | 99.860 |
| HDL cholesterol | rs7218647 | A | G | 0.559 | 0.011 | 0.002 | 1.40E-08 | 5.88E-05 | 23.754 |
| HDL cholesterol | rs141062196 | A | G | 0.194 | -0.019 | 0.002 | 5.20E-15 | 1.11E-04 | 44.992 |
| HDL cholesterol | rs4969141 | T | C | 0.490 | 0.030 | 0.002 | 3.20E-54 | 4.39E-04 | 177.407 |
| HDL cholesterol | rs3027167 | T | C | 0.682 | -0.012 | 0.002 | 1.40E-09 | 6.70E-05 | 27.053 |
| HDL cholesterol | rs9904004 | G | A | 0.061 | -0.030 | 0.004 | 4.10E-14 | 1.05E-04 | 42.237 |
| HDL cholesterol | rs2271308 | C | T | 0.733 | 0.028 | 0.002 | 3.00E-38 | 3.04E-04 | 122.929 |
| HDL cholesterol | rs2586116 | G | C | 0.259 | -0.016 | 0.002 | 7.10E-14 | 1.02E-04 | 41.030 |
| HDL cholesterol | rs3794752 | C | T | 0.281 | 0.013 | 0.002 | 2.00E-09 | 6.65E-05 | 26.859 |
| HDL cholesterol | rs8081548 | A | T | 0.659 | 0.018 | 0.002 | 6.90E-20 | 1.53E-04 | 61.850 |
| HDL cholesterol | rs35566853 | C | T | 0.783 | 0.013 | 0.002 | 1.30E-08 | 6.11E-05 | 24.699 |
| HDL cholesterol | rs56017932 | C | G | 0.200 | 0.015 | 0.003 | 8.20E-09 | 7.68E-05 | 31.021 |
| HDL cholesterol | rs73455693 | A | G | 0.037 | 0.028 | 0.005 | 3.90E-08 | 5.65E-05 | 22.810 |
| HDL cholesterol | rs2435307 | T | C | 0.486 | 0.016 | 0.002 | 1.50E-17 | 1.32E-04 | 53.475 |
| HDL cholesterol | rs77960347 | G | A | 0.013 | 0.291 | 0.008 | 1.00E-200 | 2.23E-03 | 902.847 |
| HDL cholesterol | rs8086351 | G | C | 0.824 | 0.084 | 0.003 | 1.00E-200 | 2.04E-03 | 826.791 |
| HDL cholesterol | rs11664369 | T | C | 0.267 | -0.023 | 0.002 | 1.80E-26 | 2.07E-04 | 83.508 |
| HDL cholesterol | rs983663 | G | A | 0.252 | -0.014 | 0.002 | 7.70E-11 | 7.77E-05 | 31.382 |
| HDL cholesterol | rs150237291 | C | T | 0.022 | 0.045 | 0.007 | 5.10E-12 | 8.75E-05 | 35.364 |
| HDL cholesterol | rs680321 | C | T | 0.457 | 0.011 | 0.002 | 1.50E-08 | 5.84E-05 | 23.606 |
| HDL cholesterol | rs2298624 | T | C | 0.133 | 0.030 | 0.003 | 1.10E-26 | 2.07E-04 | 83.811 |
| HDL cholesterol | rs4804101 | T | G | 0.439 | -0.014 | 0.002 | 5.30E-13 | 9.52E-05 | 38.469 |
| HDL cholesterol | rs3745683 | A | G | 0.075 | -0.055 | 0.004 | 3.20E-51 | 4.12E-04 | 166.627 |
| HDL cholesterol | rs429358 | C | T | 0.154 | -0.076 | 0.003 | 8.71E-181 | 1.49E-03 | 604.389 |
| HDL cholesterol | rs12462109 | T | C | 0.287 | -0.016 | 0.002 | 3.80E-14 | 1.04E-04 | 42.088 |
| HDL cholesterol | rs367070 | G | A | 0.226 | 0.042 | 0.002 | 1.30E-74 | 6.10E-04 | 246.675 |
| HDL cholesterol | rs554146 | C | A | 0.705 | 0.013 | 0.002 | 1.80E-09 | 6.74E-05 | 27.212 |
| HDL cholesterol | rs62117487 | G | A | 0.057 | 0.046 | 0.004 | 1.10E-28 | 2.25E-04 | 90.962 |
| HDL cholesterol | rs116843064 | A | G | 0.019 | 0.206 | 0.007 | 3.40E-195 | 1.61E-03 | 651.961 |
| HDL cholesterol | rs62102718 | T | A | 0.286 | -0.024 | 0.002 | 3.40E-29 | 2.30E-04 | 92.728 |
| HDL cholesterol | rs4803773 | G | A | 0.499 | 0.040 | 0.002 | 8.70E-90 | 8.03E-04 | 324.780 |
| HDL cholesterol | rs7251640 | C | T | 0.194 | 0.014 | 0.002 | 5.10E-09 | 6.30E-05 | 25.440 |
| HDL cholesterol | rs2965169 | C | A | 0.389 | 0.012 | 0.002 | 6.30E-10 | 6.94E-05 | 28.030 |
| HDL cholesterol | rs144311893 | T | C | 0.022 | 0.081 | 0.007 | 1.10E-33 | 2.83E-04 | 114.405 |
| HDL cholesterol | rs1132274 | A | C | 0.154 | -0.022 | 0.003 | 1.60E-16 | 1.24E-04 | 49.947 |
| HDL cholesterol | rs6018652 | A | G | 0.793 | 0.026 | 0.002 | 4.60E-28 | 2.21E-04 | 89.201 |
| HDL cholesterol | rs6123685 | A | G | 0.255 | 0.016 | 0.002 | 3.00E-13 | 9.69E-05 | 39.162 |
| HDL cholesterol | rs144033177 | C | A | 0.016 | -0.056 | 0.008 | 1.40E-12 | 9.62E-05 | 38.851 |
| HDL cholesterol | rs150224153 | T | C | 0.030 | -0.093 | 0.006 | 3.10E-59 | 5.03E-04 | 203.267 |
| HDL cholesterol | rs77767539 | A | G | 0.014 | 0.045 | 0.008 | 3.60E-08 | 5.78E-05 | 23.331 |
| HDL cholesterol | rs6066148 | C | G | 0.260 | 0.013 | 0.002 | 1.00E-09 | 6.82E-05 | 27.546 |
| HDL cholesterol | rs6059958 | T | C | 0.171 | 0.015 | 0.003 | 9.70E-09 | 6.08E-05 | 24.565 |
| HDL cholesterol | rs6142206 | A | G | 0.421 | -0.016 | 0.002 | 8.30E-17 | 1.27E-04 | 51.110 |
| HDL cholesterol | rs6073958 | C | T | 0.199 | -0.061 | 0.002 | 4.10E-143 | 1.18E-03 | 478.285 |
| HDL cholesterol | rs6075860 | A | G | 0.565 | -0.013 | 0.002 | 3.10E-11 | 8.05E-05 | 32.526 |
| HDL cholesterol | rs76602912 | C | T | 0.024 | -0.043 | 0.006 | 4.00E-12 | 8.77E-05 | 35.419 |
| HDL cholesterol | rs407133 | C | G | 0.559 | -0.011 | 0.002 | 4.60E-08 | 5.50E-05 | 22.220 |
| HDL cholesterol | rs235314 | T | C | 0.532 | -0.018 | 0.002 | 2.40E-20 | 1.57E-04 | 63.326 |
| HDL cholesterol | rs7281183 | A | G | 0.737 | -0.013 | 0.002 | 6.30E-09 | 6.27E-05 | 25.329 |
| HDL cholesterol | rs2236464 | C | T | 0.212 | -0.016 | 0.002 | 3.70E-11 | 8.12E-05 | 32.789 |
| HDL cholesterol | rs3746915 | G | A | 0.581 | 0.011 | 0.002 | 2.10E-08 | 5.77E-05 | 23.289 |
| HDL cholesterol | rs2256609 | G | A | 0.189 | -0.033 | 0.002 | 2.20E-41 | 3.32E-04 | 134.113 |
| HDL cholesterol | rs11704977 | T | C | 0.150 | 0.015 | 0.003 | 4.30E-08 | 5.53E-05 | 22.336 |
| HDL cholesterol | rs133015 | G | C | 0.440 | 0.021 | 0.002 | 1.20E-26 | 2.09E-04 | 84.489 |
| HDL cholesterol | rs9622830 | G | C | 0.354 | -0.016 | 0.002 | 2.60E-16 | 1.23E-04 | 49.601 |
| HDL cholesterol | rs460428 | C | T | 0.231 | -0.014 | 0.002 | 2.50E-10 | 7.30E-05 | 29.509 |
| LDL cholesterol | rs115458560 | C | T | 0.019 | -0.050 | 0.008 | 6.40E-11 | 9.23E-05 | 40.680 |
| LDL cholesterol | rs6667939 | T | C | 0.719 | 0.015 | 0.002 | 5.00E-11 | 9.47E-05 | 41.738 |
| LDL cholesterol | rs12078100 | G | C | 0.623 | 0.013 | 0.002 | 1.00E-09 | 8.08E-05 | 35.582 |
| LDL cholesterol | rs114165349 | C | G | 0.023 | 0.056 | 0.007 | 6.20E-16 | 1.43E-04 | 63.064 |
| LDL cholesterol | rs140584594 | G | A | 0.730 | -0.014 | 0.002 | 7.70E-10 | 8.09E-05 | 35.659 |
| LDL cholesterol | rs1183851 | C | T | 0.396 | 0.024 | 0.002 | 4.50E-30 | 2.84E-04 | 125.217 |
| LDL cholesterol | rs6680227 | A | G | 0.035 | -0.075 | 0.006 | 4.30E-40 | 3.79E-04 | 167.016 |
| LDL cholesterol | rs2642438 | G | A | 0.703 | 0.025 | 0.002 | 8.00E-29 | 2.68E-04 | 118.066 |
| LDL cholesterol | rs28631087 | C | T | 0.213 | -0.016 | 0.003 | 2.00E-10 | 8.78E-05 | 38.683 |
| LDL cholesterol | rs880315 | C | T | 0.340 | -0.015 | 0.002 | 5.30E-12 | 1.04E-04 | 45.836 |
| LDL cholesterol | rs55637835 | T | C | 0.121 | -0.019 | 0.003 | 8.40E-09 | 7.44E-05 | 32.790 |
| LDL cholesterol | rs11591147 | T | G | 0.017 | -0.348 | 0.008 | 1.00E-200 | 4.17E-03 | 1843.822 |
| LDL cholesterol | rs472495 | T | G | 0.649 | 0.043 | 0.002 | 7.30E-85 | 8.26E-04 | 364.123 |
| LDL cholesterol | rs4970834 | T | C | 0.187 | -0.105 | 0.003 | 1.00E-200 | 3.37E-03 | 1488.184 |
| LDL cholesterol | rs556107 | T | C | 0.523 | 0.035 | 0.002 | 1.20E-63 | 6.17E-04 | 272.054 |
| LDL cholesterol | rs34596921 | T | A | 0.580 | 0.027 | 0.002 | 2.20E-36 | 3.51E-04 | 154.598 |
| LDL cholesterol | rs1556562 | T | G | 0.790 | 0.019 | 0.002 | 2.10E-14 | 1.20E-04 | 52.907 |
| LDL cholesterol | rs10910476 | T | C | 0.555 | 0.012 | 0.002 | 3.70E-09 | 7.59E-05 | 33.426 |
| LDL cholesterol | rs13020929 | A | G | 0.457 | 0.015 | 0.002 | 3.40E-12 | 1.06E-04 | 46.508 |
| LDL cholesterol | rs12471768 | C | T | 0.704 | 0.014 | 0.002 | 2.50E-09 | 7.71E-05 | 33.954 |
| LDL cholesterol | rs6732741 | A | T | 0.119 | -0.028 | 0.003 | 2.90E-18 | 1.66E-04 | 73.114 |
| LDL cholesterol | rs6709904 | G | A | 0.113 | -0.043 | 0.003 | 1.10E-39 | 3.77E-04 | 166.184 |
| LDL cholesterol | rs4666384 | G | A | 0.679 | -0.017 | 0.002 | 1.10E-13 | 1.21E-04 | 53.143 |
| LDL cholesterol | rs934197 | A | G | 0.335 | 0.083 | 0.002 | 1.00E-200 | 3.09E-03 | 1364.966 |
| LDL cholesterol | rs7562734 | C | G | 0.322 | -0.020 | 0.002 | 1.80E-19 | 1.77E-04 | 77.863 |
| LDL cholesterol | rs150474434 | A | G | 0.101 | -0.035 | 0.003 | 1.30E-23 | 2.20E-04 | 96.997 |
| LDL cholesterol | rs17050272 | A | G | 0.409 | -0.021 | 0.002 | 2.50E-22 | 2.04E-04 | 90.062 |
| LDL cholesterol | rs7569317 | C | T | 0.531 | 0.018 | 0.002 | 8.90E-18 | 1.59E-04 | 70.174 |
| LDL cholesterol | rs56236159 | G | T | 0.131 | 0.018 | 0.003 | 8.80E-09 | 7.17E-05 | 31.575 |
| LDL cholesterol | rs4954192 | T | C | 0.373 | 0.015 | 0.002 | 6.70E-12 | 1.01E-04 | 44.330 |
| LDL cholesterol | rs2287622 | G | A | 0.603 | -0.021 | 0.002 | 2.90E-23 | 2.14E-04 | 94.453 |
| LDL cholesterol | rs1250258 | T | C | 0.737 | 0.014 | 0.002 | 6.00E-09 | 7.36E-05 | 32.434 |
| LDL cholesterol | rs11568318 | A | C | 0.066 | 0.026 | 0.004 | 7.80E-10 | 8.17E-05 | 35.987 |
| LDL cholesterol | rs1260326 | C | T | 0.604 | -0.035 | 0.002 | 5.10E-60 | 5.77E-04 | 254.239 |
| LDL cholesterol | rs6544713 | C | T | 0.677 | -0.054 | 0.002 | 5.00E-129 | 1.26E-03 | 556.736 |
| LDL cholesterol | rs148150904 | G | T | 0.190 | 0.016 | 0.003 | 2.50E-09 | 7.69E-05 | 33.871 |
| LDL cholesterol | rs13076933 | G | T | 0.259 | -0.021 | 0.002 | 1.60E-18 | 1.69E-04 | 74.627 |
| LDL cholesterol | rs9832727 | G | C | 0.340 | -0.015 | 0.002 | 2.10E-11 | 9.77E-05 | 43.058 |
| LDL cholesterol | rs9834932 | G | A | 0.089 | -0.032 | 0.004 | 1.50E-18 | 1.68E-04 | 74.033 |
| LDL cholesterol | rs71311871 | G | A | 0.083 | -0.028 | 0.004 | 7.50E-14 | 1.22E-04 | 53.773 |
| LDL cholesterol | rs5849920 | G | T | 0.364 | -0.014 | 0.002 | 3.10E-10 | 9.29E-05 | 40.941 |
| LDL cholesterol | rs9289196 | C | T | 0.174 | 0.017 | 0.003 | 3.00E-10 | 8.66E-05 | 38.175 |
| LDL cholesterol | rs113177823 | A | G | 0.054 | -0.041 | 0.005 | 1.00E-18 | 1.74E-04 | 76.498 |
| LDL cholesterol | rs3732359 | A | G | 0.780 | -0.017 | 0.003 | 5.70E-12 | 1.03E-04 | 45.529 |
| LDL cholesterol | rs13121616 | G | A | 0.694 | 0.013 | 0.002 | 1.30E-08 | 7.02E-05 | 30.927 |
| LDL cholesterol | rs9884390 | C | T | 0.234 | 0.025 | 0.002 | 5.90E-24 | 2.27E-04 | 99.944 |
| LDL cholesterol | rs1229984 | C | T | 0.973 | 0.053 | 0.006 | 1.00E-16 | 1.46E-04 | 64.465 |
| LDL cholesterol | rs990619 | G | C | 0.524 | -0.012 | 0.002 | 1.50E-08 | 6.95E-05 | 30.612 |
| LDL cholesterol | rs13108218 | G | A | 0.615 | -0.018 | 0.002 | 1.80E-16 | 1.50E-04 | 66.022 |
| LDL cholesterol | rs11099097 | T | C | 0.291 | -0.018 | 0.002 | 2.80E-15 | 1.37E-04 | 60.181 |
| LDL cholesterol | rs13107325 | T | C | 0.075 | -0.025 | 0.004 | 3.80E-10 | 8.55E-05 | 37.650 |
| LDL cholesterol | rs7707394 | A | G | 0.357 | 0.040 | 0.002 | 2.40E-77 | 7.51E-04 | 331.281 |
| LDL cholesterol | rs12916 | C | T | 0.401 | 0.062 | 0.002 | 1.70E-187 | 1.85E-03 | 817.820 |
| LDL cholesterol | rs146433259 | T | C | 0.012 | -0.057 | 0.010 | 4.10E-08 | 7.53E-05 | 33.169 |
| LDL cholesterol | rs28590710 | A | G | 0.197 | -0.018 | 0.003 | 1.40E-10 | 9.81E-05 | 43.232 |
| LDL cholesterol | rs7734476 | A | G | 0.550 | 0.019 | 0.002 | 3.30E-19 | 1.74E-04 | 76.754 |
| LDL cholesterol | rs6874202 | C | T | 0.634 | 0.032 | 0.002 | 1.60E-50 | 4.85E-04 | 213.749 |
| LDL cholesterol | rs116734477 | T | C | 0.041 | -0.047 | 0.005 | 3.20E-19 | 1.76E-04 | 77.346 |
| LDL cholesterol | rs1016988 | C | T | 0.191 | -0.017 | 0.003 | 4.80E-11 | 9.39E-05 | 41.387 |
| LDL cholesterol | rs3104412 | G | A | 0.452 | -0.019 | 0.002 | 1.80E-19 | 1.77E-04 | 78.008 |
| LDL cholesterol | rs2745353 | T | C | 0.518 | 0.013 | 0.002 | 1.10E-09 | 8.05E-05 | 35.463 |
| LDL cholesterol | rs7776054 | G | A | 0.261 | -0.016 | 0.002 | 8.20E-12 | 1.02E-04 | 44.813 |
| LDL cholesterol | rs12208357 | T | C | 0.070 | 0.057 | 0.004 | 5.40E-44 | 4.23E-04 | 186.586 |
| LDL cholesterol | rs146534110 | T | G | 0.013 | 0.068 | 0.009 | 7.80E-14 | 1.21E-04 | 53.521 |
| LDL cholesterol | rs118039278 | A | G | 0.079 | 0.084 | 0.004 | 1.80E-102 | 1.01E-03 | 445.784 |
| LDL cholesterol | rs7746081 | A | G | 0.304 | -0.023 | 0.002 | 4.00E-25 | 2.33E-04 | 102.757 |
| LDL cholesterol | rs9496567 | A | G | 0.243 | -0.017 | 0.002 | 7.80E-13 | 1.12E-04 | 49.317 |
| LDL cholesterol | rs79220007 | C | T | 0.076 | -0.057 | 0.004 | 2.00E-48 | 4.62E-04 | 203.844 |
| LDL cholesterol | rs3823376 | T | C | 0.502 | 0.017 | 0.002 | 1.00E-16 | 1.49E-04 | 65.762 |
| LDL cholesterol | rs34568880 | T | C | 0.013 | 0.056 | 0.009 | 6.80E-10 | 8.26E-05 | 36.406 |
| LDL cholesterol | rs35919498 | T | C | 0.117 | 0.046 | 0.003 | 1.20E-44 | 4.33E-04 | 190.991 |
| LDL cholesterol | rs3127580 | T | C | 0.155 | 0.036 | 0.003 | 2.20E-35 | 3.34E-04 | 147.253 |
| LDL cholesterol | rs3822855 | T | G | 0.402 | 0.018 | 0.002 | 4.50E-17 | 1.53E-04 | 67.417 |
| LDL cholesterol | rs117733303 | G | A | 0.018 | 0.084 | 0.008 | 2.40E-27 | 2.54E-04 | 111.960 |
| LDL cholesterol | rs2820226 | A | G | 0.452 | -0.013 | 0.002 | 8.60E-10 | 8.20E-05 | 36.117 |
| LDL cholesterol | rs9471968 | G | A | 0.546 | -0.012 | 0.002 | 2.90E-08 | 6.69E-05 | 29.469 |
| LDL cholesterol | rs869412 | C | T | 0.226 | -0.014 | 0.003 | 1.30E-08 | 7.08E-05 | 31.186 |
| LDL cholesterol | rs28406917 | T | C | 0.428 | 0.012 | 0.002 | 1.80E-08 | 6.94E-05 | 30.571 |
| LDL cholesterol | rs56130071 | C | G | 0.217 | 0.033 | 0.003 | 4.70E-39 | 3.74E-04 | 164.865 |
| LDL cholesterol | rs61003864 | C | T | 0.187 | 0.016 | 0.003 | 5.60E-09 | 7.40E-05 | 32.604 |
| LDL cholesterol | rs60612724 | G | A | 0.039 | 0.032 | 0.005 | 2.30E-09 | 7.79E-05 | 34.336 |
| LDL cholesterol | rs111338114 | G | A | 0.056 | -0.029 | 0.005 | 5.10E-09 | 8.76E-05 | 38.574 |
| LDL cholesterol | rs10231941 | C | T | 0.178 | 0.020 | 0.003 | 3.30E-13 | 1.15E-04 | 50.772 |
| LDL cholesterol | rs4148826 | C | T | 0.180 | -0.016 | 0.003 | 6.70E-09 | 7.38E-05 | 32.497 |
| LDL cholesterol | rs35990695 | C | A | 0.477 | 0.014 | 0.002 | 4.10E-11 | 9.74E-05 | 42.931 |
| LDL cholesterol | rs2073547 | G | A | 0.184 | 0.036 | 0.003 | 2.30E-40 | 3.80E-04 | 167.255 |
| LDL cholesterol | rs117139027 | A | G | 0.018 | -0.057 | 0.008 | 5.90E-13 | 1.13E-04 | 49.677 |
| LDL cholesterol | rs28615248 | C | T | 0.196 | 0.018 | 0.003 | 2.70E-12 | 1.07E-04 | 47.207 |
| LDL cholesterol | rs4738684 | G | A | 0.665 | -0.032 | 0.002 | 3.00E-46 | 4.44E-04 | 195.494 |
| LDL cholesterol | rs34207171 | C | A | 0.701 | -0.014 | 0.002 | 2.60E-08 | 8.09E-05 | 35.656 |
| LDL cholesterol | rs55831924 | T | C | 0.361 | 0.016 | 0.002 | 6.90E-13 | 1.13E-04 | 49.979 |
| LDL cholesterol | rs1350559 | G | C | 0.401 | 0.014 | 0.002 | 1.10E-10 | 9.13E-05 | 40.216 |
| LDL cholesterol | rs2737265 | G | A | 0.280 | -0.020 | 0.002 | 2.50E-18 | 1.66E-04 | 73.099 |
| LDL cholesterol | rs28601761 | G | C | 0.419 | -0.062 | 0.002 | 2.90E-185 | 1.87E-03 | 826.158 |
| LDL cholesterol | rs9987289 | G | A | 0.909 | 0.045 | 0.004 | 5.10E-36 | 3.41E-04 | 150.443 |
| LDL cholesterol | rs34265667 | A | G | 0.034 | -0.032 | 0.006 | 2.60E-08 | 6.75E-05 | 29.722 |
| LDL cholesterol | rs28814720 | G | A | 0.520 | 0.012 | 0.002 | 1.50E-08 | 7.40E-05 | 32.601 |
| LDL cholesterol | rs10448340 | G | T | 0.320 | -0.015 | 0.002 | 1.70E-11 | 9.87E-05 | 43.468 |
| LDL cholesterol | rs2519093 | T | C | 0.185 | 0.056 | 0.003 | 1.40E-95 | 9.35E-04 | 412.121 |
| LDL cholesterol | rs3780181 | G | A | 0.068 | -0.028 | 0.004 | 2.60E-11 | 9.82E-05 | 43.244 |
| LDL cholesterol | rs6475606 | T | C | 0.484 | -0.020 | 0.002 | 2.30E-22 | 2.05E-04 | 90.425 |
| LDL cholesterol | rs6560499 | A | G | 0.576 | -0.012 | 0.002 | 8.90E-09 | 7.27E-05 | 32.045 |
| LDL cholesterol | rs2066714 | C | T | 0.129 | 0.021 | 0.003 | 1.40E-11 | 9.91E-05 | 43.660 |
| LDL cholesterol | rs2740488 | C | A | 0.265 | -0.025 | 0.002 | 1.30E-26 | 2.49E-04 | 109.523 |
| LDL cholesterol | rs79828839 | T | C | 0.199 | 0.014 | 0.003 | 2.90E-08 | 6.70E-05 | 29.501 |
| LDL cholesterol | rs2068888 | A | G | 0.451 | -0.019 | 0.002 | 4.60E-20 | 1.83E-04 | 80.494 |
| LDL cholesterol | rs76468627 | T | C | 0.076 | -0.022 | 0.004 | 3.70E-08 | 6.62E-05 | 29.185 |
| LDL cholesterol | rs2250802 | A | G | 0.724 | -0.018 | 0.002 | 6.10E-15 | 1.32E-04 | 58.243 |
| LDL cholesterol | rs11014204 | T | C | 0.280 | 0.014 | 0.002 | 3.20E-09 | 7.67E-05 | 33.809 |
| LDL cholesterol | rs17476364 | C | T | 0.108 | -0.022 | 0.003 | 1.20E-10 | 9.05E-05 | 39.865 |
| LDL cholesterol | rs12246352 | G | A | 0.104 | 0.026 | 0.003 | 6.00E-14 | 1.23E-04 | 54.328 |
| LDL cholesterol | rs11601507 | A | C | 0.069 | 0.032 | 0.004 | 1.80E-15 | 1.34E-04 | 59.066 |
| LDL cholesterol | rs11226108 | C | G | 0.192 | -0.016 | 0.003 | 1.60E-09 | 7.95E-05 | 35.048 |
| LDL cholesterol | rs10832963 | G | T | 0.744 | 0.017 | 0.002 | 5.10E-13 | 1.14E-04 | 50.237 |
| LDL cholesterol | rs174564 | G | A | 0.349 | -0.032 | 0.002 | 4.50E-48 | 4.62E-04 | 203.652 |
| LDL cholesterol | rs964184 | C | G | 0.866 | -0.058 | 0.003 | 8.20E-79 | 7.68E-04 | 338.400 |
| LDL cholesterol | rs7108486 | C | T | 0.024 | -0.039 | 0.007 | 2.40E-08 | 6.90E-05 | 30.409 |
| LDL cholesterol | rs4930163 | A | G | 0.159 | 0.017 | 0.003 | 9.20E-10 | 8.17E-05 | 36.002 |
| LDL cholesterol | rs4307732 | A | G | 0.106 | 0.045 | 0.003 | 9.80E-40 | 3.81E-04 | 167.705 |
| LDL cholesterol | rs78447482 | C | T | 0.588 | -0.013 | 0.002 | 2.10E-09 | 7.78E-05 | 34.284 |
| LDL cholesterol | rs2160994 | C | T | 0.647 | 0.018 | 0.002 | 6.30E-17 | 1.52E-04 | 67.050 |
| LDL cholesterol | rs78508096 | A | G | 0.226 | 0.018 | 0.002 | 1.30E-12 | 1.10E-04 | 48.248 |
| LDL cholesterol | rs61754230 | T | C | 0.020 | 0.043 | 0.007 | 9.10E-09 | 7.18E-05 | 31.613 |
| LDL cholesterol | rs597808 | G | A | 0.516 | 0.027 | 0.002 | 2.10E-38 | 3.67E-04 | 161.744 |
| LDL cholesterol | rs35882350 | G | A | 0.261 | 0.014 | 0.002 | 4.00E-09 | 7.53E-05 | 33.167 |
| LDL cholesterol | rs11065385 | G | A | 0.691 | -0.024 | 0.002 | 2.00E-27 | 2.56E-04 | 112.887 |
| LDL cholesterol | rs2391825 | A | G | 0.279 | -0.013 | 0.002 | 2.60E-08 | 6.79E-05 | 29.900 |
| LDL cholesterol | rs2238162 | T | C | 0.523 | -0.017 | 0.002 | 2.80E-15 | 1.36E-04 | 59.857 |
| LDL cholesterol | rs6602912 | G | T | 0.285 | 0.022 | 0.002 | 7.20E-22 | 2.01E-04 | 88.474 |
| LDL cholesterol | rs11621792 | T | C | 0.453 | 0.019 | 0.002 | 1.10E-19 | 1.82E-04 | 80.066 |
| LDL cholesterol | rs60229127 | G | A | 0.557 | -0.013 | 0.002 | 2.40E-10 | 8.83E-05 | 38.905 |
| LDL cholesterol | rs61988556 | C | T | 0.086 | -0.022 | 0.004 | 1.70E-09 | 7.91E-05 | 34.854 |
| LDL cholesterol | rs145730801 | C | T | 0.044 | 0.036 | 0.005 | 3.30E-12 | 1.10E-04 | 48.430 |
| LDL cholesterol | rs35980001 | G | C | 0.787 | -0.022 | 0.003 | 3.80E-18 | 1.67E-04 | 73.499 |
| LDL cholesterol | rs2043085 | C | T | 0.612 | -0.017 | 0.002 | 1.90E-15 | 1.38E-04 | 60.588 |
| LDL cholesterol | rs6495122 | C | A | 0.591 | 0.014 | 0.002 | 1.20E-11 | 1.00E-04 | 44.067 |
| LDL cholesterol | rs12445804 | A | G | 0.075 | 0.023 | 0.004 | 9.50E-09 | 7.32E-05 | 32.267 |
| LDL cholesterol | rs7202323 | G | T | 0.230 | -0.026 | 0.002 | 6.50E-25 | 2.31E-04 | 101.699 |
| LDL cholesterol | rs4782568 | G | C | 0.451 | -0.017 | 0.002 | 4.90E-15 | 1.35E-04 | 59.441 |
| LDL cholesterol | rs183130 | T | C | 0.324 | -0.033 | 0.002 | 2.10E-49 | 4.76E-04 | 209.605 |
| LDL cholesterol | rs62033400 | G | A | 0.395 | -0.014 | 0.002 | 1.40E-11 | 9.93E-05 | 43.754 |
| LDL cholesterol | rs9929977 | A | T | 0.370 | 0.017 | 0.002 | 1.00E-14 | 1.30E-04 | 57.461 |
| LDL cholesterol | rs34042070 | G | C | 0.188 | 0.049 | 0.003 | 2.90E-73 | 7.19E-04 | 316.815 |
| LDL cholesterol | rs72631343 | G | C | 0.129 | -0.029 | 0.003 | 4.60E-21 | 1.93E-04 | 84.884 |
| LDL cholesterol | rs2611867 | G | A | 0.514 | -0.027 | 0.002 | 4.70E-37 | 3.53E-04 | 155.600 |
| LDL cholesterol | rs1801689 | C | A | 0.031 | 0.062 | 0.006 | 1.80E-24 | 2.27E-04 | 99.889 |
| LDL cholesterol | rs72911393 | T | C | 0.148 | -0.018 | 0.003 | 5.30E-10 | 8.39E-05 | 36.976 |
| LDL cholesterol | rs55714927 | T | C | 0.190 | -0.026 | 0.003 | 2.40E-23 | 2.15E-04 | 94.892 |
| LDL cholesterol | rs9894946 | G | A | 0.841 | -0.017 | 0.003 | 2.30E-09 | 8.03E-05 | 35.380 |
| LDL cholesterol | rs35278712 | T | G | 0.712 | 0.018 | 0.002 | 5.30E-15 | 1.34E-04 | 59.242 |
| LDL cholesterol | rs77542162 | G | A | 0.022 | 0.128 | 0.007 | 2.10E-74 | 7.26E-04 | 320.065 |
| LDL cholesterol | rs77960347 | G | A | 0.013 | 0.071 | 0.009 | 7.70E-15 | 1.31E-04 | 57.892 |
| LDL cholesterol | rs7241918 | T | G | 0.824 | 0.016 | 0.003 | 1.00E-08 | 7.19E-05 | 31.672 |
| LDL cholesterol | rs440677 | A | G | 0.623 | -0.016 | 0.002 | 1.50E-13 | 1.19E-04 | 52.477 |
| LDL cholesterol | rs35511051 | A | C | 0.210 | -0.022 | 0.003 | 1.10E-17 | 1.57E-04 | 69.386 |
| LDL cholesterol | rs2738447 | C | A | 0.593 | 0.042 | 0.002 | 2.90E-89 | 8.63E-04 | 380.458 |
| LDL cholesterol | rs1551891 | A | G | 0.088 | -0.174 | 0.004 | 1.00E-200 | 4.84E-03 | 2144.480 |
| LDL cholesterol | rs4263041 | G | A | 0.283 | -0.070 | 0.003 | 4.90E-171 | 1.99E-03 | 877.662 |
| LDL cholesterol | rs111273322 | A | G | 0.108 | 0.022 | 0.003 | 8.00E-11 | 9.16E-05 | 40.346 |
| LDL cholesterol | rs5112 | G | C | 0.533 | 0.028 | 0.002 | 9.40E-36 | 3.84E-04 | 169.327 |
| LDL cholesterol | rs516316 | C | G | 0.508 | 0.030 | 0.002 | 1.50E-46 | 4.39E-04 | 193.268 |
| LDL cholesterol | rs143020224 | G | C | 0.119 | -0.169 | 0.003 | 1.00E-200 | 5.97E-03 | 2644.922 |
| LDL cholesterol | rs8107974 | T | A | 0.076 | -0.105 | 0.004 | 2.70E-158 | 1.55E-03 | 682.025 |
| LDL cholesterol | rs200046586 | C | A | 0.981 | 0.309 | 0.008 | 1.00E-200 | 3.55E-03 | 1567.979 |
| LDL cholesterol | rs375972689 | G | T | 0.011 | 0.168 | 0.010 | 1.50E-61 | 5.99E-04 | 264.034 |
| LDL cholesterol | rs1010759 | A | G | 0.140 | -0.023 | 0.003 | 1.50E-14 | 1.29E-04 | 56.759 |
| LDL cholesterol | rs1883711 | C | G | 0.031 | 0.103 | 0.006 | 7.50E-64 | 6.43E-04 | 283.313 |
| LDL cholesterol | rs6031587 | T | C | 0.072 | -0.026 | 0.004 | 5.70E-10 | 8.79E-05 | 38.711 |
| LDL cholesterol | rs6074012 | C | T | 0.524 | 0.012 | 0.002 | 2.40E-08 | 6.77E-05 | 29.848 |
| LDL cholesterol | rs17569873 | T | C | 0.201 | 0.017 | 0.003 | 4.80E-11 | 9.42E-05 | 41.522 |
| LDL cholesterol | rs438568 | G | A | 0.609 | 0.012 | 0.002 | 5.50E-09 | 7.41E-05 | 32.660 |
| LDL cholesterol | rs2618566 | T | G | 0.660 | -0.025 | 0.002 | 9.60E-30 | 2.79E-04 | 122.840 |
| LDL cholesterol | rs200571020 | C | T | 0.541 | -0.026 | 0.002 | 1.80E-34 | 3.30E-04 | 145.365 |
| LDL cholesterol | rs2256814 | A | G | 0.198 | 0.015 | 0.003 | 6.10E-09 | 7.39E-05 | 32.576 |
| LDL cholesterol | rs5843957 | G | T | 0.571 | 0.014 | 0.002 | 8.10E-11 | 9.20E-05 | 40.554 |
| LDL cholesterol | rs960596 | T | C | 0.339 | 0.014 | 0.002 | 1.10E-09 | 8.18E-05 | 36.038 |
| LDL cholesterol | rs12162782 | G | T | 0.344 | 0.013 | 0.002 | 3.50E-09 | 7.58E-05 | 33.411 |
| Triglycerides | rs200610097 | C | A | 0.844 | -0.015 | 0.003 | 2.40E-08 | 6.31E-05 | 27.820 |
| Triglycerides | rs11206374 | A | G | 0.225 | 0.025 | 0.002 | 6.10E-26 | 2.18E-04 | 96.271 |
| Triglycerides | rs74090351 | A | G | 0.068 | -0.025 | 0.004 | 2.10E-10 | 7.94E-05 | 35.035 |
| Triglycerides | rs9436661 | G | T | 0.353 | -0.078 | 0.002 | 1.00E-200 | 2.76E-03 | 1220.445 |
| Triglycerides | rs34302257 | C | A | 0.649 | -0.012 | 0.002 | 2.70E-08 | 6.54E-05 | 28.863 |
| Triglycerides | rs1044808 | C | G | 0.081 | -0.025 | 0.004 | 8.30E-12 | 9.14E-05 | 40.328 |
| Triglycerides | rs1760801 | A | G | 0.296 | -0.020 | 0.002 | 1.50E-20 | 1.72E-04 | 75.707 |
| Triglycerides | rs9425589 | A | G | 0.567 | -0.014 | 0.002 | 6.20E-12 | 9.30E-05 | 41.020 |
| Triglycerides | rs1009590 | C | G | 0.915 | 0.021 | 0.004 | 5.70E-09 | 6.76E-05 | 29.799 |
| Triglycerides | rs114165349 | C | G | 0.023 | 0.082 | 0.007 | 6.30E-35 | 3.03E-04 | 133.586 |
| Triglycerides | rs213494 | T | C | 0.648 | 0.016 | 0.002 | 5.50E-14 | 1.11E-04 | 49.011 |
| Triglycerides | rs698927 | C | A | 0.184 | -0.018 | 0.003 | 9.20E-13 | 1.01E-04 | 44.343 |
| Triglycerides | rs320369 | G | A | 0.683 | -0.013 | 0.002 | 5.20E-09 | 6.80E-05 | 29.982 |
| Triglycerides | rs10631642 | C | T | 0.689 | 0.013 | 0.002 | 2.00E-09 | 7.39E-05 | 32.604 |
| Triglycerides | rs880315 | C | T | 0.340 | -0.012 | 0.002 | 2.20E-08 | 6.22E-05 | 27.439 |
| Triglycerides | rs36043408 | A | G | 0.502 | -0.013 | 0.002 | 1.10E-10 | 8.20E-05 | 36.171 |
| Triglycerides | rs61830291 | C | A | 0.096 | 0.029 | 0.003 | 1.80E-17 | 1.43E-04 | 63.149 |
| Triglycerides | rs11122450 | G | T | 0.612 | -0.048 | 0.002 | 1.30E-123 | 1.10E-03 | 486.730 |
| Triglycerides | rs2131311 | G | A | 0.715 | -0.012 | 0.002 | 2.50E-08 | 6.29E-05 | 27.740 |
| Triglycerides | rs112108602 | A | G | 0.910 | 0.027 | 0.003 | 1.10E-14 | 1.18E-04 | 52.131 |
| Triglycerides | rs1938566 | T | C | 0.835 | -0.021 | 0.003 | 2.00E-15 | 1.24E-04 | 54.812 |
| Triglycerides | rs11240358 | A | G | 0.394 | 0.014 | 0.002 | 2.30E-11 | 8.79E-05 | 38.757 |
| Triglycerides | rs11118310 | T | A | 0.593 | 0.019 | 0.002 | 1.10E-21 | 1.80E-04 | 79.426 |
| Triglycerides | rs1043897 | T | G | 0.416 | -0.015 | 0.002 | 3.40E-13 | 1.04E-04 | 46.070 |
| Triglycerides | rs1473886 | T | G | 0.478 | -0.018 | 0.002 | 6.90E-20 | 1.64E-04 | 72.281 |
| Triglycerides | rs954244 | G | C | 0.255 | 0.015 | 0.002 | 1.60E-11 | 8.91E-05 | 39.310 |
| Triglycerides | rs2382825 | T | C | 0.623 | -0.013 | 0.002 | 4.80E-11 | 8.53E-05 | 37.634 |
| Triglycerides | rs676210 | A | G | 0.205 | -0.074 | 0.002 | 7.10E-198 | 1.76E-03 | 779.120 |
| Triglycerides | rs4665972 | C | T | 0.605 | -0.100 | 0.002 | 1.00E-200 | 4.81E-03 | 2129.378 |
| Triglycerides | rs150419156 | A | G | 0.015 | -0.051 | 0.008 | 2.40E-09 | 7.68E-05 | 33.868 |
| Triglycerides | rs10210970 | T | C | 0.131 | 0.023 | 0.003 | 2.00E-15 | 1.23E-04 | 54.468 |
| Triglycerides | rs13389219 | T | C | 0.393 | -0.038 | 0.002 | 6.60E-77 | 6.75E-04 | 297.931 |
| Triglycerides | rs3731696 | G | A | 0.121 | 0.022 | 0.003 | 5.10E-13 | 1.02E-04 | 45.033 |
| Triglycerides | rs78058190 | A | G | 0.050 | 0.082 | 0.005 | 1.70E-57 | 6.38E-04 | 281.575 |
| Triglycerides | rs2943645 | T | C | 0.647 | 0.040 | 0.002 | 2.90E-84 | 7.42E-04 | 327.449 |
| Triglycerides | rs12475332 | G | T | 0.261 | -0.014 | 0.002 | 5.20E-10 | 7.56E-05 | 33.349 |
| Triglycerides | rs3820897 | C | T | 0.820 | 0.020 | 0.003 | 3.50E-14 | 1.15E-04 | 50.699 |
| Triglycerides | rs72784786 | A | G | 0.061 | 0.026 | 0.004 | 2.60E-10 | 7.91E-05 | 34.878 |
| Triglycerides | rs6752845 | C | G | 0.431 | -0.014 | 0.002 | 6.10E-12 | 9.32E-05 | 41.095 |
| Triglycerides | rs7424120 | T | C | 0.602 | -0.012 | 0.002 | 1.20E-09 | 7.32E-05 | 32.274 |
| Triglycerides | rs1009360 | C | T | 0.419 | -0.018 | 0.002 | 3.40E-20 | 1.66E-04 | 73.347 |
| Triglycerides | rs5832650 | C | A | 0.593 | 0.011 | 0.002 | 2.40E-08 | 6.13E-05 | 27.050 |
| Triglycerides | rs1420384 | T | G | 0.667 | -0.013 | 0.002 | 1.20E-09 | 7.27E-05 | 32.050 |
| Triglycerides | rs11904650 | G | A | 0.021 | 0.041 | 0.007 | 3.00E-09 | 6.93E-05 | 30.579 |
| Triglycerides | rs4128205 | C | A | 0.509 | 0.012 | 0.002 | 8.10E-09 | 6.61E-05 | 29.160 |
| Triglycerides | rs17326656 | T | G | 0.238 | 0.017 | 0.002 | 7.80E-14 | 1.10E-04 | 48.721 |
| Triglycerides | rs62135012 | A | G | 0.358 | -0.012 | 0.002 | 1.50E-08 | 6.34E-05 | 27.979 |
| Triglycerides | rs6432622 | G | A | 0.490 | -0.011 | 0.002 | 3.80E-08 | 5.92E-05 | 26.127 |
| Triglycerides | rs78545596 | A | T | 0.370 | -0.012 | 0.002 | 1.10E-08 | 6.85E-05 | 30.214 |
| Triglycerides | rs4675812 | A | G | 0.588 | -0.014 | 0.002 | 1.50E-12 | 9.81E-05 | 43.279 |
| Triglycerides | rs6800707 | G | C | 0.811 | 0.030 | 0.003 | 5.30E-32 | 2.74E-04 | 121.001 |
| Triglycerides | rs62271373 | A | T | 0.060 | 0.042 | 0.004 | 7.80E-23 | 1.99E-04 | 87.574 |
| Triglycerides | rs62274099 | T | C | 0.424 | 0.012 | 0.002 | 2.30E-09 | 7.14E-05 | 31.483 |
| Triglycerides | rs2137557 | C | T | 0.646 | 0.012 | 0.002 | 1.60E-08 | 6.33E-05 | 27.922 |
| Triglycerides | rs6805924 | T | G | 0.431 | 0.011 | 0.002 | 4.50E-08 | 5.90E-05 | 26.018 |
| Triglycerides | rs684773 | C | A | 0.767 | 0.029 | 0.002 | 2.50E-35 | 3.03E-04 | 133.725 |
| Triglycerides | rs3103310 | G | A | 0.242 | 0.020 | 0.002 | 1.10E-17 | 1.50E-04 | 66.348 |
| Triglycerides | rs9831084 | C | T | 0.462 | -0.012 | 0.002 | 2.90E-09 | 6.98E-05 | 30.795 |
| Triglycerides | rs9859117 | C | G | 0.203 | 0.015 | 0.002 | 2.40E-09 | 7.06E-05 | 31.159 |
| Triglycerides | rs73238173 | G | C | 0.130 | -0.017 | 0.003 | 1.90E-08 | 6.23E-05 | 27.462 |
| Triglycerides | rs10513688 | A | G | 0.097 | 0.025 | 0.003 | 1.40E-13 | 1.08E-04 | 47.632 |
| Triglycerides | rs79287178 | A | G | 0.031 | 0.050 | 0.006 | 7.20E-17 | 1.51E-04 | 66.813 |
| Triglycerides | rs11185542 | C | G | 0.728 | -0.013 | 0.002 | 1.60E-08 | 6.33E-05 | 27.910 |
| Triglycerides | rs34389637 | C | T | 0.935 | 0.025 | 0.004 | 3.80E-10 | 7.78E-05 | 34.305 |
| Triglycerides | rs6792725 | G | A | 0.692 | -0.015 | 0.002 | 6.20E-12 | 9.91E-05 | 43.717 |
| Triglycerides | rs55807798 | A | T | 0.421 | -0.012 | 0.002 | 6.80E-09 | 6.90E-05 | 30.432 |
| Triglycerides | rs13066793 | G | A | 0.090 | -0.022 | 0.003 | 1.00E-10 | 8.27E-05 | 36.473 |
| Triglycerides | rs57996145 | G | T | 0.833 | -0.029 | 0.003 | 1.50E-27 | 2.37E-04 | 104.758 |
| Triglycerides | rs12504746 | T | C | 0.193 | -0.015 | 0.003 | 1.40E-09 | 7.26E-05 | 32.006 |
| Triglycerides | rs2237029 | A | G | 0.601 | -0.014 | 0.002 | 8.30E-12 | 9.32E-05 | 41.092 |
| Triglycerides | rs13101504 | G | T | 0.413 | 0.020 | 0.002 | 4.50E-16 | 1.85E-04 | 81.815 |
| Triglycerides | rs1347188 | G | A | 0.246 | 0.014 | 0.002 | 1.90E-09 | 7.17E-05 | 31.604 |
| Triglycerides | rs71603401 | G | A | 0.137 | 0.026 | 0.003 | 1.10E-19 | 1.66E-04 | 73.152 |
| Triglycerides | rs11722924 | C | G | 0.535 | 0.013 | 0.002 | 8.90E-11 | 8.29E-05 | 36.568 |
| Triglycerides | rs13118477 | A | G | 0.393 | 0.015 | 0.002 | 1.90E-13 | 1.07E-04 | 47.267 |
| Triglycerides | rs148827772 | G | A | 0.023 | 0.047 | 0.007 | 5.70E-11 | 9.55E-05 | 42.123 |
| Triglycerides | rs11100083 | C | T | 0.226 | -0.016 | 0.002 | 1.50E-11 | 8.99E-05 | 39.650 |
| Triglycerides | rs2604568 | A | T | 0.664 | 0.012 | 0.002 | 2.80E-08 | 6.10E-05 | 26.908 |
| Triglycerides | rs278981 | C | T | 0.758 | 0.013 | 0.002 | 3.90E-08 | 5.81E-05 | 25.624 |
| Triglycerides | rs3775228 | T | C | 0.400 | 0.034 | 0.002 | 5.80E-62 | 5.49E-04 | 242.197 |
| Triglycerides | rs6532798 | T | C | 0.697 | 0.014 | 0.002 | 1.80E-10 | 8.03E-05 | 35.409 |
| Triglycerides | rs13108218 | G | A | 0.615 | -0.031 | 0.002 | 9.70E-50 | 4.41E-04 | 194.603 |
| Triglycerides | rs13107325 | T | C | 0.075 | 0.030 | 0.004 | 1.70E-15 | 1.25E-04 | 55.289 |
| Triglycerides | rs7735249 | G | C | 0.113 | 0.027 | 0.003 | 1.90E-17 | 1.44E-04 | 63.693 |
| Triglycerides | rs55646464 | T | G | 0.300 | 0.012 | 0.002 | 1.90E-08 | 6.24E-05 | 27.536 |
| Triglycerides | rs72644085 | C | T | 0.146 | -0.020 | 0.003 | 3.20E-12 | 9.59E-05 | 42.276 |
| Triglycerides | rs373798 | G | A | 0.917 | 0.032 | 0.004 | 5.30E-17 | 1.53E-04 | 67.511 |
| Triglycerides | rs193735 | A | G | 0.037 | 0.033 | 0.005 | 4.90E-10 | 7.68E-05 | 33.889 |
| Triglycerides | rs970069 | T | C | 0.212 | 0.016 | 0.002 | 2.60E-11 | 8.80E-05 | 38.833 |
| Triglycerides | rs6882076 | C | T | 0.634 | 0.033 | 0.002 | 4.90E-58 | 5.08E-04 | 224.293 |
| Triglycerides | rs62397245 | G | C | 0.222 | 0.015 | 0.002 | 3.90E-10 | 7.78E-05 | 34.292 |
| Triglycerides | rs11746801 | A | G | 0.637 | -0.012 | 0.002 | 2.70E-09 | 7.07E-05 | 31.186 |
| Triglycerides | rs4976033 | G | A | 0.402 | 0.018 | 0.002 | 4.30E-18 | 1.52E-04 | 67.130 |
| Triglycerides | rs7704653 | G | A | 0.723 | 0.016 | 0.002 | 2.30E-12 | 9.94E-05 | 43.839 |
| Triglycerides | rs72801474 | A | G | 0.092 | -0.031 | 0.003 | 3.60E-19 | 1.58E-04 | 69.773 |
| Triglycerides | rs13354321 | C | T | 0.410 | -0.015 | 0.002 | 1.90E-14 | 1.15E-04 | 50.799 |
| Triglycerides | rs1316753 | C | G | 0.394 | -0.015 | 0.002 | 7.00E-13 | 1.01E-04 | 44.733 |
| Triglycerides | rs7714361 | C | A | 0.234 | 0.014 | 0.002 | 4.40E-09 | 6.91E-05 | 30.456 |
| Triglycerides | rs1045241 | T | C | 0.271 | -0.021 | 0.002 | 3.20E-20 | 1.69E-04 | 74.660 |
| Triglycerides | rs11429307 | G | T | 0.809 | -0.047 | 0.003 | 2.70E-75 | 6.69E-04 | 295.352 |
| Triglycerides | rs325485 | G | A | 0.603 | -0.012 | 0.002 | 8.60E-09 | 6.60E-05 | 29.124 |
| Triglycerides | rs7244 | A | G | 0.174 | 0.015 | 0.003 | 6.40E-09 | 6.64E-05 | 29.304 |
| Triglycerides | rs78588343 | A | G | 0.176 | -0.016 | 0.003 | 2.50E-09 | 7.00E-05 | 30.855 |
| Triglycerides | rs28752924 | G | T | 0.243 | 0.024 | 0.003 | 1.10E-18 | 2.19E-04 | 96.559 |
| Triglycerides | rs186413375 | C | A | 0.083 | 0.038 | 0.004 | 2.90E-19 | 2.21E-04 | 97.368 |
| Triglycerides | rs138191773 | A | G | 0.017 | -0.047 | 0.008 | 2.60E-09 | 7.45E-05 | 32.840 |
| Triglycerides | rs9373056 | T | C | 0.320 | -0.012 | 0.002 | 7.20E-09 | 6.57E-05 | 28.976 |
| Triglycerides | rs17585887 | C | T | 0.591 | -0.029 | 0.002 | 1.30E-45 | 3.95E-04 | 174.266 |
| Triglycerides | rs9376511 | G | A | 0.203 | -0.015 | 0.002 | 3.20E-10 | 7.78E-05 | 34.320 |
| Triglycerides | rs4134963 | T | C | 0.190 | -0.019 | 0.003 | 9.00E-14 | 1.11E-04 | 48.750 |
| Triglycerides | rs28383314 | C | T | 0.624 | 0.038 | 0.002 | 1.50E-76 | 6.74E-04 | 297.432 |
| Triglycerides | rs77009508 | G | A | 0.074 | 0.045 | 0.004 | 1.80E-32 | 2.78E-04 | 122.515 |
| Triglycerides | rs9368503 | T | A | 0.510 | -0.012 | 0.002 | 1.80E-09 | 7.15E-05 | 31.539 |
| Triglycerides | rs998584 | A | C | 0.483 | 0.040 | 0.002 | 2.20E-90 | 8.04E-04 | 354.761 |
| Triglycerides | rs62427982 | T | C | 0.322 | -0.013 | 0.002 | 4.20E-10 | 7.74E-05 | 34.153 |
| Triglycerides | rs186696265 | T | C | 0.015 | -0.104 | 0.008 | 2.40E-36 | 3.15E-04 | 139.002 |
| Triglycerides | rs729761 | G | T | 0.712 | 0.018 | 0.002 | 9.50E-16 | 1.30E-04 | 57.244 |
| Triglycerides | rs1835346 | G | A | 0.024 | -0.039 | 0.007 | 2.20E-09 | 7.16E-05 | 31.568 |
| Triglycerides | rs35786744 | A | T | 0.953 | -0.027 | 0.005 | 1.90E-08 | 6.51E-05 | 28.733 |
| Triglycerides | rs1064173 | A | G | 0.284 | -0.021 | 0.002 | 1.80E-20 | 1.86E-04 | 81.904 |
| Triglycerides | rs2983896 | A | G | 0.215 | 0.014 | 0.002 | 1.20E-08 | 6.41E-05 | 28.291 |
| Triglycerides | rs9480889 | G | C | 0.783 | 0.016 | 0.002 | 1.30E-11 | 9.03E-05 | 39.836 |
| Triglycerides | rs6916318 | T | A | 0.531 | 0.027 | 0.002 | 4.20E-41 | 3.56E-04 | 156.999 |
| Triglycerides | rs73025562 | A | G | 0.246 | 0.014 | 0.002 | 2.00E-09 | 7.13E-05 | 31.435 |
| Triglycerides | rs852388 | C | G | 0.211 | 0.016 | 0.002 | 1.60E-10 | 8.23E-05 | 36.290 |
| Triglycerides | rs35140741 | A | C | 0.635 | -0.016 | 0.002 | 5.00E-14 | 1.13E-04 | 49.625 |
| Triglycerides | rs62459095 | T | C | 0.062 | -0.032 | 0.004 | 6.70E-14 | 1.19E-04 | 52.315 |
| Triglycerides | rs2240466 | A | G | 0.123 | -0.123 | 0.003 | 1.00E-200 | 3.25E-03 | 1437.881 |
| Triglycerides | rs41785 | A | C | 0.417 | -0.015 | 0.002 | 8.20E-14 | 1.10E-04 | 48.555 |
| Triglycerides | rs62473520 | C | T | 0.078 | -0.021 | 0.004 | 2.80E-08 | 6.40E-05 | 28.242 |
| Triglycerides | rs71538127 | G | C | 0.122 | 0.018 | 0.003 | 6.20E-09 | 6.68E-05 | 29.443 |
| Triglycerides | rs10242866 | T | C | 0.399 | 0.016 | 0.002 | 7.00E-15 | 1.20E-04 | 52.747 |
| Triglycerides | rs10642257 | A | C | 0.839 | 0.037 | 0.003 | 1.70E-42 | 3.70E-04 | 163.401 |
| Triglycerides | rs1799831 | T | C | 0.156 | 0.025 | 0.003 | 3.70E-19 | 1.58E-04 | 69.764 |
| Triglycerides | rs7786339 | T | C | 0.169 | 0.016 | 0.003 | 7.60E-10 | 7.49E-05 | 33.017 |
| Triglycerides | rs72555385 | G | A | 0.049 | 0.065 | 0.005 | 1.70E-45 | 3.98E-04 | 175.663 |
| Triglycerides | rs10277582 | T | C | 0.119 | -0.017 | 0.003 | 1.70E-08 | 6.31E-05 | 27.823 |
| Triglycerides | rs2699805 | A | G | 0.400 | -0.020 | 0.002 | 5.10E-23 | 1.94E-04 | 85.783 |
| Triglycerides | rs12669911 | C | A | 0.614 | -0.012 | 0.002 | 7.90E-09 | 6.66E-05 | 29.374 |
| Triglycerides | rs72603744 | C | A | 0.316 | -0.013 | 0.002 | 4.60E-09 | 6.78E-05 | 29.882 |
| Triglycerides | rs12530679 | G | A | 0.485 | -0.012 | 0.002 | 1.80E-09 | 7.35E-05 | 32.416 |
| Triglycerides | rs75721796 | A | G | 0.214 | 0.021 | 0.002 | 1.20E-17 | 1.49E-04 | 65.846 |
| Triglycerides | rs4731701 | T | C | 0.493 | -0.033 | 0.002 | 2.70E-60 | 5.31E-04 | 234.142 |
| Triglycerides | rs4841580 | C | T | 0.435 | -0.025 | 0.002 | 1.40E-34 | 2.96E-04 | 130.564 |
| Triglycerides | rs79153732 | T | C | 0.017 | 0.077 | 0.008 | 2.00E-24 | 2.05E-04 | 90.217 |
| Triglycerides | rs7000494 | C | G | 0.030 | 0.137 | 0.006 | 8.00E-121 | 1.09E-03 | 479.671 |
| Triglycerides | rs150564454 | A | G | 0.012 | -0.102 | 0.010 | 4.40E-26 | 2.37E-04 | 104.333 |
| Triglycerides | rs3860846 | T | C | 0.275 | 0.030 | 0.002 | 4.00E-40 | 3.52E-04 | 155.311 |
| Triglycerides | rs1544980 | C | T | 0.199 | 0.024 | 0.002 | 7.60E-22 | 1.81E-04 | 80.017 |
| Triglycerides | rs343 | A | C | 0.083 | -0.141 | 0.004 | 1.00E-200 | 3.05E-03 | 1350.021 |
| Triglycerides | rs2081687 | C | T | 0.663 | -0.026 | 0.002 | 7.60E-36 | 3.05E-04 | 134.639 |
| Triglycerides | rs6999569 | G | A | 0.471 | -0.086 | 0.002 | 1.00E-200 | 3.69E-03 | 1634.667 |
| Triglycerides | rs13264304 | G | C | 0.151 | 0.019 | 0.003 | 8.00E-12 | 9.17E-05 | 40.444 |
| Triglycerides | rs308 | G | T | 0.021 | -0.159 | 0.007 | 2.60E-115 | 1.03E-03 | 452.627 |
| Triglycerides | rs13269725 | G | A | 0.078 | 0.035 | 0.004 | 2.00E-21 | 1.77E-04 | 78.043 |
| Triglycerides | rs3808477 | T | C | 0.279 | -0.013 | 0.002 | 1.20E-09 | 7.21E-05 | 31.812 |
| Triglycerides | rs11434143 | G | T | 0.811 | 0.016 | 0.003 | 6.10E-10 | 7.76E-05 | 34.237 |
| Triglycerides | rs75609851 | A | G | 0.010 | -0.199 | 0.010 | 1.60E-87 | 8.22E-04 | 362.942 |
| Triglycerides | rs2407278 | G | A | 0.030 | -0.034 | 0.006 | 5.40E-09 | 6.74E-05 | 29.724 |
| Triglycerides | rs28439112 | A | T | 0.256 | 0.013 | 0.002 | 2.70E-08 | 6.04E-05 | 26.646 |
| Triglycerides | rs2244278 | A | C | 0.121 | -0.027 | 0.003 | 1.20E-18 | 1.54E-04 | 68.137 |
| Triglycerides | rs76645731 | G | A | 0.319 | 0.013 | 0.002 | 3.10E-09 | 7.01E-05 | 30.896 |
| Triglycerides | rs1567353 | G | C | 0.308 | 0.015 | 0.002 | 7.40E-12 | 9.36E-05 | 41.268 |
| Triglycerides | rs581080 | C | G | 0.819 | 0.018 | 0.003 | 1.10E-11 | 9.12E-05 | 40.243 |
| Triglycerides | rs696825 | T | C | 0.253 | -0.020 | 0.002 | 7.50E-19 | 1.55E-04 | 68.581 |
| Triglycerides | rs10797119 | C | T | 0.537 | 0.016 | 0.002 | 4.60E-15 | 1.23E-04 | 54.146 |
| Triglycerides | rs2131919 | G | A | 0.164 | 0.017 | 0.003 | 1.00E-10 | 8.27E-05 | 36.458 |
| Triglycerides | rs2519093 | T | C | 0.185 | -0.021 | 0.003 | 1.50E-16 | 1.35E-04 | 59.429 |
| Triglycerides | rs7847285 | C | T | 0.589 | -0.012 | 0.002 | 1.00E-08 | 6.53E-05 | 28.780 |
| Triglycerides | rs10811662 | A | G | 0.173 | -0.015 | 0.003 | 4.60E-09 | 6.80E-05 | 29.997 |
| Triglycerides | rs7861679 | T | C | 0.697 | 0.012 | 0.002 | 1.80E-08 | 6.29E-05 | 27.748 |
| Triglycerides | rs11187019 | G | A | 0.551 | -0.012 | 0.002 | 6.40E-09 | 6.71E-05 | 29.591 |
| Triglycerides | rs10822163 | G | C | 0.473 | -0.032 | 0.002 | 9.70E-59 | 5.16E-04 | 227.638 |
| Triglycerides | rs7077812 | C | T | 0.195 | 0.014 | 0.003 | 1.40E-08 | 6.35E-05 | 28.022 |
| Triglycerides | rs2068888 | A | G | 0.451 | -0.032 | 0.002 | 3.20E-57 | 5.01E-04 | 221.231 |
| Triglycerides | rs56397607 | G | A | 0.183 | 0.017 | 0.003 | 1.10E-11 | 9.12E-05 | 40.242 |
| Triglycerides | rs80276949 | A | G | 0.023 | 0.046 | 0.007 | 8.00E-12 | 9.28E-05 | 40.931 |
| Triglycerides | rs113344423 | A | G | 0.060 | 0.043 | 0.004 | 4.10E-24 | 2.14E-04 | 94.182 |
| Triglycerides | rs55767272 | C | A | 0.065 | -0.028 | 0.004 | 2.80E-12 | 9.68E-05 | 42.683 |
| Triglycerides | rs11000468 | T | C | 0.255 | -0.015 | 0.002 | 1.50E-10 | 8.39E-05 | 37.002 |
| Triglycerides | rs34672664 | T | G | 0.930 | 0.022 | 0.004 | 3.10E-08 | 6.10E-05 | 26.883 |
| Triglycerides | rs2487294 | T | G | 0.723 | 0.018 | 0.002 | 1.60E-16 | 1.34E-04 | 59.225 |
| Triglycerides | rs140107293 | G | A | 0.155 | -0.023 | 0.003 | 1.60E-16 | 1.35E-04 | 59.446 |
| Triglycerides | rs3758413 | C | T | 0.418 | 0.011 | 0.002 | 3.50E-08 | 6.02E-05 | 26.545 |
| Triglycerides | rs10883026 | T | C | 0.522 | -0.014 | 0.002 | 5.50E-13 | 1.04E-04 | 46.028 |
| Triglycerides | rs2773469 | G | A | 0.733 | -0.019 | 0.002 | 5.70E-17 | 1.39E-04 | 61.445 |
| Triglycerides | rs1133400 | G | A | 0.220 | 0.014 | 0.002 | 9.80E-09 | 6.48E-05 | 28.595 |
| Triglycerides | rs499293 | A | G | 0.658 | -0.012 | 0.002 | 1.70E-08 | 6.26E-05 | 27.595 |
| Triglycerides | rs174566 | G | A | 0.350 | 0.049 | 0.002 | 2.80E-120 | 1.07E-03 | 472.386 |
| Triglycerides | rs480823 | C | T | 0.079 | 0.156 | 0.004 | 1.00E-200 | 3.53E-03 | 1561.611 |
| Triglycerides | rs150423652 | T | G | 0.007 | 0.286 | 0.012 | 1.40E-119 | 1.08E-03 | 478.292 |
| Triglycerides | rs551243 | C | G | 0.469 | 0.014 | 0.002 | 4.90E-13 | 1.03E-04 | 45.526 |
| Triglycerides | rs10899490 | T | C | 0.161 | -0.017 | 0.003 | 2.70E-10 | 7.83E-05 | 34.532 |
| Triglycerides | rs61905078 | C | A | 0.074 | 0.200 | 0.004 | 1.00E-200 | 5.47E-03 | 2423.609 |
| Triglycerides | rs150555490 | T | C | 0.057 | -0.038 | 0.004 | 2.50E-19 | 1.58E-04 | 69.853 |
| Triglycerides | rs61729990 | A | C | 0.018 | -0.056 | 0.008 | 1.10E-13 | 1.10E-04 | 48.337 |
| Triglycerides | rs117291242 | T | C | 0.037 | 0.030 | 0.005 | 1.70E-08 | 6.25E-05 | 27.546 |
| Triglycerides | rs117287238 | A | G | 0.028 | -0.039 | 0.006 | 1.40E-10 | 8.52E-05 | 37.569 |
| Triglycerides | rs75268115 | G | A | 0.085 | -0.021 | 0.004 | 6.80E-09 | 6.67E-05 | 29.425 |
| Triglycerides | rs326222 | C | T | 0.698 | 0.025 | 0.002 | 1.40E-31 | 2.68E-04 | 118.303 |
| Triglycerides | rs11600815 | A | G | 0.052 | -0.032 | 0.005 | 2.10E-12 | 1.02E-04 | 45.015 |
| Triglycerides | rs2302263 | T | C | 0.089 | 0.044 | 0.003 | 8.00E-36 | 3.08E-04 | 135.868 |
| Triglycerides | rs7947951 | G | A | 0.689 | 0.019 | 0.002 | 1.40E-19 | 1.61E-04 | 70.948 |
| Triglycerides | rs11030107 | G | A | 0.261 | 0.016 | 0.002 | 1.20E-12 | 9.92E-05 | 43.758 |
| Triglycerides | rs3974807 | T | C | 0.189 | 0.016 | 0.003 | 2.80E-10 | 7.83E-05 | 34.554 |
| Triglycerides | rs10750766 | A | C | 0.710 | 0.019 | 0.002 | 7.80E-19 | 1.55E-04 | 68.265 |
| Triglycerides | rs78484485 | A | G | 0.054 | -0.076 | 0.004 | 1.20E-66 | 5.88E-04 | 259.471 |
| Triglycerides | rs79357714 | G | A | 0.046 | -0.029 | 0.005 | 1.20E-09 | 7.31E-05 | 32.249 |
| Triglycerides | rs75942983 | T | A | 0.084 | -0.020 | 0.004 | 2.50E-08 | 6.21E-05 | 27.392 |
| Triglycerides | rs10773049 | C | T | 0.395 | -0.029 | 0.002 | 4.10E-46 | 4.03E-04 | 177.881 |
| Triglycerides | rs67981690 | G | A | 0.130 | 0.030 | 0.003 | 8.30E-24 | 2.02E-04 | 89.270 |
| Triglycerides | rs4760254 | C | G | 0.239 | -0.028 | 0.002 | 1.00E-33 | 2.88E-04 | 127.110 |
| Triglycerides | rs139386986 | T | C | 0.092 | -0.022 | 0.004 | 1.10E-09 | 7.87E-05 | 34.711 |
| Triglycerides | rs4761234 | C | T | 0.484 | -0.014 | 0.002 | 2.00E-12 | 9.82E-05 | 43.327 |
| Triglycerides | rs117233107 | A | G | 0.015 | -0.073 | 0.009 | 1.80E-17 | 1.60E-04 | 70.708 |
| Triglycerides | rs10773000 | T | G | 0.332 | -0.015 | 0.002 | 1.70E-12 | 9.89E-05 | 43.622 |
| Triglycerides | rs7308584 | A | G | 0.184 | 0.015 | 0.003 | 5.30E-09 | 6.77E-05 | 29.851 |
| Triglycerides | rs7134375 | A | C | 0.431 | -0.017 | 0.002 | 1.20E-17 | 1.44E-04 | 63.588 |
| Triglycerides | rs7135509 | C | T | 0.293 | -0.012 | 0.002 | 4.10E-08 | 6.10E-05 | 26.913 |
| Triglycerides | rs35763453 | C | T | 0.059 | 0.028 | 0.004 | 6.20E-11 | 8.94E-05 | 39.417 |
| Triglycerides | rs12424054 | A | G | 0.232 | 0.019 | 0.002 | 4.50E-16 | 1.30E-04 | 57.542 |
| Triglycerides | rs4765148 | T | G | 0.313 | -0.025 | 0.002 | 9.70E-32 | 2.72E-04 | 119.969 |
| Triglycerides | rs11274835 | T | C | 0.174 | -0.025 | 0.003 | 4.30E-20 | 1.86E-04 | 82.109 |
| Triglycerides | rs149778057 | C | A | 0.328 | -0.016 | 0.002 | 1.50E-12 | 1.10E-04 | 48.298 |
| Triglycerides | rs7400002 | G | A | 0.231 | 0.014 | 0.002 | 3.60E-09 | 6.93E-05 | 30.545 |
| Triglycerides | rs7140110 | C | T | 0.298 | 0.028 | 0.002 | 1.20E-38 | 3.36E-04 | 148.104 |
| Triglycerides | rs6562773 | G | A | 0.548 | -0.012 | 0.002 | 2.00E-09 | 7.20E-05 | 31.751 |
| Triglycerides | rs9561643 | C | A | 0.315 | 0.017 | 0.002 | 6.50E-15 | 1.21E-04 | 53.154 |
| Triglycerides | rs1340819 | C | A | 0.345 | -0.012 | 0.002 | 6.30E-09 | 6.69E-05 | 29.501 |
| Triglycerides | rs2812208 | C | G | 0.021 | -0.048 | 0.007 | 3.50E-12 | 9.59E-05 | 42.311 |
| Triglycerides | rs9584870 | C | T | 0.366 | -0.012 | 0.002 | 4.30E-09 | 7.09E-05 | 31.257 |
| Triglycerides | rs2070341 | T | C | 0.603 | 0.011 | 0.002 | 2.80E-08 | 6.11E-05 | 26.967 |
| Triglycerides | rs2240533 | C | T | 0.310 | -0.013 | 0.002 | 1.60E-09 | 7.22E-05 | 31.831 |
| Triglycerides | rs61993685 | C | T | 0.076 | -0.023 | 0.004 | 3.90E-10 | 7.73E-05 | 34.113 |
| Triglycerides | rs6572807 | G | A | 0.267 | 0.012 | 0.002 | 2.90E-08 | 6.09E-05 | 26.874 |
| Triglycerides | rs12880341 | C | T | 0.159 | 0.021 | 0.003 | 1.80E-14 | 1.18E-04 | 51.875 |
| Triglycerides | rs56902258 | A | T | 0.196 | -0.015 | 0.003 | 1.70E-09 | 7.23E-05 | 31.901 |
| Triglycerides | rs139974673 | C | T | 0.026 | 0.143 | 0.006 | 2.20E-116 | 1.04E-03 | 459.946 |
| Triglycerides | rs11637681 | G | A | 0.276 | 0.013 | 0.002 | 2.00E-08 | 6.34E-05 | 27.942 |
| Triglycerides | rs150460588 | C | T | 0.044 | 0.033 | 0.005 | 1.60E-11 | 9.08E-05 | 40.030 |
| Triglycerides | rs2043085 | C | T | 0.612 | -0.031 | 0.002 | 1.90E-51 | 4.51E-04 | 198.777 |
| Triglycerides | rs1077835 | G | A | 0.220 | 0.047 | 0.002 | 2.20E-86 | 7.71E-04 | 340.355 |
| Triglycerides | rs12440800 | T | A | 0.255 | 0.016 | 0.002 | 2.20E-12 | 9.85E-05 | 43.455 |
| Triglycerides | rs12902047 | C | A | 0.314 | -0.013 | 0.002 | 1.70E-09 | 7.20E-05 | 31.738 |
| Triglycerides | rs10152471 | A | G | 0.389 | -0.014 | 0.002 | 4.00E-11 | 8.69E-05 | 38.316 |
| Triglycerides | rs275184 | G | T | 0.162 | -0.017 | 0.003 | 2.90E-10 | 8.17E-05 | 36.051 |
| Triglycerides | rs17184382 | C | A | 0.425 | -0.022 | 0.002 | 1.10E-27 | 2.35E-04 | 103.663 |
| Triglycerides | rs1037117 | A | G | 0.255 | 0.017 | 0.002 | 5.70E-14 | 1.13E-04 | 49.674 |
| Triglycerides | rs143076454 | A | G | 0.019 | 0.040 | 0.007 | 3.90E-08 | 5.97E-05 | 26.345 |
| Triglycerides | rs12926107 | G | A | 0.455 | 0.013 | 0.002 | 2.70E-10 | 7.92E-05 | 34.927 |
| Triglycerides | rs2937124 | T | C | 0.363 | -0.018 | 0.002 | 8.10E-18 | 1.54E-04 | 67.966 |
| Triglycerides | rs12928099 | A | C | 0.296 | -0.028 | 0.002 | 2.90E-38 | 3.32E-04 | 146.461 |
| Triglycerides | rs3814883 | T | C | 0.482 | 0.015 | 0.002 | 8.70E-14 | 1.11E-04 | 48.852 |
| Triglycerides | rs12446515 | T | C | 0.323 | -0.033 | 0.002 | 3.50E-55 | 4.88E-04 | 215.496 |
| Triglycerides | rs34682685 | A | G | 0.104 | 0.034 | 0.003 | 5.80E-25 | 2.13E-04 | 93.758 |
| Triglycerides | rs4471666 | G | T | 0.069 | -0.022 | 0.004 | 1.50E-08 | 6.52E-05 | 28.774 |
| Triglycerides | rs28577186 | A | G | 0.665 | -0.016 | 0.002 | 1.20E-14 | 1.19E-04 | 52.521 |
| Triglycerides | rs2925979 | C | T | 0.700 | -0.032 | 0.002 | 8.50E-50 | 4.36E-04 | 192.252 |
| Triglycerides | rs742036 | A | G | 0.375 | -0.014 | 0.002 | 2.50E-12 | 9.67E-05 | 42.664 |
| Triglycerides | rs112381903 | T | A | 0.067 | 0.023 | 0.004 | 7.50E-09 | 6.74E-05 | 29.716 |
| Triglycerides | rs117431393 | G | A | 0.035 | 0.031 | 0.005 | 6.50E-09 | 6.67E-05 | 29.405 |
| Triglycerides | rs12185242 | C | A | 0.455 | 0.018 | 0.002 | 1.80E-18 | 1.52E-04 | 67.193 |
| Triglycerides | rs1801689 | C | A | 0.031 | -0.066 | 0.006 | 2.40E-30 | 2.59E-04 | 114.244 |
| Triglycerides | rs60856912 | T | G | 0.163 | 0.025 | 0.003 | 4.80E-20 | 1.68E-04 | 74.186 |
| Triglycerides | rs9889402 | A | G | 0.728 | 0.012 | 0.002 | 3.60E-08 | 5.97E-05 | 26.339 |
| Triglycerides | rs4969179 | G | T | 0.604 | -0.018 | 0.002 | 2.80E-18 | 1.51E-04 | 66.473 |
| Triglycerides | rs12948505 | T | C | 0.194 | 0.014 | 0.003 | 3.20E-08 | 6.04E-05 | 26.618 |
| Triglycerides | rs2304969 | T | G | 0.145 | -0.016 | 0.003 | 1.50E-08 | 6.46E-05 | 28.501 |
| Triglycerides | rs1292065 | G | C | 0.709 | -0.014 | 0.002 | 1.90E-10 | 8.00E-05 | 35.266 |
| Triglycerides | rs11078597 | C | T | 0.187 | 0.019 | 0.003 | 5.70E-14 | 1.12E-04 | 49.210 |
| Triglycerides | rs9902027 | T | C | 0.774 | -0.015 | 0.002 | 2.10E-10 | 8.03E-05 | 35.425 |
| Triglycerides | rs7215055 | G | A | 0.063 | 0.039 | 0.004 | 2.30E-21 | 1.79E-04 | 78.771 |
| Triglycerides | rs75634664 | G | C | 0.060 | 0.036 | 0.004 | 6.40E-17 | 1.48E-04 | 65.081 |
| Triglycerides | rs10775406 | G | A | 0.760 | 0.021 | 0.002 | 6.60E-19 | 1.56E-04 | 68.721 |
| Triglycerides | rs145947882 | C | A | 0.026 | 0.137 | 0.006 | 3.40E-103 | 9.54E-04 | 420.918 |
| Triglycerides | rs11664106 | T | A | 0.374 | -0.013 | 0.002 | 2.40E-09 | 7.41E-05 | 32.703 |
| Triglycerides | rs867939 | A | G | 0.576 | -0.014 | 0.002 | 1.80E-11 | 9.04E-05 | 39.869 |
| Triglycerides | rs7239575 | C | T | 0.490 | -0.016 | 0.002 | 6.80E-16 | 1.29E-04 | 56.979 |
| Triglycerides | rs2187114 | A | G | 0.101 | -0.019 | 0.003 | 2.10E-08 | 6.23E-05 | 27.462 |
| Triglycerides | rs6506033 | T | C | 0.073 | -0.023 | 0.004 | 2.50E-09 | 7.05E-05 | 31.100 |
| Triglycerides | rs921971 | C | T | 0.266 | 0.016 | 0.002 | 4.00E-12 | 9.56E-05 | 42.172 |
| Triglycerides | rs62102718 | T | A | 0.286 | 0.020 | 0.002 | 3.90E-20 | 1.67E-04 | 73.790 |
| Triglycerides | rs5112 | G | C | 0.533 | 0.068 | 0.002 | 1.00E-200 | 2.33E-03 | 1030.954 |
| Triglycerides | rs117316645 | A | G | 0.042 | 0.028 | 0.005 | 2.10E-08 | 6.23E-05 | 27.456 |
| Triglycerides | rs62112763 | G | C | 0.439 | 0.019 | 0.002 | 5.60E-22 | 1.84E-04 | 81.346 |
| Triglycerides | rs483808 | T | C | 0.702 | -0.014 | 0.002 | 4.00E-10 | 7.81E-05 | 34.453 |
| Triglycerides | rs62117489 | A | C | 0.056 | -0.043 | 0.004 | 3.60E-23 | 1.95E-04 | 85.947 |
| Triglycerides | rs2305746 | G | A | 0.933 | 0.028 | 0.004 | 1.20E-12 | 9.94E-05 | 43.826 |
| Triglycerides | rs8102873 | T | C | 0.585 | 0.012 | 0.002 | 9.40E-10 | 7.38E-05 | 32.560 |
| Triglycerides | rs10405944 | C | T | 0.483 | -0.013 | 0.002 | 6.20E-11 | 8.71E-05 | 38.433 |
| Triglycerides | rs483082 | T | G | 0.235 | 0.086 | 0.002 | 1.00E-200 | 2.68E-03 | 1182.925 |
| Triglycerides | rs116843064 | A | G | 0.019 | -0.227 | 0.007 | 1.00E-200 | 1.94E-03 | 859.354 |
| Triglycerides | rs58542926 | T | C | 0.075 | -0.103 | 0.004 | 5.30E-164 | 1.47E-03 | 649.318 |
| Triglycerides | rs188247550 | T | C | 0.013 | -0.134 | 0.009 | 5.90E-49 | 4.67E-04 | 205.990 |
| Triglycerides | rs62128802 | T | C | 0.183 | -0.016 | 0.003 | 7.40E-10 | 7.54E-05 | 33.265 |
| Triglycerides | rs2081194 | C | G | 0.601 | -0.021 | 0.002 | 6.30E-25 | 2.20E-04 | 96.994 |
| Triglycerides | rs71368855 | T | C | 0.115 | 0.026 | 0.003 | 7.80E-17 | 1.38E-04 | 61.073 |
| Triglycerides | rs2092203 | T | C | 0.481 | 0.014 | 0.002 | 6.80E-12 | 9.34E-05 | 41.185 |
| Triglycerides | rs6073958 | C | T | 0.199 | 0.056 | 0.002 | 1.60E-110 | 9.88E-04 | 436.011 |
| Triglycerides | rs8126001 | T | C | 0.490 | -0.016 | 0.002 | 2.40E-16 | 1.34E-04 | 59.129 |
| Triglycerides | rs149142833 | T | C | 0.156 | 0.017 | 0.003 | 9.10E-10 | 7.57E-05 | 33.374 |
| Triglycerides | rs6028716 | A | G | 0.258 | -0.013 | 0.002 | 2.00E-08 | 6.30E-05 | 27.790 |
| Triglycerides | rs55966194 | G | C | 0.282 | -0.018 | 0.002 | 7.10E-16 | 1.29E-04 | 56.915 |
| Triglycerides | rs151235402 | T | C | 0.016 | 0.052 | 0.008 | 1.50E-10 | 8.48E-05 | 37.416 |
| Triglycerides | rs7274718 | A | G | 0.599 | 0.016 | 0.002 | 3.80E-15 | 1.22E-04 | 53.920 |
| Triglycerides | rs394872 | T | C | 0.536 | 0.011 | 0.002 | 2.40E-08 | 6.22E-05 | 27.422 |
| Triglycerides | rs6517522 | C | T | 0.498 | -0.013 | 0.002 | 9.40E-11 | 8.32E-05 | 36.715 |
| Triglycerides | rs140288 | A | G | 0.567 | -0.013 | 0.002 | 4.00E-11 | 8.64E-05 | 38.098 |
| Triglycerides | rs134551 | T | C | 0.335 | -0.012 | 0.002 | 3.40E-08 | 6.05E-05 | 26.704 |
| Triglycerides | rs2267373 | T | C | 0.581 | 0.022 | 0.002 | 1.40E-26 | 2.26E-04 | 99.887 |
| Triglycerides | rs5755799 | G | C | 0.454 | 0.012 | 0.002 | 3.00E-09 | 7.00E-05 | 30.888 |
| Triglycerides | rs2071887 | A | T | 0.345 | 0.016 | 0.002 | 7.30E-15 | 1.20E-04 | 53.026 |
| Triglycerides | rs4253750 | C | T | 0.214 | 0.018 | 0.002 | 3.20E-13 | 1.06E-04 | 46.860 |
| Glycated hemoglobin | rs1800562 | A | G | 0.046 | -0.038 | 0.003 | 2.33E-50 | 1.29E-04 | 18.903 |
| Glycated hemoglobin | rs13389076 | A | G | 0.034 | 0.033 | 0.004 | 3.04E-18 | 7.24E-05 | 10.630 |
| Glycated hemoglobin | rs7903146 | T | C | 0.307 | 0.013 | 0.001 | 1.04E-22 | 7.53E-05 | 11.050 |
| Glycated hemoglobin | rs6474359 | C | T | 0.022 | -0.043 | 0.004 | 1.91E-33 | 7.85E-05 | 11.519 |
| Glycated hemoglobin | rs17533945 | C | T | 0.418 | 0.013 | 0.001 | 1.62E-23 | 7.97E-05 | 11.704 |
| Glycated hemoglobin | rs4760682 | A | C | 0.817 | 0.016 | 0.002 | 3.20E-20 | 8.04E-05 | 11.808 |
| Glycated hemoglobin | rs117233107 | A | G | 0.020 | -0.047 | 0.007 | 8.45E-11 | 8.66E-05 | 12.713 |
| Glycated hemoglobin | rs12612492 | T | C | 0.148 | 0.019 | 0.002 | 1.88E-26 | 8.91E-05 | 13.087 |
| Glycated hemoglobin | rs13134327 | A | G | 0.331 | 0.014 | 0.001 | 2.81E-26 | 9.18E-05 | 13.483 |
| Glycated hemoglobin | rs11558471 | G | A | 0.293 | -0.015 | 0.001 | 3.38E-25 | 9.45E-05 | 13.869 |
| Glycated hemoglobin | rs76533333 | G | A | 0.087 | 0.027 | 0.003 | 2.81E-29 | 1.12E-04 | 16.379 |
| Glycated hemoglobin | rs17476364 | C | T | 0.099 | -0.086 | 0.002 | 1.00E-200 | 1.31E-03 | 193.052 |
| Glycated hemoglobin | rs837763 | T | C | 0.578 | 0.018 | 0.001 | 5.20E-38 | 1.51E-04 | 22.187 |
| Glycated hemoglobin | rs10830963 | G | C | 0.286 | 0.020 | 0.002 | 1.54E-36 | 1.58E-04 | 23.272 |
| Glycated hemoglobin | rs855791 | G | A | 0.600 | -0.019 | 0.001 | 1.34E-56 | 1.70E-04 | 24.910 |
| Glycated hemoglobin | rs857725 | G | T | 0.277 | 0.021 | 0.001 | 5.43E-55 | 1.73E-04 | 25.444 |
| Glycated hemoglobin | rs4737009 | A | G | 0.262 | 0.023 | 0.002 | 8.29E-56 | 2.01E-04 | 29.518 |
| Glycated hemoglobin | rs9376090 | C | T | 0.272 | -0.025 | 0.001 | 1.90E-62 | 2.42E-04 | 35.479 |
| Glycated hemoglobin | rs2613522 | G | A | 0.260 | 0.027 | 0.002 | 9.34E-39 | 2.81E-04 | 41.193 |
| Glycated hemoglobin | rs2971670 | T | C | 0.181 | 0.032 | 0.002 | 5.10E-88 | 2.96E-04 | 43.474 |
| Glycated hemoglobin | rs560887 | C | T | 0.694 | 0.031 | 0.001 | 5.55E-122 | 4.00E-04 | 58.789 |
| Glycated hemoglobin | rs9909940 | T | C | 0.323 | 0.032 | 0.001 | 1.43E-116 | 4.53E-04 | 66.599 |
| Fasting insulin | rs6674544 | A | G | 0.574 | 0.018 | 0.002 | 6.97E-21 | 1.53E-04 | 23.141 |
| Fasting insulin | rs13389219 | T | C | 0.409 | -0.020 | 0.002 | 5.84E-28 | 1.91E-04 | 28.916 |
| Fasting insulin | rs5017305 | T | A | 0.764 | -0.014 | 0.003 | 1.07E-08 | 6.77E-05 | 10.221 |
| Fasting insulin | rs1260326 | C | T | 0.587 | 0.023 | 0.002 | 8.42E-38 | 2.59E-04 | 39.081 |
| Fasting insulin | rs2943646 | G | A | 0.623 | 0.025 | 0.002 | 8.47E-39 | 2.94E-04 | 44.348 |
| Fasting insulin | rs17036126 | T | C | 0.129 | 0.021 | 0.003 | 1.28E-10 | 9.82E-05 | 14.825 |
| Fasting insulin | rs35000407 | G | T | 0.117 | -0.026 | 0.003 | 1.50E-21 | 1.38E-04 | 20.772 |
| Fasting insulin | rs62271373 | A | T | 0.059 | 0.026 | 0.005 | 1.60E-08 | 7.28E-05 | 10.990 |
| Fasting insulin | rs10865959 | C | G | 0.300 | 0.014 | 0.002 | 1.99E-08 | 8.00E-05 | 12.080 |
| Fasting insulin | rs3775380 | G | A | 0.500 | 0.012 | 0.002 | 1.48E-11 | 7.08E-05 | 10.693 |
| Fasting insulin | rs9884482 | C | T | 0.392 | 0.013 | 0.002 | 2.88E-11 | 7.45E-05 | 11.248 |
| Fasting insulin | rs6855363 | C | T | 0.347 | -0.013 | 0.002 | 4.04E-08 | 7.08E-05 | 10.694 |
| Fasting insulin | rs4865796 | A | G | 0.707 | 0.017 | 0.002 | 7.33E-17 | 1.13E-04 | 17.035 |
| Fasting insulin | rs459193 | G | A | 0.715 | 0.018 | 0.002 | 1.12E-18 | 1.34E-04 | 20.165 |
| Fasting insulin | rs1474696 | G | A | 0.476 | 0.015 | 0.002 | 3.02E-16 | 1.08E-04 | 16.280 |
| Fasting insulin | rs73013411 | A | C | 0.120 | -0.018 | 0.003 | 2.08E-08 | 6.84E-05 | 10.334 |
| Fasting insulin | rs2780215 | G | A | 0.042 | -0.039 | 0.006 | 1.06E-09 | 1.24E-04 | 18.676 |
| Fasting insulin | rs116141873 | T | G | 0.032 | 0.043 | 0.006 | 1.42E-11 | 1.13E-04 | 17.140 |
| Fasting insulin | rs7012814 | A | G | 0.471 | -0.022 | 0.002 | 8.34E-30 | 2.39E-04 | 36.100 |
| Fasting insulin | rs75179845 | C | T | 0.079 | 0.022 | 0.004 | 6.05E-11 | 6.79E-05 | 10.253 |
| Fasting insulin | rs118164457 | C | T | 0.037 | 0.035 | 0.006 | 3.86E-10 | 8.48E-05 | 12.810 |
| Fasting insulin | rs6487237 | A | C | 0.790 | 0.015 | 0.003 | 4.68E-09 | 7.87E-05 | 11.884 |
| Fasting insulin | rs860598 | A | G | 0.824 | 0.018 | 0.003 | 6.88E-12 | 9.09E-05 | 13.723 |
| Fasting insulin | rs7133378 | A | G | 0.339 | -0.013 | 0.002 | 6.00E-11 | 7.23E-05 | 10.916 |
| Fasting insulin | rs12454712 | C | T | 0.398 | -0.014 | 0.003 | 1.78E-09 | 9.66E-05 | 14.593 |
| Fasting glucose | rs6662924 | A | C | 0.198 | 0.014 | 0.002 | 3.34E-10 | 6.49E-05 | 13.030 |
| Fasting glucose | rs78132593 | A | C | 0.203 | -0.015 | 0.002 | 2.60E-10 | 6.99E-05 | 14.029 |
| Fasting glucose | rs2075423 | T | G | 0.376 | -0.016 | 0.002 | 3.18E-21 | 1.22E-04 | 24.405 |
| Fasting glucose | rs348330 | A | G | 0.631 | -0.012 | 0.002 | 3.04E-10 | 6.93E-05 | 13.906 |
| Fasting glucose | rs537183 | T | C | 0.640 | 0.066 | 0.002 | 1.00E-200 | 2.03E-03 | 407.187 |
| Fasting glucose | rs1260326 | C | T | 0.587 | 0.028 | 0.002 | 4.48E-65 | 3.86E-04 | 77.385 |
| Fasting glucose | rs2461385 | C | T | 0.852 | -0.022 | 0.002 | 2.73E-19 | 1.19E-04 | 23.827 |
| Fasting glucose | rs1057394 | A | G | 0.625 | -0.012 | 0.002 | 1.91E-12 | 7.21E-05 | 14.461 |
| Fasting glucose | rs77981966 | T | C | 0.055 | -0.025 | 0.004 | 1.58E-14 | 6.29E-05 | 12.621 |
| Fasting glucose | rs189548 | A | G | 0.723 | -0.012 | 0.002 | 2.81E-09 | 6.06E-05 | 12.158 |
| Fasting glucose | rs17437560 | T | C | 0.110 | -0.018 | 0.003 | 3.33E-08 | 6.00E-05 | 12.031 |
| Fasting glucose | rs11708067 | G | A | 0.177 | -0.028 | 0.002 | 1.63E-43 | 2.30E-04 | 46.163 |
| Fasting glucose | rs16851397 | G | A | 0.045 | -0.033 | 0.004 | 1.26E-12 | 9.19E-05 | 18.440 |
| Fasting glucose | rs1604038 | T | C | 0.288 | -0.020 | 0.002 | 4.47E-28 | 1.61E-04 | 32.261 |
| Fasting glucose | rs6808574 | C | T | 0.606 | 0.013 | 0.002 | 7.21E-14 | 7.70E-05 | 15.453 |
| Fasting glucose | rs4862423 | T | C | 0.401 | 0.012 | 0.002 | 4.45E-10 | 7.27E-05 | 14.582 |
| Fasting glucose | rs7708285 | A | G | 0.686 | -0.013 | 0.002 | 1.25E-09 | 7.62E-05 | 15.290 |
| Fasting glucose | rs1820176 | C | T | 0.296 | -0.025 | 0.002 | 1.91E-34 | 2.54E-04 | 51.024 |
| Fasting glucose | rs157512 | C | T | 0.270 | -0.013 | 0.002 | 5.43E-10 | 7.08E-05 | 14.201 |
| Fasting glucose | rs10305457 | T | C | 0.065 | 0.024 | 0.003 | 1.21E-14 | 6.71E-05 | 13.468 |
| Fasting glucose | rs12055786 | T | C | 0.384 | 0.012 | 0.002 | 1.17E-11 | 6.81E-05 | 13.668 |
| Fasting glucose | rs3778321 | A | G | 0.176 | -0.019 | 0.002 | 3.16E-17 | 1.00E-04 | 20.133 |
| Fasting glucose | rs9348441 | A | T | 0.272 | 0.018 | 0.002 | 4.40E-20 | 1.23E-04 | 24.614 |
| Fasting glucose | rs878521 | A | G | 0.249 | 0.055 | 0.002 | 2.65E-174 | 1.13E-03 | 226.401 |
| Fasting glucose | rs10487796 | A | T | 0.484 | -0.026 | 0.002 | 4.62E-52 | 3.40E-04 | 68.285 |
| Fasting glucose | rs2595701 | G | A | 0.683 | -0.019 | 0.002 | 4.48E-19 | 1.55E-04 | 31.037 |
| Fasting glucose | rs58925536 | T | C | 0.032 | 0.031 | 0.005 | 5.82E-09 | 5.80E-05 | 11.639 |
| Fasting glucose | rs194518 | A | G | 0.520 | 0.010 | 0.002 | 8.76E-09 | 5.19E-05 | 10.420 |
| Fasting glucose | rs17168486 | T | C | 0.176 | 0.028 | 0.002 | 4.17E-36 | 2.27E-04 | 45.631 |
| Fasting glucose | rs7012637 | A | G | 0.470 | -0.018 | 0.002 | 9.75E-25 | 1.61E-04 | 32.389 |
| Fasting glucose | rs12541643 | T | C | 0.479 | 0.012 | 0.002 | 4.51E-09 | 6.95E-05 | 13.943 |
| Fasting glucose | rs9650069 | T | C | 0.284 | -0.029 | 0.002 | 8.31E-58 | 3.33E-04 | 66.759 |
| Fasting glucose | rs16913693 | G | T | 0.029 | -0.039 | 0.005 | 2.82E-16 | 8.74E-05 | 17.541 |
| Fasting glucose | rs10974438 | C | A | 0.380 | 0.020 | 0.002 | 9.85E-31 | 1.85E-04 | 37.067 |
| Fasting glucose | rs10811660 | A | G | 0.165 | -0.022 | 0.002 | 7.94E-25 | 1.37E-04 | 27.494 |
| Fasting glucose | rs507666 | A | G | 0.189 | 0.016 | 0.002 | 6.99E-17 | 8.25E-05 | 16.543 |
| Fasting glucose | rs3829109 | A | G | 0.276 | -0.016 | 0.002 | 1.09E-15 | 1.06E-04 | 21.305 |
| Fasting glucose | rs7095788 | T | C | 0.358 | -0.011 | 0.002 | 1.98E-09 | 5.16E-05 | 10.362 |
| Fasting glucose | rs7903146 | T | C | 0.307 | 0.026 | 0.002 | 1.99E-35 | 2.85E-04 | 57.280 |
| Fasting glucose | rs2839671 | A | G | 0.164 | -0.016 | 0.002 | 8.38E-14 | 7.02E-05 | 14.084 |
| Fasting glucose | rs12784552 | G | A | 0.076 | -0.033 | 0.003 | 2.86E-31 | 1.52E-04 | 30.503 |
| Fasting glucose | rs174583 | T | C | 0.375 | -0.017 | 0.002 | 3.37E-22 | 1.32E-04 | 26.546 |
| Fasting glucose | rs3842753 | G | T | 0.720 | -0.013 | 0.002 | 2.84E-09 | 7.24E-05 | 14.526 |
| Fasting glucose | rs10830963 | G | C | 0.286 | 0.077 | 0.002 | 1.00E-200 | 2.43E-03 | 489.510 |
| Fasting glucose | rs10838693 | C | G | 0.314 | 0.018 | 0.002 | 3.44E-23 | 1.35E-04 | 27.081 |
| Fasting glucose | rs11603349 | C | T | 0.168 | -0.024 | 0.002 | 3.12E-25 | 1.56E-04 | 31.241 |
| Fasting glucose | rs10838524 | G | A | 0.520 | -0.024 | 0.002 | 1.56E-40 | 2.83E-04 | 56.745 |
| Fasting glucose | rs6489811 | G | A | 0.512 | 0.011 | 0.002 | 3.27E-09 | 6.05E-05 | 12.131 |
| Fasting glucose | rs6538804 | G | C | 0.377 | -0.014 | 0.002 | 9.41E-14 | 9.47E-05 | 19.004 |
| Fasting glucose | rs11610045 | A | G | 0.454 | 0.014 | 0.002 | 3.26E-13 | 1.03E-04 | 20.626 |
| Fasting glucose | rs11619319 | G | A | 0.234 | 0.017 | 0.002 | 3.41E-20 | 1.07E-04 | 21.527 |
| Fasting glucose | rs35889227 | T | G | 0.617 | -0.013 | 0.002 | 3.37E-10 | 7.99E-05 | 16.025 |
| Fasting glucose | rs12888855 | A | C | 0.189 | -0.014 | 0.002 | 6.02E-12 | 5.59E-05 | 11.209 |
| Fasting glucose | rs7163757 | T | C | 0.433 | -0.022 | 0.002 | 2.64E-36 | 2.31E-04 | 46.398 |
| Fasting glucose | rs7178572 | G | A | 0.679 | 0.012 | 0.002 | 7.09E-10 | 6.38E-05 | 12.805 |
| Fasting glucose | rs6598541 | G | A | 0.648 | -0.011 | 0.002 | 4.12E-12 | 5.93E-05 | 11.895 |
| Fasting glucose | rs2238435 | G | C | 0.619 | -0.011 | 0.002 | 3.82E-09 | 5.92E-05 | 11.871 |
| Fasting glucose | rs2302593 | G | C | 0.495 | -0.011 | 0.002 | 5.67E-10 | 5.62E-05 | 11.270 |
| Fasting glucose | rs6113722 | A | G | 0.066 | -0.042 | 0.004 | 7.66E-25 | 2.22E-04 | 44.476 |
| Fasting glucose | rs17265513 | C | T | 0.204 | 0.016 | 0.002 | 5.10E-14 | 8.11E-05 | 16.267 |

**Abbreviation: SNPs**: single nucleotide polymorphisms; **EA**: effect allele; **OA**: other allele; **SE**: standard error; **HDL**, high density lipoprotein; **LDL**, low density lipoprotein

**Supplementary Table 2**. Specific information for univariable MR analysis of dietary factor and metabolic factor as exposure and migraine as outcome

| **Exposure** | **Method** | **SNPs** | **Beta** | **SE** | **OR** | **95%CI** | **p-value** |
| --- | --- | --- | --- | --- | --- | --- | --- |
| Beef intake | MR Egger | 14 | 24.770 | 14.013 | 5.72E+10 | 0.068-4.84E+22 | 0.103 |
|  | Weighted median | 14 | 4.446 | 3.132 | 85.244 | 0.184-3.95E+04 | 0.156 |
|  | Inverse variance weighted | 14 | 3.718 | 2.330 | 41.194 | 0.428-3.96E+03 | 0.111 |
|  | Simple mode | 14 | 3.967 | 4.839 | 52.830 | 0.004-6.95E+05 | 0.427 |
|  | Weighted mode | 14 | 4.200 | 5.011 | 66.656 | 0.004-1.23E+06 | 0.417 |
| Pork intake | MR Egger | 13 | 10.923 | 18.114 | 5.54E+04 | 2.11E-11-1.45E+20 | 0.559 |
|  | Weighted median | 13 | 5.553 | 3.953 | 258.098 | 0.111-5.98E+05 | 0.160 |
|  | Inverse variance weighted | 13 | 1.064 | 2.824 | 2.898 | 0.011-734.261 | 0.706 |
|  | Simple mode | 13 | 6.160 | 6.531 | 473.493 | 0.001-1.72E+08 | 0.364 |
|  | Weighted mode | 13 | 6.267 | 6.150 | 527.008 | 0.003-9.06E+07 | 0.328 |
| Poultry intake | MR Egger | 7 | -145.614 | 124.378 | 5.76291E-64 | 7.7224E-170-4.30E+42 | 0.294 |
|  | Weighted median | 7 | -4.201 | 4.750 | 0.015 | 1.36E-06-1.66E+02 | 0.376 |
|  | Inverse variance weighted | 7 | -2.405 | 4.256 | 0.090 | 2.15E-05-3.79E+02 | 0.572 |
|  | Simple mode | 7 | -4.839 | 7.448 | 0.008 | 3.62E-09-1.73E+04 | 0.540 |
|  | Weighted mode | 7 | -4.705 | 7.568 | 0.009 | 3.27E-09-2.50E+04 | 0.557 |
| Oily fish intake | MR Egger | 61 | -2.722 | 4.607 | 0.066 | 7.87E-06-548.583 | 0.557 |
|  | Weighted median | 61 | -0.598 | 1.345 | 0.550 | 0.039-7.675 | 0.657 |
|  | Inverse variance weighted | 61 | -0.145 | 1.085 | 0.865 | 0.103-7.258 | 0.894 |
|  | Simple mode | 61 | -0.702 | 3.337 | 0.496 | 0.001-343.305 | 0.834 |
|  | Weighted mode | 61 | -2.088 | 2.847 | 0.124 | 4.67E-04-32.832 | 0.466 |
| Non-oily fish intake | MR Egger | 11 | -12.638 | 14.899 | 3.25E-06 | 0.012-3.71E+04 | 0.418 |
|  | Weighted median | 11 | 3.030 | 3.822 | 20.690 | 0.028-6.02E+03 | 0.428 |
|  | Inverse variance weighted | 11 | 2.559 | 3.135 | 12.918 | 1.43E-04-6.66E+06 | 0.414 |
|  | Simple mode | 11 | 3.431 | 6.266 | 30.904 | 9.17E-05-5.57E+05 | 0.596 |
|  | Weighted mode | 11 | 1.967 | 5.747 | 7.150 | 0.012-3.71E+04 | 0.739 |
| Processed meat intake | MR Egger | 23 | -0.432 | 7.693 | 0.649 | 1.84E-07-2.29E+06 | 0.956 |
|  | Weighted median | 23 | -1.564 | 2.178 | 0.209 | 2.93E-03-1.49E+01 | 0.473 |
|  | Inverse variance weighted | 23 | -1.546 | 1.517 | 0.213 | 1.09E-02-4.17E+00 | 0.308 |
|  | Simple mode | 23 | -4.459 | 4.423 | 0.012 | 1.99E-06-6.73E+01 | 0.324 |
|  | Weighted mode | 23 | -4.361 | 4.037 | 0.013 | 4.68E-06-3.48E+01 | 0.292 |
| Tea intake | MR Egger | 40 | 0.436 | 1.965 | 1.546 | 0.033-72.721 | 0.826 |
|  | Weighted median | 40 | 1.256 | 1.411 | 3.511 | 0.221-55.783 | 0.373 |
|  | Inverse variance weighted | 40 | -0.453 | 0.895 | 0.636 | 0.110-3.676 | 0.613 |
|  | Simple mode | 40 | 1.030 | 2.478 | 2.802 | 0.022-360.088 | 0.680 |
|  | Weighted mode | 40 | 1.198 | 1.360 | 3.312 | 0.230-47.628 | 0.384 |
| Water intake | MR Egger | 37 | -2.979 | 3.446 | 0.051 | 5.93E-05-43.606 | 0.393 |
|  | Weighted median | 37 | -2.672 | 1.807 | 0.069 | 0.002-2.387 | 0.139 |
|  | Inverse variance weighted | 37 | -1.411 | 1.199 | 0.244 | 0.023-2.555 | 0.239 |
|  | Simple mode | 37 | -3.885 | 3.344 | 0.021 | 2.92E-05-14.438 | 0.253 |
|  | Weighted mode | 37 | -2.919 | 2.202 | 0.054 | 0.001-4.044 | 0.193 |
| Alcohol intake frequency | MR Egger | 95 | -0.741 | 1.447 | 0.477 | 0.028-8.127 | 0.610 |
|  | Weighted median | 95 | 0.452 | 0.725 | 1.571 | 0.379-6.511 | 0.533 |
|  | Inverse variance weighted | 95 | 0.438 | 0.467 | 1.550 | 0.620-3.875 | 0.348 |
|  | Simple mode | 95 | 1.530 | 1.737 | 4.617 | 0.153-138.880 | 0.381 |
|  | Weighted mode | 95 | 0.646 | 1.293 | 1.908 | 0.151-24.049 | 0.618 |
| Bread intake | MR Egger | 27 | 0.434 | 6.672 | 1.544 | 3.23E-06-7.38E+05 | 0.949 |
|  | Weighted median | 27 | -0.906 | 2.017 | 0.404 | 0.008-21.054 | 0.653 |
|  | Inverse variance weighted | 27 | -1.364 | 1.411 | 0.256 | 0.016-4.061 | 0.334 |
|  | Simple mode | 27 | -4.931 | 4.138 | 0.007 | 2.17E-06-24.022 | 0.244 |
|  | Weighted mode | 27 | 0.408 | 3.614 | 1.504 | 0.001-1.79E+03 | 0.911 |
| Cheese intake | MR Egger | 61 | 2.666 | 3.761 | 14.380 | 0.009-2.29E+04 | 0.481 |
|  | Weighted median | 61 | 1.011 | 1.281 | 2.747 | 0.223-33.849 | 0.430 |
|  | Inverse variance weighted | 61 | 0.643 | 0.885 | 1.902 | 0.336-10.779 | 0.467 |
|  | Simple mode | 61 | -0.346 | 3.050 | 0.708 | 0.002-279.272 | 0.910 |
|  | Weighted mode | 61 | 0.937 | 2.391 | 2.551 | 0.024-276.815 | 0.697 |
| Cereal intake | MR Egger | 39 | -3.768 | 6.317 | 0.023 | 9.69E-08-5.50E+03 | 0.554 |
|  | Weighted median | 39 | -2.084 | 1.864 | 0.124 | 0.003-4.802 | 0.264 |
|  | Inverse variance weighted | 39 | -1.027 | 1.451 | 0.358 | 0.021-6.157 | 0.479 |
|  | Simple mode | 39 | -3.926 | 3.738 | 0.020 | 1.30E-05-30.003 | 0.300 |
|  | Weighted mode | 39 | -3.130 | 3.157 | 0.044 | 8.98E-05-21.282 | 0.328 |
| Dried fruit intake | MR Egger | 41 | -6.826 | 5.923 | 0.001 | 9.86E-09-119.461 | 0.256 |
|  | Weighted median | 41 | -2.915 | 1.888 | 0.054 | 0.001-2.194 | 0.123 |
|  | Inverse variance weighted | 41 | -1.180 | 1.318 | 0.307 | 0.023-4.071 | 0.371 |
|  | Simple mode | 41 | -5.419 | 4.445 | 0.004 | 7.29E-07-26.928 | 0.230 |
|  | Weighted mode | 41 | -4.625 | 3.619 | 0.010 | 8.14E-06-11.801 | 0.209 |
| Fresh fruit intake | MR Egger | 41 | -6.257 | 5.810 | 0.002 | 2.17E-08-169.071 | 0.288 |
|  | Weighted median | 41 | -4.200 | 2.589 | 0.015 | 9.37E-05-2.399 | 0.105 |
|  | Inverse variance weighted | 41 | -3.754 | 1.721 | 0.023 | 0.001-0.683 | 0.029 |
|  | Simple mode | 41 | -4.978 | 5.173 | 0.007 | 0.000-174.343 | 0.342 |
|  | Weighted mode | 41 | -5.503 | 3.923 | 0.004 | 0.000-8.905 | 0.168 |
| Cooked vegetable intake | MR Egger | 17 | -28.478 | 28.734 | 4.29E-13 | 1.49E-37-1.23E+12 | 0.337 |
|  | Weighted median | 17 | -1.955 | 3.382 | 0.142 | 1.87E-04-106.963 | 0.563 |
|  | Inverse variance weighted | 17 | -0.425 | 2.611 | 0.654 | 0.004-109.196 | 0.871 |
|  | Simple mode | 17 | -3.632 | 5.939 | 0.026 | 2.33E-07-3.01E+03 | 0.549 |
|  | Weighted mode | 17 | -3.047 | 5.062 | 0.047 | 2.33E-06-967.747 | 0.556 |
| Salad/raw vegetable intake | MR Egger | 18 | -17.885 | 15.143 | 1.71E-08 | 2.20E-21-1.32E+05 | 0.255 |
|  | Weighted median | 18 | -1.400 | 4.024 | 0.247 | 9.26E-05-6.57E+02 | 0.728 |
|  | Inverse variance weighted | 18 | -1.905 | 3.205 | 0.149 | 2.79E-04-7.95E+01 | 0.552 |
|  | Simple mode | 18 | -2.423 | 6.569 | 0.089 | 2.27E-07-3.46E+04 | 0.717 |
|  | Weighted mode | 18 | -1.413 | 6.699 | 0.243 | 4.83E-07-1.23E+05 | 0.835 |
| Body mass index | MR Egger | 424 | 2.046 | 0.787 | 7.735 | 1.653-36.197 | 0.010 |
|  | Weighted median | 424 | 0.992 | 0.520 | 2.696 | 0.974-7.463 | 0.056 |
|  | Inverse variance weighted | 424 | 0.992 | 0.291 | 2.696 | 1.524-4.770 | 0.001 |
|  | Simple mode | 424 | 1.957 | 1.314 | 7.077 | 0.539-92.926 | 0.137 |
|  | Weighted mode | 424 | 1.075 | 0.861 | 2.930 | 0.542-15.850 | 0.213 |
| Waist circumference | MR Egger | 351 | 1.415 | 1.047 | 4.115 | 0.528-32.065 | 0.178 |
|  | Weighted median | 351 | 1.052 | 0.654 | 2.864 | 0.796-10.311 | 0.107 |
|  | Inverse variance weighted | 351 | 1.097 | 0.368 | 2.995 | 1.457-6.156 | 0.003 |
|  | Simple mode | 351 | 1.280 | 1.675 | 3.598 | 0.135-95.868 | 0.445 |
|  | Weighted mode | 351 | 1.166 | 1.042 | 3.211 | 0.416-24.770 | 0.264 |
| Hypothyroidism | MR Egger | 68 | 0.420 | 0.283 | 1.522 | 0.873-2.651 | 0.143 |
|  | Weighted median | 68 | 0.537 | 0.212 | 1.711 | 1.128-2.595 | 0.012 |
|  | Inverse variance weighted | 68 | 0.290 | 0.132 | 1.337 | 1.033-1.730 | 0.027 |
|  | Simple mode | 68 | 0.657 | 0.448 | 1.930 | 0.802-4.644 | 0.147 |
|  | Weighted mode | 68 | 0.550 | 0.275 | 1.733 | 1.011-2.973 | 0.050 |
| Hyperthyroidism | MR Egger | 11 | 1.122 | 0.372 | 3.071 | 1.482-6.363 | 0.015 |
|  | Weighted median | 11 | 0.761 | 0.209 | 2.141 | 1.421-3.228 | 2.74E-04 |
|  | Inverse variance weighted | 11 | 0.807 | 0.157 | 2.240 | 1.649-3.045 | 2.55E-07 |
|  | Simple mode | 11 | 0.513 | 0.312 | 1.670 | 0.907-3.076 | 0.131 |
|  | Weighted mode | 11 | 0.789 | 0.247 | 2.200 | 1.356-3.571 | 0.010 |
| HDL cholesterol | MR Egger | 326 | 0.435 | 0.329 | 1.545 | 0.811-2.942 | 0.186 |
|  | Weighted median | 326 | 0.092 | 0.371 | 1.096 | 0.530-2.266 | 0.805 |
|  | Inverse variance weighted | 326 | -0.026 | 0.217 | 0.974 | 0.637-1.491 | 0.905 |
|  | Simple mode | 326 | -0.454 | 0.635 | 0.635 | 0.183-2.202 | 0.475 |
|  | Weighted mode | 326 | 0.030 | 0.339 | 1.030 | 0.530-2.002 | 0.930 |
| LDL cholesterol | MR Egger | 156 | 0.126 | 0.420 | 1.134 | 0.498-2.583 | 0.764 |
|  | Weighted median | 156 | 0.250 | 0.442 | 1.284 | 0.540-3.052 | 0.571 |
|  | Inverse variance weighted | 156 | -0.180 | 0.286 | 0.835 | 0.477-1.463 | 0.529 |
|  | Simple mode | 156 | -0.427 | 0.852 | 0.652 | 0.123-3.467 | 0.617 |
|  | Weighted mode | 156 | 0.180 | 0.392 | 1.197 | 0.555-2.583 | 0.647 |
| Triglycerides | MR Egger | 284 | -0.058 | 0.330 | 0.944 | 0.494-1.802 | 0.861 |
|  | Weighted median | 284 | 0.130 | 0.383 | 1.139 | 0.538-2.413 | 0.734 |
|  | Inverse variance weighted | 284 | 0.280 | 0.223 | 1.323 | 0.854-2.049 | 0.210 |
|  | Simple mode | 284 | -0.459 | 0.734 | 0.632 | 0.150-2.663 | 0.532 |
|  | Weighted mode | 284 | 0.037 | 0.330 | 1.038 | 0.543-1.983 | 0.911 |
| Glycated hemoglobin | MR Egger | 22 | -0.693 | 2.413 | 0.500 | 0.004-56.597 | 0.777 |
|  | Weighted median | 22 | 0.339 | 1.485 | 1.403 | 0.076-25.795 | 0.820 |
|  | Inverse variance weighted | 22 | -0.468 | 1.023 | 0.626 | 0.084-4.648 | 0.647 |
|  | Simple mode | 22 | 0.799 | 2.418 | 2.223 | 0.019-254.063 | 0.744 |
|  | Weighted mode | 22 | 0.944 | 2.029 | 2.569 | 0.048-137.098 | 0.647 |
| Fasting insulin | MR Egger | 25 | -2.514 | 5.332 | 0.081 | 2.34E-06-2.80E+03 | 0.642 |
|  | Weighted median | 25 | -0.938 | 1.846 | 0.391 | 0.010-14.593 | 0.611 |
|  | Inverse variance weighted | 25 | 0.323 | 1.465 | 1.381 | 0.078-24.400 | 0.825 |
|  | Simple mode | 25 | -1.390 | 2.946 | 0.249 | 0.001-80.246 | 0.641 |
|  | Weighted mode | 25 | -1.173 | 2.425 | 0.309 | 0.003-35.884 | 0.633 |
| Fasting glucose | MR Egger | 57 | 0.163 | 1.085 | 1.177 | 0.141-9.866 | 0.881 |
|  | Weighted median | 57 | 0.415 | 0.910 | 1.515 | 0.255-9.013 | 0.648 |
|  | Inverse variance weighted | 57 | -0.258 | 0.595 | 0.773 | 0.241-2.480 | 0.665 |
|  | Simple mode | 57 | -0.885 | 1.675 | 0.413 | 0.015-11.006 | 0.600 |
|  | Weighted mode | 57 | 0.077 | 0.870 | 1.080 | 0.196-5.942 | 0.930 |

**Abbreviation: SNPs**: single nucleotide polymorphisms; **EA**: effect allele; **OA**: other allele; **SE**: standard error; **HDL**, high density lipoprotein; **LDL**, low density lipoprotein
